# Bayesian optimization for enzyme-catalyzed, site-selective acetylation of carbohydrate OH groups

**DOI:** 10.1039/d6sc06368c

**Published:** 2026-11-04

**Authors:** Katrina H. Ruzicka, Matthew T. Zambri, Jolene P. Reid, Mark S. Taylor

**Affiliations:** a Department of Chemistry, University of Toronto 80 St. George St. Toronto Ontario M5S 3H6 Canada mtaylor@chem.utoronto.ca; b Department of Chemistry, University of British Columbia 2036 Main Mall Vancouver British Columbia V6T 1Z1 Canada jreid@chem.ubc.ca

## Abstract

In this study, we employ Bayesian optimization to identify conditions for enzyme-catalyzed, site-selective esterifications of pyranoside derivatives. Our findings indicate that Bayesian algorithms facilitate these challenging optimizations, which involve simultaneous variation of enzyme identity, solvent, acyl donor loading, time and temperature, across a range of substrates with various configurations, protective groups and anomeric substituents. Using the experimental optimization data obtained for eighteen pyranoside substrates, we evaluate models for yield prediction and explore approaches for ‘warm-starting’ optimizations of previously unseen substrates. Products of the method are applied in photocatalytic hydrogen atom transfer reactions to generate ketodeoxy sugars.

## Abstract 2

Bayesian algorithms facilitate challenging optimizations of site-selective, enzyme-catalyzed monoacetylations of pyranoside derivatives. The data were used to evaluate transfer learning models for warm-starting new optimizations.

## Graphical abstract

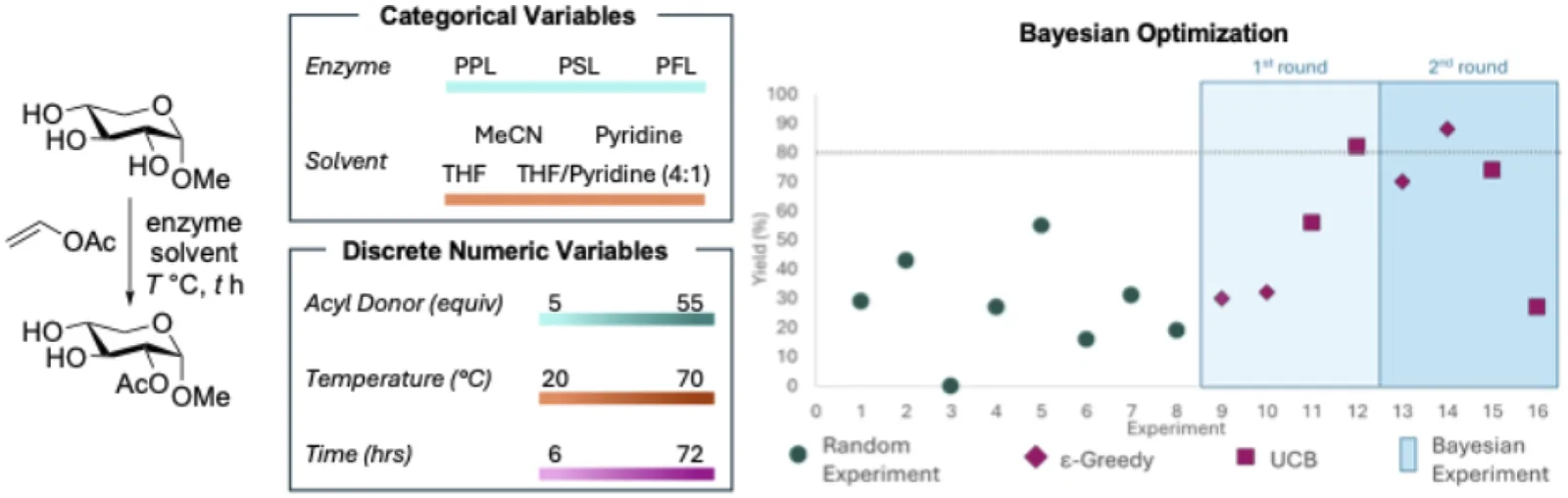

## Introduction

Site-selective functionalization of hydroxy (OH) groups is an enduring problem in carbohydrate chemistry.^1^ Enzymes offer unique opportunities in this context, particularly for selective esterification and ester hydrolysis.^2,3^ Applications of lipases and esterases to sugar-derived substrates were pioneered by the Wong and Klibanov groups in the late 1980s,^4,5^ and continue to attract interest.^6,7^ Sugars having a free primary OH group generally undergo enzyme-catalyzed esterification at this position. Differentiation between two or more secondary OH groups has also been achieved *via* enzyme catalysis,^6,8^ including examples of reaction outcomes that would be challenging to obtain by other means.

While exploring enzyme-catalyzed acylations to prepare substrates for use in photocatalysis,^9^ we encountered challenges with optimization. Reaction outcomes show a complex dependence on the configuration and substitution pattern of the sugar substrate, as well as the choice of enzyme and solvent;^10–12^ these issues are in addition to typical considerations for achieving efficient monofunctionalizations (dependence on reaction temperature, time and loading of the acylating agent). Reported reaction conditions differ significantly between substrates, and trends that might enable the rational improvement of yields were not apparent to us. Bayesian optimization,^13^ an approach for optimizing ‘black box’ objectives when the number of observations is constrained, appeared to be well suited to this problem. Previous work has shown that Bayesian methods are useful for optimizing protocols in chemical synthesis.^14–16^ Applications in carbohydrate chemistry are beginning to appear, with site-selective benzoylations and stereoselective glycosylations reported by the Pedersen group.^17,18^ Bayesian algorithms have also been implemented in various ways in biocatalysis,^19,20^ including for optimization of enzyme-catalyzed reactions.^21–27^ More broadly, applications of automation, flow chemistry, data science and machine learning to problems in carbohydrate chemistry are an important current direction for the field.^28–30^

In this study, we explore the utility of Bayesian optimization to identify synthetically viable conditions for enzyme-catalyzed, site-selective acetylation of secondary OH groups in pyranoside derivatives. While compatibility with automation and self-driving laboratories is an enabling feature of Bayesian algorithms,^31,32^ our focus here is on optimization schemes capable of delivering useful outcomes with a relatively small number of experiments, suitable for practitioners lacking access to specialized equipment. Indeed, we find that Bayesian optimization facilitates efficient exploration of the broad parameter space for enzyme-catalyzed monoacetylation of representative pyranoside substrates, with simultaneous variation of enzyme, solvent, temperature, time, and loading of the acetylating agent. The resulting dataset enables ‘warm-starting’ of new substrates by transfer learning, a machine learning approach in which a model developed for one objective is used as the starting point for a separate but related objective.^17,33–35^ We further demonstrate applications of the site-selective enzymatic acetylation in a two-step synthesis of ketodeoxysugars from unprotected pyranosides.

## Results and discussion

### Optimization parameters, substrates and protocols

The parameter space for Bayesian optimization was defined based on previously reported conditions for enzyme-catalyzed esterifications of secondary OH groups in carbohydrate derivatives,^2^ focusing on acetylation with vinyl acetate.^36^ The variables were enzyme identity, solvent, temperature, time and acyl donor loading (Fig. 1), with enzyme loading and substrate concentration held constant at 1 mol% and 0.2 M, respectively. The acyl donor loading was varied from 5 to 55 equivalents, the reaction temperature from 20 °C to 70 °C, and the reaction time from 6 h to 72 h. While reported reaction times of five days or longer are quite typical for such transformations, we did not evaluate such extended durations. Three lipase enzymes – porcine pancreatic lipase (PPL), amano lipase PS from *Burkholderia cepacia* (PSL) and amano lipase from *Pseudomonas fluorescens* (PFL) – were selected based on their prevalence in protocols for acylations of secondary OH groups in sugars.^2^ Novozym 435, lipase B from *Candida antarctica* immobilized on crosslinked poly(methyl methacrylate),^37^ was not included in the screen. We elected to avoid comparing immobilized and non-immobilized enzymes, and aimed to evaluate areas of reaction space distinct from those explored in recent studies focused on Novozym 435.^6,7^ The four reaction media investigated were acetonitrile, tetrahydrofuran (THF), pyridine and a THF/pyridine mixture (4 : 1 by volume), again selected from a survey of previously developed protocols.^2^ Neat vinyl acetate was not included as an option so that solvent and loading of acylating agent could be treated as independent variables. The number of enzymes and solvents evaluated was chosen based on considerations of experimental throughput; with a higher-throughput platform, additional candidates could be included by modifying the Python code provided (see the SI).

**Fig. 1:**
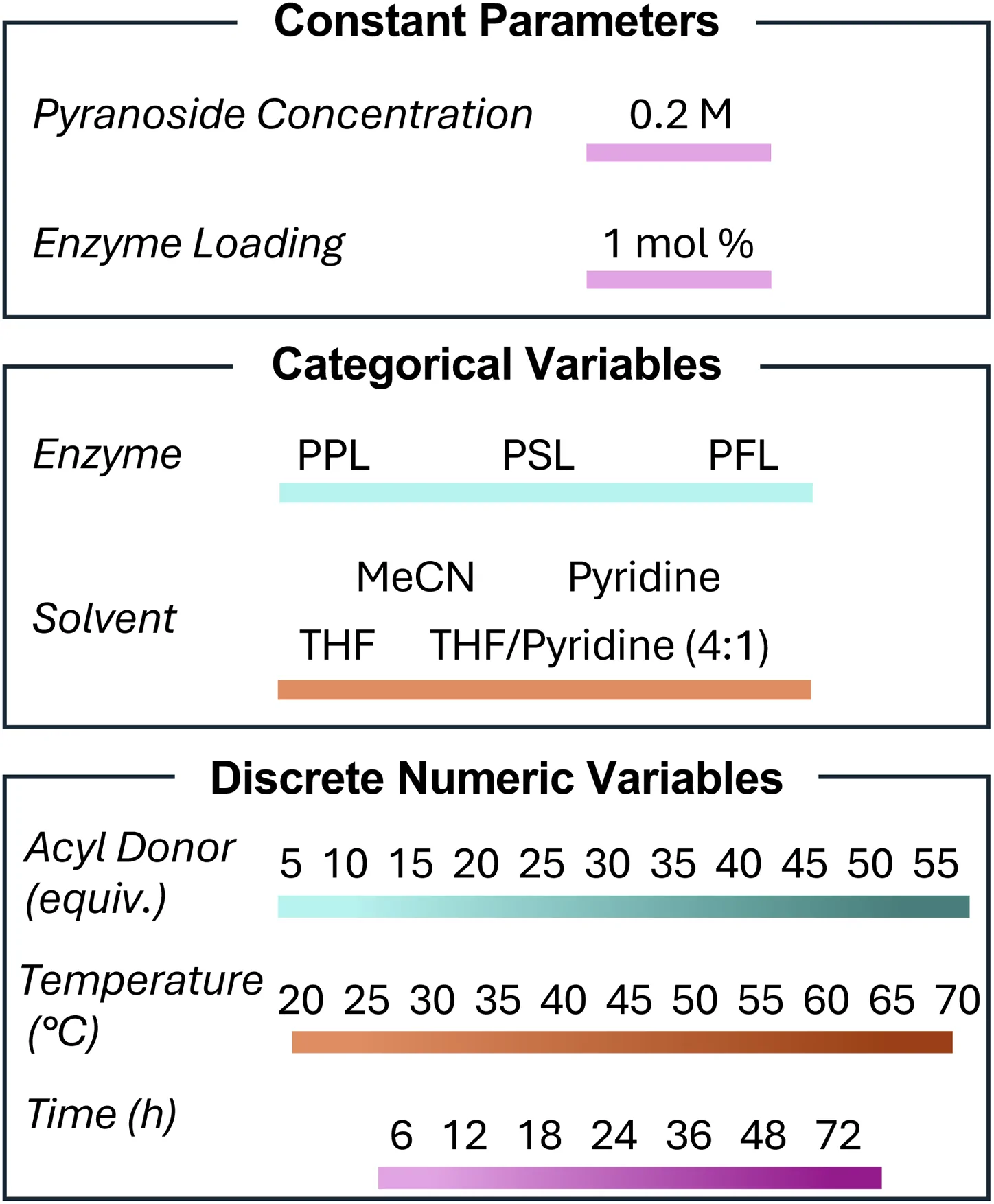
Summary of the experimental parameters explored *via* Bayesian optimization. PPL, PSL and PFL denote porcine pancreatic lipase, amano lipase PS from *Burkholderia cepacia* and amano lipase from *Pseudonomas fluorescens*, respectively.

Fig. 2 depicts the pyranoside substrates explored in this study, encompassing derivatives of the d-sugars α-Glc, β-Glc, α-Gal, β-Gal and α-Man, along with 6-deoxysugars (β-l-Fuc, α-l-Rha) and pentoses (α-d-Xyl, β-l-Ara, β-d-Ara). Both *O*- and *S*-glycosides were tested, along with various protective groups and substitution patterns (*tert*-butyldimethylsilyl (TBS) ethers, benzylidene acetals and a 1,6-anhydrosugar). While the focus was on site-selective acetylation of secondary OH groups, four representative pyranosides having a free primary 6-OH group were included.

**Fig. 2:**
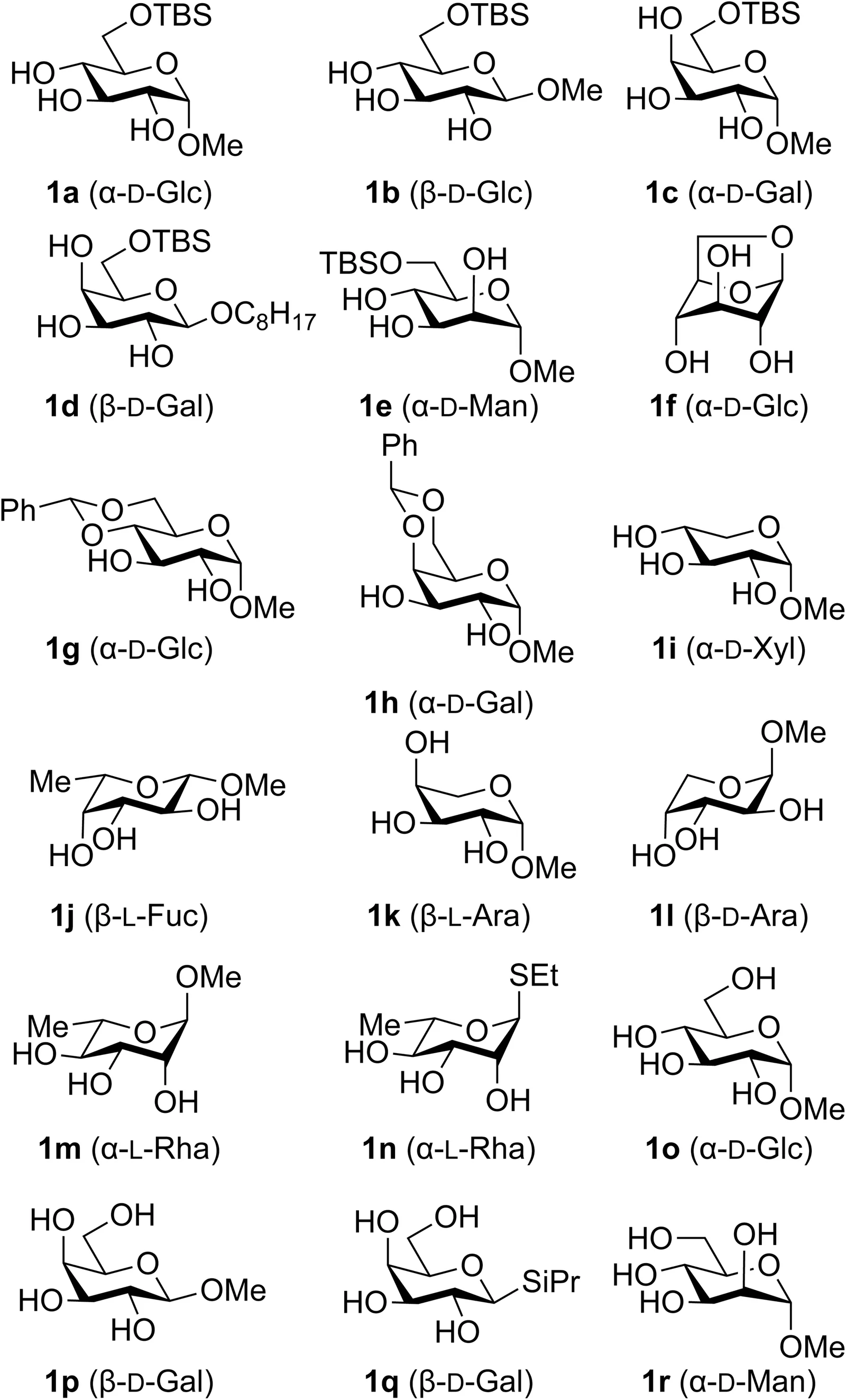
Substrates explored for Bayesian optimization of lipase-catalyzed, site-selective acetylation.

Bayesian optimization was conducted using the EDBO (Experimental Design by Bayesian Optimization) software developed by Doyle, Adams, Janey and co-workers.^14^ One-hot encoding was used for the non-numeric parameters (enzyme and solvent), while the numeric parameters were encoded to be discrete rather than continuous, for experimental tractability. Details and Python code are provided in the SI. In total, 10 163 combinations are possible from the defined parameter space. For each pyranoside substrate, eight initial sets of conditions were selected at random, allowing simultaneous variation of all five parameters. The experiments were conducted, and yields of the major acetylated product were determined by proton nuclear magnetic resonance (^1^H NMR) spectroscopic analysis of unpurified reaction mixtures. In a standard Bayesian optimization workflow, a surrogate model is updated after each round of experiments, and an acquisition function is used to select the next experiment. This selection can be performed sequentially, with one experiment chosen at a time, or in batch mode, with several experiments proposed for parallel evaluation. Batch selection is especially useful in experimental chemistry because multiple reactions can often be conducted at once. However, it requires the authors to define how the batch is constructed, including whether the selected experiments are intended to exploit the most promising predicted conditions, explore uncertain regions of chemical space, or balance both objectives. Based on the results of the initial eight experiments, two acquisition functions – *ε*-greedy (*ε* = 0.1), which prioritizes conditions predicted to give higher yield while retaining a small exploratory component, and upper confidence bound (UCB), which balances exploitation of high predicted yields with exploration of uncertain regions – were employed simultaneously, each generating two new sets of conditions, for a total of four new experiments. We anticipated that the combination of the two functions would be useful given the small number of experiments conducted, to ensure that more of the experimental space could also be surveyed whilst also improving the yield.^38,39^ Adaptive approaches, in which the balance between exploitation and exploration is dynamically controlled during the optimization, have also been developed to address this issue.^40^ The optimization was stopped either after two iterations of Bayesian optimization, corresponding to a maximum of eight additional experiments, or upon reaching a yield of 80%. Because a single product was observed in most cases, the optimization was based solely on yield, rather than a combination of yield and selectivity. The exceptions are 1f, 1o and 1p, which furnished mixtures of mono- and bis-acetylated products; in these cases, the algorithm was applied to optimize for the formation of each of the two possible products (see below). Representative plots illustrating the progress of the optimizations are provided in Fig. 3. For the majority of substrates, the optimal conditions were obtained by applying the exploitative *ε*-greedy acquisition function. However, for four pyranosides (1i, 1n, 1p and 1r: see the SI), the optimization path involved at least one set of conditions generated by the UCB acquisition function. These examples support the use of a mixed-acquisition batch strategy in data-limited optimizations, where uncertainty-guided sampling can help to avoid premature convergence on locally promising conditions.

**Fig. 3:**
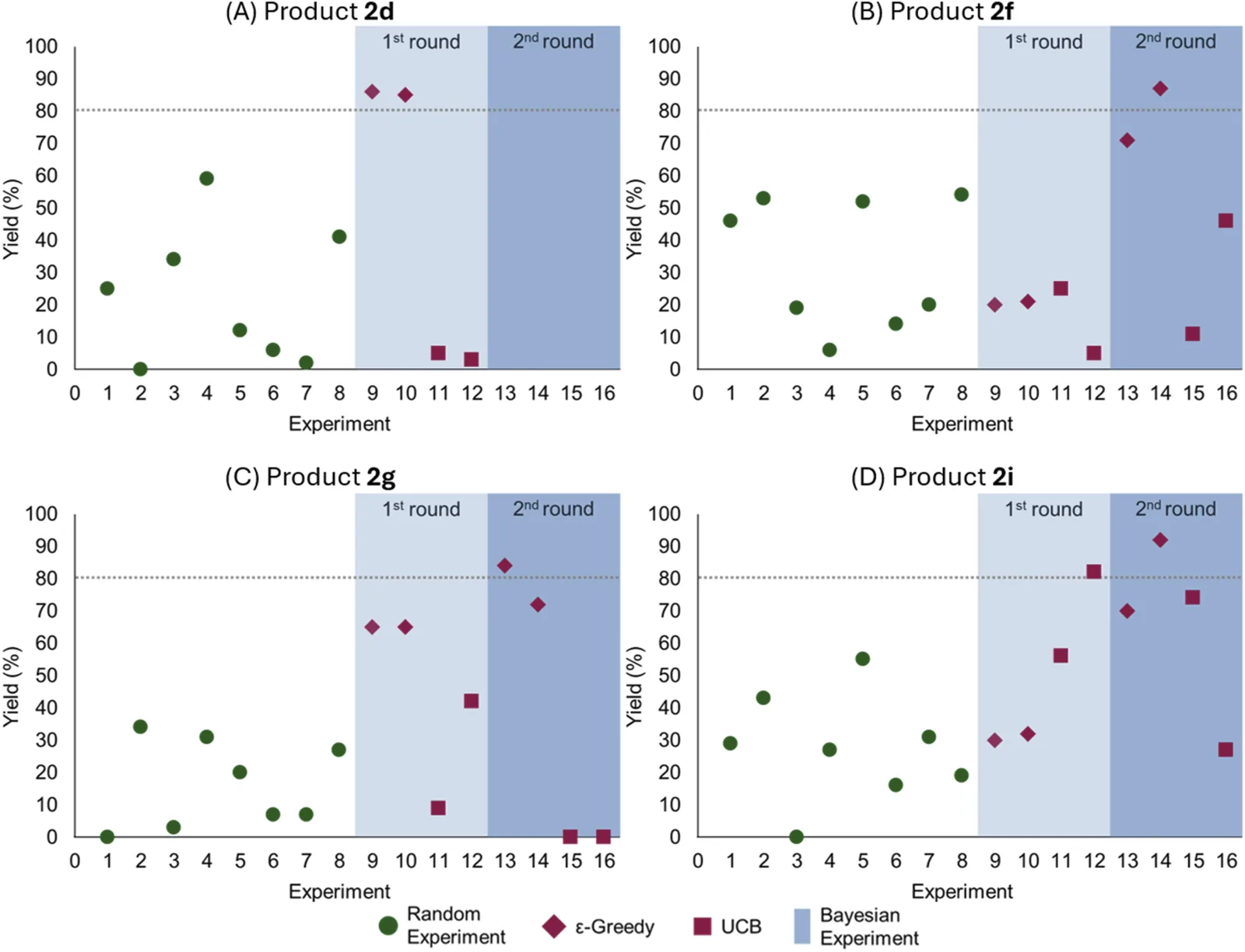
Results of Bayesian optimization experiments for the syntheses of 2d (A), 2f (B), 2g (C) and 2i (D). Yields were determined by ^1^H NMR spectroscopic analysis of unpurified reaction mixtures using 1,3,5-trimethoxybenzene as a quantitative internal standard.

### Optimized conditions and analysis

Table 1 depicts the products obtained, along with yields and optimized conditions for each. The lipase from *Pseudomonas fluorescens* (PFL) shows broad utility for site-selective acetylations of secondary OH groups, emerging as optimal in ten of the twelve such cases examined (products 2a–2n). The magnitude of this effect was somewhat unexpected to us based on compilations of literature protocols^2^ and previously reported head-to-head comparisons of enzyme activities.^41^ Nonetheless, both PPL and PSL were each selected once in optimizations for acetylations of secondary OH groups. Most often, THF/pyridine (6 examples) and THF (5 examples) proved to be the solvents of choice for such acetylation reactions. The optimal conditions show considerable variation; there is a preference for higher temperatures, reaction times and vinyl acetate loadings, but with exceptions (*e.g*., products 2i, 2j, 2l). A different set of trends is evident for substrates having free primary OH groups, where porcine pancreatic lipase was generally the optimal enzyme and pyridine the preferred solvent. Although PSL is listed as the optimal enzyme for the preparation of 6-*O*-acetylated thiogalactopyranoside 2q, six of the eight randomly selected experiments for this substrate gave yields exceeding 80%, including the 92% yield depicted in the table, and optimization with the Bayesian algorithm was not conducted.

**Table 1:** Optimized conditions and yields for the acylated pyranoside products^a^

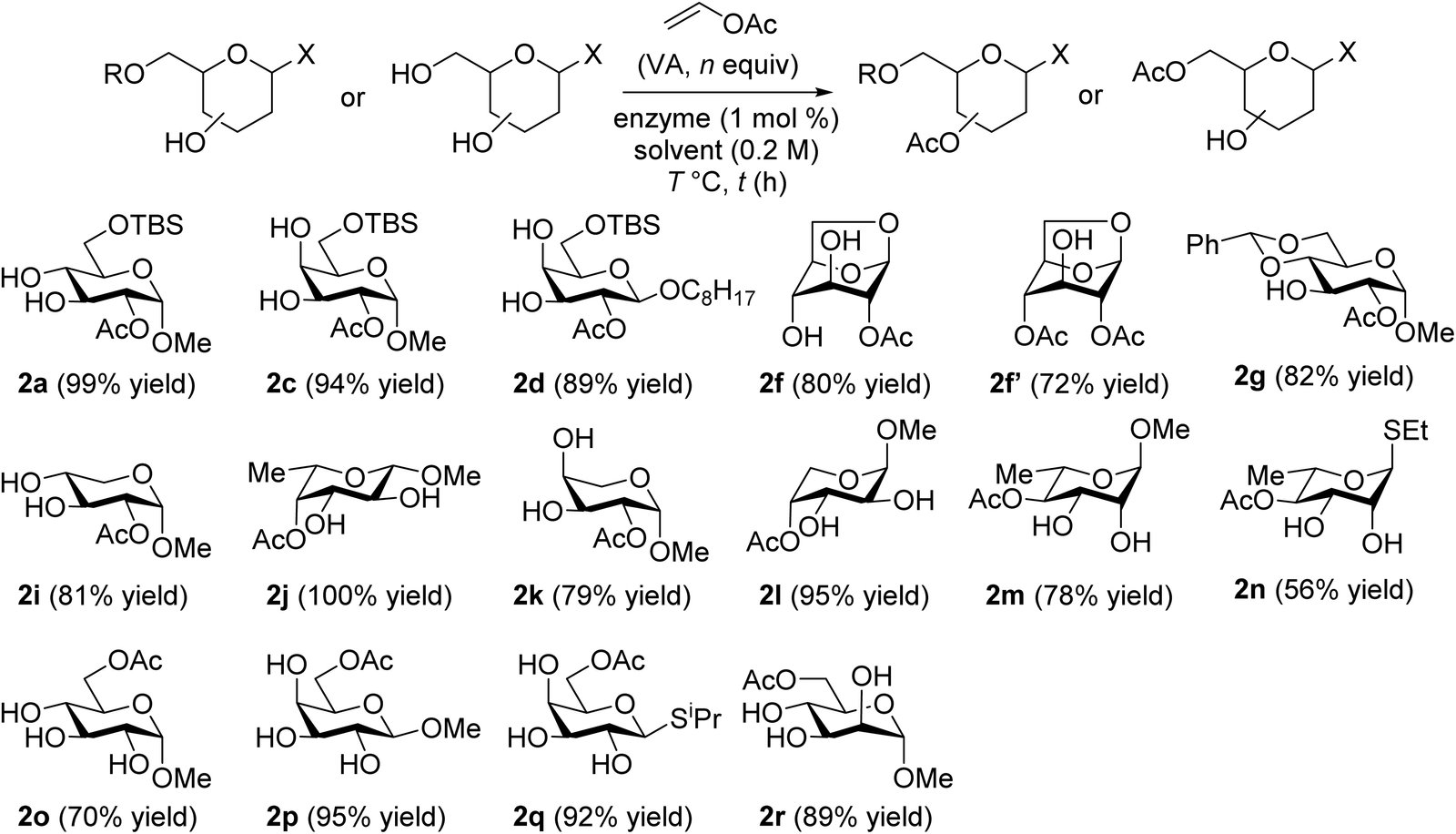					
Product	Enzyme	Solvent	Equiv. VA	*t* (h)	*T* (°C)
2a	PFL	THF	55	72	65
2c	PFL	THF/Py	55	72	35
2d	PFL	MeCN	55	72	35
2f	PPL	THF/Py	25	72	35
2f’	PFL	THF	5	36	65
2g	PFL	THF	55	72	70
2i	PFL	THF/Py	5	72	25
2j	PFL	THF/Py	5	48	40
2k	PFL	MeCN	25	36	70
2l	PFL	THF/Py	5	18	65
2m	PSL	THF/Py	50	72	70
2n	PFL	THF	10	72	70
2o	PPL	Py	45	12	70
2p	PPL	Py	55	72	20
2q	PSL	Py	55	36	40
2r	PPL	THF	55	6	70

a Yields from reactions conducted on 0.2 mmol scale, after purification by silica gel chromatography. PPL, PSL and PFL denote porcine pancreatic lipase, amano lipase PS from *Burkholderia cepacia* and Amano lipase from *Pseudonomas fluorescens*, respectively. THF/Py denotes a 4 : 1 v/v mixture of THF and pyridine.

The overall trends are illustrated in Fig. 4, which depicts the dependence of yield on enzyme and solvent across all experiments, grouping the substrates by presence or absence of a free primary OH group. It is clear from the plot that certain enzyme/substrate combinations are generally unproductive, and that these combinations differ across broadly defined substrate classes (*e.g.*, presence *versus* absence of a free primary OH group). This observation motivated our efforts to develop a transfer learning model for ‘warm-starting’ new optimizations (see below).

**Fig. 4:**
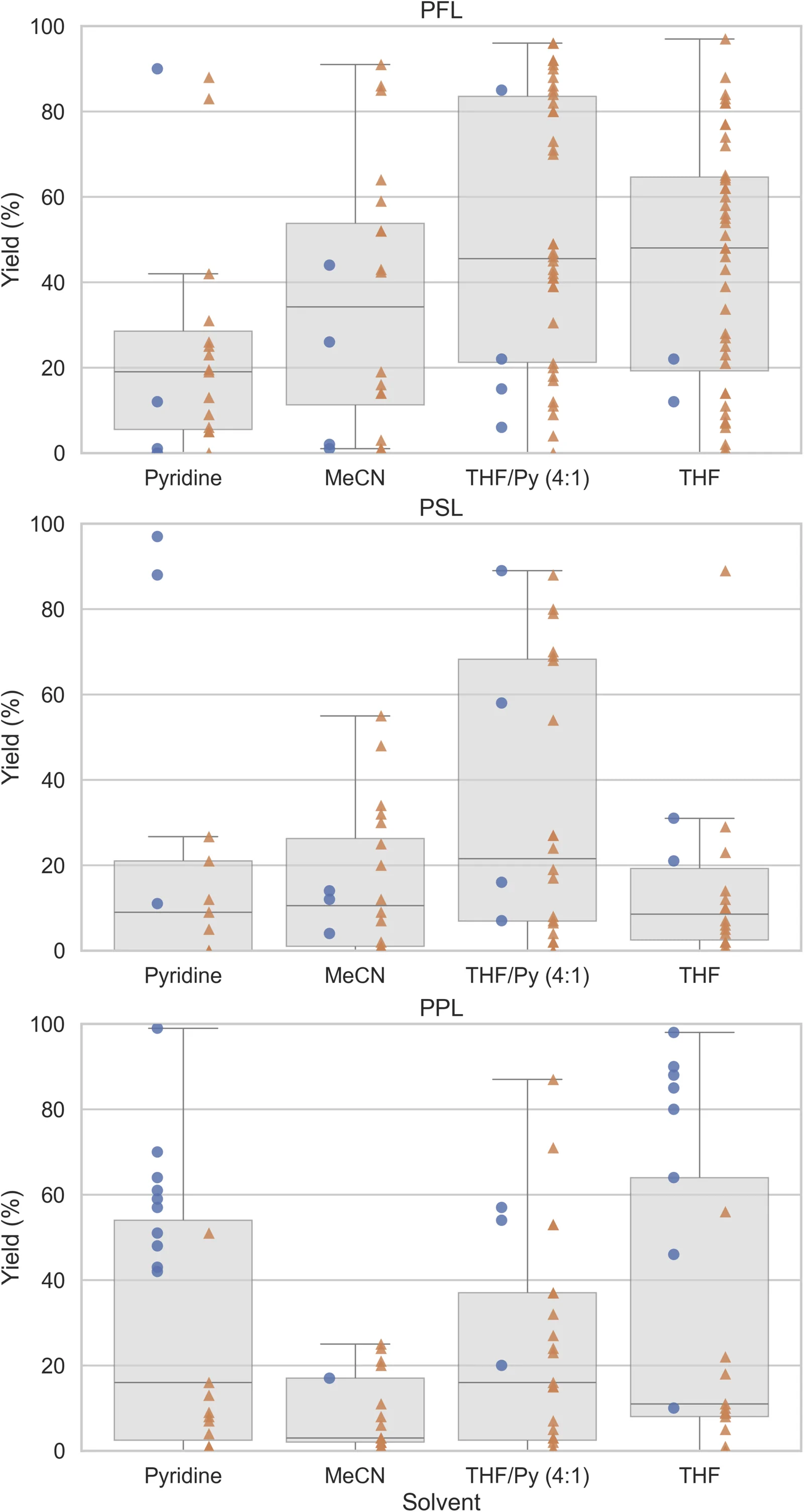
Variation of yield (determined by ^1^H NMR spectroscopic analysis) across all experiments, divided by enzyme, solvent and substrate type. Circle markers (blue) denote substrates with a free primary OH group, while triangle markers (orange) denote substrates without a free primary OH group.

Limitations are depicted in Fig. 5. TBS-protected β-glucopyranoside 1b was acylated in high yield but with poor selectivity, forming the 2- and 3-*O*-acetylated products as an inseparable mixture. The α-d-mannopyranoside-derived triol 1e showed low reactivity, with a maximum 14% yield of the 2-*O*-acetate obtained after 12 experiments. With the exception of 4,6-*O*-isopropylidene-protected congeners, α-d-mannopyranosides are known to be poorly reactive towards acetylation of secondary OH groups with the lipases employed here;^2,11^ acetylation at the 3-position has been achieved with Novozym 435.^6^ Benzylidene-protected α-galactopyranoside 1h was still less reactive, also consistent with literature findings.^42,43^ The 2,6-bis-*O*-acetates 2o′ and 2p′ were obtained as minor products while optimizing the syntheses of 2o and 2p. After two iterations of optimization, the bis-acetylation yields could not be improved beyond 37% and 49%, respectively. The problematic cases appear to reflect the intrinsic reactivity and selectivity profiles of the lipases employed; expanding the screen to include more enzymes or different acylating agents could be useful in this regard.

**Fig. 5:**
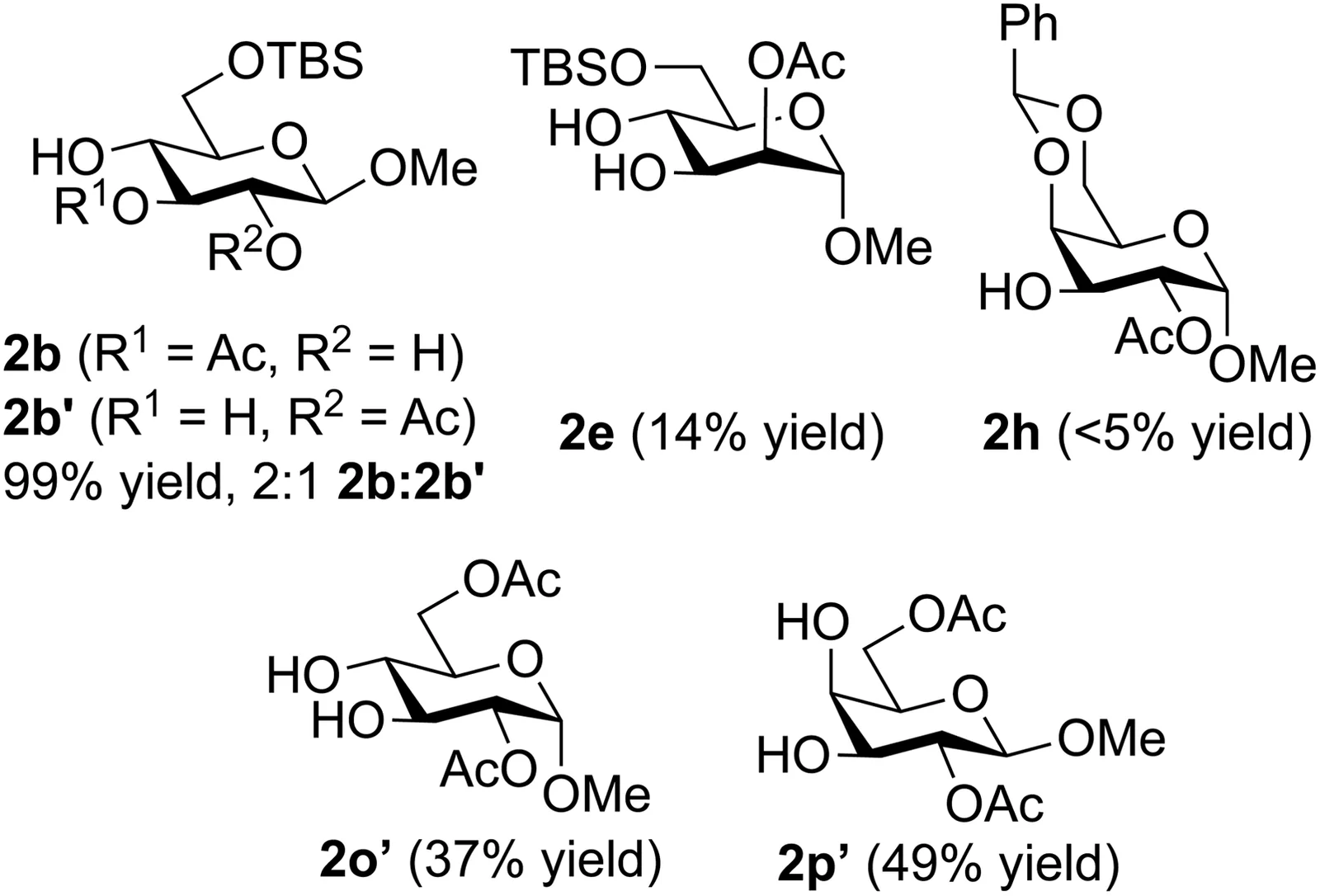
Limitations encountered when applying Bayesian optimization to pyranoside acetylation using the set of parameters summarized in Fig. 1. Yields were determined by ^1^H NMR spectroscopic analysis of unpurified reaction mixtures using 1,3,5-trimethoxybenzene as a quantitative internal standard.

As noted in the Introduction, the goal of this work was to define a pathway for identifying synthetically viable conditions, rather than to find truly optimum conditions. It is likely that improvements can be achieved by using the conditions summarized in Table 1 as a starting point for further optimization and intensification using established methods.^21^ Nonetheless, it is possible to compare our results to previously reported protocols (see the SI for details). While syntheses of 2a–2d, 2j, 2k and 2n*via* lipase-catalyzed acetylation with vinyl acetate have not been reported previously, the yields of 2f,^44^2f′,^44^2l,^41^ and 2p^12^ compare favorably with results from the literature. Higher yields have been obtained for products 2g,^42^2i^6^ and 2o;^12^ in the former case, the enzyme loading was 4.5 mol% (*versus* 1 mol% here), and in the latter two, Novozym 435 (not one of the enzymes evaluated) was used. The yield of product 2m^41^ exceeds the previously reported result using PFL, and a direct comparison for 2n is not available; however, similar or higher yields have been achieved for similar α-l-rhamnopyranoside derivatives using Novozym 435.^12,45^ Similarly, a directly comparable result for methyl α-mannopyranoside derivative 2r is not available, but Novozym 435 provides a high yield of the corresponding 6-*O*-acetylated benzyl glycoside.^12^

### Transfer learning

As illustrated by the graphs in Fig. 4, the randomly selected initial experiments have a high likelihood of sampling regions of reaction space that are broadly unproductive. To alleviate this issue, we aimed to use the data acquired for substrates 1a–1r to ‘warm-start’ new optimizations by transfer learning. Pedersen and co-workers found that the number of experiments needed to reach convergence for site-selective monobenzoylations of pyranosides (Bz_2_O, Et_3_N) could be decreased by using data from previously explored substrates to initialize new optimizations.^17^ More broadly, data-driven methods to select and prioritize reaction conditions are an area of active investigation.^46–50^

Several workflows for data-informed selection of reaction conditions begin by building a predictive model for yield. We investigated various such models, including random forest, Gaussian process regression, gradient boosting (GB) and CatBoost,^51^ a variant of GB. Considering the modest size of the dataset and the complexity of the underlying chemistry, we did not attempt to quantify subtle steric, electronic or stereochemical features of the substrates. Instead, we used simple descriptors – molecular weight, number of heavy atoms, oxygen atoms and free OH groups, configuration (gluco/galacto/manno) and presence/absence of a free primary OH group – along with the experimental conditions from the optimization (enzyme, solvent, loading of acylating agent, temperature and time). The four models performed similarly, and rather poorly: mean absolute errors (MAE) in yield for leave-one-substrate-out (LOSO) tests ranged from 20.1% to 20.6% (see the SI). The limited size of the dataset and relatively coarse descriptors used likely contribute to the low predictive power. Nonetheless, the models provide insight into broad trends. Relative feature importances from SHAP analysis of the CatBoost model, along with category-level SHAP contributions for the effect of enzyme, solvent and substrate configuration, are depicted in Fig. 6. The five most important features are enzyme, reaction time, solvent, substrate configuration and molecular weight. The utility of PFL and THF/pyridine or THF across most of the substrates, as discussed above in the context of Fig. 4, is evident from the category-level contributions. The effect of configuration is dominated by the low reactivity of manno-configured secondary alcohol substrates, particularly in the D series. We also evaluated an alternative approach, using ranking of conditions rather than yield prediction, building on work conducted by Zimmerman, Cernak and co-workers (see the SI).^52^

**Fig. 6:**
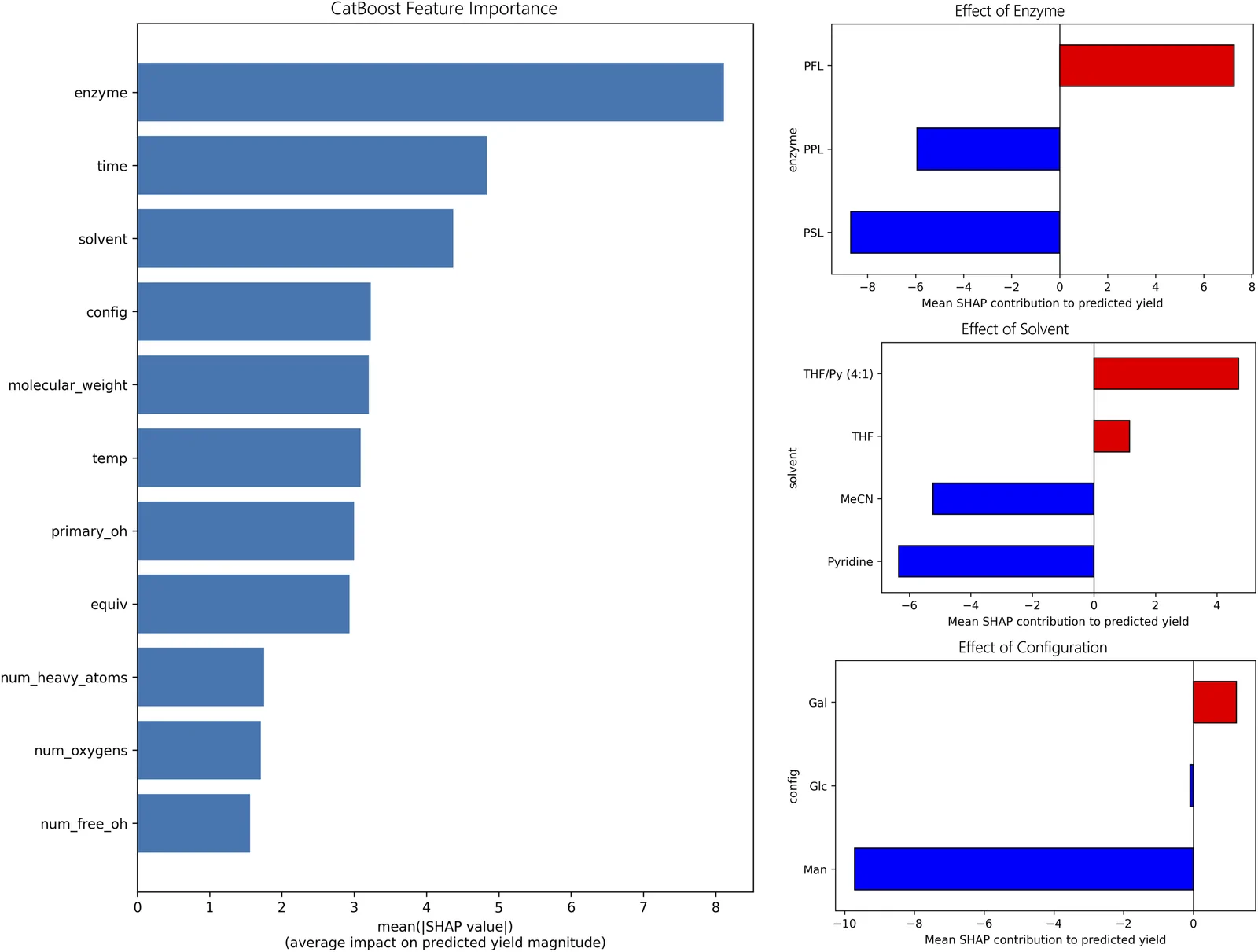
Feature importance from SHAP analysis of the CatBoost model, and category-level SHAP contributions for the effect of enzyme, solvent and substrate configuration.

Transfer learning was explored using substrates 1s–1v (Scheme 1). Conditions for the ‘warm-starting’ experiments were obtained by applying the above-mentioned yield prediction models to the descriptors for an unseen substrate, constraining the possible values of temperature, time and equivalents of vinyl acetate to a 4 × 4 × 3 grid (see the SI). For each of the top four-ranked enzyme–substrate combinations, two sets of conditions were selected, giving a total of eight initial experiments. These constraints were put in place to encourage exploration, as opposed to fine-tuning the predicted best enzyme/substrate combination, while initializing the optimization. The various models generally provided similar outputs (see the SI); we opted to conduct the experiments generated by an untuned GB model, which tended to favor more diverse enzyme/substrate combinations. The use of transfer learning significantly improved the success rates of the initial experiments *versus* random selection (see the SI). For compound 1s, an 84% yield was obtained in the initial round, and no further experiments were conducted. For 1t, the initial set of eight experiments gave a maximum yield of 79%, which was further increased to 82% by applying the Bayesian algorithm. Substrate 1q was the most challenging: a maximum yield of 31% was obtained after the first eight experiments, with no improvement obtained after a subsequent Bayesian optimization round. The presence of the aryl thioglycoside group, which is not represented among substrates 1a–1r, is likely responsible. However, it is noteworthy that each of the initial ‘warm-starting’ experiments gave appreciable amounts of product 2q (17% yield or higher), indicating that this approach was helpful for identifying potentially viable experimental regimes. Finally, α-configured mannopyranosyl thioglycoside 1v bearing a free primary OH group was investigated, giving an 84% yield after the initial 8 experiments.

**Scheme 1:**
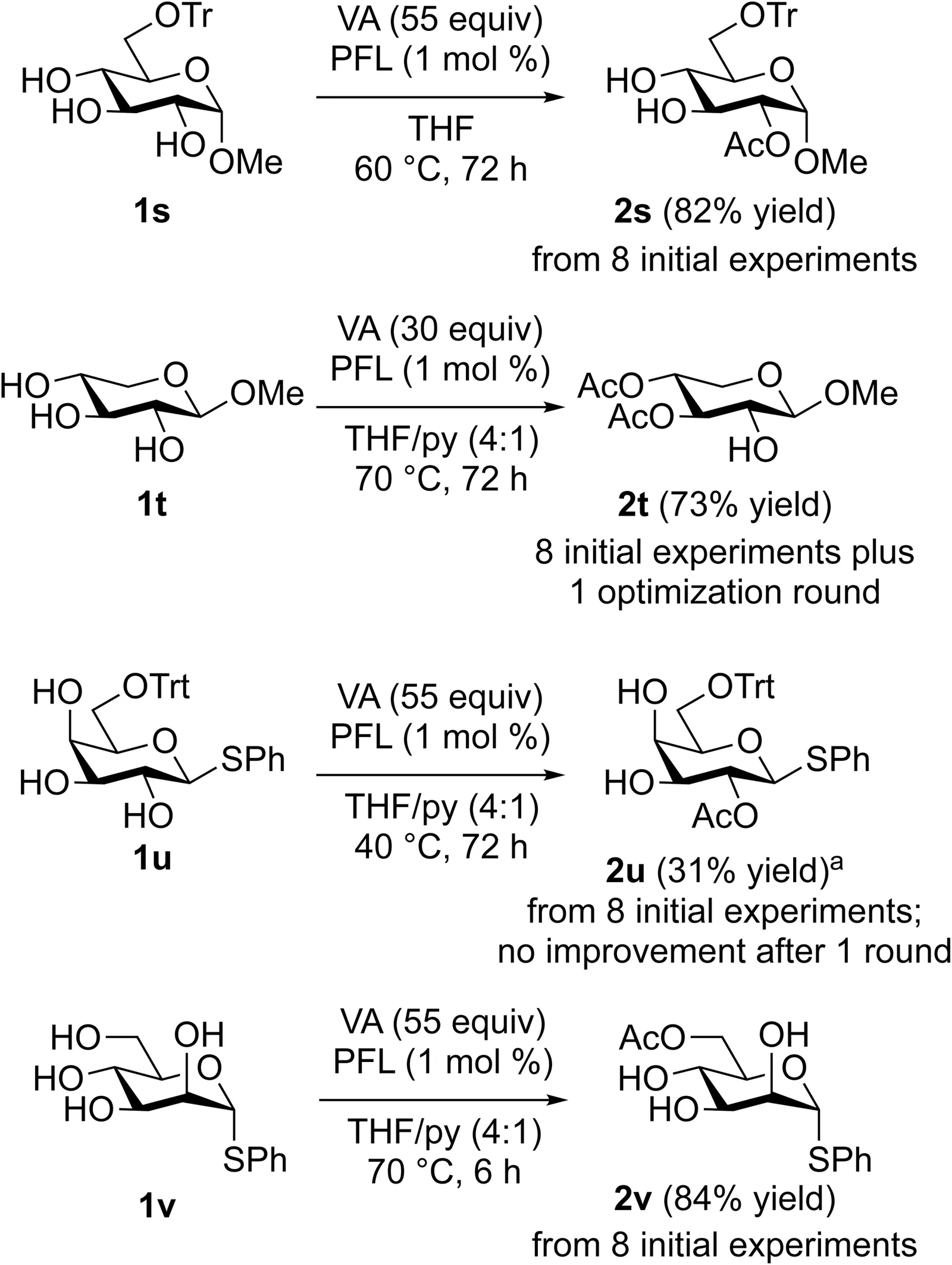
Results of ‘warm-starting’ experiments for substrates 1s–1v. ^*a*^ Denotes yield determined by ^1^H NMR spectroscopic analysis with 1,3,5-trimethoxybenzene as quantitative internal standard.

As an alternative to the transfer learning approach described above, we sought to investigate whether conditions that are optimal for most substrates could be used as a starting point for optimization. The best eight conditions, as judged by the number of experiments giving a yield of 80% or higher across the full dataset (Table 2 see the SI for details), were applied to the ‘unseen’ substrates 1s–1v.

**Table 2:** The best eight sets of reaction conditions, as judged by the number of instances of a yield of 80% or higher across the full dataset^a^

Experiment type	Enzyme	Solvent	Acyl equiv.	Time (hours)	Temp. (°C)	Count (≥80%)
Top conditions 1	PFL	THF/Py (4 : 1)	5	24	60	4
Top conditions 2	PFL	THF	55	72	70	3
Top conditions 2	PFL	THF/Py (4 : 1)	5	72	20	3
Top conditions 3	PFL	THF/Py (4 : 1)	55	72	40	2
Top conditions 3	PFL	THF/Py (4 : 1)	5	48	40	2
Top conditions 3	PSL	THF/Py (4 : 1)	55	72	70	2
Top conditions 3	PPL	THF	55	24	20	2
Top conditions 4	PPL	THF/Py (4 : 1)	25	72	40	1

a The values of acyl group loading, temperature and time were constrained to the same 4 × 4 × 3 grid used for the warm-starting experiments.

For consistency, the numerical variables (loading of vinyl acetate, time and temperature) were constrained to the same 4 × 4 × 3 grid used for the warm-starting experiments (see the SI). The highest yields obtained from this set of experiments exceeded those from the transfer learning-based warm-starting experiments for substrates 1s, 1t and 1v (Scheme 2), underscoring the utility of PFL in THF or THF/pyridine as a generally applicable set of conditions for selective acetylation of secondary OH groups in pyranosides. However, it is noteworthy that the warm-starting experiments provided higher average yields than the ‘best eight’ conditions for three of the four substrates examined (see the SI), despite being constrained to explore distinct enzyme/substrate combinations.

**Scheme 2:**
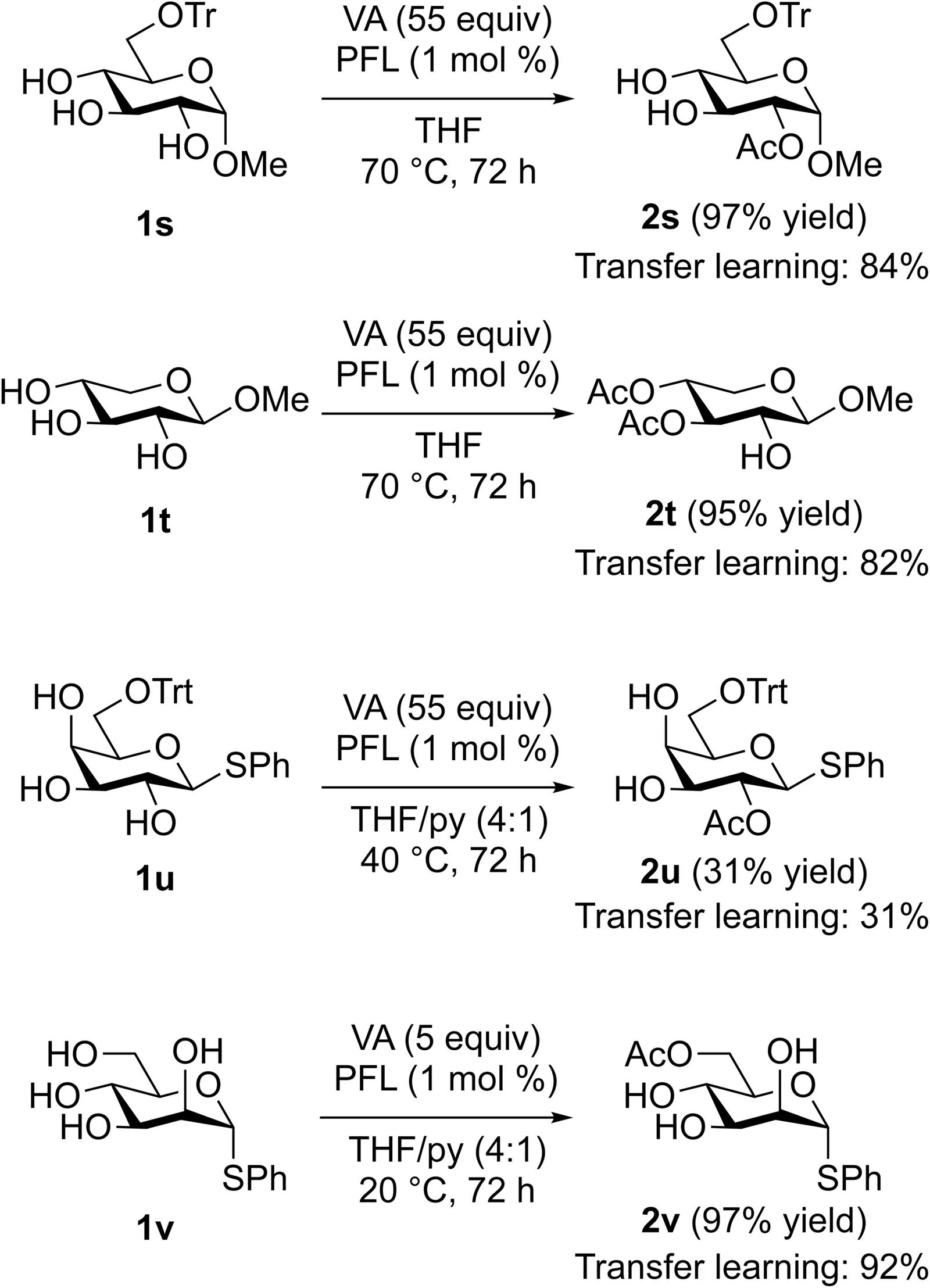
Results of ‘best eight’ experiments for substrates 1s–1v. Yields were determined by ^1^H NMR spectroscopic analysis with 1,3,5-trimethoxybenzene as quantitative internal standard.

Finally, the monoacetylated products of the method were applied as substrates for the synthesis of ketodeoxysugars *via* photocatalytic hydrogen atom transfer, using a protocol developed in our laboratory (Scheme 3).^9^ The synthesis of 3a from 2a in 80% yield compares favorably to the 71% that our group achieved previously using the corresponding 2-*O*-pivaloyl-bearing substrate. The yields obtained from β-fucose derivative 2e, and the pentose derivatives 2f and 2h, were more modest; however, it should be noted that 3f and 3g have not been synthesized to date, and that the only reported synthesis of 3j requires four steps (55% overall yield, d-enantiomer).^53^ Attempts at isolation of products 3i–3m resulted in loss of material; each product was determined to be volatile *in vacuo*, and related compounds are known to dimerize through hemiketal formation or aldol addition.^53,54^ Accordingly, the yields of 3i–3m reported in Scheme 3 were determined by ^1^H NMR spectroscopic analysis.

**Scheme 3:**
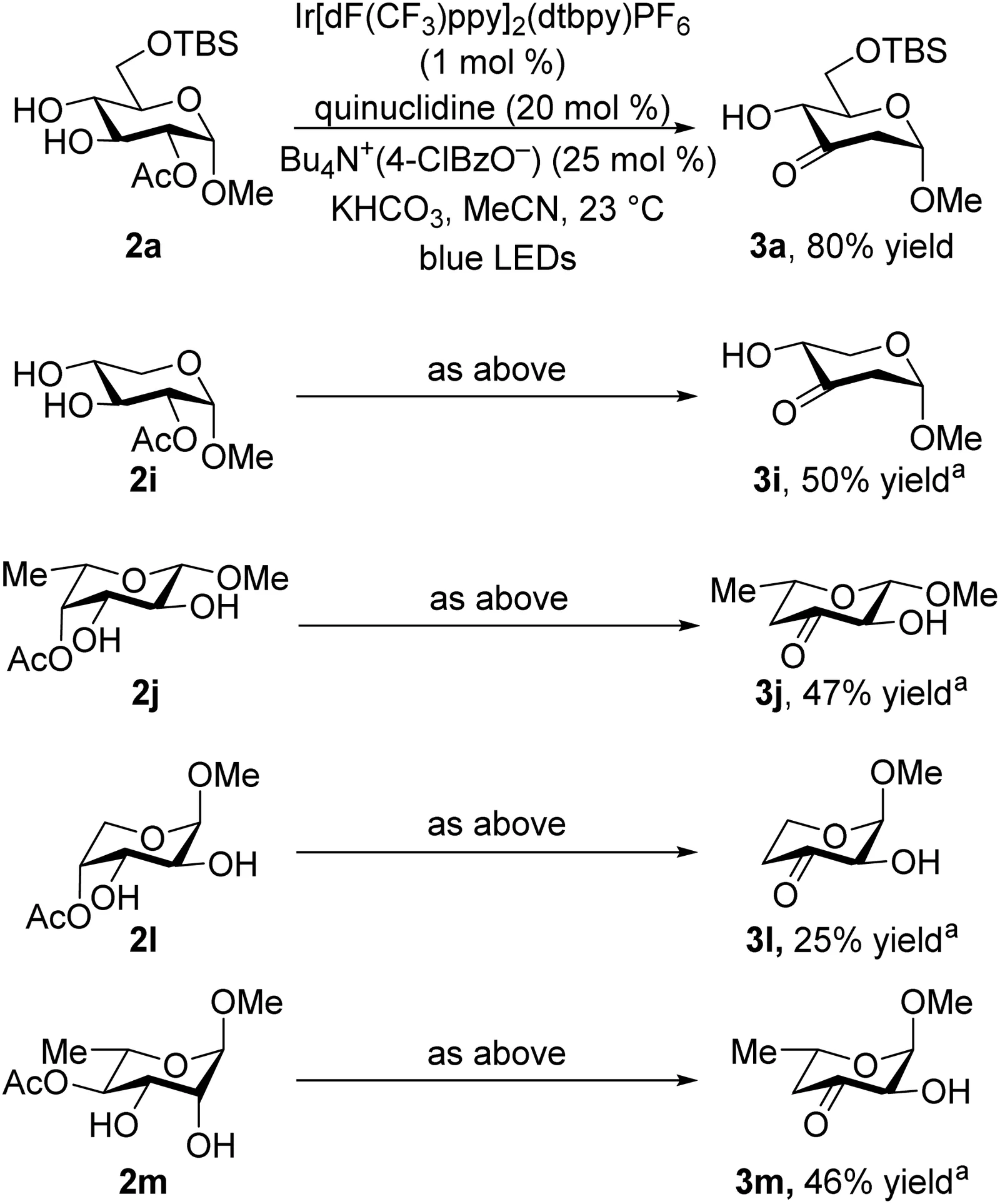
Synthesis of ketodeoxysugars from monoacetylated pyranosides. ^*a*^Denotes yield determined by ^1^H NMR spectroscopic analysis with 1,3,5-trimethoxybenzene as quantitative internal standard.

## Conclusions

In conclusion, Bayesian optimization has been used to facilitate the development of protocols for lipase-catalyzed monoacetylation of diverse pyranoside substrates. Such optimizations are challenging due to the interplay between categorical and numerical variables, along with the complexity of the underlying chemistry. The workflow has been applied to develop conditions for previously unexplored substrates, and to avoid the use of the extended reaction times that are often a hallmark of certain lipase-catalyzed esterifications. While substantial variations in the optimized conditions are evident across the range of pyranosides examined, the collected data also reveal general trends that can be exploited to facilitate the optimization of new substrates. Opportunities exist to further analyze and augment this dataset, and to extend the approach to other complex processes involving lipases,^55^ or more broadly to other chemoenzymatic reactions where simultaneous variation of enzyme, solvent and conditions is needed.

## Author contributions

The manuscript was written through contributions of all authors. All authors have given approval to the final version of the manuscript.

## Conflicts of interest

There are no conflicts to declare.

## Supplementary Material

SC-OLF-D6SC06368C-s001

## Data Availability

The data supporting this article have been included as part of the supplementary information (SI). Supplementary information: experimental details, analysis methods and characterization data, including copies of ^1^H, ^13^C{^1^H} and COSY NMR spectra (PDF). See DOI: https://doi.org/10.1039/d6sc06368c.
