# Supplementary material for: Bayesian optimization for enzyme-catalyzed, site-selective acetylation of carbohydrate OH groups
Source: Chem Sci. 2026 Nov 4. Online ahead of print. doi: 10.1039/d6sc06368c (PMC13732138; doi:10.1039/d6sc06368c)

Supplementary Information (SI) for Chemical Science.
This journal is © The Royal Society of Chemistry

Supplementary Information

Bayesian Optimization for Enzyme-Catalyzed, Site-Selective Acetylation of Carbohydrate OH Groups

Katrina H. Ruzicka,[a] Matthew T. Zambri,[a] Jolene P. Reid,[b] * and Mark S. Taylor[a] *

marks.taylor@utoronto.ca

[a]Department of Chemistry, University of Toronto, 80 St. George St., Toronto, ON M5S 3H6, Canada

[b]Department of Chemistry, University of British Columbia, 2036 Main Mall, Vancouver, BC V6T 1Z1, Canada

Table of Contents

General Information **5**
Materials 5
Instrumentation 5
General Procedure for Screening Reactions **5**
Preparation of Starting Materials **6**
Methyl 6-*O*-(*tert*-butyldimethylsilyl)-α-D-glucopyranoside (1a) 6
Methyl 6-*O*-(tert-butyldimethylsilyl)-β-D-glucopyranoside (1b) 6
Methyl 6-*O*-(tert-butyldimethylsilyl)-α-D-galactopyranoside (1c) 6
n-Octyl 6-*O*-(tert-butyldimethylsilyl)-α-D-galactopyranoside (1d) 7
Methyl 6-*O*-(tert-butyldimethylsilyl)-α-D-mannopyranoside (1e) 7
Methyl 4,6-benzylidene α-D-glucopyranoside (1g) 7
Methyl 4,6-benzylidene α-D-galactopyranoside (1h) 8
Ethyl thio-α-L-rhamnopyranoside (1n) 8
Methyl 6-O-trityl-α-D-glucopyranoside (1s) 8
Phenyl 6-O-trityl-β-D-1-thio-galactopyranoside (1s) 8
Characterization of Products **9**
General Procedure A 9
General Procedure B 9
Methyl 2-*O*-acetyl-6-*O*-(tert-butyldimethylsilyl)-α-D-glucopyranoside (2a) 10
Methyl 2-O-acetyl 6-*O*-(tert-butyldimethylsilyl)-α-D-galactopyranoside (2c) 10
n-Octyl 2-*O*-acetyl 6-*O*-(tert-butyldimethylsilyl)-α-D-galactopyranoside (2d) 10
4-*O*-acetyl 1,6-β-D-anhydroglucose (2f) 11
2,4-di-*O*-acetyl 1,6-β-D-anhydroglucose (2f') 11
Methyl 2-O-acetyl-4,6-benzylidene α-D-glucopyranoside (2g) 12
Methyl 2-*O*-acetyl-α-D-xylopyranoside (2i) 12
Methyl 4-*O*-acetyl-β-L-fucopyranoside (2j) 13
Methyl 2-*O*-acetyl-β-L-arabinopyranoside (2k) 13
Methyl 4-*O*-acetyl-β-D-arabinopyranoside (2l) 13
Methyl 4-*O*-acetyl-α-L-rhamnopyranoside (2m) 14
Ethyl 1-thio-4-*O*-acetyl-α-L-rhamnopyranoside (2n) 14
Methyl 6-*O*-acetyl-α-D-glucopyranoside (2o) 15
Methyl 6-*O*-acetyl-β-D-galactopyranoside (2p) 15
Isopropyl 6-*O*-acetyl-β-D-1-thio-galactopyranoside (2q) 15

Methyl 6-*O*-acetyl-α-D-mannopyranoside (2r) 16
Methyl 6-*O*-trityl-2-*O*-acetyl-α-D-glucopyranoside (2s) 16
Methyl 2,6-di-*O*-acetyl-β-D-xylopyranoside (2t) 17
Phenyl 6-*O*-acetyl-α-D-1-thio-mannopyranoside (2v) 17
Methyl 2-deoxy-6-*O*-(tert-butyldimethylsilyl)-***α***-D-erythro-hexopyranoside-3-ulose (3a) 18
Python Code for Parameter Generation 20
Bayesian Optimization Data Tables 23
2a – Methyl 2-*O*-acetyl-6-*O*-(tert-butyldimethylsilyl)-α-D-glucopyranoside 23
2b – Methyl 2-*O*-acetyl-6-*O*-(tert-butyldimethylsilyl)-β-D-galactopyranoside 24
2c – Methyl 2-*O*-acetyl-6-*O*-(tert-butyldimethylsilyl)-α-D-galactopyranoside 25
2d – n-Octyl 2-*O*-acetyl-6-*O*-(*tert*-butyldimethylsilyl)-α-D-galactopyranoside 26
2e – Methyl 2-*O*-acetyl-6-*O*-(tert-butyldimethylsilyl)-α-D-mannopyranoside 27
2f – 4-*O*-acetyl 1,6-β-D-anhydroglucose 28
2f' – 2,4-di-*O*-acetyl 1,6-β-D-anhydroglucose 29
2g – Methyl 2-*O*-acetyl-4,6-benzylidene α-D-glucopyranoside 30
2h – methyl 2-*O*-acetyl-4,6-benzylidene α-D-galactopyranoside 31
2i – Methyl 2-*O*-acetyl-α-D-xylopyranoside 32
2j – Methyl 4-*O*-acetyl-β-L-fucopyranoside 33
2k – Methyl 2-*O*-acetyl-β-L-arabinopyranoside 34
2l – Methyl 4-*O*-acetyl-β-D-arabinopyranoside 35
2m – Methyl 4-*O*-acetyl-α-L-rhamnopyranoside 36
2n – ethyl 1-thio-4-*O*-acetyl-α-L-rhamnopyranoside 37
2o – Methyl 6-*O*-acetyl-α-D-glucopyranoside 38
2o' – Methyl 6-*O*-acetyl-α-D-glucopyranoside 39
2p – Methyl 6-*O*-acetyl-β-D-galactopyranoside 40
2p' – Methyl 6-*O*-acetyl-β-D-galactopyranoside 41
2q – 6-*O*-acetyl-Isopropyl-β-D-Thiogalactopyranoside 42
2r – Methyl 6-*O*-acetyl-α-D-mannopyranoside 43
2w – Methyl 2-*O*-acetyl-5-*O*-(tert-butyldimethylsilyl)-α-D-ribofuranoside 44
Comparisons to Literature 45
Transfer Learning 47
Untuned Gradient Boosting Model 50
Substrate 1v 51
Tuned Gradient Boosting Model 51
Random Forest Model 53

Gaussian Process Regression Model 55
CatBoost Model 56
Warm-Starting Experimental Results 61
2s – Methyl 6-*O*-trityl-2-*O*-acetyl-α-D-glucopyranoside 61
2t – Methyl 3,4-di-*O*-acetyl-β-D-xylopyranoside 62
2u – Phenyl 1-thio-6-O-trityl-2-*O*-acetyl-β-D-galactopyranoside 63
2v – Phenyl 6-*O*-acetyl-α-D-1-thio-mannopyranoside 64
Comparison of transfer learning to most common reaction conditions 65
2s – Methyl 6-*O*-trityl-2-*O*-acetyl-α-D-glucopyranoside 66
2t – Methyl 3,4-di-*O*-acetyl-β-D-xylopyranoside 67
2u – Phenyl 1-thio-6-O-trityl-2-*O*-acetyl-β-D-galactopyranoside 68
2v – Phenyl 6-*O*-acetyl-α-D-1-thio-mannopyranoside 69
References 70
^{1}H, ^{13}C and 2D NMR Spectra 71
Methyl 2-*O*-acetyl-6-*O*-(tert-butyldimethylsilyl)-α-D-glucopyranoside (2a) 72
Methyl 2-*O*-acetyl-6-*O*-(tert-butyldimethylsilyl)-α-D-galactopyranoside (2c) 75
n-Octyl 2-*O*-acetyl-6-*O*-(*tert*-butyldimethylsilyl)-α-D-galactopyranoside (2d) 78
4-*O*-acetyl 1,6-β-D-anhydroglucose (2f) 81
2,4-di-*O*-acetyl 1,6-β-D-anhydroglucose (2f') 84
Methyl 2-*O*-acetyl-4,6-benzylidene α-D-glucopyranoside (2g) 87
Methyl 2-*O*-acetyl-α-D-xylopyranoside (2i) 90
Methyl 4-*O*-acetyl-β-D-fucopyranoside (2j) 93
Methyl 2-*O*-acetyl-β-L-Arabinopyranoside (2k) 96
Methyl 4-*O*-acetyl-β-D-Arabinopyranoside (2l) 99
Methyl 4-*O*-acetyl-α-L-rhamnopyranoside (2m) 102
Ethyl 1-thio-4-*O*-acetyl-α-L-rhamnopyranoside (2n) 105
Methyl 6-*O*-acetyl-α-D-glucopyranoside (2o) 108
Methyl 6-*O*-acetyl-β-D-galactopyranoside (2p) 111
Isopropyl 6-*O*-acetyl-β-D-1-thiogalactopyranoside (2q) 114
Methyl 6-*O*-acetyl-α-D-mannopyranoside (2r) 117
Methyl 2-*O*-acetyl-6-*O*-trityl-α-D-glucopyranoside (2s) 120
Methyl 4-*O*-acetyl β-D-xylopyranoside (2t) 123
Phenyl 6-O-acetyl-α-D-1-thio-mannopyranoside (2v) 126
Methyl 2-deoxy-6-O-(tert-butyldimethylsilyl)-***α***-D-erythro-hexopyranoside-3-ulose (3a) 129
Methyl 4,6-dideoxy-α-D-erythro-hexopyranoside-3-ulose (3m) 132

Methyl 4,6-dideoxy-β-D-erythro-hexopyranoside-3-ulose (3j) 134
Methyl 2,6-dideoxy-α-D- erythro-hexopyranoside-3-ulose (3i) 136
Methyl 5,6-dideoxy-α-D-erythro-hexopyranoside-3-ulose (3l) 138

General Information

Materials

Stainless steel needles and syringes were used for the transfer of air and moisture sensitive solvents. Analytical thin layer chromatography was performed using aluminum-backed silica gel 60 F254 plates (EMD), with visualization of compounds using $KMnO_4$ stain. Flash column chromatography was performed with neutral silica gel (60 Å, 230–400 mesh, Silicycle). All solvents were dried over activated 4 Å molecular sieves. 4 Å molecular sieves were stored at 140 °C for at least 24 hours before use. Stainless steel needles and syringes were used to transfer air and moisture sensitive liquids. Lipase enzymes were obtained from Sigma Aldrich.

Instrumentation

Proton NMR and Carbon NMR were obtained using a Bruker Avance III 400 MHz spectrometer. ^{1}H and ^{13}C NMR are reported in parts per million (ppm) and referenced to solvent peak(s). Spectral features are tabulated in the following order: chemical shift (δ, ppm); multiplicity (s-singlet, d-doublet, t-triplet, q-quartet, p-pentet, m-complex multiplet); coupling constant(s) (J, Hz); number of protons. Infrared (IR) spectra were obtained on a Perkin-Elmer Spectrum 100 instrument equipped with a singlebounce diamond/ZnSe ATR accessory as neat samples, or as thin film from CH_2Cl_2 as indicated. Spectral features are tabulated as follows: wavenumber (cm–1); intensity (s-strong, m-medium, w-weak).

General Procedure for Screening Reactions

To a 2 dram vial was added glycoside (1 equiv.), enzyme (1 mol%) and a small magnetic stir bar. A rubber septum was then used to seal the vial. The vial was then evacuated and backfilled with argon on a Schlenk line three times. Dry solvent (0.2 M) and vinyl acetate was then added under a balloon of argon. The rubber septum was removed and quickly replaced with the vial cap. The vial was then sealed and covered with Teflon tape and parafilm. The vial was placed on a hot plate in an oil bath and stirred at 500 rpm for the specified time at the specified temperature. After the required amount of time, the crude reaction was filtered through a plug of celite with a 50:50 v/v mixture of DCM/MeOH to remove the solid enzyme, then concentrated under reduced pressure. Internal standard (1,3,5-trimethoxybenzene) was added, and the material was analyzed by ^{1}H NMR spectroscopy.

General Procedure A

To a 2 dram vial was added glycoside (1 equiv.), enzyme (1 mol%) and a small magnetic stir bar. A rubber septum was then used to seal the vial. The vial was then evacuated and backfilled with argon on a Schlenk line three times. Dry solvent (0.2 M) and vinyl acetate was then added under a balloon of argon. The rubber septum was removed and quickly replaced with the vial cap. The vial was then sealed and covered with

Teflon tape and parafilm. The vial was placed on a hot plate in an oil bath and stirred at 500 rpm for the specified time at the specified temperature. After the required amount of time, the crude reaction was concentrated under reduced pressure, then filtered through a plug of celite with a 50:50 v/v mixture of DCM/MeOH to remove the solid enzyme, then concentrated under reduced pressure. The crude material was then subjected to flash column chromatography on silica.

Preparation of Starting Materials

Methyl 6-*O*-(*tert*-butyldimethylsilyl)-α-ᴅ-glucopyranoside (1a)

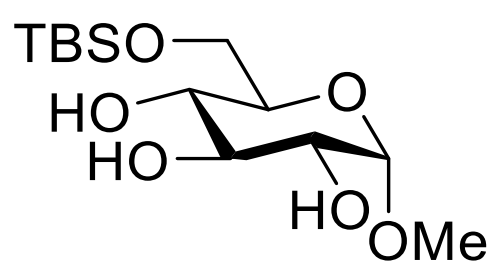

Synthesized according to literature procedure from methyl α-ᴅ-glucopyranoside and *tert*-butyldimethyl silyl chloride.[1]

^{1}H NMR (400 MHz, $CDCl_3$): δ 4.74 (d, J = 3.8 Hz, 1H), 3.88–3.80 (m, 2H), 3.74 (app t, J = 9.2 Hz, 1H), 3.59 (dt, J = 9.7, 4.8 Hz, 2H), 3.50 (d, J = 9.3 Hz, 3H), 3.41 (s, 3H), 0.90 (s, 9H), 0.09 (s, 6H).

Methyl 6-*O*-(tert-butyldimethylsilyl)-β-ᴅ-glucopyranoside (1b)

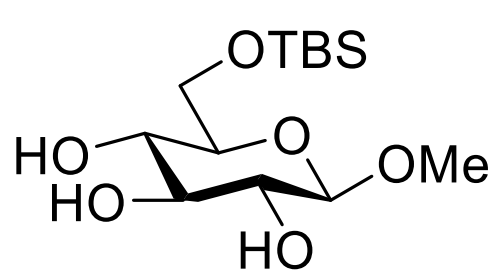

Synthesized according to literature procedure from methyl β-ᴅ-glucopyranoside and *tert*-butyldimethyl silyl chloride.[2]

^{1}H NMR (400 MHz, $CDCl_3$): δ 4.21 (dd, J = 7.7, 0.6 Hz, 1H), 3.94 (ddd, J = 10.5, 5.1, 2.0 Hz, 1H), 3.88–3.80 (m, 1H), 3.62–3.55 (m, 2H), 3.53 (s, 3H), 3.43–3.33 (m, 2H), 0.90 (s, 9H), 0.10 (d, J = 1.4 Hz, 6H).

Methyl 6-*O*-(tert-butyldimethylsilyl)-α-ᴅ-galactopyranoside (1c)

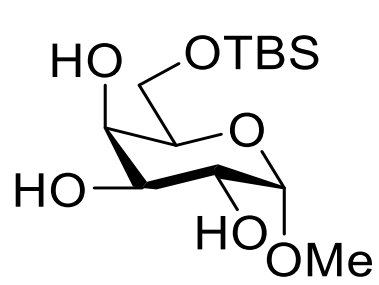

Synthesized according to literature procedure from methyl α-ᴅ-galactopyranoside and *tert*-butyldimethyl silyl chloride.[2]

^{1}H NMR (400 MHz, $CDCl_3$): δ 4.82 (d, J = 3.9 Hz, 1H), 4.10 (dd, J = 3.3, 1.2 Hz, 1H), 3.89 (dd, J = 8.1, 5.2 Hz, 2H), 3.84 (dd, J = 9.7, 4.1 Hz, 1H), 3.76 – 3.71 (m, 2H), 3.42 (s, 3H), 0.90 (s, 9H), 0.10 (s, 6H).

n-Octyl 6-*O*-(tert-butyldimethylsilyl)-α-ᴅ-galactopyranoside (1d)

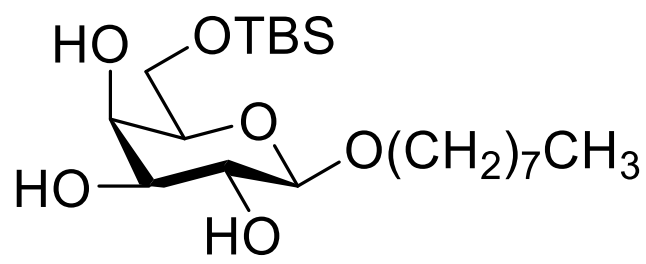

Synthesized according to literature procedure from *n*-octyl β-ᴅ-galactopyranoside and *tert*-butyldimethyl silyl chloride.

^{1}H NMR (400 MHz, CDCl$_3$): δ 4.25 (d, *J* = 7.5 Hz, 1H), 4.06 (dd, *J* = 3.3, 1.1 Hz, 1H), 3.95 (dd, *J* = 10.4, 6.1 Hz, 1H), 3.92–3.87 (m, 1H), 3.67 (dd, *J* = 9.5, 7.5 Hz, 1H), 3.60 (dd, *J* = 9.5, 3.3 Hz, 1H), 3.56–3.49 (m, 1H), 1.65 (p, *J* = 6.9 Hz, 3H), 1.32 (d, *J* = 11.7 Hz, 11H), 0.93 (s, 12H), 0.12 (d, *J* = 2.0 Hz, 6H).

^{13}C NMR (101 MHz, CDCl$_3$): δ 103.1, 74.7, 73.9, 72.1, 70.2, 69.0, 62.6, 32.0, 29.7, 29.5, 29.4, 26.1, 26.0, 22.8, 18.4, 14.2, -5.3.

Methyl 6-*O*-(tert-butyldimethylsilyl)-α-ᴅ-mannopyranoside (1e)

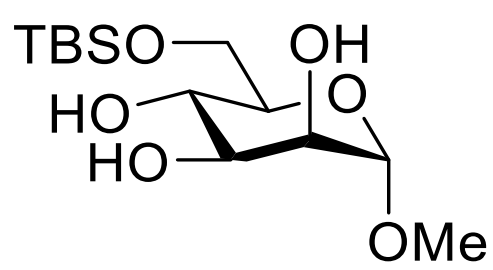

Synthesized according to literature procedure from methyl α-ᴅ-mannopyranoside and *tert*-butyldimethyl silyl chloride.[2]

^{1}H NMR (400 MHz, CDCl$_3$): δ 4.70 (d, *J* = 1.6 Hz, 1H), 3.92 (dd, *J* = 10.6, 4.6 Hz, 2H), 3.86 (dd, *J* = 10.7, 5.6 Hz, 1H), 3.80 (dd, *J* = 9.1, 3.2 Hz, 1H), 3.73 (app t, *J* = 9.4 Hz, 1H), 3.57 (dt, *J* = 9.8, 5.2 Hz, 1H), 3.37 (s, 3H), 0.92 (s, 9H), 0.12 (d, *J* = 1.1 Hz, 6H).

Methyl 4,6-benzylidene α-ᴅ-glucopyranoside (1g)

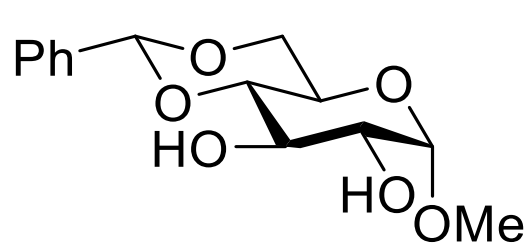

Synthesized according to literature procedure from methyl α-ᴅ-glucopyranoside and benzaldehyde dimethyl acetal.[3]

^{1}H NMR (400 MHz, CDCl$_3$): δ 7.51–7.47 (m, 2H), 7.42–7.31 (m, 3H), 5.53 (s, 1H), 4.79 (d, *J* = 3.9 Hz, 1H), 4.29 (dd, *J* = 9.6, 4.3 Hz, 1H), 3.93 (app t, *J* = 9.2 Hz, 1H), 3.63 (dd, *J* = 9.2, 3.9 Hz, 1H), 3.50 (d, *J* = 9.3 Hz, 1H), 3.46 (s, 3H).

Methyl 4,6-benzylidene α-D-galactopyranoside (1h)

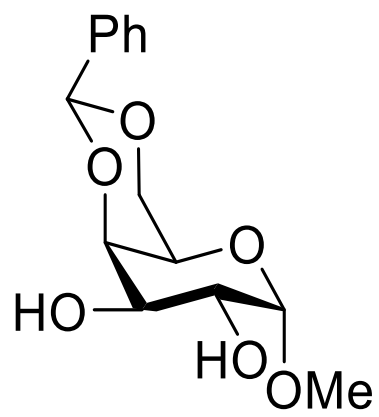

Synthesized according to literature procedure from methyl α-D-galactopyranoside and benzaldehyde dimethyl acetal.[3]

^{1}H NMR (400 MHz, CDCl$_3$): δ 7.55–7.50 (m, 2H), 7.39 (dd, *J* = 5.1, 2.0 Hz, 3H), 5.58 (s, 1H), 4.96 (d, *J* = 3.2 Hz, 1H), 4.35–4.26 (m, 2H), 4.11 (dd, *J* = 12.6, 1.8 Hz, 1H), 3.97–3.91 (m, 2H), 3.73 (d, *J* = 1.7 Hz, 1H), 3.50 (s, 3H).

Ethyl thio-α-L-rhamnopyranoside (1n)

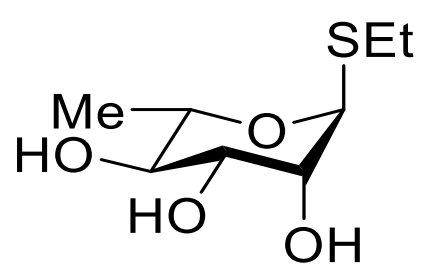

Synthesized according to literature procedure from methyl rhamnose monohydrate.

^{1}H NMR (400 MHz, CD$_3$OD): δ 5.19 (d, *J* = 1.5 Hz, 1H), 3.99–3.89 (m, 2H), 3.62 (dd, *J* = 9.5, 3.4 Hz, 1H), 3.43 (app t, *J* = 9.5 Hz, 1H), 2.77–2.54 (m, 2H), 1.35–1.26 (m, 6H).

^{13}C NMR (101 MHz, CD$_3$OD): δ 86.2, 74.2, 73.9, 73.0, 70.1, 26.0, 17.9, 15.4.

Methyl 6-O-trityl-α-D-glucopyranoside (1s)

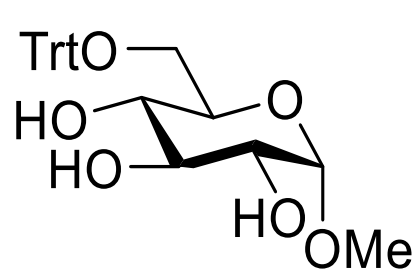

Synthesized according to literature procedure from methyl α-D-glucopyranoside and trityl chloride.[4]

^{1}H NMR (400 MHz, CDCl$_3$): δ 7.48–7.44 (m, 6H), 7.34–7.28 (m, 9H), 4.78 (d, *J* = 3.9 Hz, 1H), 3.73–3.61 (m, 2H), 3.59–3.48 (m, 2H), 3.43 (s, 3H), 3.40 (t, *J* = 4.4 Hz, 2H).

Phenyl 6-O-trityl-β-D-1-thio-galactopyranoside (1s)

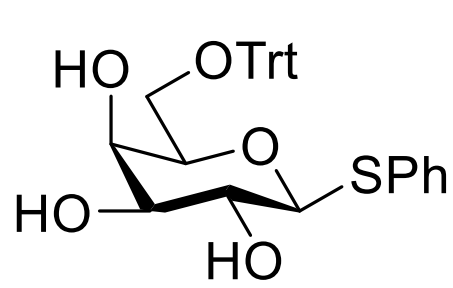

Synthesized according to literature procedure from phenyl 1-thio-β-D-galactopyranoside and trityl chloride.

^{1}H NMR (400 MHz, $CDCl_3$): δ 7.63–7.58 (m, 2H), 7.49–7.43 (m, 6H), 7.33–7.28 (m, 10H), 7.27–7.25 (m, 2H), 4.51 (d, *J* = 9.5 Hz, 1H), 3.99 (s, 1H), 3.65 (app t, *J* = 9.3 Hz, 1H), 3.57 (dt, *J* = 5.4, 2.7 Hz, 2H), 3.50 (dd, *J* = 9.9, 5.6 Hz, 1H), 3.41 (dd, *J* = 9.9, 5.2 Hz, 1H).

^{13}C NMR (101 MHz, $CDCl_3$): δ 143.8, 132.7, 132.4, 129.2, 128.8, 128.1, 128.0, 127.3, 88.7, 87.2, 77.7, 75.0, 70.1, 69.7, 63.7.

Characterization of Products

General Procedure A

To a 2 dram vial was added glycoside (1 equiv), enzyme (1 mol%) and a small magnetic stir bar. A rubber septum was then used to seal the vial. The vial was then evacuated and backfilled with argon on a Schlenk line three times. Dry solvent (0.2 M) and vinyl acetate was then added under a balloon of argon. The rubber septum was removed and quickly replaced with the vial cap. The vial was then sealed and covered with Teflon tape and parafilm. The vial was placed on a hot plate in an oil bath and stirred at 500 rpm for the specified time at the specified temperature After the required amount of time, the crude reaction was concentrated under reduced pressure, then filtered through a plug of celite with a 50:50 v/v mixture of DCM/MeOH to remove the solid enzyme, then concentrated under reduced pressure. Internal standard (1,3,5-trimethoxybenzene) was added, and the material was analyzed by ^{1}H NMR spectroscopy. The crude material was then subjected to flash column chromatography on silica.

General Procedure B

Prepared according to literature procedures.[5]

To a 1 dram vial was added acylated pyranoside derivative (1 equiv.), $(Ir[dF(CF_3)ppy]_2(dtbpy))PF_6$ (1 mol%), quinuclidine (20 mol%), tetrabutylammonium 4-chlorobenzoate (25 mol%), potassium bicarbonate (1 equiv.) and a small magnetic stir bar. A rubber septum was then used to seal the vial. The vial was then evacuated and backfilled with argon on a Schlenk line three times. Dry, degassed acetonitrile (0.125 M) was added under a balloon of argon. The rubber septum was removed and quickly replaced with the vial cap. The vial was then sealed and covered with Teflon tape and parafilm. The vial was placed 5 inches from a blue LED Kessil lamp and stirred at 1050 rpm for 24 hours at 25 °C. After 24 hours the crude reaction was concentrated under reduced pressure and analyzed by 1H NMR spectroscopy. The crude material was then subjected to flash column chromatography on silica.

Methyl 2-*O*-acetyl-6-*O*-(tert-butyldimethylsilyl)-α-ᴅ-glucopyranoside (2a)

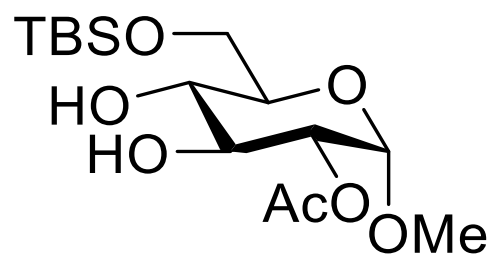

Prepared according to General Procedure A from methyl 2-*O*-acetyl-6-*O*-(*tert*-butyldimethylsilyl)-α-ᴅ-glucopyranoside (61.9 mg, 0.2 mmol) using established optimized conditions. Obtained as a clear colorless oil (70 mg, 99%) after flash column chromatography on silica (2% MeOH in DCM)

^{1}H NMR (400 MHz, CDCl$_3$): δ 4.84 (d, *J* = 3.7 Hz, 1H, H-1), 4.64 (dd, *J* = 10.1, 3.7 Hz, 1H, H-2), 3.92 (dd, *J* = 10.1, 8.5 Hz, 1H, H-3), 3.83 (dd, *J* = 4.6, 2.1 Hz, 2H, H-6), 3.59 (dt, *J* = 9.4, 4.6 Hz, 1H, H-5), 3.55–3.49 (m, 1H, H-4), 3.34 (s, 3H), 2.11 (s, 3H), 0.88 (s, 9H), 0.07 (s, 6H).

^{13}C NMR (101 MHz, CDCl$_3$): δ 171.1, 96.9, 73.4, 72.7, 71.6, 70.4, 64.0, 55.1, 26.0, 21.1, 18.4, –5.3.

Methyl 2-O-acetyl 6-*O*-(tert-butyldimethylsilyl)-α-ᴅ-galactopyranoside (2c)

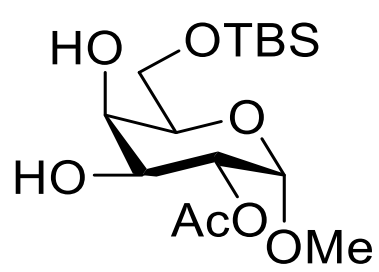

Prepared according to General Procedure A from methyl 2-*O*-acetyl-6-*O*-(*tert*-butyldimethylsilyl)-α-ᴅ-galactopyranoside (62.0 mg, 0.2 mmol). Obtained as a clear colorless oil (66 mg, 94%) after flash column chromatography on silica (2% MeOH in DCM).

^{1}H NMR (400 MHz, CDCl$_3$): δ 5.00 (dd, *J* = 10.2, 3.8 Hz, 1H, H-2), 4.84 (d, *J* = 3.8 Hz, 1H, H-1), 4.04 (dd, *J* = 3.5, 1.3 Hz, 1H, H-4), 3.90 (dd, *J* = 10.2, 3.5 Hz, 1H, H-3), 3.87–3.77 (m, 2H, H-6), 3.74–3.71 (m, 1H, H-5), 3.32 (s, 3H), 2.08 (s, 3H), 0.85 (s, 9H), 0.04 (d, *J* = 0.9 Hz, 6H).

^{13}C NMR (101 MHz, CDCl$_3$): δ 171.5, 97.4, 71.7, 70.1, 69.6, 68.5, 63.1, 55.1, 25.8, 21.1, 18.2, –5.5.

n-Octyl 2-*O*-acetyl 6-*O*-(tert-butyldimethylsilyl)-α-ᴅ-galactopyranoside (2d)

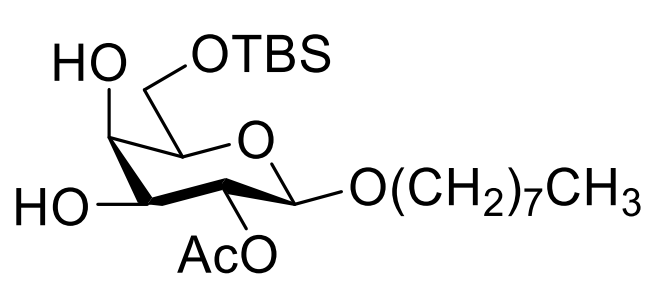

Prepared according to General Procedure A from n-octyl 6-*O*-(*tert*-butyldimethylsilyl)-β-ᴅ-galactopyranoside (81.3 mg, 0.2 mmol). Obtained as a clear colorless oil (80 mg, 89%) after flash column chromatography on silica (40% EtOAc in Hexane).

^{1}H NMR (400 MHz, CDCl$_3$): δ 4.95 (dd, *J* = 9.7, 7.9 Hz, 1H, H-2), 4.36 (d, *J* = 7.9 Hz, 1H, H-1), 4.06–4.01 (br s, 1H), 3.97–3.89 (m, 2H, H-6), 3.86–3.80 (m, 1H, H-3), 3.60 (br s, 1H), 3.49–3.42 (m, 2H, H-4 and H-5), 2.12 (s, 3H), 1.63–1.51 (m, 3H), 1.33–1.23 (m, 11H), 0.90 (d, *J* = 2.5 Hz, 12H).

^{13}C NMR (101 MHz, CDCl$_3$): δ 171.4, 101.0, 74.6, 73.6, 73.2, 69.7, 69.6, 62.8, 31.9, 29.6, 29.4, 29.4, 26.0, 25.9, 22.7, 21.1, 18.4, 14.2, –5.32, –5.34.

4-*O*-acetyl 1,6-β-ᴅ-anhydroglucose (2f)

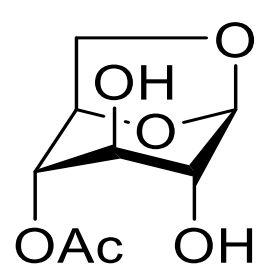

Prepared according to General Procedure A from 1,6-anhydro-β-ᴅ-glucose (33 mg, 0.2 mmol). Obtained as a clear colorless oil (32.6 mg, 80%) after flash column chromatography on silica (2% MeOH in DCM).

^{1}H NMR (400 MHz, (CD$_3$)$_2$SO): δ 5.26 (d, *J* = 4.6 Hz, 1H, OH), 5.18 (app t, *J* = 1.1 Hz, 1H, H-1), 5.14 (d, *J* = 5.1 Hz, 1H, OH), 4.44 (dd, *J* = 5.8, 1.3 Hz, 1H, H-5), 4.41 (dd, *J* = 3.9, 1.5 Hz, 1H, H-4), 3.90 (dd, *J* = 7.2, 1.2 Hz, 1H, H-6), 3.54 (dd, *J* = 7.3, 5.8 Hz, 1H, H-6), 3.40 (ddd, *J* = 3.6, 3.6, 2.3 Hz, 1H, H-3), 3.22 (ddd, *J* = 4.9, 3.3, 1.1 Hz, 1H, H-2), 2.06 (s, 3H).

^{13}C NMR (101 MHz, (CD$_3$)$_2$SO): δ 102.8, 75.0, 74.0, 72.0, 70.9, 65.3, 21.0.

2,4-di-*O*-acetyl 1,6-β-ᴅ-anhydroglucose (2f')

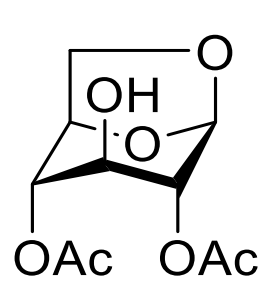

Prepared according to General Procedure A from 1,6-anhydro-β-ᴅ-glucose (32.7 mg, 0.2 mmol). **2.32** was obtained as a clear colorless oil (36 mg, 72%) after flash column chromatography on silica (2% MeOH in DCM).

^{1}H NMR (400 MHz, $(CD_3)_2SO$): δ 5.66 (d, *J* = 4.8 Hz, 1H, OH), 5.31 (d, *J* = 1.2 Hz, 1H, H-1), 4.55 (dt, *J* = 4.6, 1.4 Hz, 1H, H-5), 4.52–4.50 (m, 1H, H-4), 4.40–4.37 (m, 1H, H-6), 4.02 (dd, *J* = 7.4, 1.2 Hz, 1H, H-6), 3.60 (dd, *J* = 7.4, 5.8 Hz, 1H, H-2), 3.51 (dtt, *J* = 4.7, 3.5, 1.2 Hz, 1H, H-3), 2.07 (s, 3H), 2.06 (s, 3H).

^{13}C NMR (101 MHz, $(CD_3)_2SO$): δ 170.1, 169.9, 99.5, 74.0, 73.9, 73.1, 68.0, 65.4, 20.9, 20.8.

Methyl 2-O-acetyl-4,6-benzylidene α-ᴅ-glucopyranoside (2g)

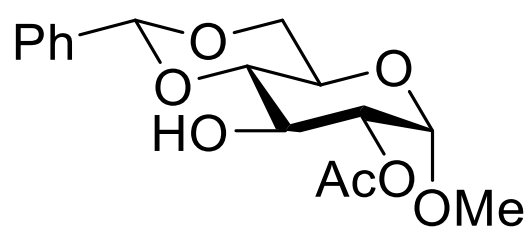

Prepared according to General Procedure A from methyl 4,6-benzylidene α-ᴅ-glucopyranoside (56.8 mg, 0.2 mmol). Obtained as a clear colorless oil (54 mg, 82%) after flash column chromatography on silica (2% MeOH in DCM).

^{1}H NMR (400 MHz, $CDCl_3$): δ 7.52–7.46 (m, 2H), 7.36 (dd, *J* = 5.1, 2.0 Hz, 3H), 5.52 (s, 1H), 4.93 (d, *J* = 3.7 Hz, 1H, H-1), 4.78 (dd, *J* = 9.7, 3.7 Hz, 1H, H-2), 4.27 (dd, *J* = 10.0, 4.6 Hz, 1H, H-6), 4.14 (app t, *J* = 9.5 Hz, 1H, H-3), 3.82 (td, *J* = 9.8, 4.6 Hz, 1H, H-5), 3.73 (app t, *J* = 10.2 Hz, 1H, H-6), 3.51 (app t, *J* = 9.3 Hz, 1H, H-4), 3.38 (s, 3H), 2.13 (s, 3H).

^{13}C NMR (101 MHz, $CDCl_3$): δ 170.8, 137.1, 129.3, 128.4, 126.4, 102.0, 97.6, 81.4, 73.6, 68.9, 68.6, 62.1, 55.4, 21.0.

Methyl 2-*O*-acetyl-α-ᴅ-xylopyranoside (2i)

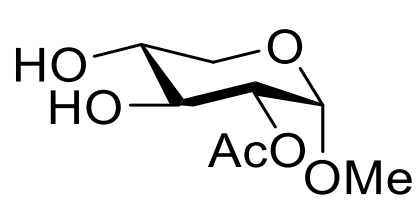

Prepared according to General Procedure A from methyl α-ᴅ-xylopyranoside (32.5 mg, 0.2 mmol). Obtained as a clear colorless oil (33 mg, 81%) after flash column chromatography on silica (2% MeOH in DCM).

^{1}H NMR (400 MHz, CD_3OD): 4.82 (d, *J* = 3.6 Hz, 1H, H-1), 4.60 (dd, *J* = 9.9, 3.6 Hz, 1H, H-2), 3.81–3.74 (m, 1H, H-3), 3.66–3.55 (m, 2H, H-4 and H-5), 3.51–3.46 (m, 1H, H-5), 3.39 (s, 3H), 2.13 (s, 3H).

^{13}C NMR (101 MHz, CD_3OD): 172.4, 98.6, 75.0, 72.5, 71.5, 62.5, 55.5, 20.8.

Methyl 4-*O*-acetyl-β-L-fucopyranoside (2j)

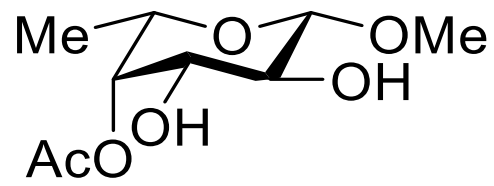

Prepared according to General Procedure A from methyl β-L-fucopyranoside (35.4 mg, 0.2 mmol). Obtained as a white powder (44 mg, 100%) after flash column chromatography on silica (2% MeOH in DCM).

^{1}H NMR (400 MHz, CD_3OD): δ 5.18 (dd, J = 3.7, 1.1 Hz, 1H, H-4), 4.24 (d, J = 7.7 Hz, 1H, H-1), 3.87 (qd, J = 6.5, 1.1 Hz, 1H, H-5), 3.73 (dd, J = 9.9, 3.7 Hz, 1H, H-3), 3.58 (s, 3H), 3.49 (dd, J = 9.9, 7.7 Hz, 1H, H-2), 2.20 (s, 3H), 1.20 (d, J = 6.5 Hz, 3H).

^{13}C NMR (101 MHz, CD_3OD): δ 172.7, 105.7, 74.7, 73.2, 72.4, 70.5, 57.3, 20.8, 16.7.

Methyl 2-*O*-acetyl-β-L-arabinopyranoside (2k)

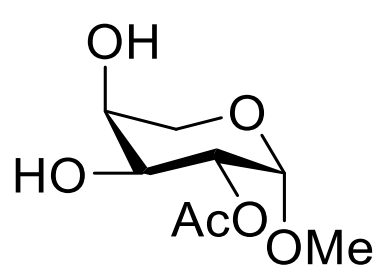

Prepared according to General Procedure A from methyl β-L-arabinopyranoside (32.5 mg, 0.2 mmol). Obtained as a clear colorless oil (32 mg, 79%) after flash column chromatography on silica (2% MeOH in DCM).

^{1}H NMR (400 MHz, CD_3OD): 5.03 (dd, J = 9.8, 3.6 Hz, 1H, H-2), 4.89 (d, J = 3.6 Hz, 1H, H-2), 4.02–3.92 (m, 2H, H-3 and H-4), 3.86 (dd, J = 12.4, 1.5 Hz, 1H, H-5), 3.66 (dd, J = 12.4, 2.2 Hz, 1H, H-5), 3.40 (s, 3H), 2.13 (s, 3H).

^{13}C NMR (101 MHz, CD_3OD): 172.6, 99.1, 72.7, 70.8, 68.1, 63.8, 55.6, 20.8.

Methyl 4-*O*-acetyl-β-D-arabinopyranoside (2l)

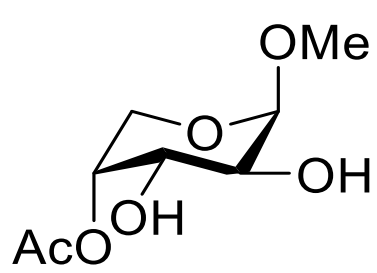

Prepared according to General Procedure A from methyl β-D-arabinopyranoside (32.2 mg, 0.2 mmol). Obtained as a clear colorless oil (38.4 mg, 95%) after flash column chromatography on silica (2% MeOH in DCM).

^{1}H NMR (400 MHz, CD$_3$OD): δ 5.15 (app p, 1H, H-4), 4.79 (d, *J* = 3.6 Hz, 1H, H-1), 3.98 (dd, *J* = 10.0, 3.6 Hz, 1H, H-3), 3.91 (dd, *J* = 13.1, 1.2 Hz, 1H, H-5), 3.84 (dd, *J* = 10.0, 3.6 Hz, 1H, H-2), 3.69 (dd, *J* = 13.1, 2.2 Hz, 1H, H-5), 3.48 (s, 3H), 2.18 (s, 3H).

^{13}C NMR (101 MHz, CD$_3$OD): δ 172.5, 101.8, 73.5, 70.5, 68.9, 61.5, 55.9, 21.0.

Methyl 4-*O*-acetyl-α-L-rhamnopyranoside (2m)

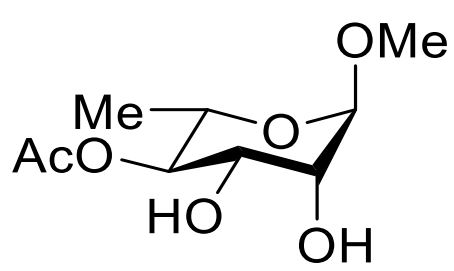

Prepared according to General Procedure A from methyl α-L-rhamnopyranoside (35.8 mg, 0.2 mmol). Obtained as a clear colorless oil (35 mg, 78%) after flash column chromatography on silica (2% MeOH in DCM).

^{1}H NMR (400 MHz, CD$_3$OD): δ 4.77 (app t, *J* = 9.7 Hz, 1H, H-4), 4.63 (d, *J* = 1.6 Hz, 1H, H-1), 3.86 (dd, *J* = 3.5, 1.6 Hz, 1H, H-2), 3.77 (dd, *J* = 9.7, 3.5 Hz, 1H, H-3), 3.68 (dq, *J* = 9.7, 6.4 Hz, 1H, H-5), 3.30 (s, 3H), 2.05 (s, 3H), 1.13 (d, *J* = 6.4 Hz, 3H).

^{13}C NMR (101 MHz, CD$_3$OD): δ 172.5, 102.6, 75.5, 72.1, 70.3, 67.4, 55.2, 21.0, 17.8.

Ethyl 1-thio-4-*O*-acetyl-α-L-rhamnopyranoside (2n)

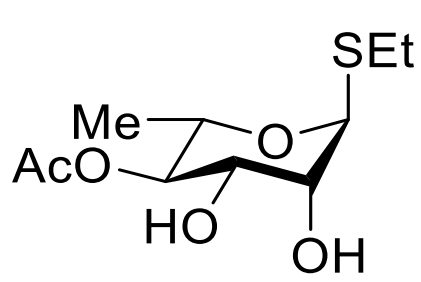

Prepared according to General Procedure A from methyl α-L-1-thio-rhamnopyranoside (41.7 mg, 0.2 mmol). Obtained as a clear colorless oil (28 mg, 56%) after flash column chromatography on silica (50% EtOAc in hexane).

^{1}H NMR (400 MHz, CD$_3$OD): δ 5.23 (d, *J* = 1.5 Hz, 1H, H-1), 4.98 (app t, *J* = 9.7 Hz, 1H, H-4), 4.10 (dq, *J* = 9.7, 6.3 Hz, 1H, H-5), 3.96 (dd, *J* = 3.4, 1.5 Hz, 1H, H-2), 3.76 (dd, *J* = 9.7, 3.4 Hz, 1H, H-3), 2.75–2.54 (m, 2H), 2.11 (s, 3H), 1.31 (t, *J* = 7.4 Hz, 3H), 1.17 (d, *J* = 6.3 Hz, 3H).

^{13}C NMR (101 MHz, CD$_3$OD): δ 172.4, 86.2, 75.8, 73.9, 71.0, 68.0, 26.1, 21.0, 17.7, 15.4.

Methyl 6-*O*-acetyl-α-ᴅ-glucopyranoside (2o)

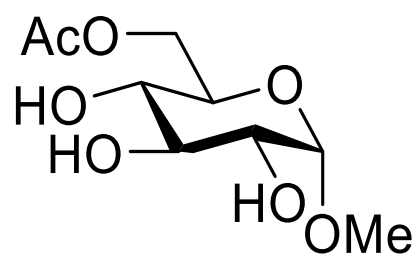

Prepared according to General Procedure A from methyl α-ᴅ-glucopyranoside (38.8 mg, 0.2 mmol). Obtained as a clear colorless oil (33 mg, 70%) after flash column chromatography on silica (2% MeOH in DCM).

^{1}H NMR (400 MHz, CD$_3$OD): δ 4.56 (d, *J* = 3.7 Hz, 1H, H-1), 4.26 (dd, *J* = 11.8, 2.2 Hz, 1H, H-6), 4.10 (dd, *J* = 11.8, 6.0 Hz, 1H, H-6), 3.61 (ddd, *J* = 10.1, 6.0, 2.2 Hz, 1H, H-5), 3.52 (dd, *J* = 9.7, 8.8 Hz, 1H, H-3), 3.31 (app d, *J* = 3.7 Hz, 1H, H-2), 3.30 (s, 3H), 3.19 (dd, *J* = 10.1, 8.8 Hz, 1H, H-4), 1.97 (s, 3H).

^{13}C NMR (101 MHz, CD$_3$OD): δ 172.8, 101.2, 74.9, 73.3, 71.7, 70.9, 64.9, 55.6, 20.7.

Methyl 6-*O*-acetyl-β-ᴅ-galactopyranoside (2p)

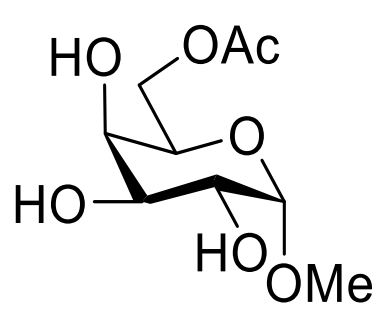

Prepared according to General Procedure A from methyl β-ᴅ-galactopyranoside (38.8 mg, 0.2 mmol). Obtained as a clear colorless oil (45 mg, 95%) after flash column chromatography on silica (10% MeOH in DCM).

^{1}H NMR (400 MHz, CD$_3$OD): δ 4.39 (dd, *J* = 11.3, 7.3 Hz, 1H, H-6), 4.32 (dd, *J* = 11.3, 5.1 Hz, 1H, H-6), 4.26–4.23 (m, 1H, H-3), 3.92 (d, *J* = 1.5 Hz, 1H, H-1), 3.83 (ddd, *J* = 7.3, 5.1, 1.2 Hz, 1H, H-5), 3.61 (s, 3H), 3.61–3.58 (m, 2H, H-2 and H-4), 2.16 (s, 3H).

^{13}C NMR (101 MHz, CD$_3$OD): δ 172.6, 105.8, 74.6, 73.8, 72.2, 70.2, 64.7, 57.2, 20.7.

Isopropyl 6-*O*-acetyl-β-ᴅ-1-thio-galactopyranoside (2q)

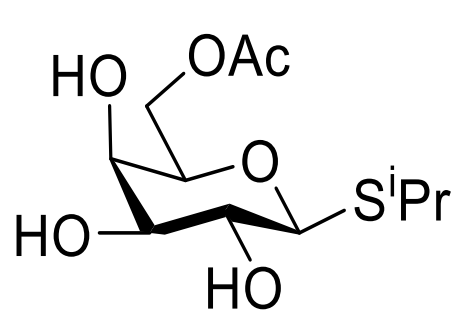

Prepared according to General Procedure A from isopropyl β-D-1-thio-galactopyranoside (47.6 mg, 0.2 mmol). **2.29** was obtained as a clear colorless oil (53 mg, 92%) after flash column chromatography on silica (0-10% MeOH in DCM).

^{1}H NMR (400 MHz, CD_3OD): δ 4.58–4.51 (m, 1H, H-1), 4.40 (dd, *J* = 11.5, 7.8 Hz, 1H, H-6), 4.28 (dd, *J* = 11.5, 4.5 Hz, 1H, H-6), 4.01–3.96 (m, 1H, H-4), 3.86 (ddd, *J* = 7.8, 4.5, 1.0 Hz, 1H, H-5), 3.62 (dd, *J* = 5.5, 2.1 Hz, 2H, H-2 and H-3), 3.30 (hept, *J* = 6.7 Hz, 1H), 2.15 (s, 3H), 1.43 (dd, *J* = 6.7, 4.3 Hz, 6H).

^{13}C NMR (101 MHz, CD_3OD): δ 172.5, 87.0, 77.4, 75.9, 71.3, 70.3, 65.0, 36.2, 24.6, 24.2, 20.7.

Methyl 6-*O*-acetyl-α-D-mannopyranoside (2r)

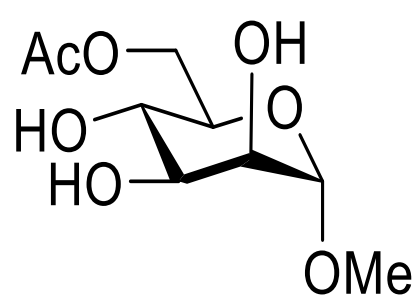

Prepared according to General Procedure A from methyl α-D-mannopyranoside (38.8 mg, 0.2 mmol). Obtained as a clear colorless oil (42 mg, 89%) after flash column chromatography on silica (5-10% MeOH in DCM).

^{1}H NMR (400 MHz, CD_3OD): δ 4.70 (d, *J* = 1.7 Hz, 1H, H-1), 4.47 (dd, *J* = 11.7, 1.6 Hz, 1H, H-6), 4.30 (dd, *J* = 11.7, 6.1 Hz, 1H, H-6), 3.88 (dd, *J* = 3.0, 1.7 Hz, 1H, H-2), 3.77–3.67 (m, 3H, H-3 and H-4 and H-5), 3.45 (s, 3H), 2.15 (s, 3H).

^{13}C NMR (101 MHz, CD_3OD): δ 172.9, 102.7, 72.4, 71.8, 71.8, 68.5, 65.1, 55.2, 20.7.

Methyl 6-*O*-trityl-2-*O*-acetyl-α-D-glucopyranoside (2s)

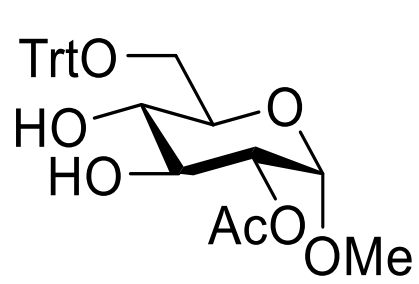

Prepared according to General Procedure A from methyl 6-*O*-trityl α-D-glucopyranoside (87.3 mg, 0.2 mmol). Obtained as a clear colorless oil (82 mg, 82% adjusted for 95% purity) after flash column chromatography on silica (50-70% EtOAc in hexane).

^{1}H NMR (400 MHz, CDCl$_3$): δ 7.42–7.37 (m, 6H), 7.26–7.21 (m, 6H), 7.19–7.13 (m, 3H), 4.84 (d, *J* = 3.7 Hz, 1H, H-1), 4.64 (dd, *J* = 10.1, 3.7 Hz, 1H, H-2), 3.85 (dd, *J* = 10.1, 8.7 Hz, 1H, H-3), 3.60 (m, 1H, H-5), 3.50 (dd, *J* = 9.8, 8.7 Hz, 1H, H-4), 3.33 (m, 2H, H-6), 3.31 (s, 3H), 2.07 (s, 3H).

^{13}C NMR (101 MHz, CDCl$_3$): δ 171.1, 143.8, 128.8, 128.1, 127.3, 97.0, 87.3, 77.5, 77.2, 76.8, 73.4, 72.6, 71.6, 69.5, 64.1, 55.3, 21.1.

Methyl 2,6-di-*O*-acetyl-β-ᴅ-xylopyranoside (2t)

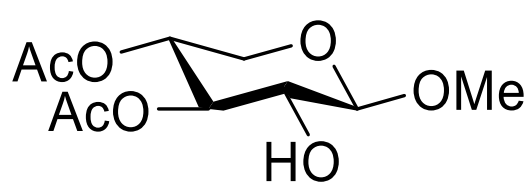

Prepared according to General Procedure A from methyl β-ᴅ-xylopyranoside (32.8 mg, 0.2 mmol). Obtained as a white solid (36 mg, 73%) after flash column chromatography on silica (50% EtOAc in hexane).

^{1}H NMR (400 MHz, CDCl$_3$): δ 5.05 (app t, *J* = 9.3 Hz, 1H, H-3), 4.87–4.80 (m, 1H, H-4), 4.26 (d, *J* = 7.4 Hz, 1H, H-1), 4.01 (dd, *J* = 11.6, 5.5 Hz, 1H, H-5), 3.50 (s, 3H), 3.38 (app td, *J* = 9.8, 2.3 Hz, 2H, H-2 and H-5), 2.05 (s, 3H), 2.00 (s, 3H).

^{13}C NMR (101 MHz, CDCl$_3$): δ 172.1, 171.7, 105.7, 75.7, 72.9, 70.9, 63.5, 57.3, 20.8, 20.6.

Phenyl 6-*O*-acetyl-α-ᴅ-1-thio-mannopyranoside (2v)

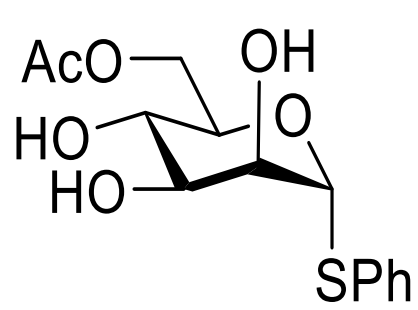

Prepared according to General Procedure A from phenyl α-ᴅ-thio-mannopyranoside (54.4 mg, 0.2 mmol). Obtained as a yellow oil (53 mg, 84%) after flash column chromatography on silica (5-10% MeOH in DCM).

^{1}H NMR (400 MHz, CDCl$_3$): δ 7.50–7.39 (m, 2H), 7.23 (dd, *J* = 5.1, 2.0 Hz, 3H), 5.55 (d, *J* = 1.3 Hz, 1H, H-1), 4.43 (dd, *J* = 12.0, 7.0 Hz, 1H, H-6), 4.34 (m, 2H, H-4 and H-6), 4.25 (dd, *J* = 3.3, 1.3 Hz, 1H, H-2), 3.88 (dd, *J* = 9.5, 3.3 Hz, 1H, H-3), 3.79 (app t, *J* = 9.6 Hz, 1H, H-5), 1.97 (s, 3H).

^{13}C NMR (101 MHz, CDCl$_3$): δ 172.1, 133.7, 131.6, 129.1, 127.6, 87.9, 72.2, 72.2, 71.4, 68.3, 64.0, 21.0.

Methyl 2-deoxy-6-*O*-(tert-butyldimethylsilyl)-*α*-D-erythro-hexopyranoside-3-ulose (3a)

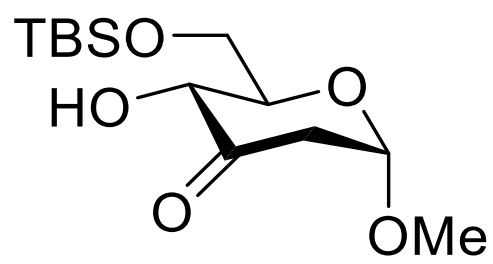

Prepared according to General Procedure B from methyl 2-*O*-acetyl-6-*O*-(*tert*-butyldimethylsilyl)-α-D-glucopyranoside (20 mg, 0.1 mmol). Obtained as a clear colorless oil (15 mg, 83%) after flash column chromatography on silica (10% acetone in DCM).

^{1}H NMR (400 MHz, $CDCl_3$): δ (ppm) = 5.15 (d, *J* = 4.4 Hz, 1H), 4.20 (dd, *J* = 9.9, 1.2 Hz, 1H), 4.04–3.91 (m, 2H), 3.71 (dddd, *J* = 9.9, 4.5, 2.2, 0.6 Hz, 1H), 3.36 (s, 3H), 2.88 (ddd, *J* = 14.2, 4.5, 1.3 Hz, 1H), 2.53 (dd, *J* = 14.2, 1.1 Hz, 1H), 0.96 (s, 9H), 0.15 (s, 6H).

^{13}C NMR (101 MHz, $CDCl_3$): δ (ppm) = 207.3, 101.1, 76.5, 74.1, 64.1, 55.0, 46.7, 26.4, 19.3, -5.1.

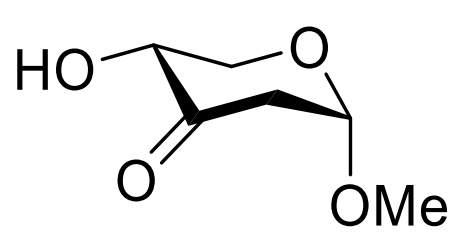

Prepared according to General Procedure B in MeCN-d3 from methyl 2-*O*-acetyl-α-D-xylopyranoside (11 mg, 0.1 mmol). Obtained as a crude mixture.

^{1}H NMR (400 MHz, CD_3CN): δ 5.11 (d, *J* = 4.4 Hz, 1H), 4.29 (ddd, *J* = 10.7, 7.7, 1.3 Hz, 1H), 4.03 (dd, *J* = 10.5, 7.7 Hz, 1H), 3.55 (td, *J* = 10.5, 0.6 Hz, 1H), 3.31 (s, 3H), 2.84 (ddd, *J* = 14.1, 4.4, 1.3 Hz, 1H), 2.46 (dd, *J* = 14.1, 1.2 Hz, 1H).

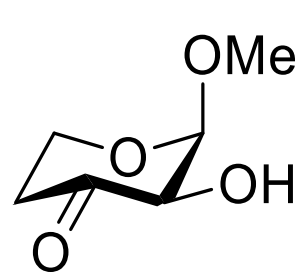

Prepared according to General Procedure B from methyl 4-*O*-acetyl-α-D-arabinopyranoside (11 mg, 0.1 mmol). Obtained as a crude mixture

^{1}H NMR (400 MHz, $CDCl_3$): δ 5.04 (d, *J* = 4.0 Hz, 1H), 4.31 (dd, *J* = 4.0, 1.4 Hz, 2H), 3.98 – 3.93 (m, 2H), 3.36 (s, 4H), 2.77 (dddd, *J* = 14.1, 12.0, 8.3, 1.3 Hz, 2H), 2.35 (dddd, *J* = 14.1, 3.0, 1.2, 0.5 Hz, 2H).

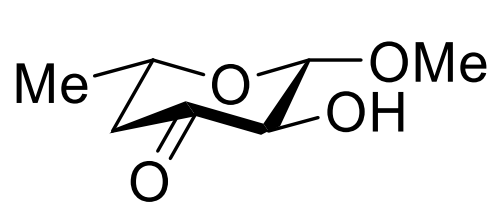

Prepared according to General Procedure B from methyl 4-*O*-acetyl-β-D-fucopyranoside (22 mg, 0.1 mmol). Obtained as a crude mixture

^{1}H NMR (400 MHz, CDCl$_3$): δ 4.24 (d, *J* = 7.9 Hz, 1H), 3.94 – 3.89 (m, 1H), 3.77 – 3.71 (m, 1H), 3.52 (s, 3H), 2.48 – 2.43 (m, 2H), 1.33 (d, *J* = 6.1 Hz, 3H).

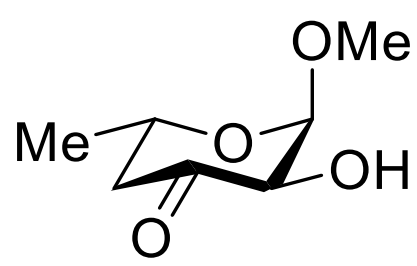

Prepared according to General Procedure B from methyl 4-*O*-acetyl-α-D-rhamnoopyranoside (22 mg, 0.1 mmol). Obtained as a crude mixture

^{1}H NMR (400 MHz, CDCl$_3$): δ 5.01 (d, 1H), 4.30 – 4.28 (m, 1H), 4.14 – 4.05 (m, 1H), 3.36 (s, 3H), 2.47 – 2.41 (m, 2H), 1.29 (d, *J* = 6.2 Hz, 3H).

Python Code for Parameter Generation

```
import pandas as pd
from edbo.feature_utils import build_experiment_index

# Build data sets from indices

def enzyme(enzymeID='ohe', solvent='ohe'):
    """
    Load data with different features.
    """

    # Experiment index

    index = pd.read_csv('data/enzyme/experiment_index.csv')

    # Enzyme features

    if enzyme_type == 'ohe':
        enzyme_features = pd.read_csv('data/enzyme/enzyme_ohe.csv')

    # Solvent features

    if solvent_type == 'ohe':
        solvent_features = pd.read_csv('data/enzyme/solvent_ohe.csv')

    # Build the descriptor set

    index_list = [index['enzymeID'], index['solventID']]

    lookup_table_list = [enzyme_features, solvent_features]

    lookup_list = ['enzymeID', 'solventID']

    experiment_index = build_experiment_index(index['entry'],
                                              index_list,
                                              lookup_table_list,
                                              lookup_list)

    experiment_index['enzyme loading'] = index['enzyme_loading']
    experiment_index['time'] = index['time_hrs']
    experiment_index['temperature'] = index['temp_C']
    experiment_index['yield'] = index['yield']

    return experiment_index

print('loadup')
```

Python script for loading one-hot encoding data, will be called on by other scripts using 'from data-loader import enzyme'.

```python
from edbo.bro import BO_express
from edbo.bro import BO
from data_loader import enzyme
import pandas as pd

# (0) Define all possible parameters

enzyme_name = ['ppl', 'pfl', 'psl']

solvent_name = ['MeCN', 'THF', 'Pyridine', 'THF4Pyl']

acyl_loading = [5, 10, 15, 20, 25, 30, 35, 40, 45, 50, 55]

times = [6, 12, 18, 24, 36, 48, 72]

temperatures = [20, 25, 30, 35, 40, 45, 50, 55, 60, 65, 70]

# (1) Define a dictionary of components

reaction_components={'enzymeID':enzyme_name,
    'solventID':solvent_name,
    'acyl equivalents':acyl_loading,
    'time':times,
    'temperature': temperatures}

# (2) Define a dictionary of desired encodings

encoding={'enzymeID':'ohe',        #ohe one-hot-encoding for non-numeric (need to make identity matrix
          'solventID':'ohe',
          'acyl equivalents':'numeric',
          'time':'numeric',
          'temperature':'numeric'}

# (3) Call the Bayesian program

bo = BO_express(reaction_components,
                encoding,
                batch_size=8,
                init_method='rand',
                target='yield')

# (4) Generate random experiments
#bo.add_results(results_path=None) #this will make the full 20000 entry spreadsheet
bo.init_sample()  # Initialize with a random sample. if include (seed=x)where x is an integer will generate same dataset
bo.export_proposed('experiments.csv')     # Write the proposed experiments to experiments.csv in dir
bo.get_experiments()                 # Get a DataFrame of the proposed experiments

print('success')
```

Python code for generating new, random experimental dataset for initial experiments.

```python
from edbo.bro import BO_express
from edbo.bro import BO
from data_loader import enzyme
import pandas as pd

# (0) Define all possible parameters
enzyme_name = ['ppl', 'pfl', 'psl']
solvent_name = ['MeCN', 'THF', 'Pyridine', 'THF4Pyl']
acyl_loading = [5, 10, 15, 20, 25, 30, 35, 40, 45, 50, 55]
times = [6, 12, 18, 24, 36, 48, 72]
temperatures = [20, 25, 30, 35, 40, 45, 50, 55, 60, 65, 70]

# (1) Define a dictionary of components
reaction_components={'enzymeID':enzyme_name,
    'solventID':solvent_name,
    'acyl equivalents':acyl_loading,
    'time':times,
    'temperature': temperatures}

# (2) Define a dictionary of desired encodings
encoding={'enzymeID':'ohe',        #ohe one-hot-encoding for non-numeric (need to make identity matrix
          'solventID':'ohe',
          'acyl equivalents':'numeric',
          'time':'numeric',
          'temperature':'numeric'}

# (3) Call the Bayesian program
uo = BO_express(reaction_components,
                encoding,
                batch_size=2,
                acquisition_function='UCB',
                target='yield')

ego = BO_express(reaction_components,
                encoding,
                batch_size=2,
                acquisition_function='ε-greedy(ε=0.1)',
                target='yield')

# (4) Generate next experiments based on data
uo.add_results('experiments.csv')
uo.run()
print('Upper Confidence Bound...')
uo.model.regression()
uo.export_proposed('UCB_experiments.csv')

ego.add_results('experiments.csv')
ego.run()
print('ε-greedy...')
ego.model.regression()
ego.export_proposed('e_greedy_experiments.csv')

print('done')
```

Python code for running Bayesian optimization to generate Bayesian experiments.

Bayesian Optimization Data Tables

All yields are determined from crude ^{1}H NMR spectroscopic analysis, integrated against the internal standard 1,3,5-trimethoxybenzene. Enzymes used were Porcine Pancreatic Lipase (PPL), Amano Lipase PS from Burkholderia cepacia (PSL), Amano Lipase from Pseudomonas fluorescens (PFL).

2a – Methyl 2-*O*-acetyl-6-*O*-(tert-butyldimethylsilyl)-α-ᴅ-glucopyranoside

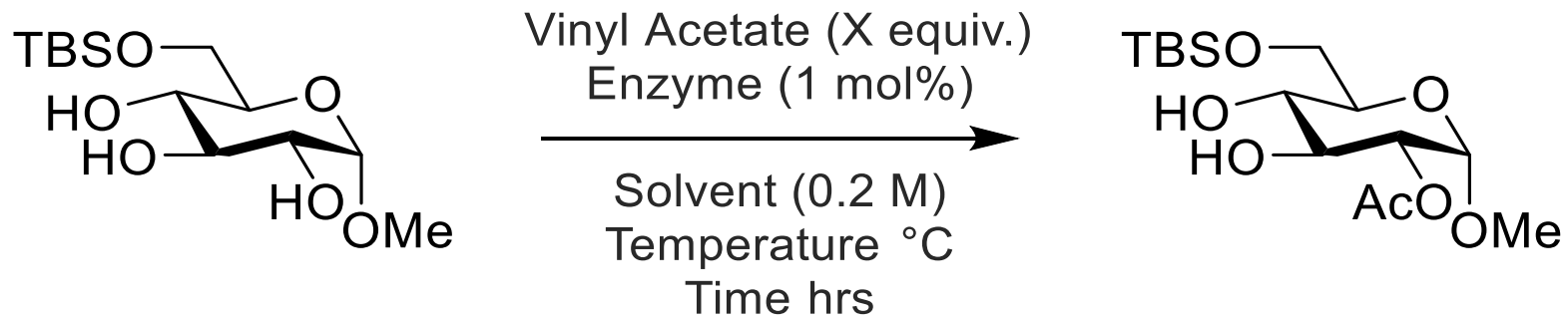

Round of Bayesian	Experiment Type	Enzyme	Solvent	Acyl equiv.	Time (hrs)	Temp (°C)	Yield (%)
Round 0	Random 1	PPL	MeCN	55	48	20	24
	Random 2	PSL	THF/ Py (4:1)	25	24	25	2
	Random 3	PSL	THF/ Py (4:1)	55	24	20	2
	Random 4	PPL	Pyridine	35	48	70	7
	Random 5	PFL	THF	55	72	70	77
	Random 6	PSL	MeCN	30	18	60	20
	Random 7	PPL	MeCN	5	12	60	2
	Random 8	PSL	THF/ Py (4:1)	15	48	40	17
Round 1	**Bayesian (ε-greedy) 1**	**PFL**	**THF**	**55**	**72**	**65**	**97**
	Bayesian (ε-greedy) 2	PFL	THF	50	72	70	82
	Bayesian (UCB) 1	PFL	Pyridine	5	72	20	13
	Bayesian (UCB) 2	PSL	THF	5	72	20	10

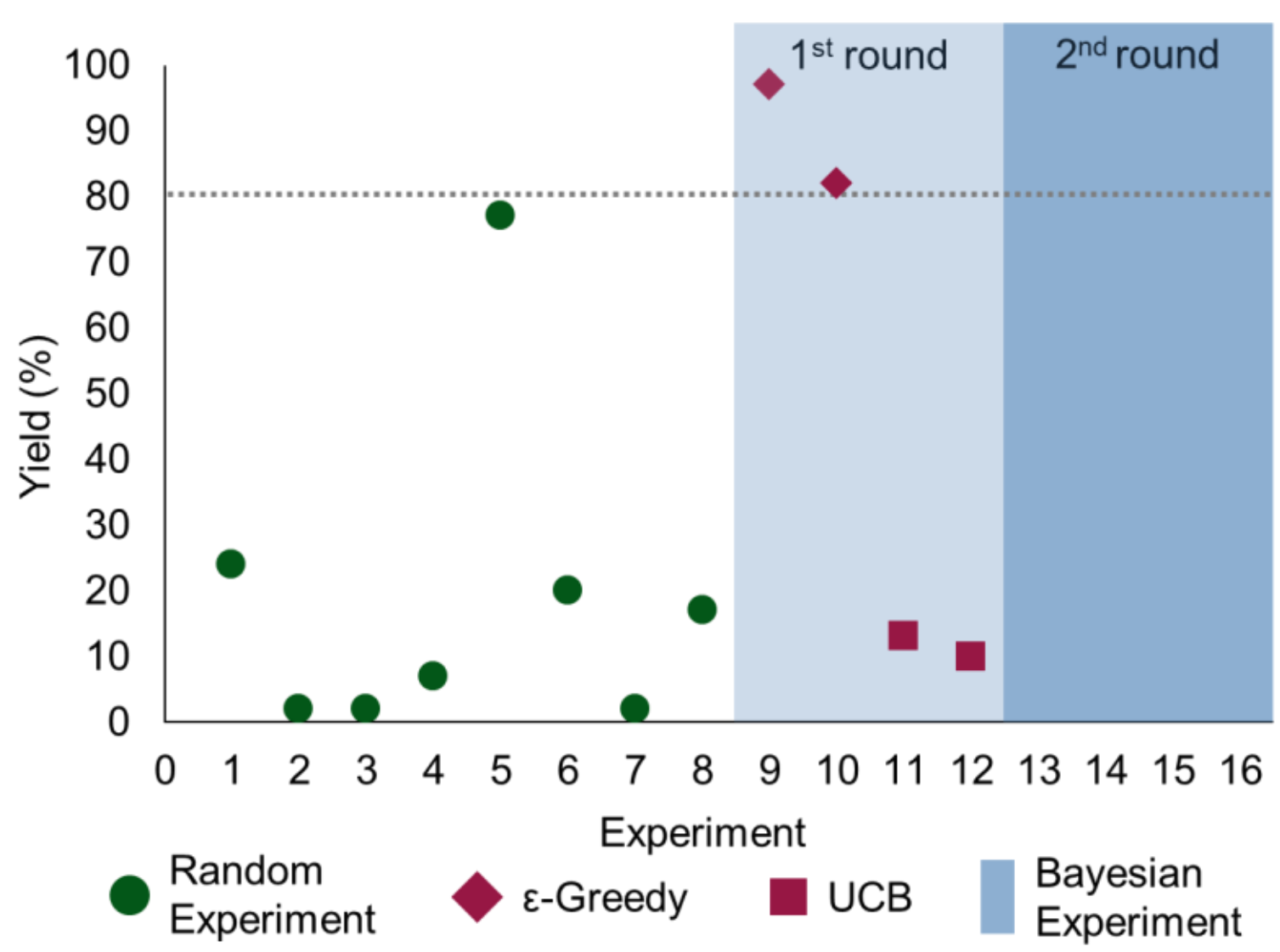

Figure S1

2b – Methyl 3-*O*-acetyl-6-*O*-(tert-butyldimethylsilyl)-β-ᴅ-glucopyranoside

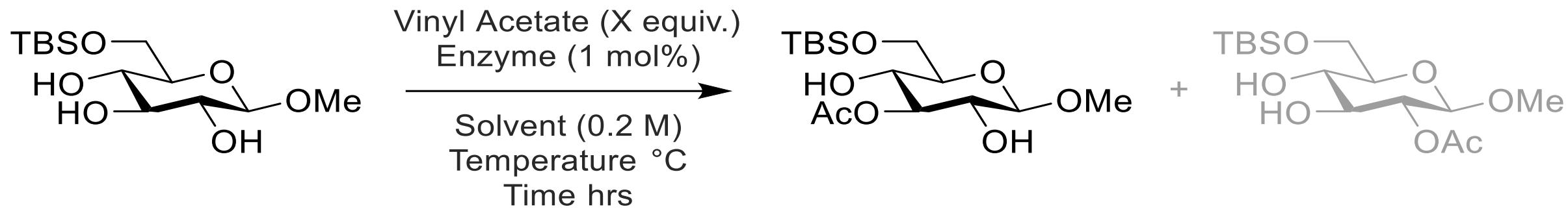

Round of Bayesian	Experiment Type	Enzyme	Solvent	Acyl equiv.	Time (hrs)	Temp (°C)	Yield (%)
Round 0	Random 1	PPL	THF/Py (4:1)	45	18	20	1
	Random 2	PSL	MeCN	45	12	35	12
	Random 3	PFL	THF/Py (4:1)	10	18	35	18
	Random 4	PFL	Pyridine	30	72	45	25
	Random 5	PFL	MeCN	40	18	50	43
	Random 6	PFL	THF	30	36	55	60
	Random 7	PPL	MeCN	15	6	65	1
	Random 8	PPL	THF/Py (4:1)	20	12	50	3
Round 1	**Bayesian (ε-greedy) 1**	**PFL**	**THF**	**45**	**36**	**65**	**64**
	Bayesian (ε-greedy) 2	PFL	THF	45	36	70	62
	Bayesian (UCB) 1	PSL	THF	5	72	70	10
	Bayesian (UCB) 2	PFL	THF	55	72	20	39
Round 2	Bayesian (ε-greedy) 1	PFL	THF	55	6	70	27
	Bayesian (ε-greedy) 2	PFL	THF	50	6	70	14
	Bayesian (UCB) 1	psl	Pyridine	55	6	70	0
	Bayesian (UCB) 2	PFL	THF	55	6	20	21

Resulted in a mixture of regioisomers

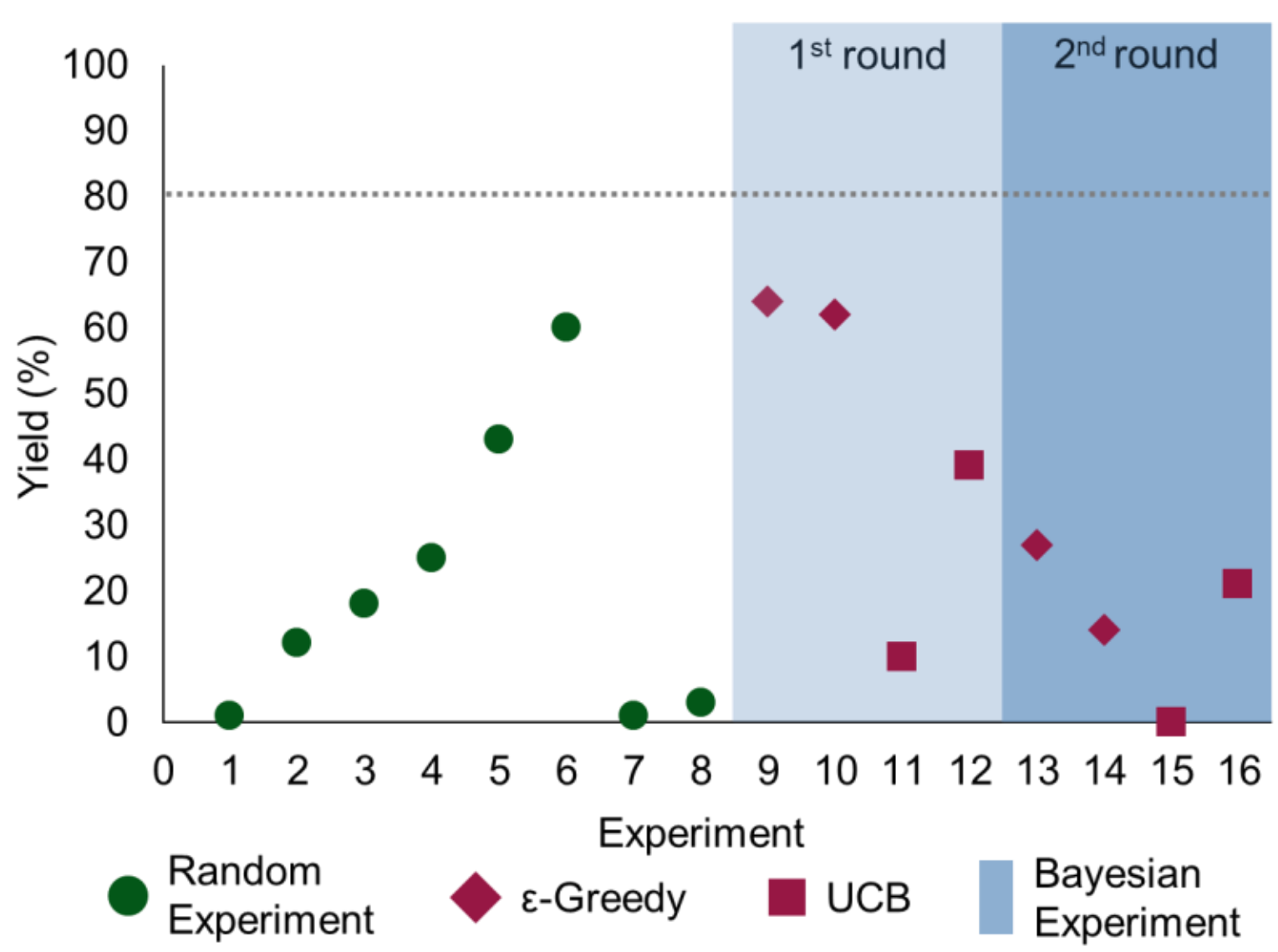

Figure S2

2c – Methyl 2-*O*-acetyl-6-*O*-(tert-butyldimethylsilyl)-α-ᴅ-galactopyranoside

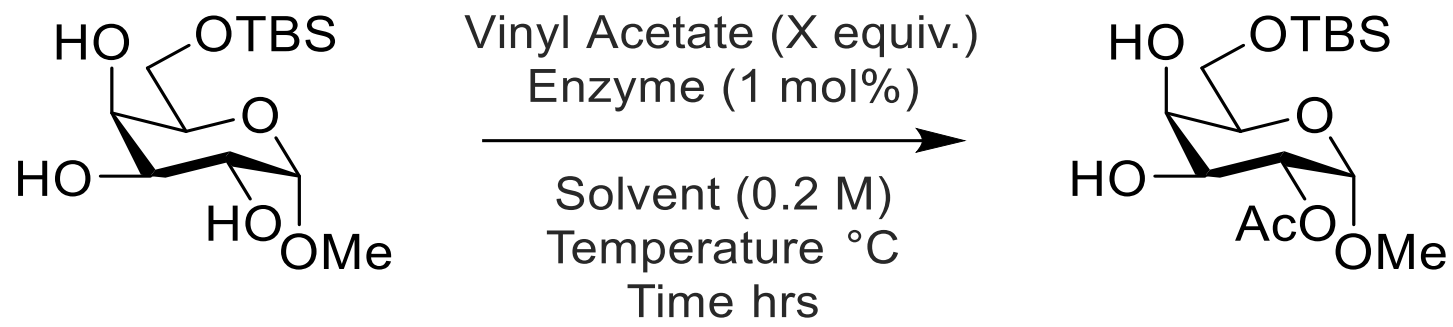

Round of Bayesian	Experiment Type	Enzyme	Solvent	Acyl equiv.	Time (hrs)	Temp (°C)	Yield (%)
Round 0	Random 1	PPL	THF	25	18	30	0
	Random 2	PFL	Pyridine	55	72	35	83
	Random 3	PSL	THF/Py (4:1)	45	36	45	69
	Random 4	PSL	MeCN	25	6	50	1
	Random 5	PSL	Pyridine	25	12	50	9
	Random 6	PPL	THF/Py (4:1)	10	18	60	0
	Random 7	PFL	THF/Py (4:1)	50	18	60	86
	Random 8	PSL	Pyridine	10	36	65	0
Round 1	**Bayesian (ε-greedy) 1**	**PFL**	**THF/Py (4:1)**	**55**	**72**	**35**	**92**
	Bayesian (ε-greedy) 2	PFL	THF/Py (4:1)	55	72	40	90
	Bayesian (UCB) 1	PFL	THF/Py (4:1)	5	72	20	88
	Bayesian (UCB) 2	PFL	THF	55	72	70	83
Round 2	Bayesian (ε-greedy) 1	PPL	THF	25	18	30	0
	Bayesian (ε-greedy) 2	PFL	Pyridine	55	72	35	83
	Bayesian (UCB) 1	PSL	THF/Py (4:1)	45	36	45	69
	Bayesian (UCB) 2	PSL	MeCN	25	6	50	1

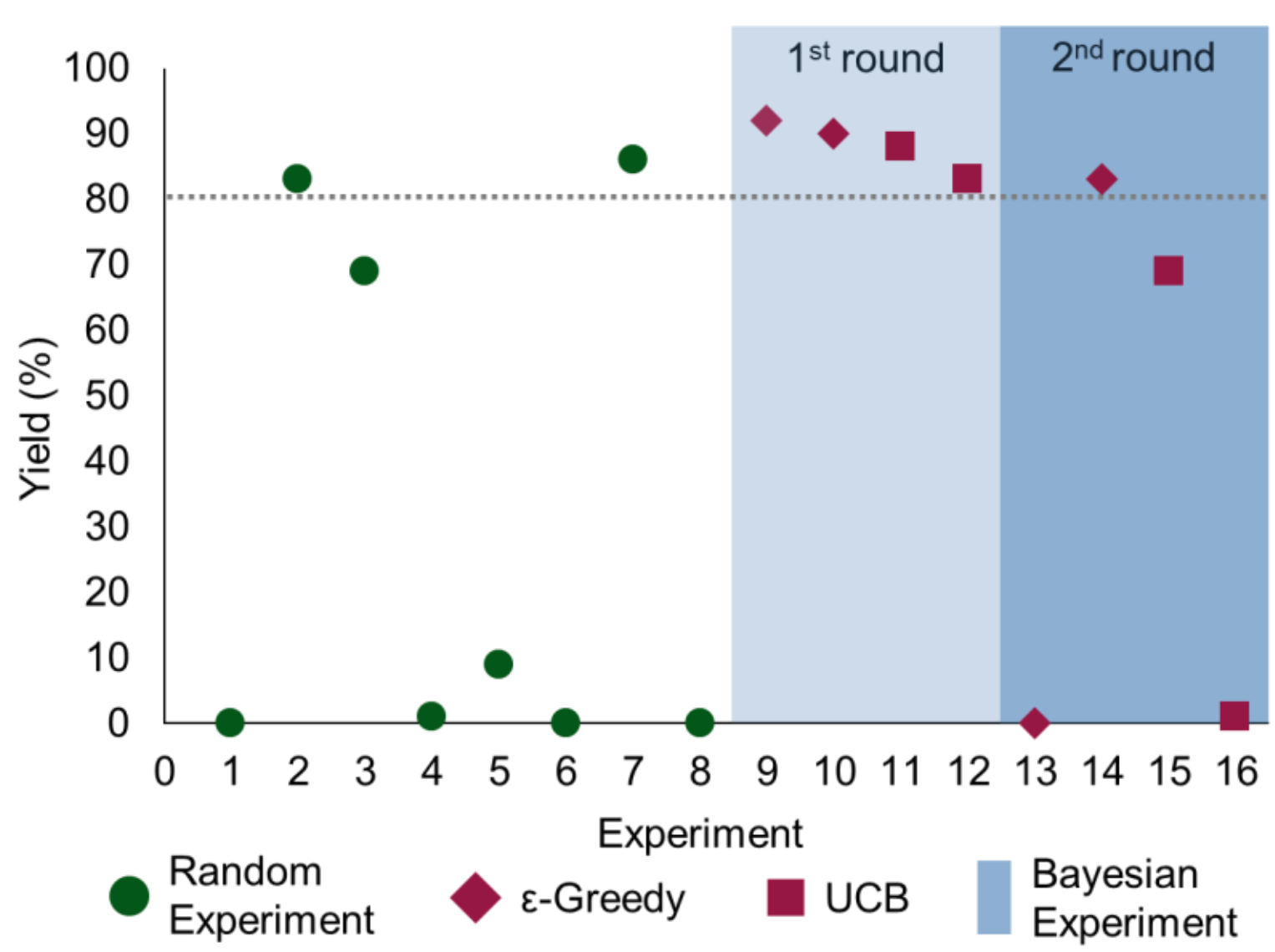

Figure S3

2d – n-Octyl 2-*O*-acetyl-6-*O*-(*tert*-butyldimethylsilyl)-α-ᴅ-galactopyranoside

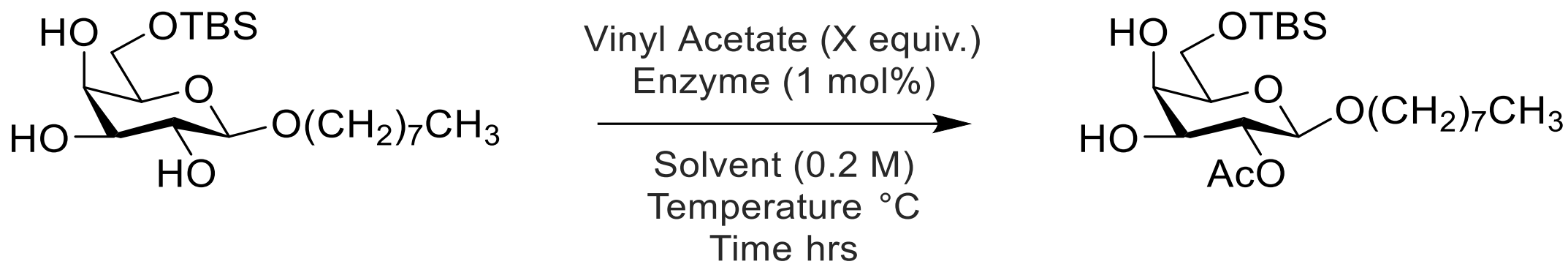

Round of Bayesian	Experiment Type	Enzyme	Solvent	Acyl equiv.	Time (hrs)	Temp (°C)	Yield (%)
Round 0	Random 1	PSL	MeCN	25	6	50	25
	Random 2	PPL	THF/Py (4:1)	45	12	45	0
	Random 3	PSL	MeCN	45	12	40	34
	Random 4	PFL	MeCN	45	12	40	59
	Random 5	PFL	THF/Py (4:1)	25	24	25	12
	Random 6	PSL	THF	50	24	50	6
	Random 7	PSL	THF	5	48	20	2
	Random 8	PFL	THF/Py (4:1)	40	72	25	41
Round 1	**Bayesian (ε-greedy) 1**	**PFL**	**MeCN**	**55**	**72**	**35**	**86**
	Bayesian (ε-greedy) 2	PFL	MeCN	55	72	30	85
	Bayesian (UCB) 1	PFL	Pyridine	55	72	70	5
	Bayesian (UCB) 2	PPL	MeCN	55	72	20	3

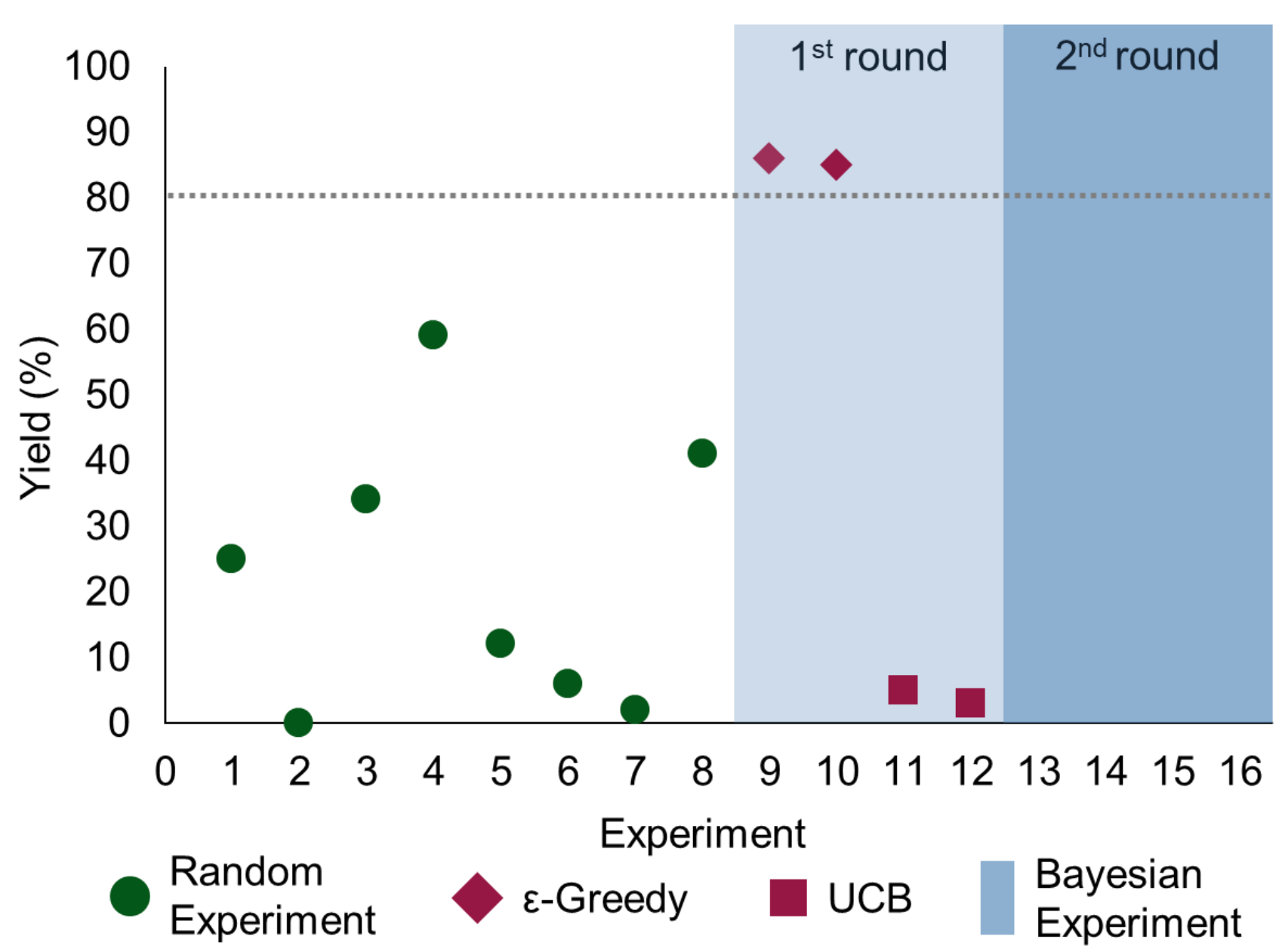

Figure S4

2e – Methyl 2-*O*-acetyl-6-*O*-(tert-butyldimethylsilyl)-α-ᴅ-mannopyranoside

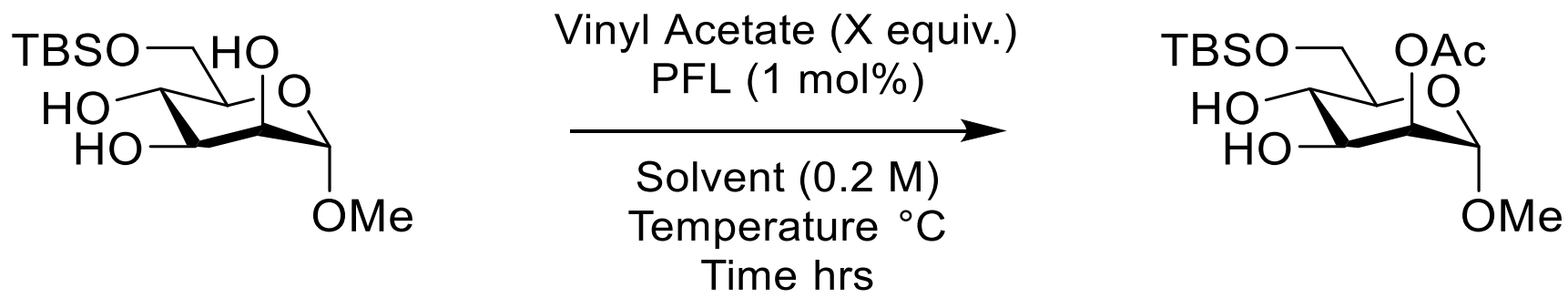

Round of Bayesian	Experiment Type	Enzyme	Solvent	Acyl equiv.	Time (hrs)	Temp (°C)	Yield (%)
Round 0	Random 1	PPL	THF	50	12	65	0
	Random 2	PPL	Pyridine	15	48	50	0
	Random 3	PFL	THF/Py (4:1)	30	12	50	0
	Random 4	PPL	THF	30	12	65	0
	Random 5	PSL	THF/Py (4:1)	55	6	35	0
	Random 6	PPL	THF/Py (4:1)	55	48	65	0
	Random 7	PFL	THF	15	72	35	6
	Random 8	PFL	THF	45	24	45	2
Round 1	Bayesian (ε-greedy) 1	PFL	THF	5	72	20	1
	Bayesian (ε-greedy) 2	PFL	THF	5	72	25	9
	Bayesian (UCB) 1	PSL	MeCN	5	72	20	0
	Bayesian (UCB) 2	PSL	THF	5	72	70	4
Round 2	Bayesian (ε-greedy) 1	PFL	THF	5	72	50	7
	Bayesian (ε-greedy) 2	PPL	THF	55	36	70	11
	Bayesian (UCB) 1	PFL	MeCN	55	72	70	14
	Bayesian (UCB) 2	PFL	Pyridine	55	72	20	5

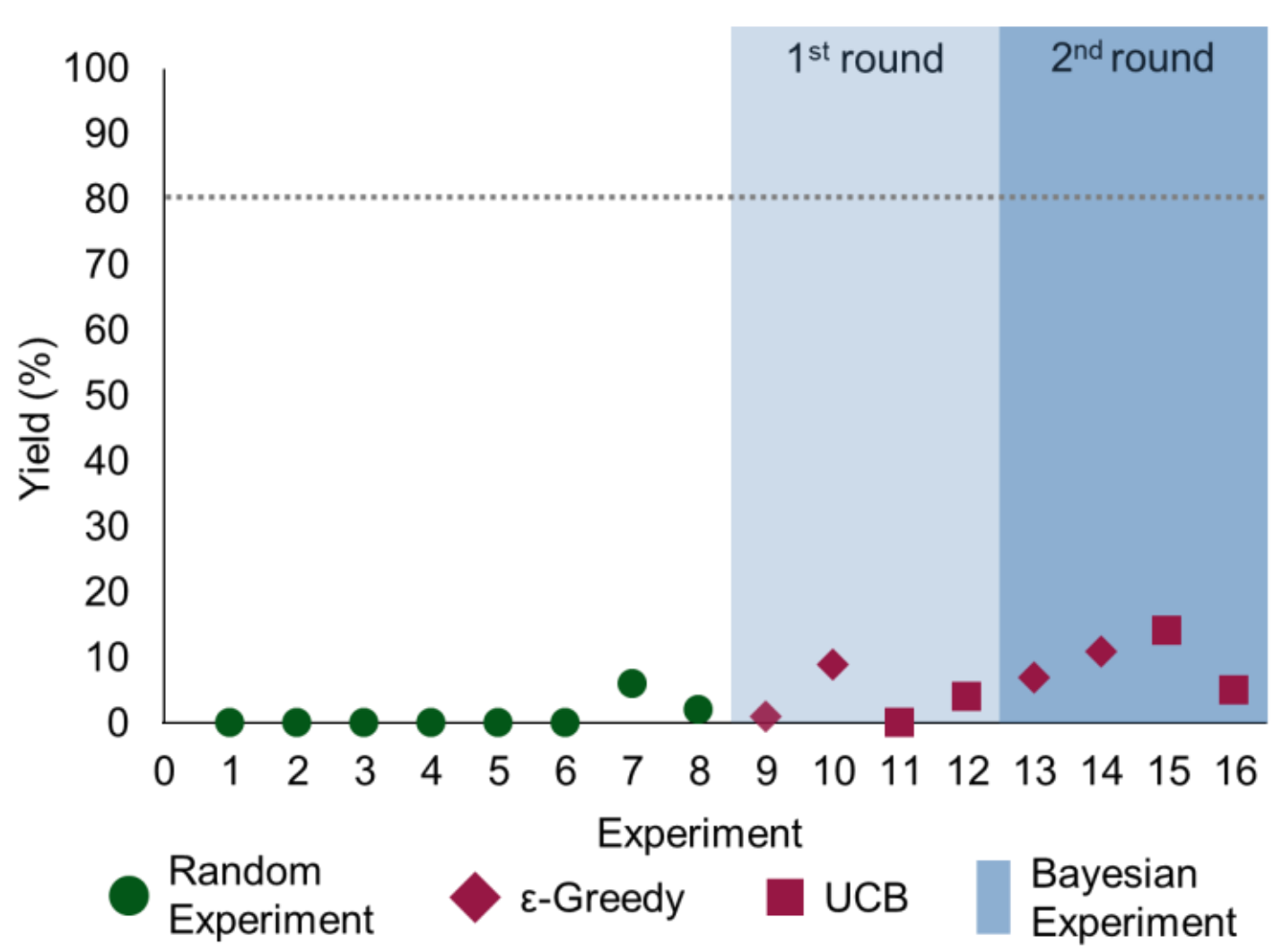

Figure S5

2f – 4-*O*-acetyl 1,6-β-D-anhydroglucose

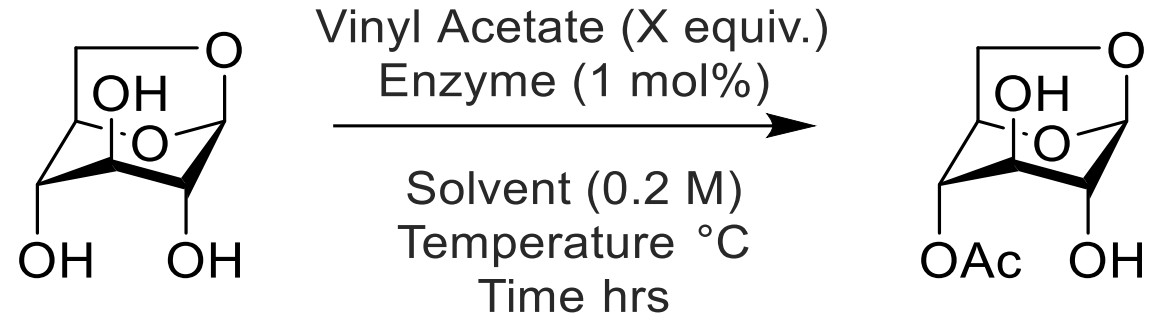

Round of Bayesian	Experiment Type	Enzyme	Solvent	Acyl equiv.	Time (hrs)	Temp (°C)	Yield (%)
Round 0	Random 1	PFL	THF/Py (4:1)	50	24	25	46
	Random 2	PPL	THF/Py (4:1)	20	24	30	53
	Random 3	PSL	THF/Py (4:1)	10	6	35	19
	Random 4	PFL	Pyridine	40	18	45	6
	Random 5	PFL	MeCN	45	6	50	52
	Random 6	PFL	THF	15	36	65	14
	Random 7	PFL	THF/Py (4:1)	50	36	65	20
	Random 8	PSL	THF/Py (4:1)	40	72	70	54
Round 1	Bayesian (ε-greedy) 1	PPL	MeCN	55	48	20	20
	Bayesian (ε-greedy) 2	PPL	MeCN	55	72	25	21
	Bayesian (UCB) 1	PPL	MeCN	55	72	20	25
	Bayesian (UCB) 2	PSL	THF	55	72	20	5
Round 2	Bayesian (ε-greedy) 1	PPL	THF/Py (4:1)	20	72	25	71
	**Bayesian (ε-greedy) 2**	**PPL**	**THF/Py (4:1)**	**25**	**72**	**35**	**87**
	Bayesian (UCB) 1	PPL	MeCN	5	6	70	11
	Bayesian (UCB) 2	PFL	THF/Py (4:1)	50	24	25	46

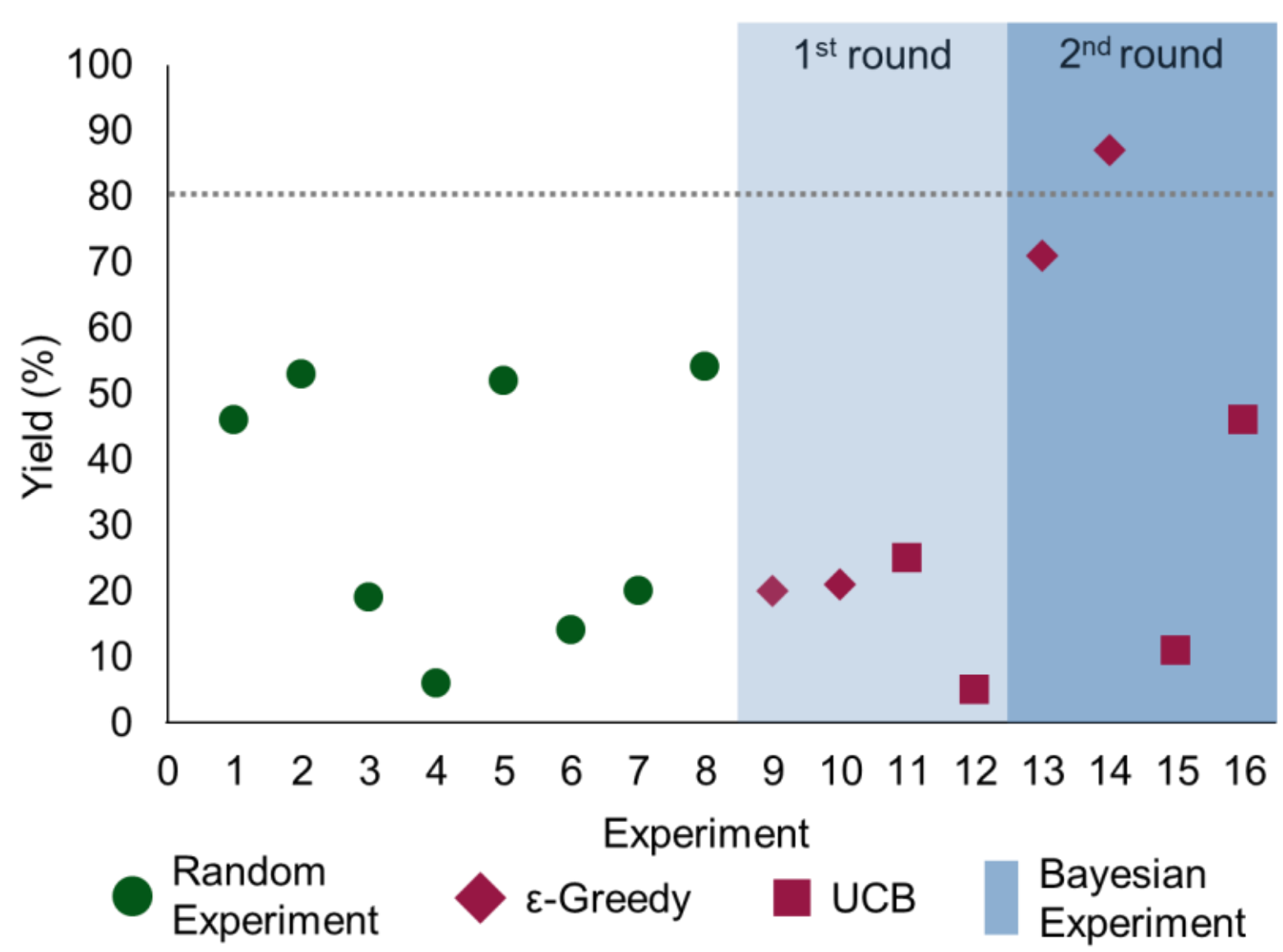

Figure S6

2f' – 2,4-di-*O*-acetyl 1,6-β-ᴅ-anhydroglucose

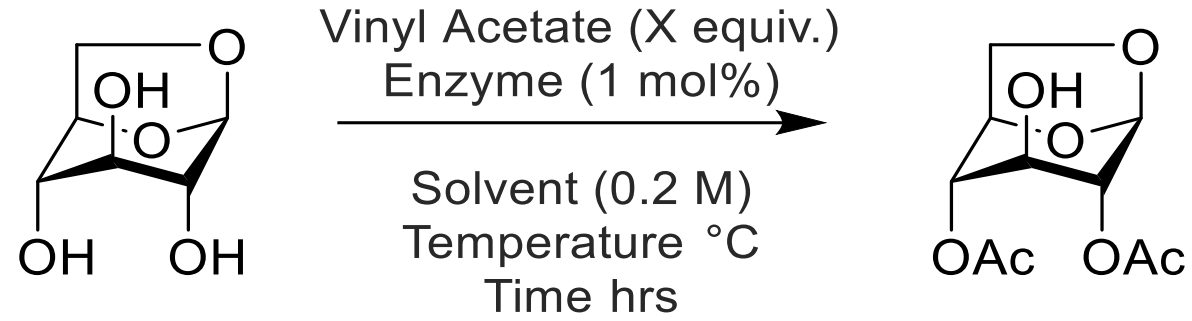

Round of Bayesian	Experiment Type	Enzyme	Solvent	Acyl equiv.	Time (hrs)	Temp (°C)	Yield (%)
Round 0	Random 1	PFL	THF/Py (4:1)	50	24	25	17
	Random 2	PPL	THF/Py (4:1)	20	24	30	5
	Random 3	PSL	THF/Py (4:1)	10	6	35	0
	Random 4	PFL	Pyridine	40	18	45	19
	Random 5	PFL	MeCN	45	6	50	1
	Random 6	PFL	THF	15	36	65	56
	Random 7	PFL	THF/Py (4:1)	50	36	65	45
	Random 8	PSL	THF/Py (4:1)	40	72	70	24
Round 1	Bayesian (ε-greedy) 1	PFL	THF	20	72	70	46
	Bayesian (ε-greedy) 2	PFL	THF	15	72	70	48
	Bayesian (UCB) 1	PPL	THF	55	72	70	10
	Bayesian (UCB) 2	PSL	THF	5	72	20	14
Round 2	Bayesian (ε-greedy) 1	PFL	THF	5	12	70	48
	**Bayesian (ε-greedy) 2**	**PFL**	**THF**	**5**	**18**	**70**	**55**
	Bayesian (UCB) 1	PSL	THF	55	6	70	0
	Bayesian (UCB) 2	PFL	THF/Py (4:1)	5	72	70	41

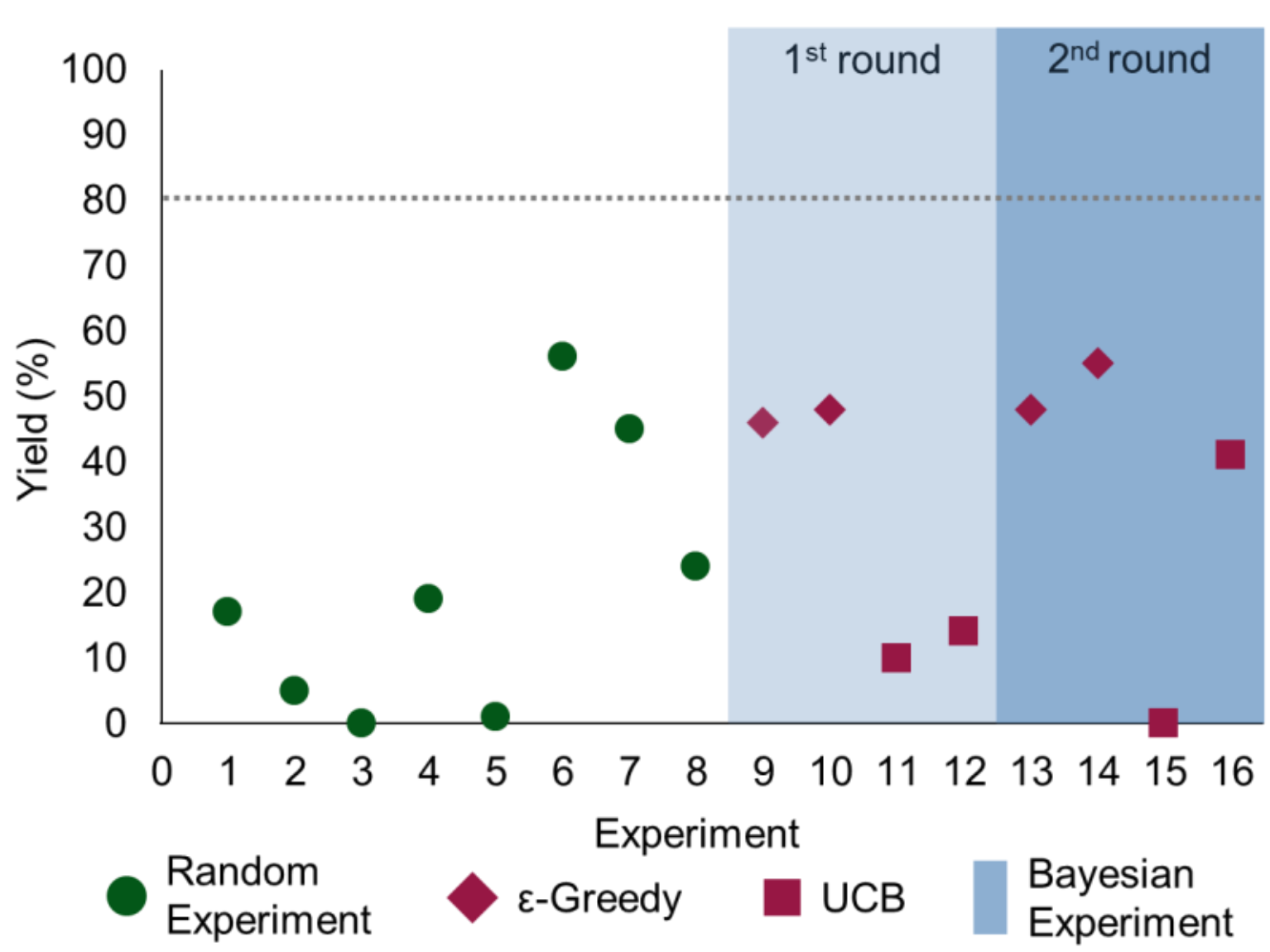

Figure S7

2g – Methyl 2-*O*-acetyl-4,6-benzylidene α-D-glucopyranoside

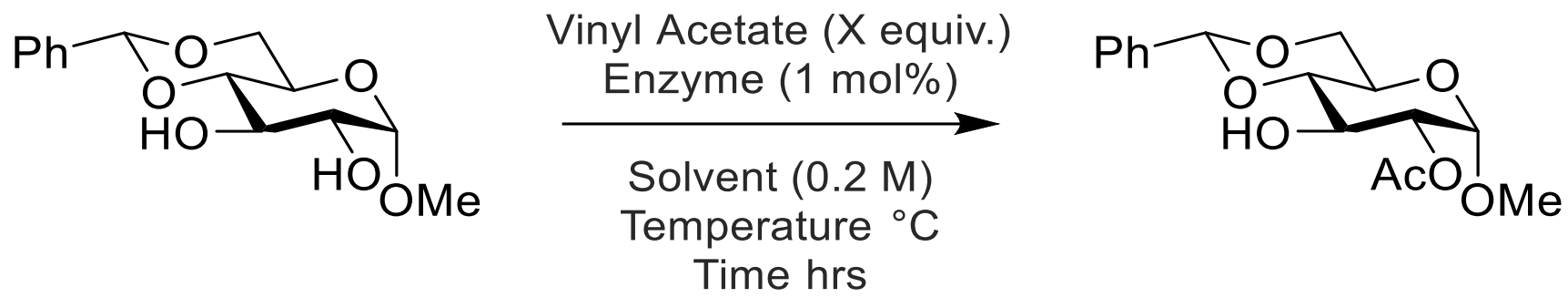

Round of Bayesian	Experiment Type	Enzyme	Solvent	Acyl equiv.	Time (hrs)	Temp (°C)	Yield (%)
Round 0	Random 1	PSL	Pyridine	55	36	20	0
	Random 2	PFL	THF	45	48	25	34
	Random 3	PPL	MeCN	35	18	35	3
	Random 4	PFL	THF/Py (4:1)	20	72	40	31
	Random 5	PFL	Pyridine	50	24	40	20
	Random 6	PFL	THF	5	6	50	7
	Random 7	PSL	THF/Py (4:1)	5	12	65	7
	Random 8	PSL	Pyridine	50	48	70	27
Round 1	Bayesian (ε-greedy) 1	PFL	THF	55	72	45	65
	Bayesian (ε-greedy) 2	PFL	THF	55	72	50	65
	Bayesian (UCB) 1	PPL	THF	55	72	70	9
	Bayesian (UCB) 2	PFL	MeCN	55	72	70	42
Round 2	**Bayesian (ε-greedy) 1**	**PFL**	**THF**	**55**	**72**	**70**	**84**
	Bayesian (ε-greedy) 2	PFL	THF	55	72	65	72
	Bayesian (UCB) 1	PSL	MeCN	5	72	70	0
	Bayesian (UCB) 2	PFL	Pyridine	5	72	70	0

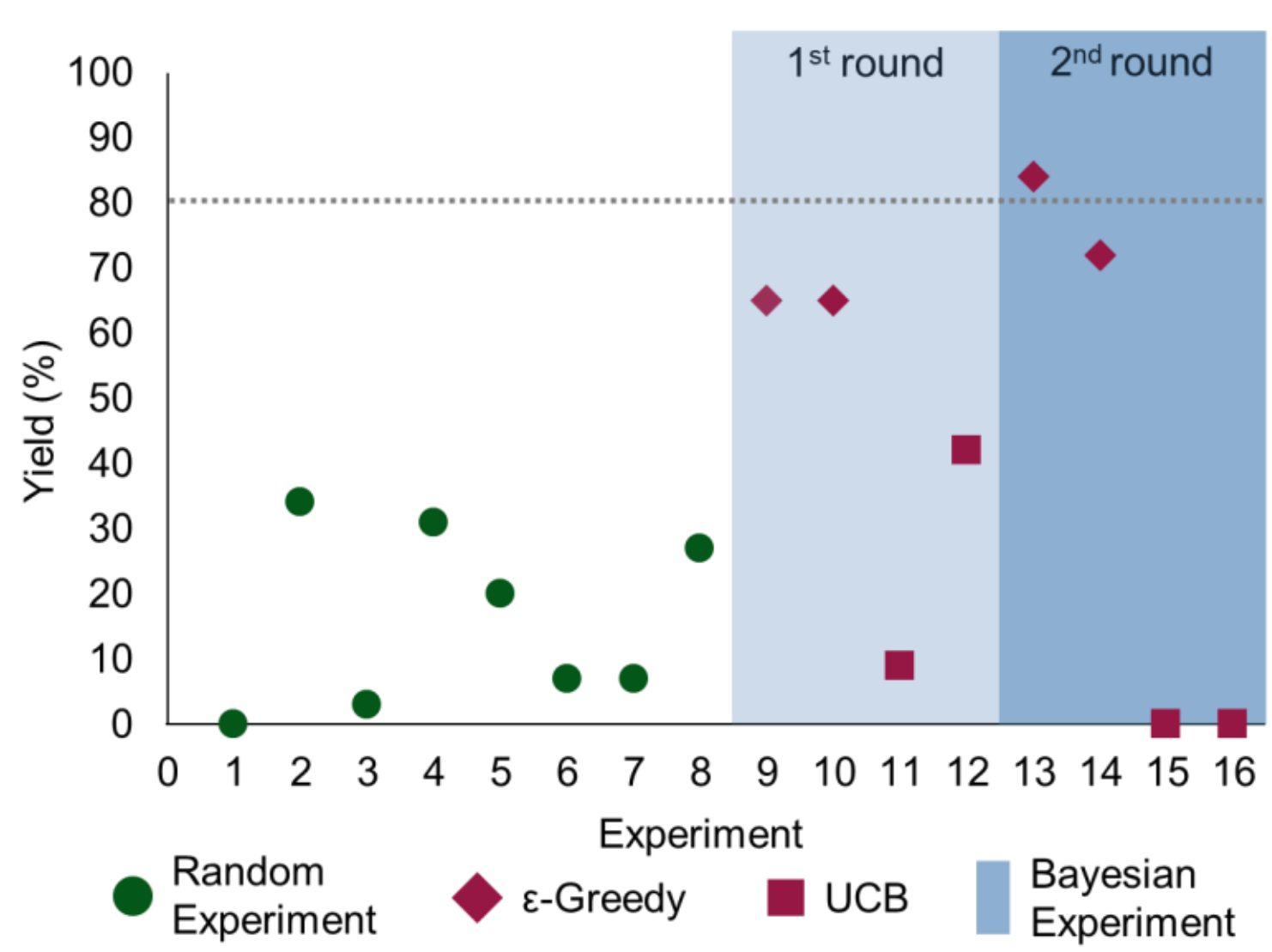

Figure S8

2h – methyl 2-*O*-acetyl-4,6-benzylidene α-ᴅ-galactopyranoside

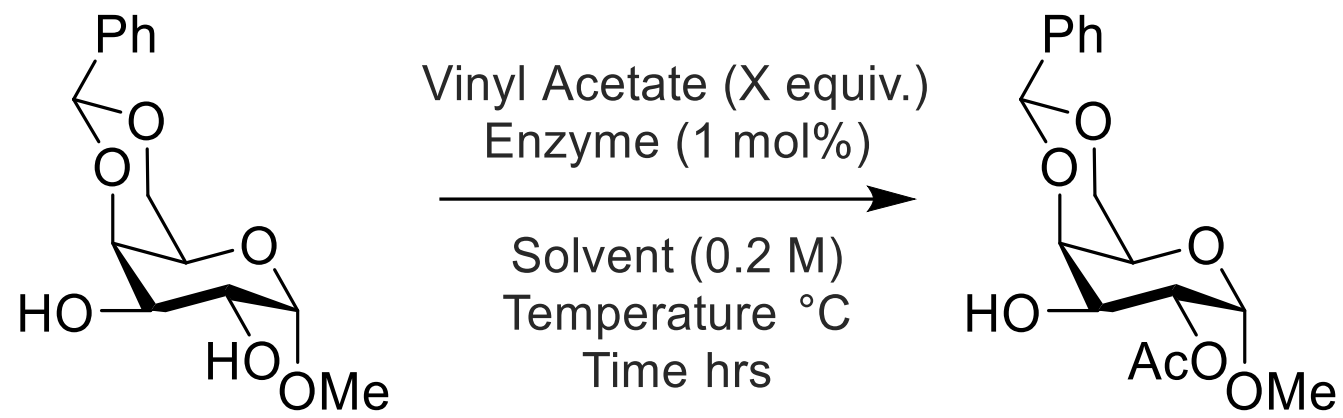

Round of Bayesian	Experiment Type	Enzyme	Solvent	Acyl equiv.	Time (hrs)	Temp (°C)	Yield (%)
Round 0	Random 1	PPL	Pyridine	35	36	20	0
	Random 2	PSL	MeCN	35	48	25	1
	Random 3	PSL	THF	20	36	30	1
	Random 4	PFL	MeCN	20	36	30	1
	Random 5	PPL	THF/Py (4:1)	25	24	35	0
	Random 6	PSL	Pyridine	25	36	45	0
	Random 7	PSL	MeCN	10	18	50	1
	Random 8	PPL	MeCN	25	24	60	0

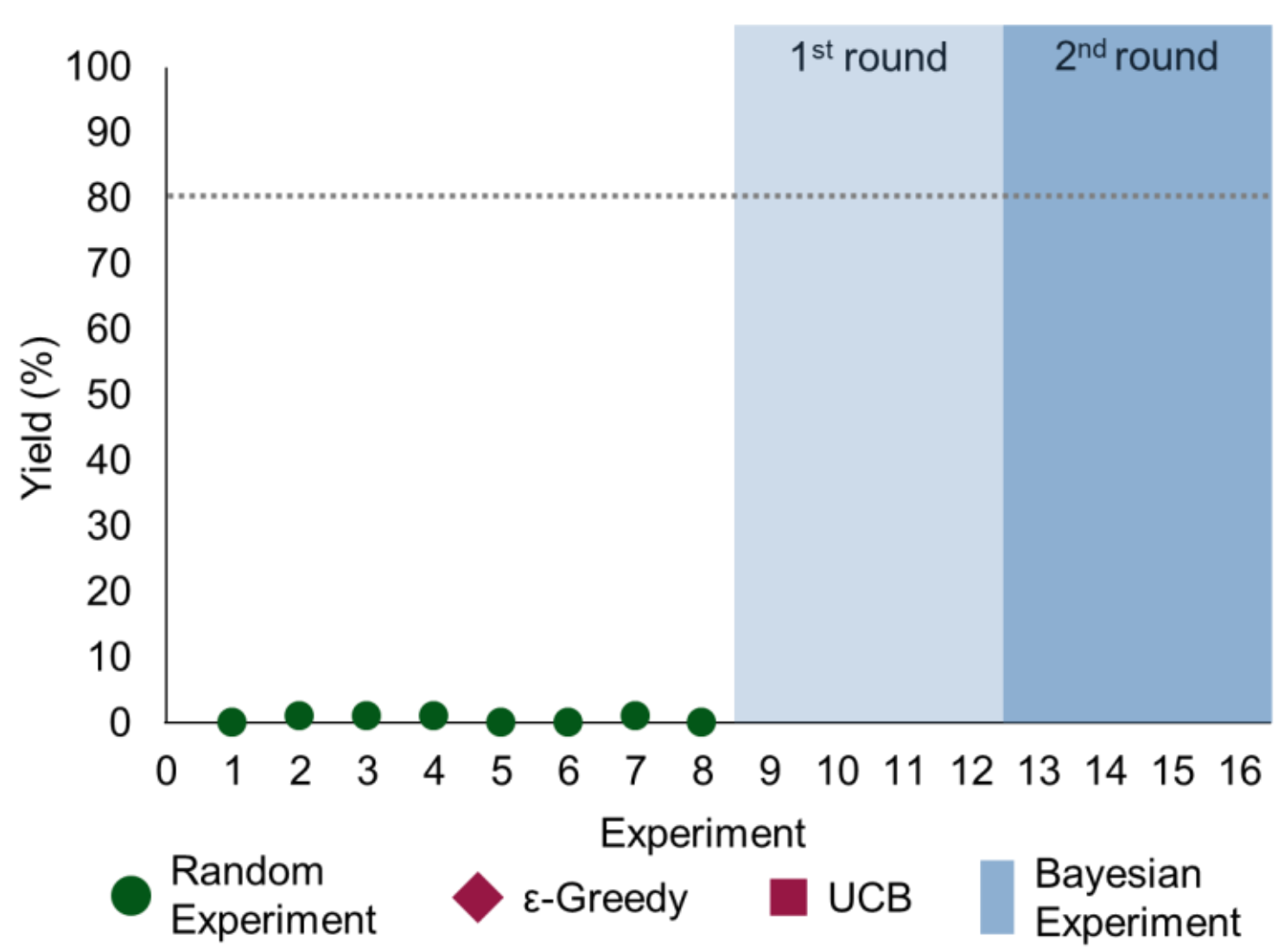

Figure S9

2i – Methyl 2-*O*-acetyl-α-D-xylopyranoside

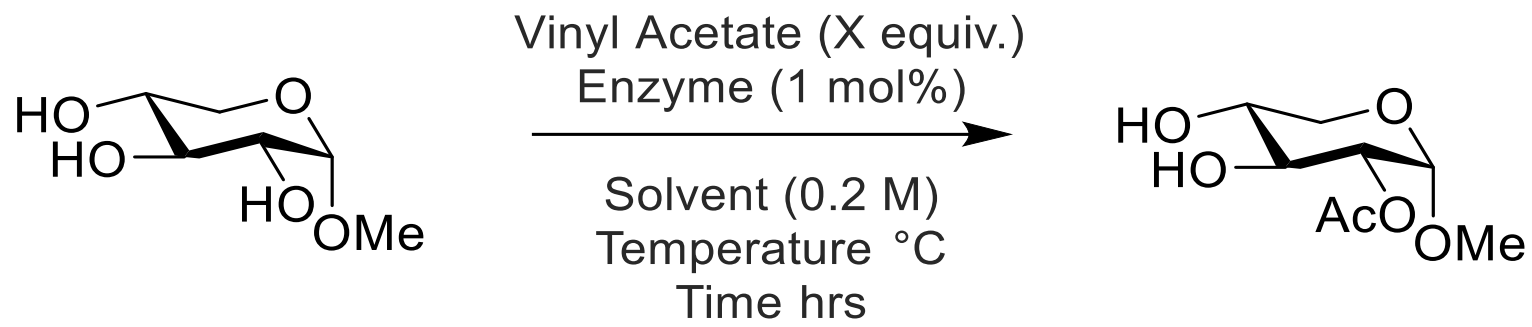

Round of Bayesian	Experiment Type	Enzyme	Solvent	Acyl equiv.	Time (hrs)	Temp (°C)	Yield (%)
Round 0	Random 1	PSL	THF	25	12	30	29
	Random 2	PFL	THF	10	6	35	43
	Random 3	PSL	MeCN	55	6	40	0
	Random 4	PPL	THF/Py (4:1)	35	12	45	27
	Random 5	PSL	MeCN	20	72	60	55
	Random 6	PPL	THF/Py (4:1)	50	18	65	16
	Random 7	PFL	Pyridine	35	36	65	31
	Random 8	PFL	MeCN	10	18	70	19
Round 1	Bayesian (ε-greedy) 1	PSL	MeCN	15	72	50	30
	Bayesian (ε-greedy) 2	PSL	MeCN	5	72	55	32
	Bayesian (UCB) 1	PPL	THF	5	72	20	56
	Bayesian (UCB) 2	PFL	THF/Py (4:1)	5	72	20	82
Round 2	Bayesian (ε-greedy) 1	PFL	THF/Py (4:1)	10	72	20	70
	**Bayesian (ε-greedy) 2**	**PFL**	**THF/Py (4:1)**	**5**	**72**	**25**	**92**
	Bayesian (UCB) 1	PFL	THF	55	72	70	74
	Bayesian (UCB) 2	PSL	THF/Py (4:1)	55	72	20	27

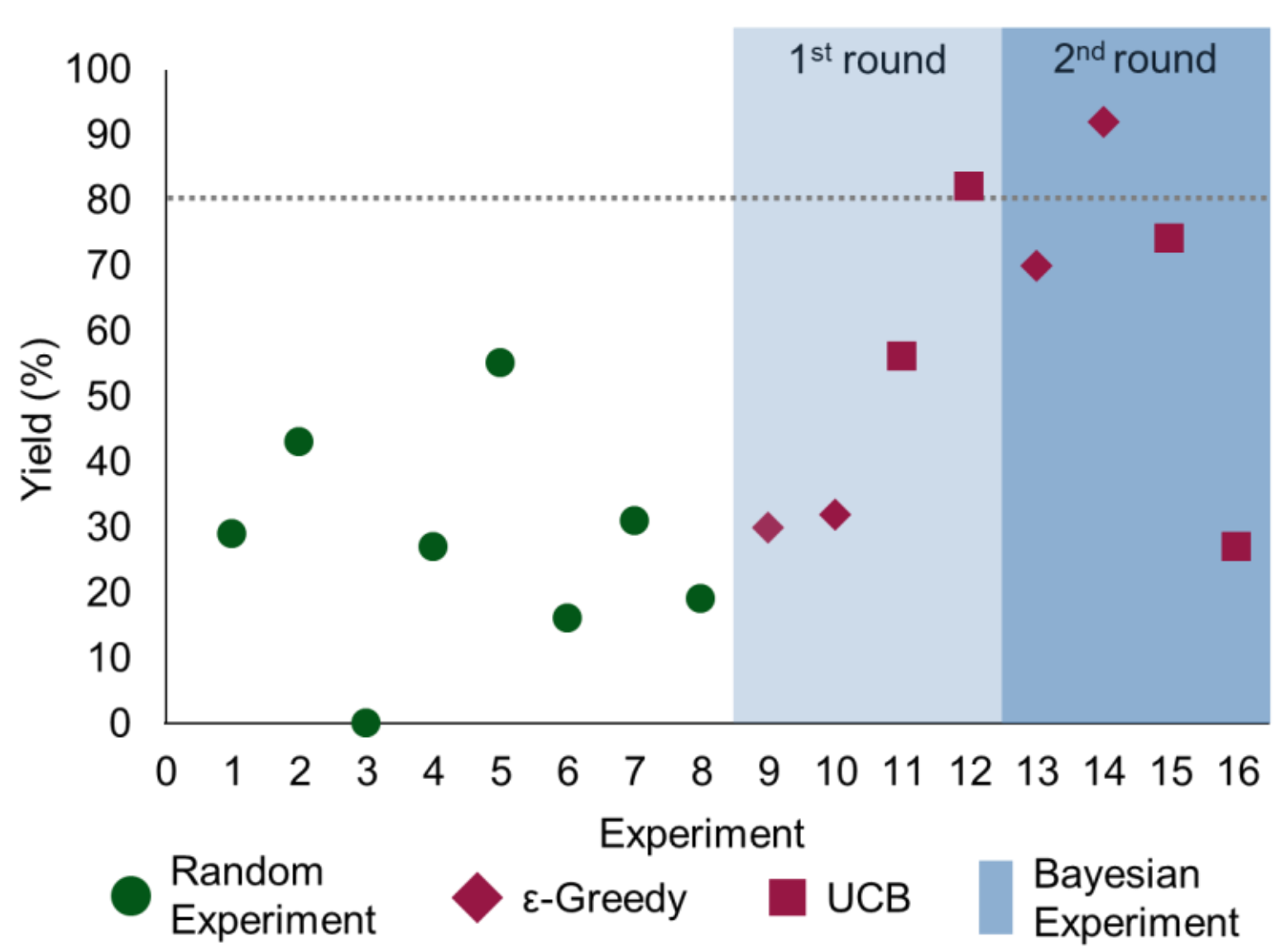

Figure S10

2j – Methyl 4-*O*-acetyl-β-L-fucopyranoside

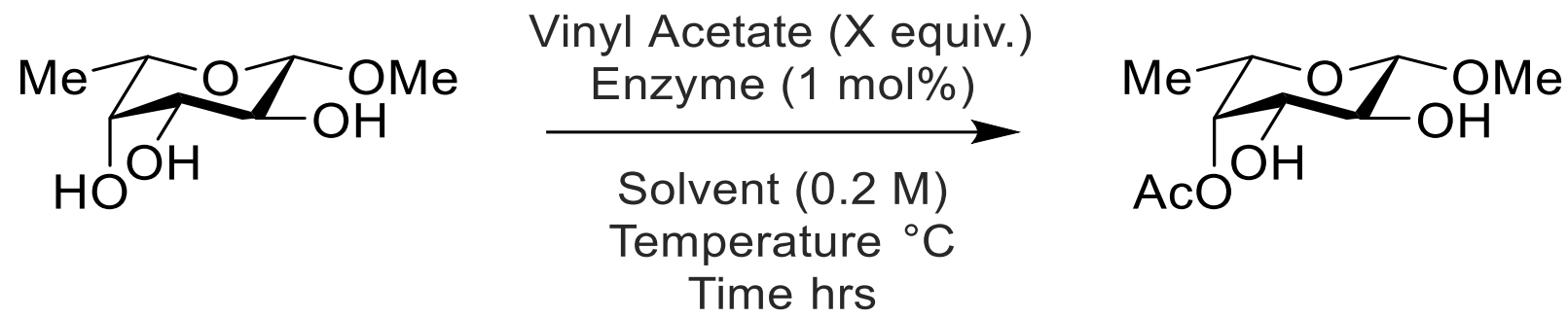

Round of Bayesian	Experiment Type	Enzyme	Solvent	Acyl equiv.	Time (hrs)	Temp (°C)	Yield (%)
Round 0	Random 1	PPL	Pyridine	40	6	25	1
	Random 2	PFL	THF/Py (4:1)	10	36	25	80
	Random 3	PSL	THF	55	18	30	2
	Random 4	PPL	Pyridine	10	36	40	9
	Random 5	PPL	MeCN	20	24	65	2
	Random 6	PFL	THF	35	36	65	58
	Random 7	PSL	Pyridine	55	48	65	12
	Random 8	PPL	Pyridine	35	18	70	16
Round 1	**Bayesian (ε-greedy) 1**	**PFL**	**THF/Py (4:1)**	**5**	**48**	**40**	**96**
	Bayesian (ε-greedy) 2	PFL	THF/Py (4:1)	5	48	45	91
	Bayesian (UCB) 1	PFL	MeCN	55	72	20	52
	Bayesian (UCB) 2	PFL	THF/Py (4:1)	55	72	70	73

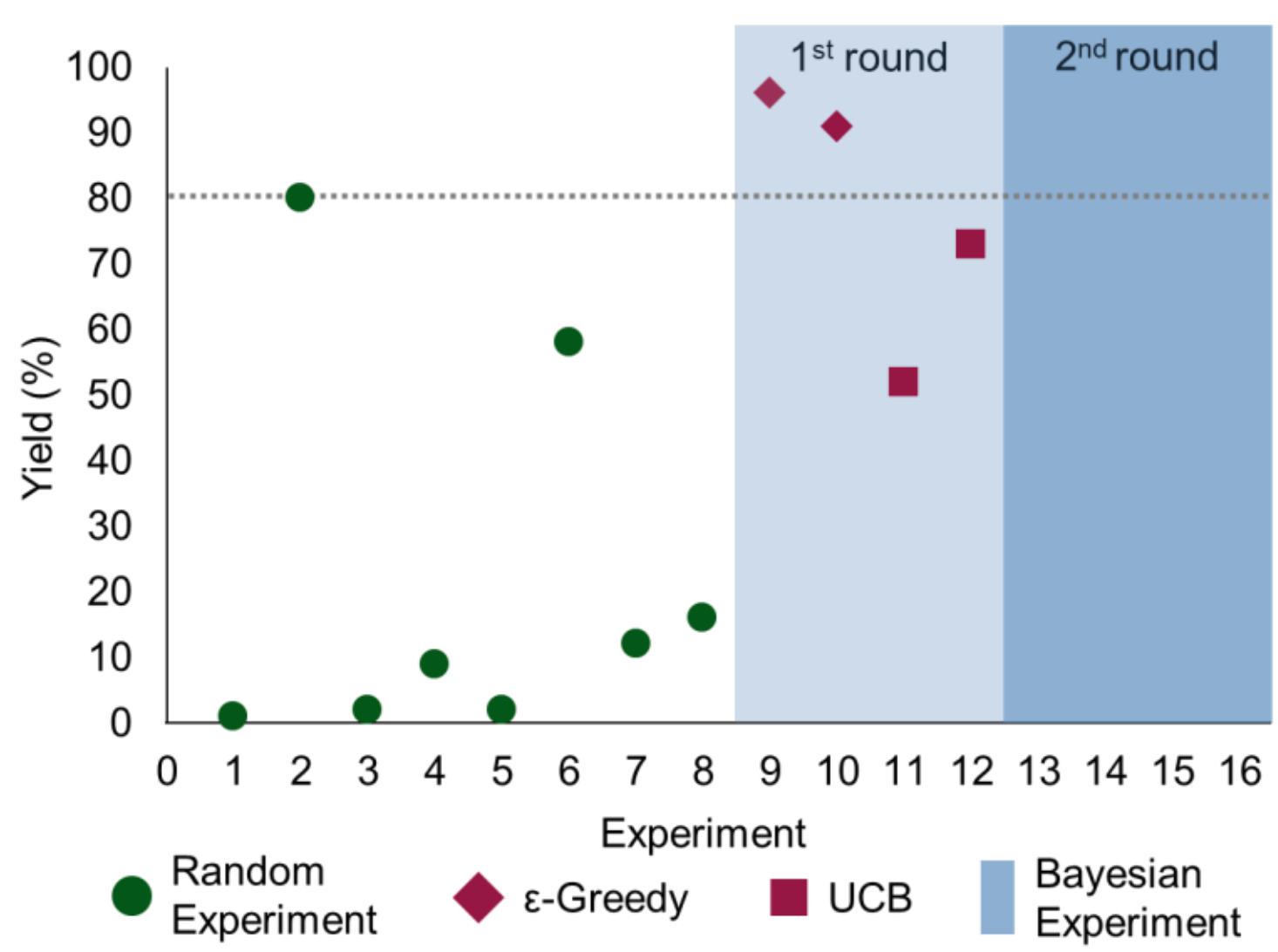

Figure S11

2k – Methyl 2-*O*-acetyl-β-L-arabinopyranoside

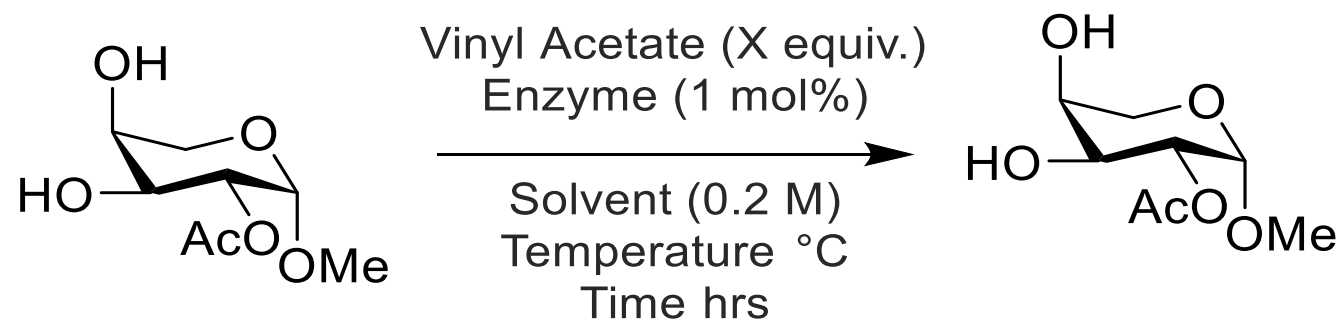

Round of Bayesian	Experiment Type	Enzyme	Solvent	Acyl equiv.	Time (hrs)	Temp (°C)	Yield (%)
Round 0	Random 1	PFL	Pyridine	40	24	30	42
	Random 2	PSL	THF	10	72	30	89
	Random 3	PSL	MeCN	20	36	40	7
	Random 4	PPL	MeCN	30	18	40	2
	Random 5	PPL	Pyridine	50	24	50	8
	Random 6	PFL	THF/Py (4:1)	10	36	65	84
	Random 7	PFL	THF	30	18	65	82
	**Random 8**	**PFL**	**MeCN**	**25**	**36**	**70**	**91**
Round 1	Bayesian (ε-greedy) 1	PFL	THF	5	72	70	77
	Bayesian (ε-greedy) 2	PFL	THF	5	72	65	88
	Bayesian (UCB) 1	PSL	THF/Py (4:1)	55	72	70	88
	Bayesian (UCB) 2	PFL	THF	55	72	20	54

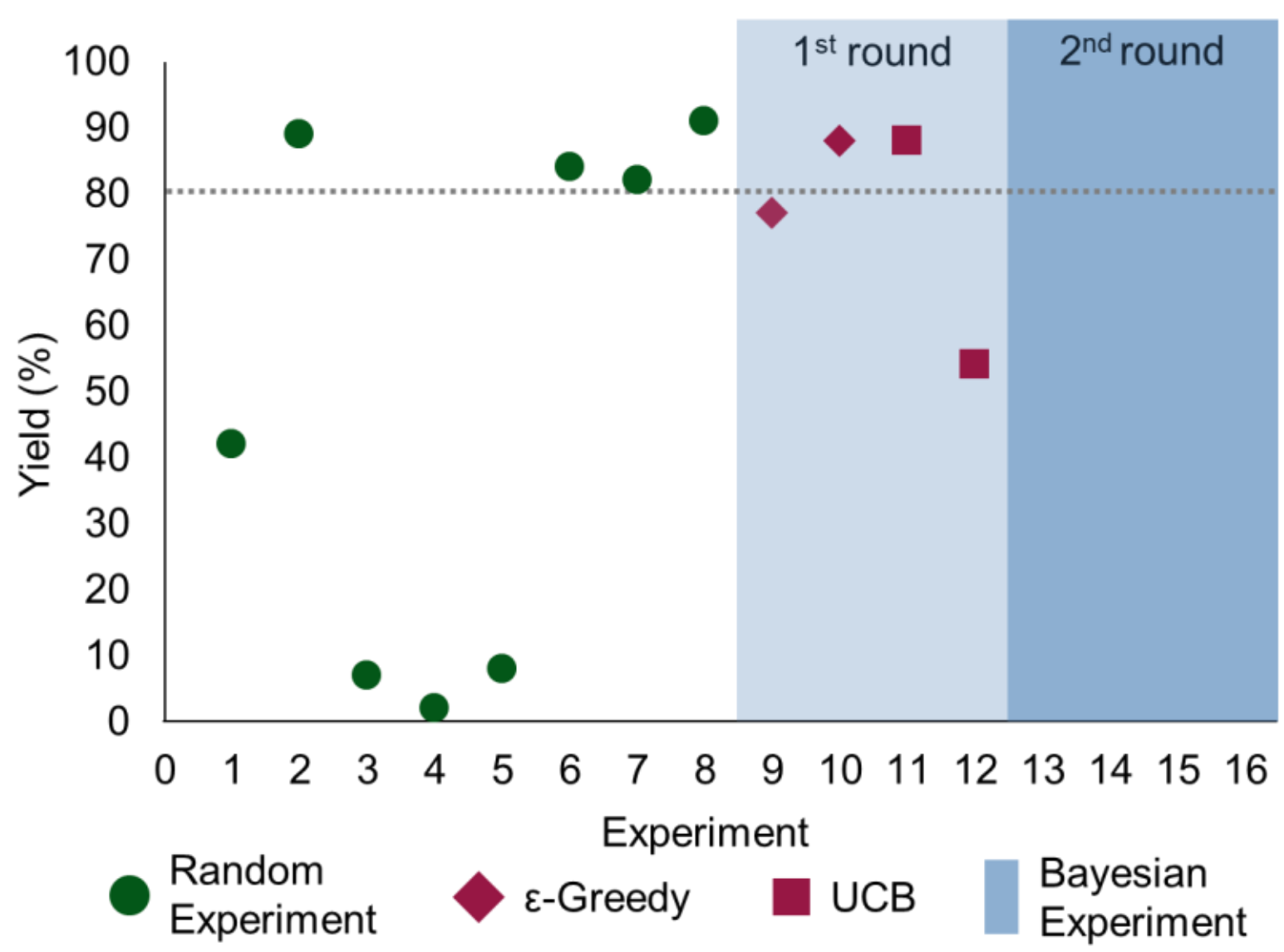

Figure S12

2l – Methyl 4-*O*-acetyl-β-ᴅ-arabinopyranoside

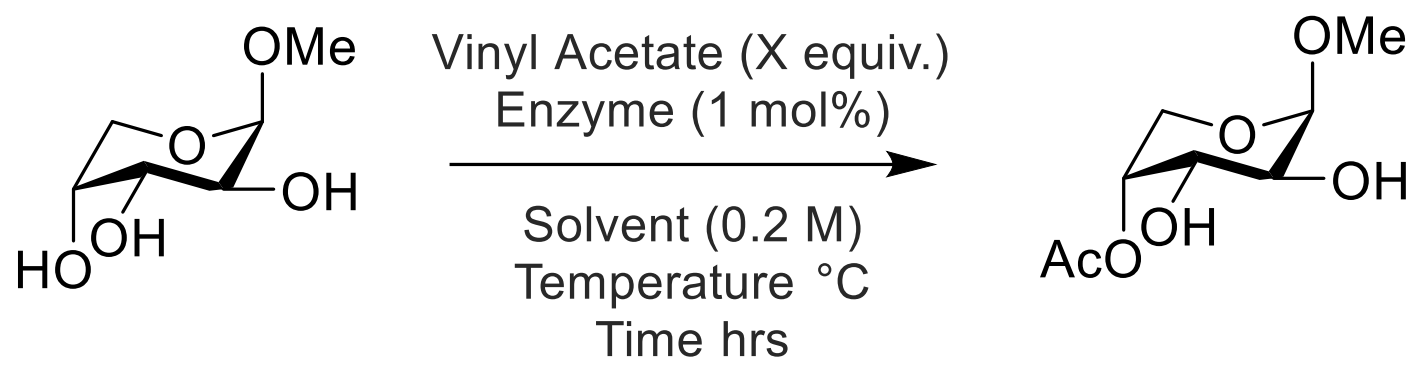

Round of Bayesian	Experiment Type	Enzyme	Solvent	Acyl equiv.	Time (hrs)	Temp (°C)	Yield (%)
Round 0	Random 1	PPL	Pyridine	25	48	20	51
	Random 2	PSL	Pyridine	35	48	25	5
	Random 3	PPL	MeCN	50	48	25	8
	Random 4	PPL	THF/Py (4:1)	30	6	50	32
	Random 5	PFL	THF/Py (4:1)	15	36	55	80
	Random 6	PSL	MeCN	35	18	60	48
	Random 7	PSL	THF	30	72	70	23
	Random 8	PPL	THF	10	72	70	18
Round 1	Bayesian (ε-greedy) 1	PFL	THF/Py (4:1)	5	24	65	85
	**Bayesian (ε-greedy) 2**	**PFL**	**THF/Py (4:1)**	**5**	**18**	**65**	**96**
	Bayesian (UCB) 1	PFL	Pyridine	55	6	70	88
	Bayesian (UCB) 2	PFL	THF	5	6	20	0

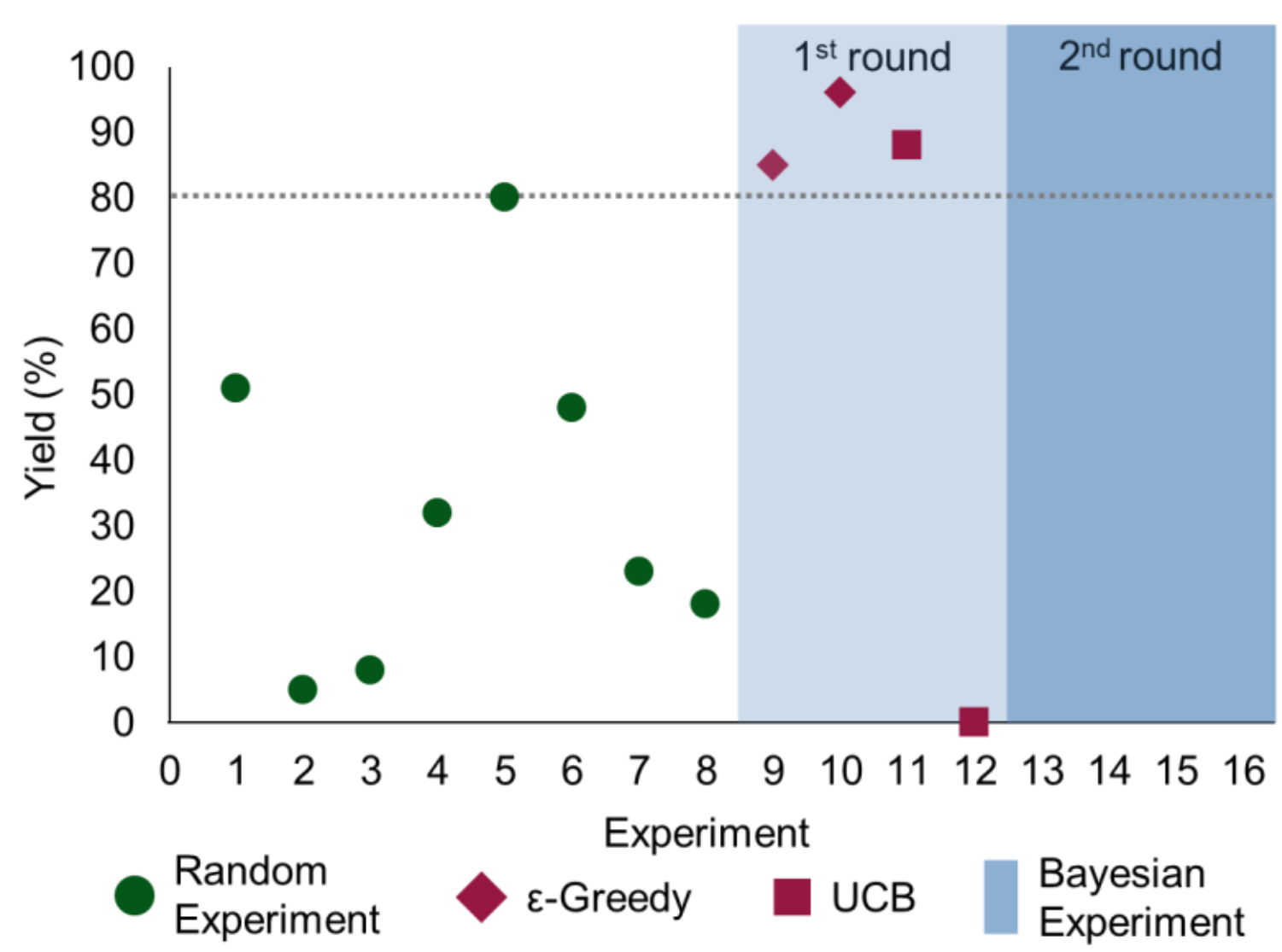

Figure S13

2m – Methyl 4-*O*-acetyl-α-L-rhamnopyranoside

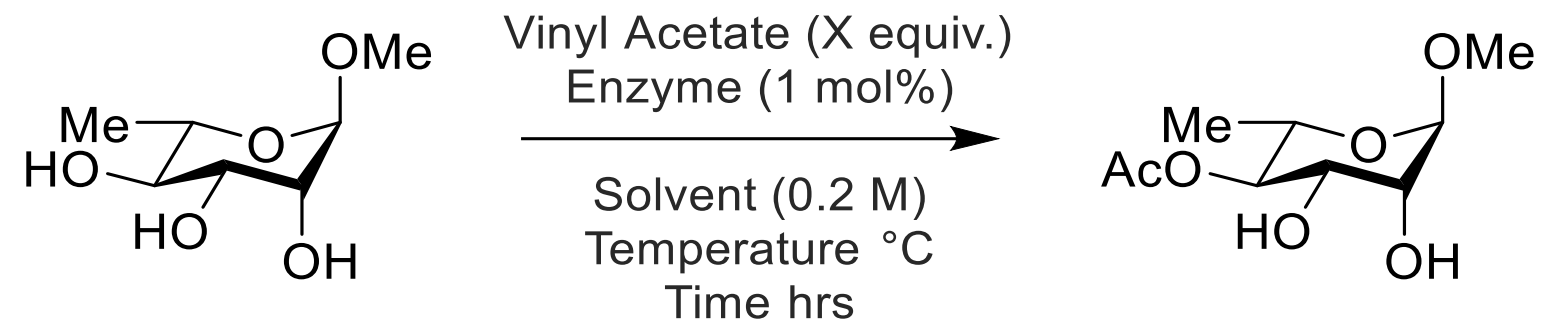

Round of Bayesian	Experiment Type	Enzyme	Solvent	Acyl equiv.	Time (hrs)	Temp (°C)	Yield (%)
Round 0	Random 1	PSL	THF	35	6	25	0
	Random 2	PPL	MeCN	30	24	30	0
	Random 3	PFL	MeCN	55	24	50	64
	Random 4	PPL	THF	50	48	55	8
	Random 5	PPL	Pyridine	25	72	55	0
	Random 6	PPL	MeCN	40	36	60	6
	Random 7	PSL	Pyridine	5	36	60	0
	Random 8	PSL	THF/Py (4:1)	50	72	70	80
Round 1	Bayesian (ε-greedy) 1	PFL	THF/Py (4:1)	55	72	70	71
	**Bayesian (ε-greedy) 2**	**PSL**	**THF/Py (4:1)**	**55**	**72**	**70**	**79**
	Bayesian (UCB) 1	PFL	THF/Py (4:1)	5	72	20	42
	Bayesian (UCB) 2	PFL	THF/Py (4:1)	55	6	70	47
Round 2	Bayesian (ε-greedy) 1	PSL	THF/Py (4:1)	55	72	65	68
	Bayesian (ε-greedy) 2	PSL	THF/Py (4:1)	55	72	60	70
	Bayesian (UCB) 1	PFL	THF	5	72	70	62
	Bayesian (UCB) 2	PFL	Pyridine	55	72	20	23

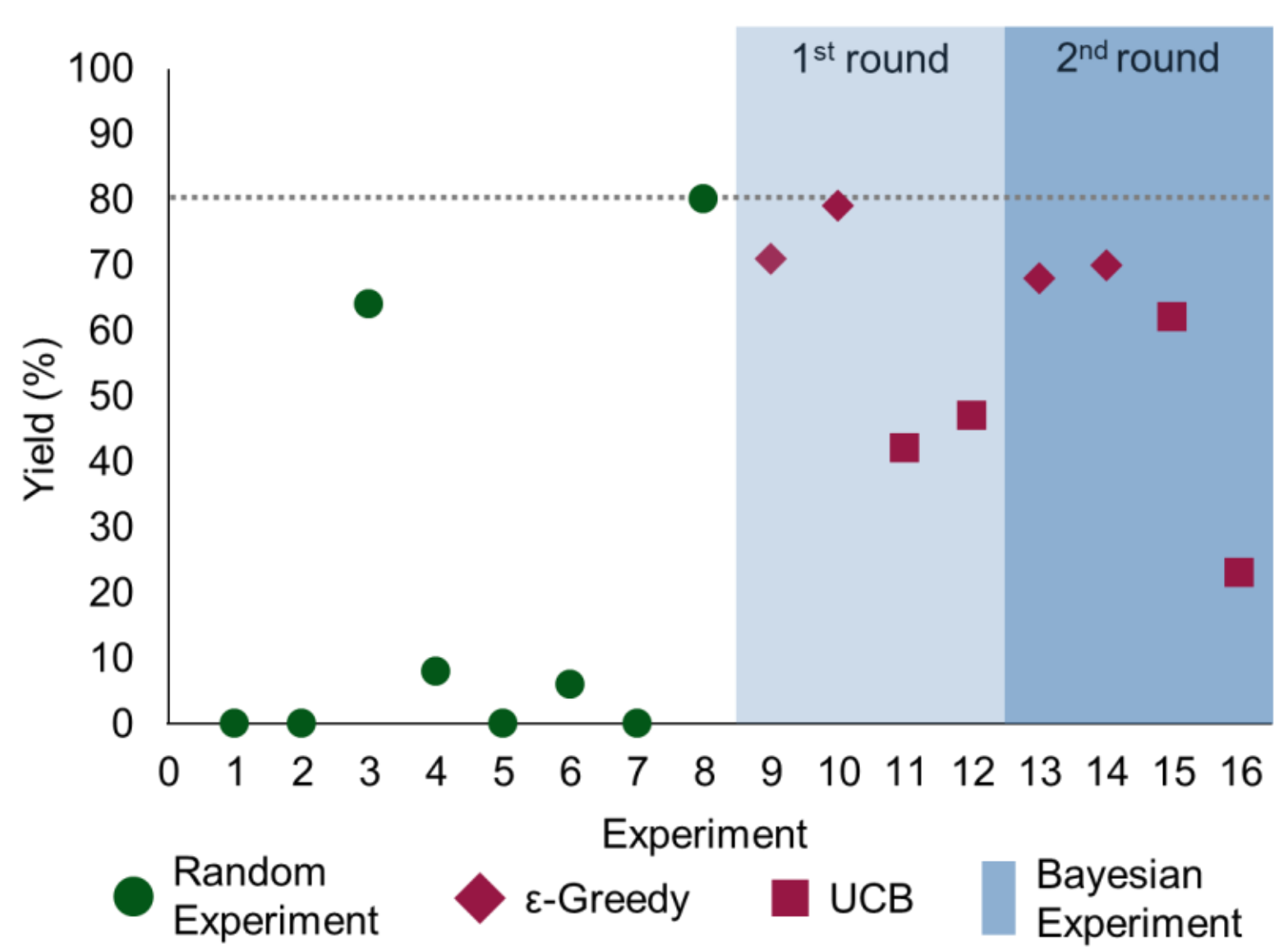

Figure S14

2n – ethyl 1-thio-4-*O*-acetyl-α-L-rhamnopyranoside

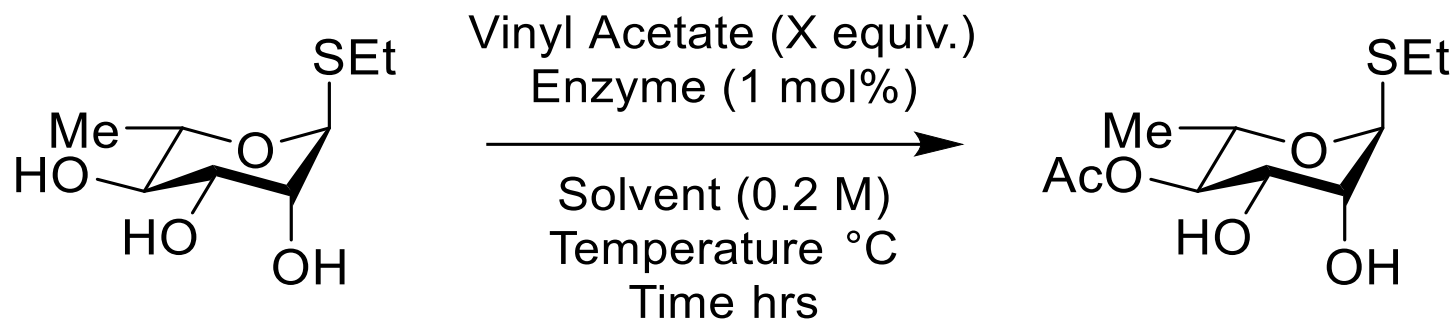

Round of Bayesian	Experiment Type	Enzyme	Solvent	Acyl equiv.	Time (hrs)	Temp (°C)	Yield (%)
Round 0	Random 1	PPL	THF/Py (4:1)	20	18	20	0
	Random 2	PFL	THF/Py (4:1)	20	36	20	9
	Random 3	PPL	Pyridine	15	12	30	0
	Random 4	PPL	Pyridine	50	72	70	4
	Random 5	PSL	THF	50	36	35	7
	Random 6	PSL	THF/Py (4:1)	30	36	35	8
	Random 7	PSL	THF/Py (4:1)	55	12	35	4
	Random 8	PPL	MeCN	20	6	55	1
Round 1	Bayesian (ε-greedy) 1	PFL	THF/Py (4:1)	20	72	40	4
	Bayesian (ε-greedy) 2	PFL	THF/Py (4:1)	20	72	45	11
	Bayesian (UCB) 1	PFL	THF	5	72	70	28
	Bayesian (UCB) 2	PFL	MeCN	55	72	20	16
Round 2	**Bayesian (ε-greedy) 1**	**PFL**	**THF**	**10**	**72**	**70**	**51**
	Bayesian (ε-greedy) 2	PPL	Pyridine	15	18	55	1
	Bayesian (UCB) 1	PFL	THF	55	6	70	25
	Bayesian (UCB) 2	PFL	Pyridine	5	6	70	9

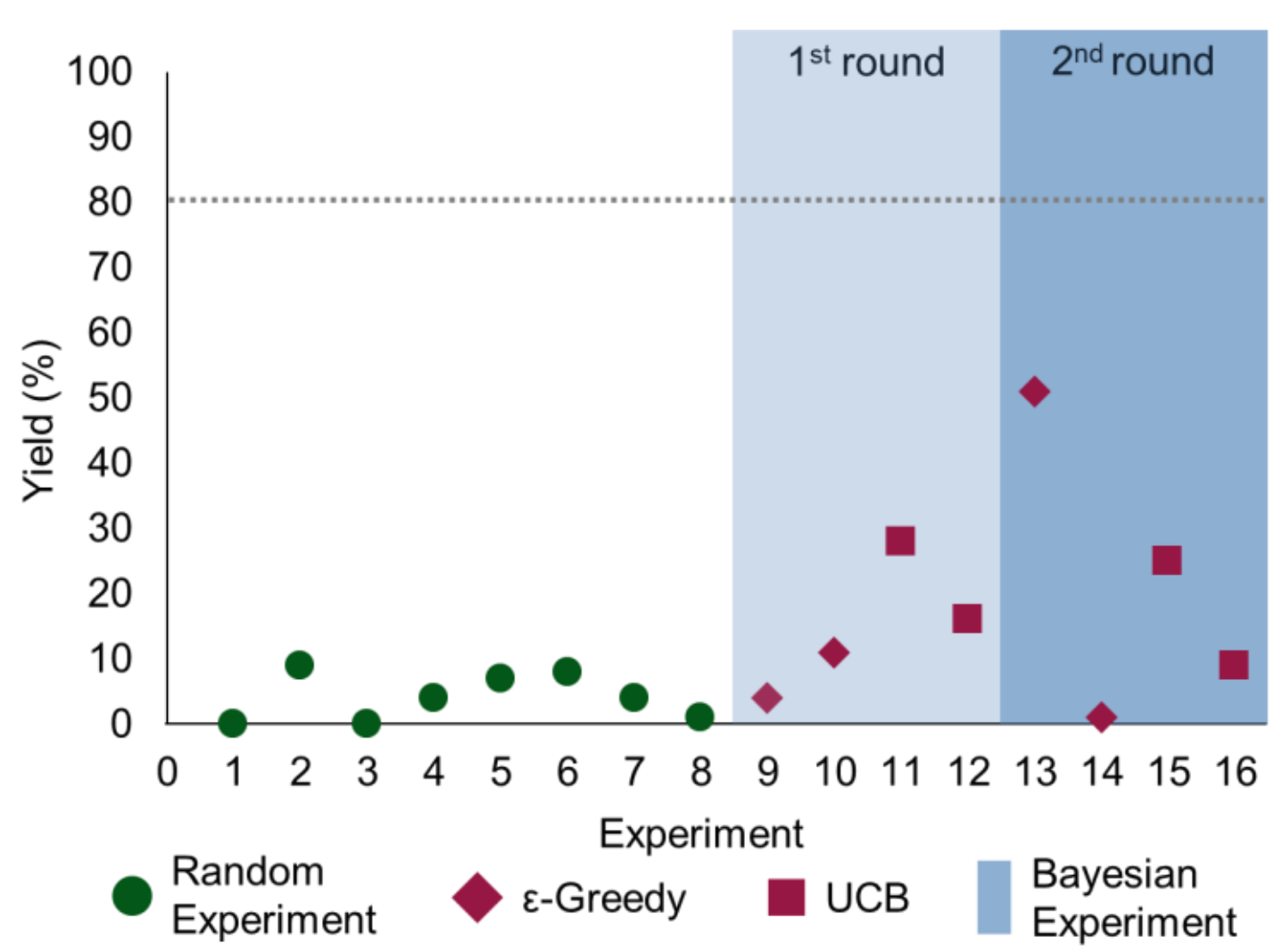

Figure S15

2o – Methyl 6-*O*-acetyl-α-ᴅ-glucopyranoside

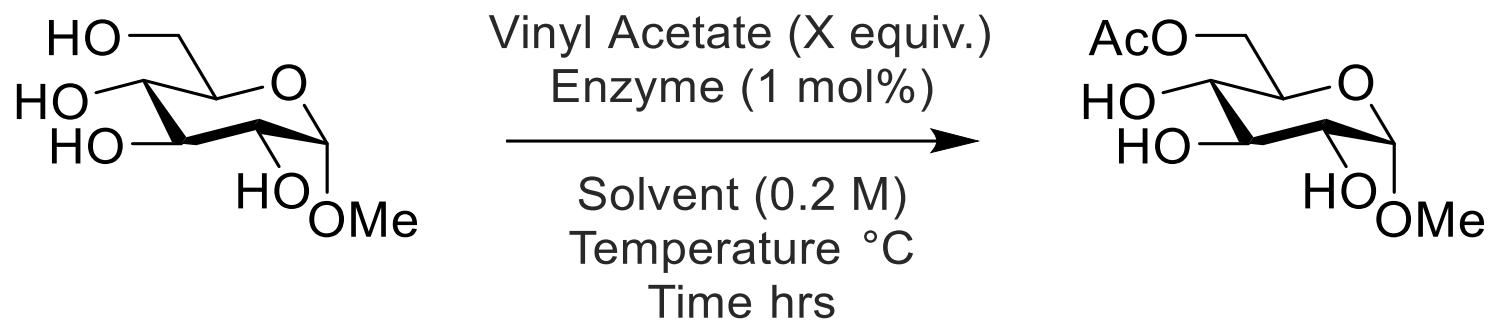

Round of Bayesian	Experiment Type	Enzyme	Solvent	Acyl equiv.	Time (hrs)	Temp (°C)	Yield (%)
Round 0	Random 1	PFL	THF/Py (4:1)	5	18	60	6
	Random 2	PPL	THF/Py (4:1)	35	6	20	20
	Random 3	PPL	THF	10	6	50	46
	Random 4	PSL	THF	5	48	70	31
	Random 5	PSL	THF/Py (4:1)	35	12	65	58
	Random 6	PPL	Pyridine	40	12	60	64
	Random 7	PSL	MeCN	25	24	20	14
	Random 8	PPL	THF/Py (4:1)	25	18	50	54
Round 1	Bayesian (ε-greedy) 1	PPL	Pyridine	50	12	70	61
	**Bayesian (ε-greedy) 2**	**PPL**	**Pyridine**	**45**	**12**	**70**	**70**
	Bayesian (UCB) 1	PFL	Pyridine	55	72	20	0
	Bayesian (UCB) 2	PPL	MeCN	55	72	70	17
Round 2	Bayesian (ε-greedy) 1	PPL	Pyridine	15	6	70	57
	Bayesian (ε-greedy) 2	PPL	Pyridine	10	6	70	43
	Bayesian (UCB) 1	PPL	Pyridine	5	72	70	42
	Bayesian (UCB) 2	PSL	Pyridine	5	6	70	11

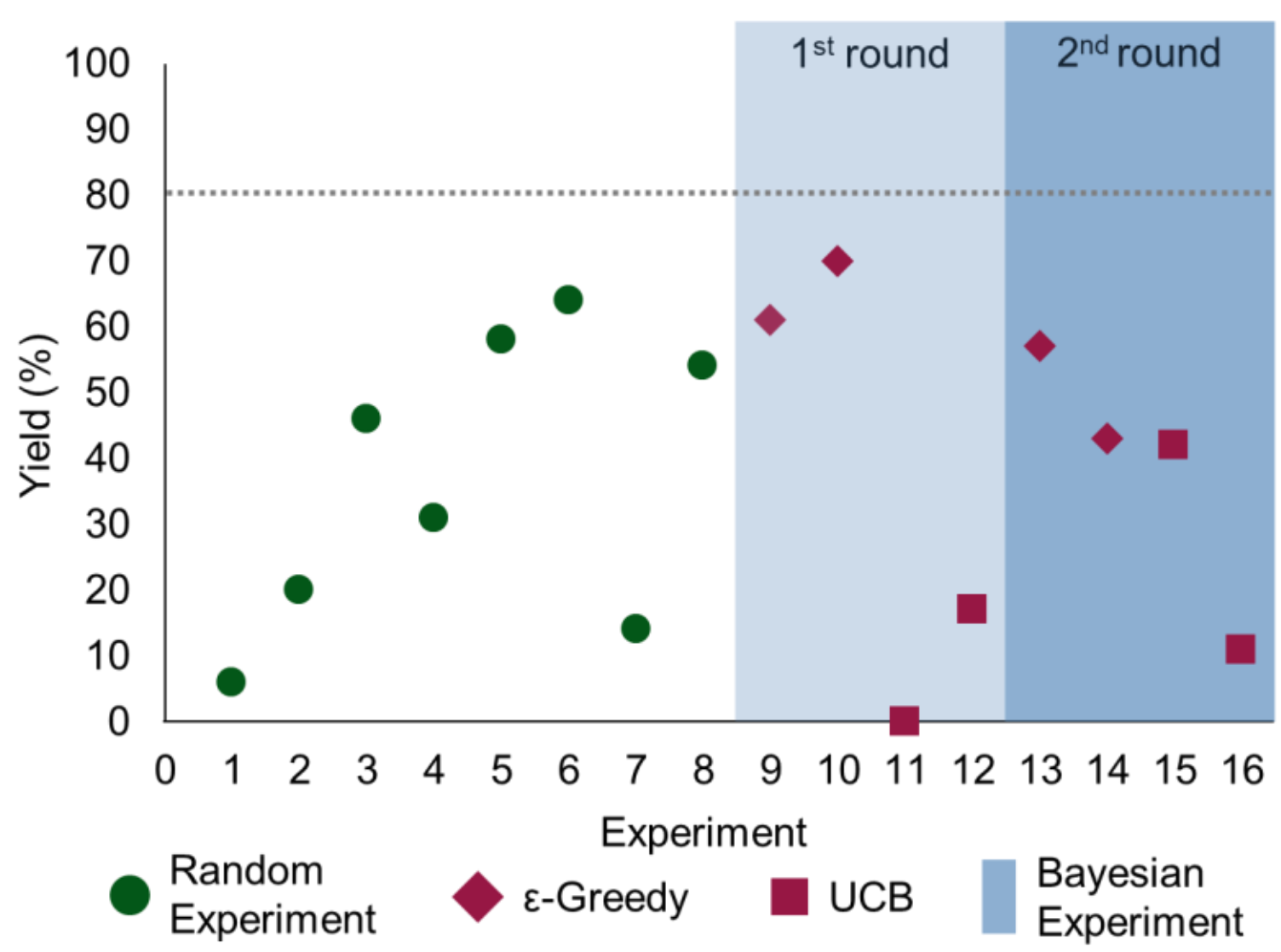

Figure S16

2o' – Methyl 6-*O*-acetyl-α-D-glucopyranoside

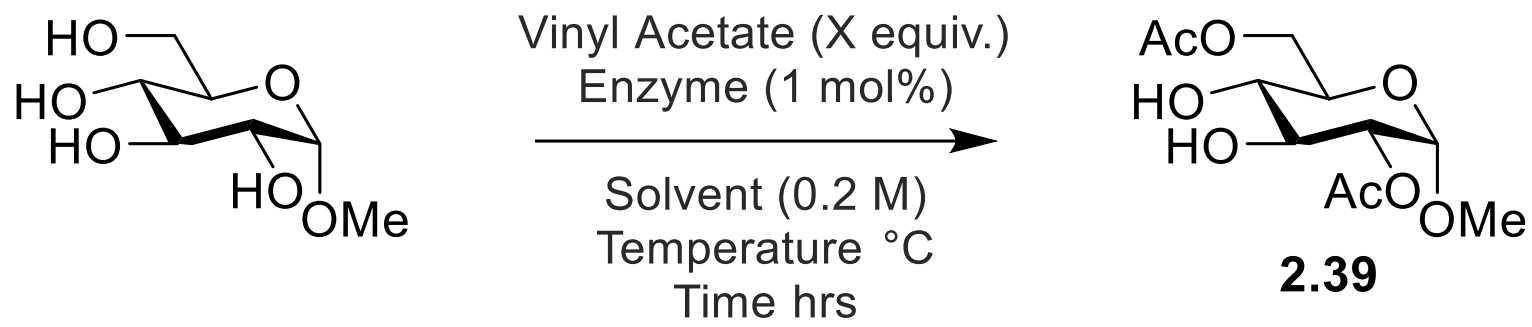

Round of Bayesian	Experiment Type	Enzyme	Solvent	Acyl equiv.	Time (hrs)	Temp (°C)	Yield (%)
Round 0	Random 1	PFL	THF/Py (4:1)	5	12	60	21
	Random 2	PPL	THF/Py (4:1)	35	6	20	3
	Random 3	PPL	THF	10	6	50	22
	Random 4	PSL	THF	5	6	70	12
	Random 5	PSL	THF/Py (4:1)	35	12	65	7
	Random 6	PPL	Pyridine	40	6	60	13
	Random 7	PSL	MeCN	25	24	20	2
	**Random 8**	**PPL**	**THF/Py (4:1)**	**25**	**18**	**50**	**37**
Round 1	Bayesian (ε-greedy) 1	PPL	THF/Py (4:1)	25	6	35	15
	Bayesian (ε-greedy) 2	PPL	THF/Py (4:1)	25	6	40	23
	Bayesian (UCB) 1	PFL	THF	55	72	20	11
	Bayesian (UCB) 2	PFL	MeCN	55	6	70	14
Round 2	Bayesian (ε-greedy) 1	PPL	THF/Py (4:1)	15	72	70	15
	Bayesian (ε-greedy) 2	PPL	THF/Py (4:1)	20	72	70	24
	Bayesian (UCB) 1	PPL	THF/Py (4:1)	5	72	70	7
	Bayesian (UCB) 2	PPL	THF/Py (4:1)	55	72	70	37

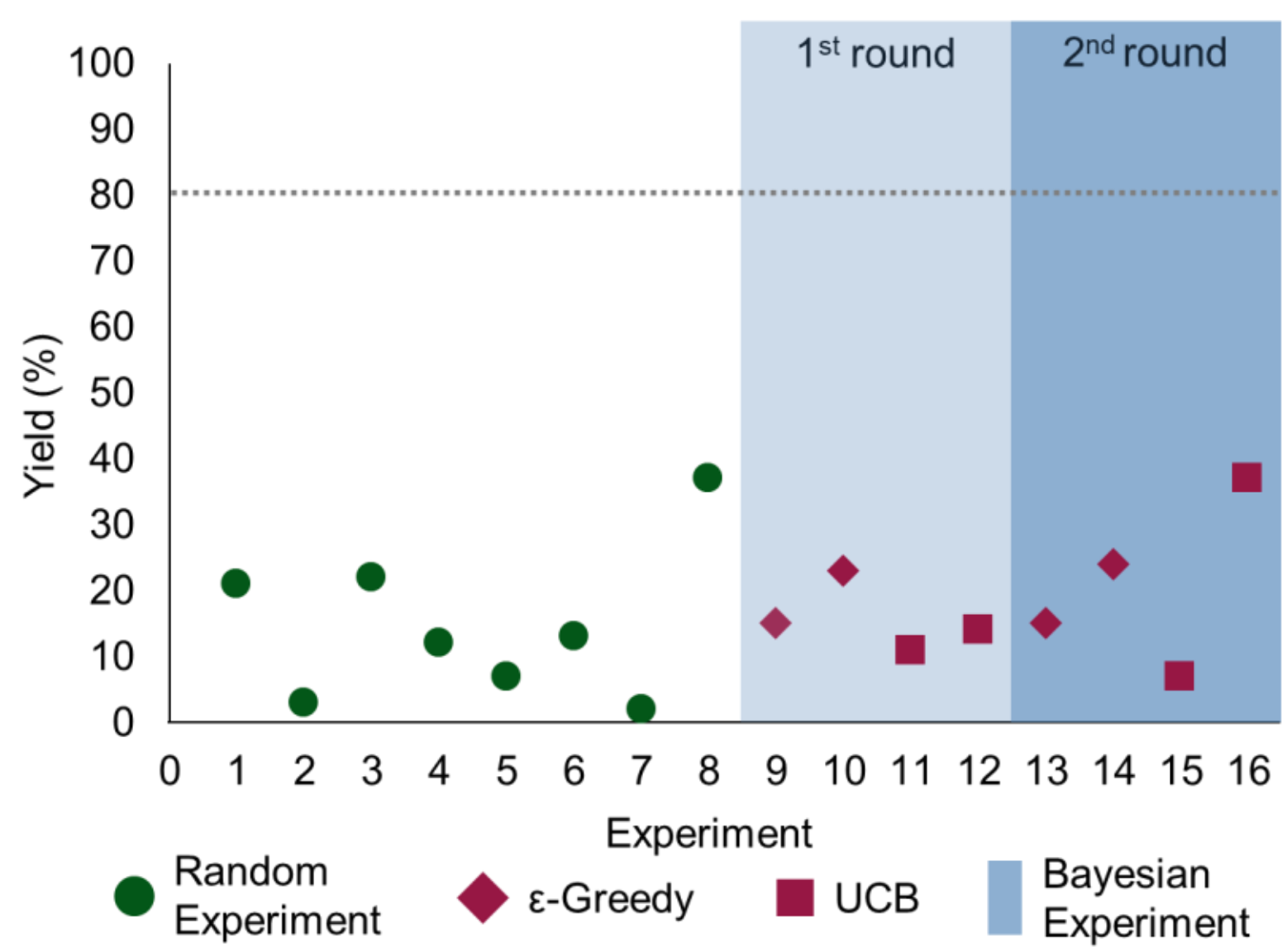

Figure S17

2p – Methyl 6-*O*-acetyl-β-D-galactopyranoside

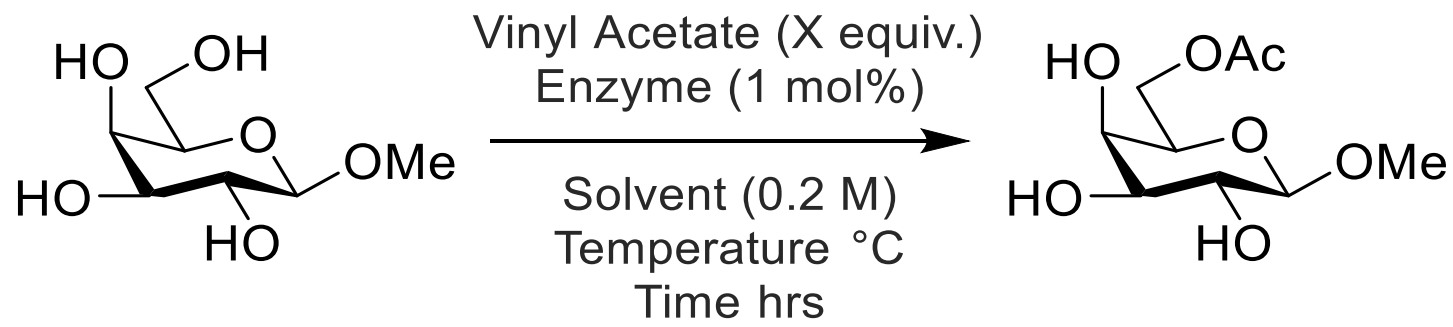

Round of Bayesian	Experiment Type	Enzyme	Solvent	Acyl equiv.	Time (hrs)	Temp (°C)	Yield (%)
Round 0	Random 1	PFL	MeCN	5	18	25	26
	Random 2	PPL	THF	40	36	30	85
	Random 3	PPL	THF	30	72	50	64
	Random 4	PFL	Pyridine	15	48	60	12
	Random 5	PSL	THF/Py (4:1)	10	36	60	16
	Random 6	PFL	THF/Py (4:1)	45	36	60	22
	Random 7	PSL	MeCN	35	6	65	12
	Random 8	PPL	THF	25	72	70	10
Round 1	Bayesian (ε-greedy) 1	PPL	THF	55	36	20	90
	Bayesian (ε-greedy) 2	PPL	THF	50	36	20	88
	**Bayesian (UCB) 1**	**PPL**	**Pyridine**	**55**	**72**	**20**	**99**
	Bayesian (UCB) 2	PSL	THF	55	72	20	21
Round 2	Bayesian (ε-greedy) 1	PFL	MeCN	5	18	25	26
	Bayesian (ε-greedy) 2	PPL	THF	40	36	30	85
	Bayesian (UCB) 1	PPL	THF	30	72	50	64
	Bayesian (UCB) 2	PFL	Pyridine	15	48	60	12

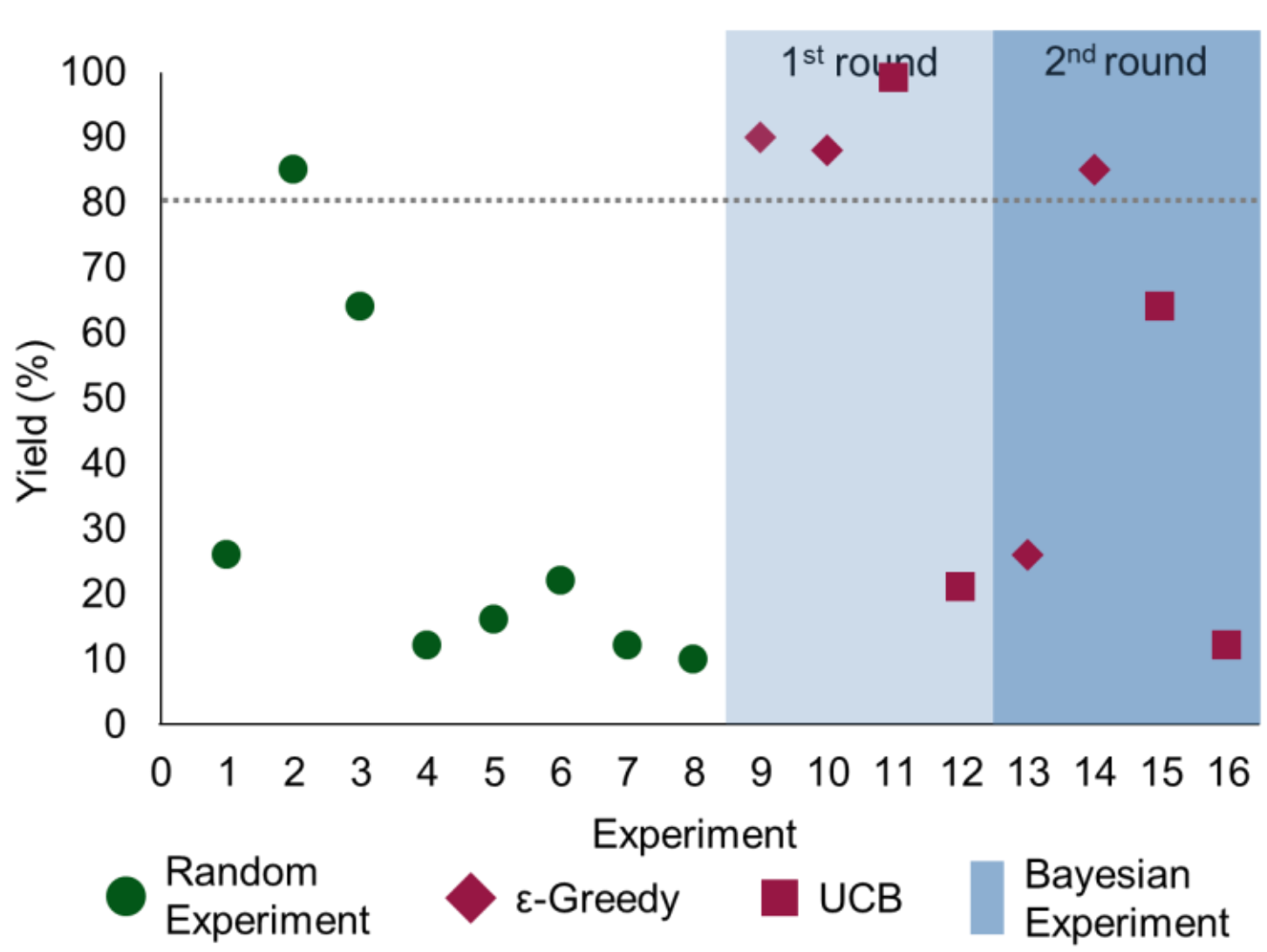

Figure S18

2p' – Methyl 6-*O*-acetyl-β-ᴅ-galactopyranoside

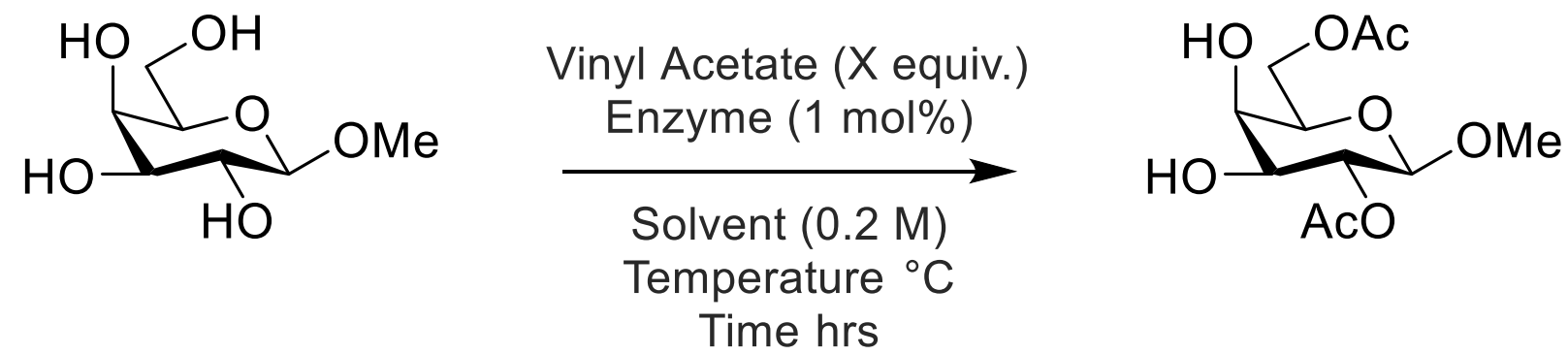

Round of Bayesian	Experiment Type	Enzyme	Solvent	Acyl equiv.	Time (hrs)	Temp (°C)	Yield (%)
Round 0	Random 1	PFL	MeCN	5	18	25	3
	Random 2	PPL	THF	40	36	30	5
	Random 3	PPL	THF	30	72	50	1
	Random 4	PFL	Pyridine	15	48	60	26
	Random 5	PSL	THF/Py (4:1)	10	36	60	27
	Random 6	PFL	THF/Py (4:1)	45	36	60	49
	Random 7	PSL	MeCN	35	6	65	9
	Random 8	PPL	THF	25	72	70	9
Round 1	Bayesian (ε-greedy) 1	PFL	THF/Py (4:1)	55	36	70	39
	Bayesian (ε-greedy) 2	PFL	THF/Py (4:1)	55	24	70	41
	Bayesian (UCB) 1	PPL	THF/Py (4:1)	55	6	70	2
	Bayesian (UCB) 2	PSL	Pyridine	55	72	20	21
Round 2	**Bayesian (ε-greedy) 1**	**PFL**	**THF/Py (4:1)**	**30**	**48**	**50**	**48**
	Bayesian (ε-greedy) 2	PFL	THF/Py (4:1)	35	48	50	43
	Bayesian (UCB) 1	PFL	THF	5	6	70	23
	Bayesian (UCB) 2	PFL	THF/Py (4:1)	5	72	20	39

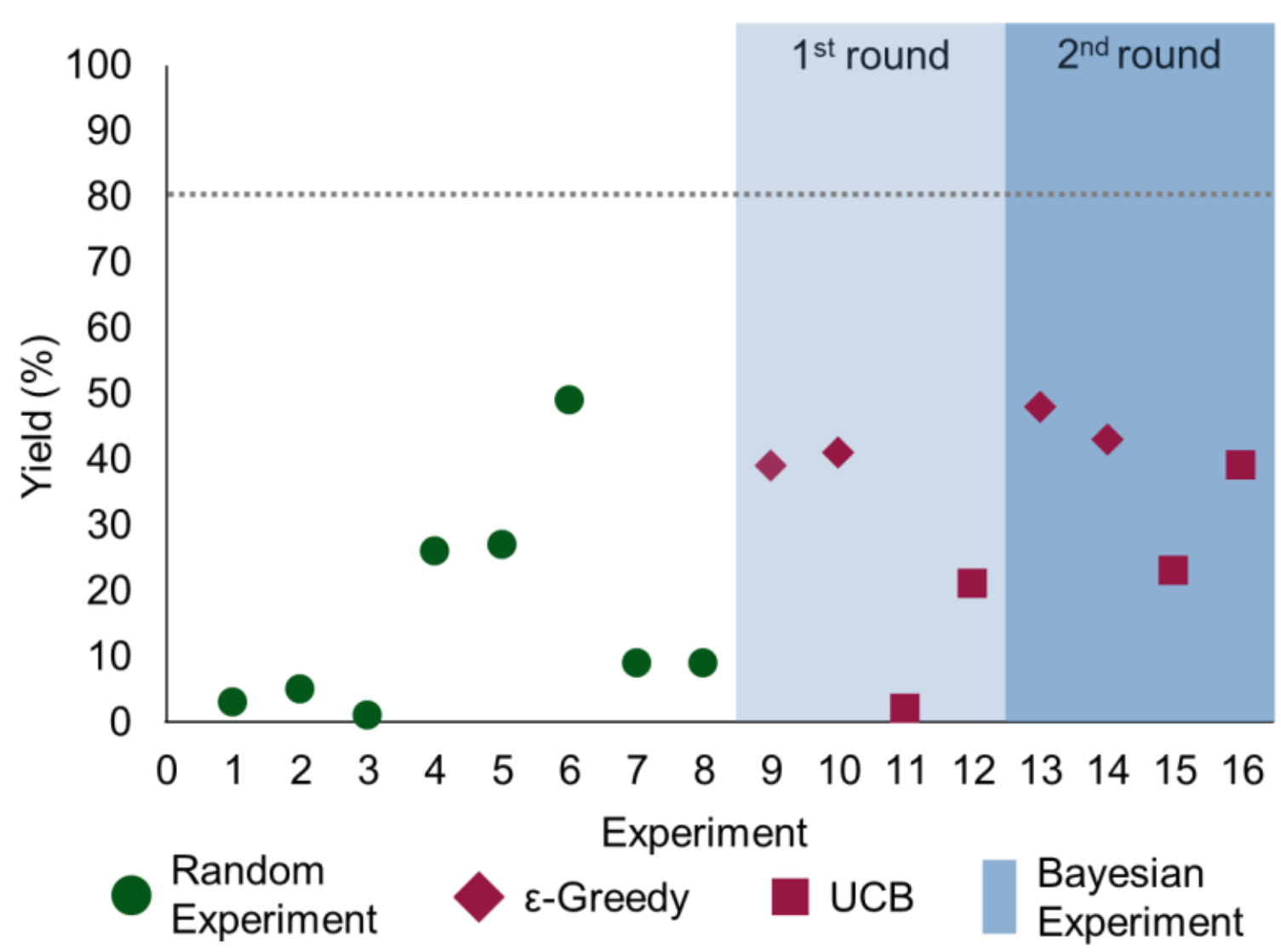

Figure S19

2q – 6-*O*-acetyl-Isopropyl-β-ᴅ-Thiogalactopyranoside

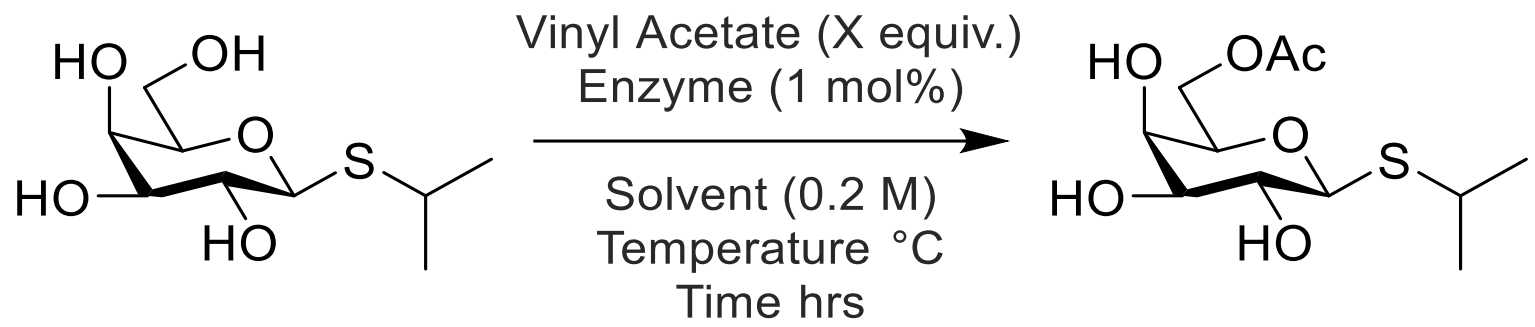

Round of Bayesian	Experiment Type	Enzyme	Solvent	Acyl equiv.	Time (hrs)	Temp (°C)	Yield (%)
Round 0	Random 1	PFL	THF/Py (4:1)	40	18	20	85
	Random 2	PFL	THF	30	48	20	22
	Random 3	PSL	THF/Py (4:1)	35	36	35	89
	**Random 4**	**PSL**	**Pyridine**	**55**	**36**	**40**	**97**
	Random 5	PFL	Pyridine	10	72	45	90
	Random 6	PSL	Pyridine	20	36	55	88
	Random 7	PFL	MeCN	30	6	65	44
	Random 8	PPL	THF	35	48	65	80

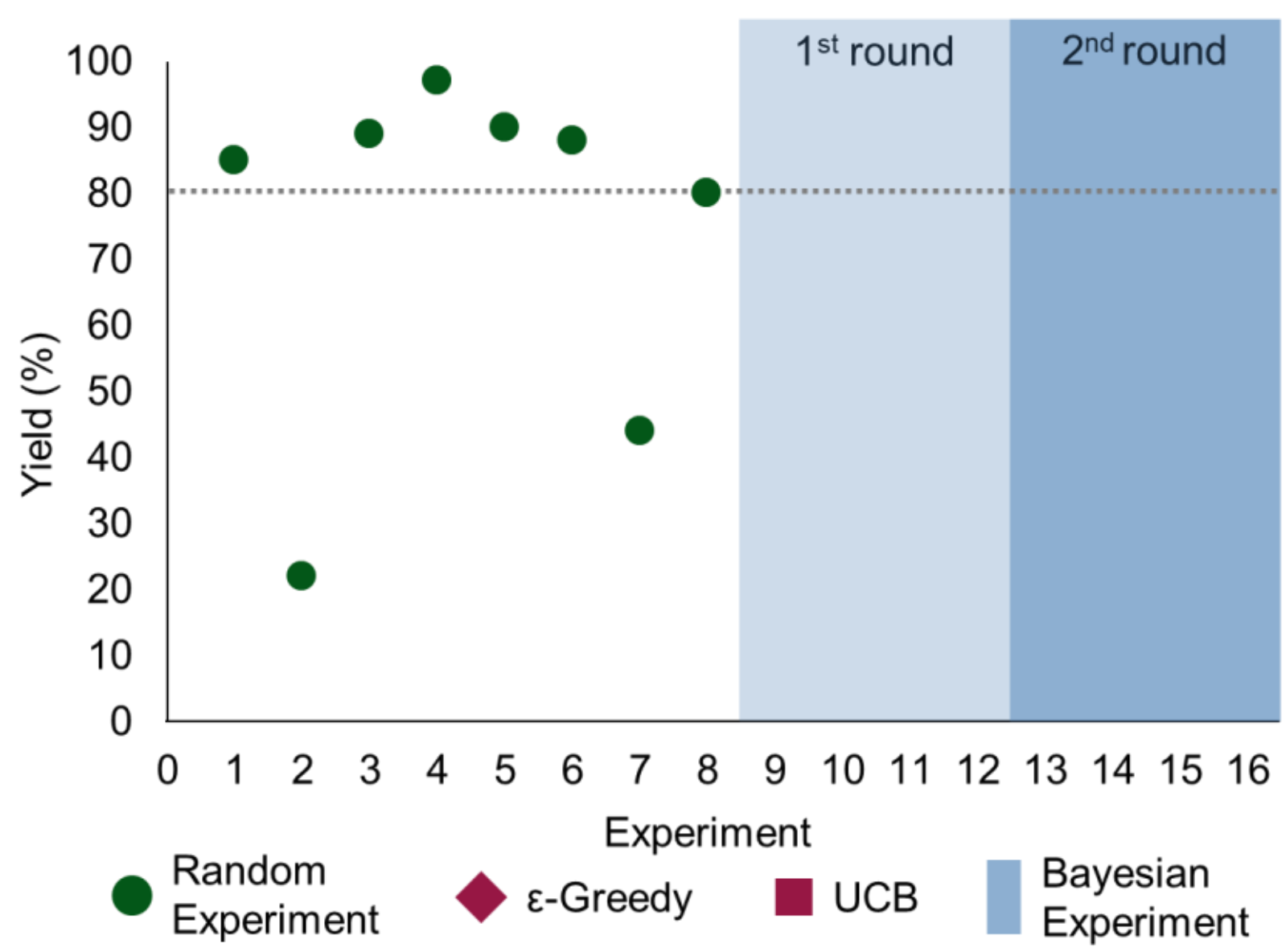

Figure S20

2r – Methyl 6-*O*-acetyl-α-D-mannopyranoside

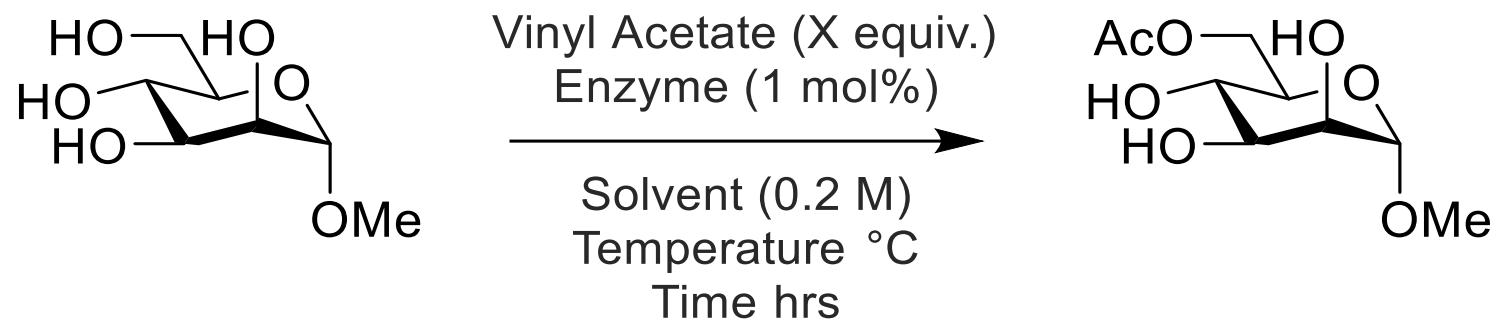

Round of Bayesian	Experiment Type	Enzyme	Solvent	Acyl equiv.	Time (hrs)	Temp (°C)	Yield (%)
Round 0	Random 1	PFL	MeCN	25	12	20	2
	Random 2	PPL	Pyridine	25	12	20	59
	Random 3	PFL	MeCN	10	6	30	1
	Random 4	PSL	MeCN	45	12	30	4
	Random 5	PSL	THF/Py (4:1)	45	18	35	7
	Random 6	PFL	THF	5	48	50	12
	Random 7	PFL	THF/Py (4:1)	55	24	50	15
	Random 8	PFL	Pyridine	45	36	65	1
Round 1	Bayesian (ε-greedy) 1	PPL	Pyridine	5	48	60	48
	Bayesian (ε-greedy) 2	PPL	Pyridine	20	6	20	51
	Bayesian (UCB) 1	PPL	THF/Py (4:1)	5	72	70	57
	**Bayesian (UCB) 2**	**PPL**	**THF**	**55**	**6**	**70**	**98**

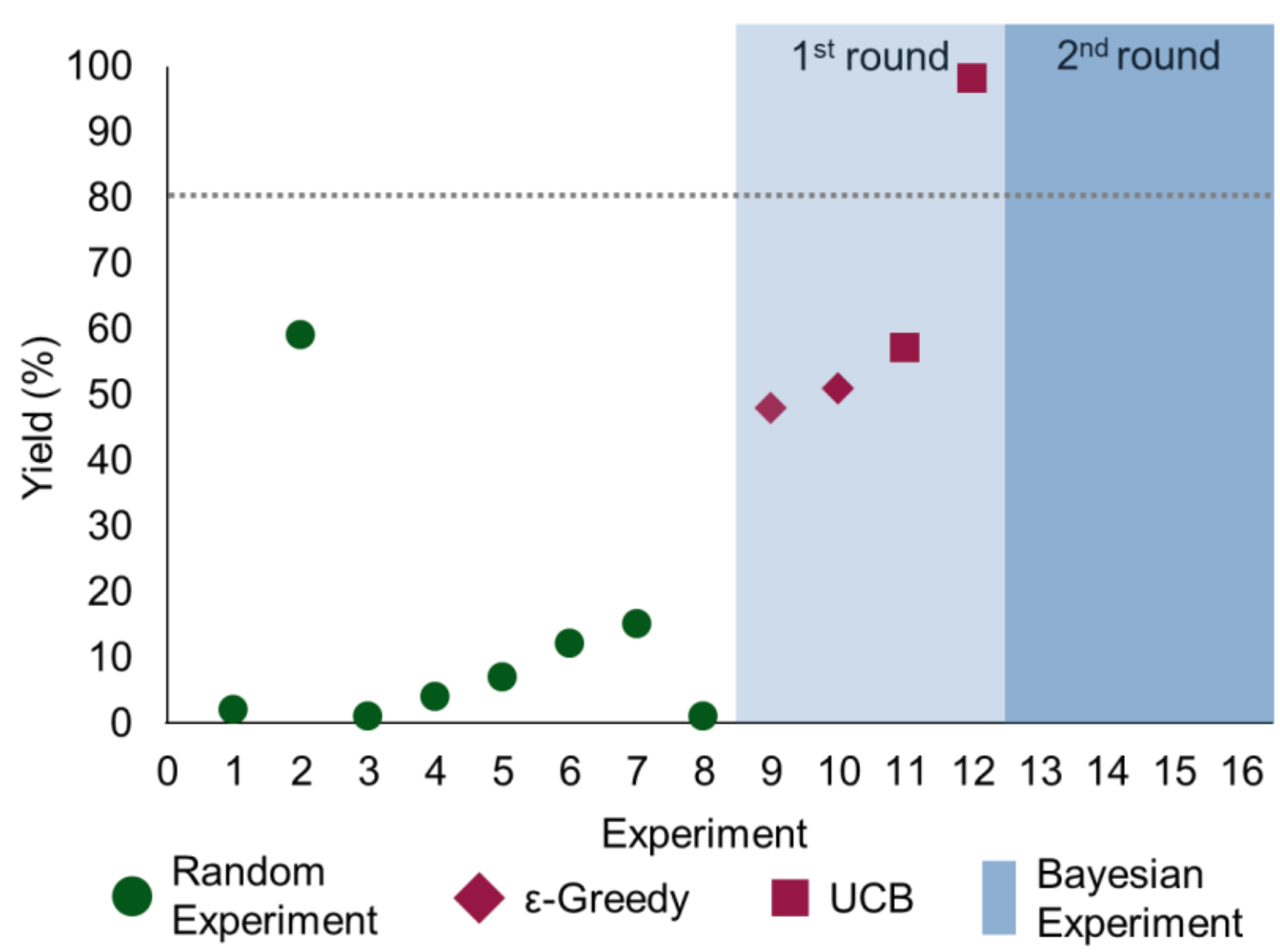

Figure S21

2w – Methyl 2-*O*-acetyl-5-*O*-(tert-butyldimethylsilyl)-α-ᴅ-ribofuranoside

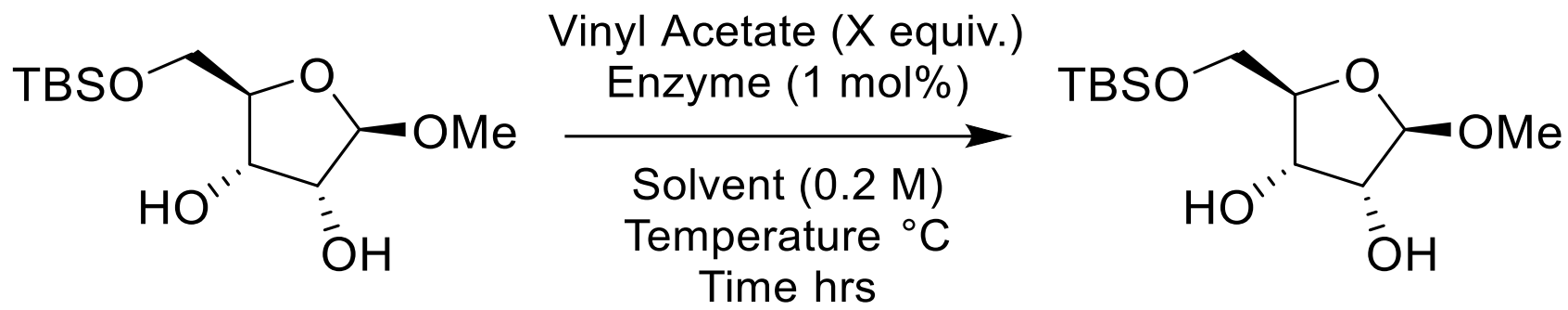

Round of Bayesian	Experiment Type	Enzyme	Solvent	Acyl equiv.	Time (hrs)	Temp (°C)	Yield (%)
Round 0	Random 1	PPL	THF	55	24	25	0
	Random 2	PFL	THF/Py (4:1)	35	6	30	0
	Random 3	PFL	Pyridine	15	36	20	0
	Random 4	PSL	Pyridine	45	72	35	0
	Random 5	PSL	THF/Py (4:1)	30	72	60	0
	Random 6	PFL	THF/Py (4:1)	20	18	50	0
	Random 7	PSL	THF	10	18	50	0
	Random 8	PPL	MeCN	55	36	60	0

Comparisons to Literature

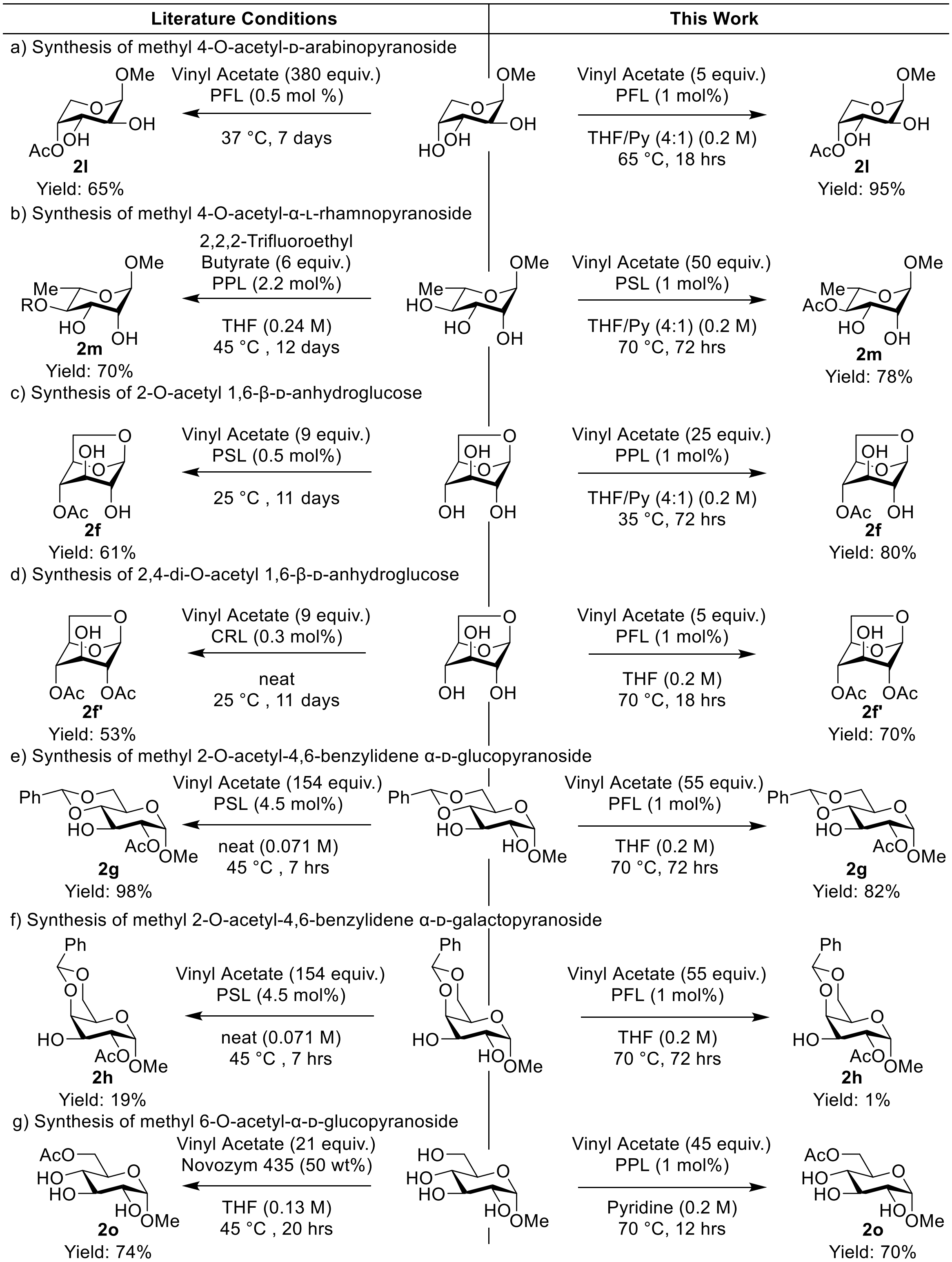

Many of the substrates used in this investigation have previously been synthesized enzymatically and reported in literature. As such, comparisons to previous conditions can be made to assess the

merit of the individualized Bayesian optimization conditions. In most cases the yields observed with this method were on par with or improved upon existing conditions. Bayesian optimization to methyl 4-O-acetyl-β-D-arabinopyranoside (**2l**) found conditions that improved the yield by 30%. The same enzyme was used in both cases but with a notable decrease in reaction time (18 hours compared to 7 days) and significantly decreased acyl donor loading.[6] New conditions for the acetylation of methyl 4-O-acetyl-α-L-rhamnopyranoside resulted in a similar yield of product **2m** but with improvements to the efficiency of the transformation with the reaction time decreasing from previously reported 12 days down to 72 hours.[7] Both the 4-O-acetyl and 2,4-di-O-acetyl derivatives of 1,6-anhydro-β-D-glucopyranose (**2f** and **2f'**) were previously synthesized enzymatically.[8,9] The new methods determined through the optimization campaigns saw a 20% increase in yield of both products.

Benzylidene-protected sugars **2g** and **2h** exhibited diminished performance compared to literature reports, albeit with significantly decreased enzyme loading.[10,11,12] Methyl 2-O-acetyl-4,6-benzylidene-α-D-glucopyranoside **2g** was synthesized in an 82% yield using Bayesian optimized conditions compared to the quantitative yield reported in literature. Methyl 2-O-acetyl-4,6-benzylidene-α-D-galactopyranoside **2h** was unsuccessful during initial Bayesian optimization attempts. This is not dissimilar to literature, which reports a yield of 19% for **2h**.

Primary acylation of methyl α-D-galactopyranoside **2o** performed very similar to literature conditions, albeit with very different reaction conditions.[13]

Transfer Learning

Yield Prediction

Modeling for yield prediction was conducted using scikit-learn through Python. Pipeline was used for feature processing, with OneHotEncoder for categorical features and mean imputation for numerical features, unless stated otherwise. Model performance was evaluated using R^2 (identity), RMSD, MAE, MBE and Pearson correlation coefficient. Generalization performance was assessed using leave-one-substrate-out (LOSO) cross validation.

The following substrate descriptors were used:

- Molecular weight
- Number of heavy (non-hydrogen) atoms
- Number of oxygen atoms
- Number of free OH groups
- Configration (gluco/galacto/manno)
- Free primary OH group (yes/no)

For substrates that underwent bis-acylation, the substrate descriptors were used to model the formation of monoacylated product, and the descriptors for the monoacylated product were used to model bis-acylation. This is an approximation, but it should be noted that the bis-acetylated products were generally obtained in relatively poor yields.

Untuned Gradient Boosting Model

The default hyperparameters from the scikit-learn Gradient Boosting Regressor were used: n_estimators = 100, learning_rate = 0.1, max_depth = 3, subsample = 1.0.

	R2_identity	RMSD	MAE	MBE	Pearson_r
**FULL**	0.86	11.6	8.98	-1.48	0.93
**LOSO**	0.17	27.67	21.7	0.57	0.47

Tuned Gradient Boosting Model

Preprocessing: median imputation. The following hyperparameters were tuned: n_estimators (300, 600), learning_rate (0.03, 0.05), max_depth (2, 3), subsample (0.7, 0.85), giving a total of 16 models. Relative to the default hyperparameters, the tuning grid explores lower learning rates to reduce overfitting, with a compensating increase in the number of estimators. The best-performing model, as judged by LOSO MAE, used the following hyperparameters: n_estimators = 300, learning_rate = 0.03, max_depth = 3, subsample = 0.7.

learning_rate	n_estimators	max_depth	subsample	R2_identity	MAE	RMSD	Pearson_r
**0.03**	300	3	0.7	0.27	20.3	26.1	0.53
**0.05**	300	3	0.7	0.26	20.4	26.3	0.54
**0.03**	600	3	0.7	0.24	20.4	26.5	0.53
**0.05**	600	3	0.7	0.24	20.5	26.6	0.54
**0.03**	600	3	0.85	0.23	20.6	26.8	0.52
**0.03**	300	3	0.85	0.24	20.7	26.4	0.52
**0.03**	300	2	0.7	0.26	20.9	26.2	0.52
**0.05**	300	3	0.85	0.21	21.0	27.1	0.50
**0.05**	600	3	0.85	0.18	21.3	27.5	0.50
**0.05**	300	2	0.7	0.22	21.3	26.9	0.50
**0.03**	300	2	0.85	0.24	21.3	26.6	0.50
**0.05**	300	2	0.85	0.22	21.4	26.9	0.49
**0.03**	600	2	0.7	0.21	21.4	27.0	0.50
**0.03**	600	2	0.85	0.20	21.6	27.2	0.49
**0.05**	600	2	0.85	0.16	22.0	27.9	0.48
**0.05**	600	2	0.7	0.14	22.3	28.2	0.47

Random forest model

The default hyperparameters from the scikit-learn Random Forest Regressor were used: n_estimators = 100, criterion = “squared_error”, max_depth = non, min_samples_split = 2, min_samples_leaf = 1, max_features = “sqrt”, bootstrap = True.

	R2_identity	RMSD	MAE	MBE	Pearson_r
**FULL**	0.82	12.9	9.9	0.59	0.93
**LOSO**	0.28	25.9	20.6	1.08	0.53

Gaussian Process Regression model

StandardScaler was used for numeric features. The default hyperparameters from the scikit-learn Gaussian Process Regressor were used. Kernel parameters: constant value, length scale, noise level. Alpha = 1e–10, optimizer = L-BFGS-B, n_restarts_optimizer = 0.

kernel
0.987**2 * RBF(length_scale=1.66) + WhiteKernel(noise_level=0.0474)

	R2_identity	RMSD	MAE	MBE	Pearson_r
**FULL**	0.99	3.1	2.2	0.04	1.00
**LOSO**	0.31	25.4	20.1	-0.37	0.56

CatBoost model

OneHotEncoder was not used. The default hyperparameters from the scikit-learn CatBoost Regressor were used: iterations = 1000, learning-rate ~ 0.04, depth = 6, loss_function = RMSE, l2_leaf_reg = 3, bootstrap_type = Bayesian, random_strength = 1, bagging_temperature = 1.

	R2_identity	RMSD	MAE	MBE	Pearson_r
**FULL**	0.86	11.5	8.0	-2.5	0.94
**LOSO**	0.28	25.8	20.2	0.2	0.55

Warm-starting methodology

Each of the above models was used to generate a set of eight experimental conditions based on the substrate descriptors. To reduce the likelihood of including incremental changes among the initial eight experiments, conditions were chosen from the following grid:

Vinyl acetate loading: 5, 25, 55 equiv

Time: 6, 24, 48, 72 h

Temperature: 20, 40, 60, 70 °C

The four enzyme/substrate combinations giving the highest predicted yields were chosen, with two sets of conditions (vinyl acetate loading, temperature, time) for each.

Untuned Gradient Boosting Model

Substrate 1s

Enzyme	Solvent	Acyl equiv.	Time (hrs)	Temp (°C)	predicted_yield
PFL	MeCN	55	72	40	67.5
PFL	MeCN	55	72	60	67.4
PFL	THF	55	72	60	49.2
PFL	THF	55	72	40	47.7
PSL	MeCN	55	72	40	45.9
PSL	MeCN	55	72	60	45.8
PFL	THF/Py (4:1)	55	72	40	45.2
PFL	THF/Py (4:1)	55	72	60	44.5

Substrate 1t

Enzyme	Solvent	Acyl equiv.	Time (hrs)	Temp (°C)	predicted_yield
PFL	THF/Py (4:1)	25	72	40	71.5
PFL	THF/Py (4:1)	25	72	60	70.8
PFL	THF	25	72	60	67.0
PFL	THF	25	72	40	65.4
PSL	THF/Py (4:1)	25	72	40	62.1
PPL	THF/Py (4:1)	5	72	20	61.5
PPL	THF/Py (4:1)	25	72	20	61.3
PSL	THF/Py (4:1)	25	72	70	57.7

Substrate 1u

Enzyme	Solvent	Acyl equiv.	Time (hrs)	Temp (°C)	predicted_yield
PFL	MeCN	55	72	40	83.7
PFL	MeCN	55	72	60	82.8
PFL	THF/Py (4:1)	55	72	40	62.9
PFL	THF/Py (4:1)	55	72	60	61.4

PFL	THF	55	72	40	60.5
PFL	THF	55	72	60	59.9
PSL	MeCN	55	72	40	52.4
PSL	MeCN	55	72	60	51.5

Substrate 1v

Enzyme	Solvent	Acyl equiv.	Time (hrs)	Temp (°C)	predicted_yield
PPL	Pyridine	55	6	20	98.0
PPL	Pyridine	55	24	20	97.0
PPL	THF/Py (4:1)	55	6	70	90.4
PPL	THF/Py (4:1)	55	24	20	90.2
PSL	THF/Py (4:1)	55	72	60	83.6
PSL	THF/Py (4:1)	55	72	70	83.5
PSL	Pyridine	55	24	70	78.0
PSL	Pyridine	55	72	60	77.7

Tuned Gradient Boosting Model

Substrate 1s

Enzyme	Solvent	Acyl equiv.	Time (hrs)	Temp (°C)	predicted_yield_gb_tuned
PFL	THF	55	72	60	56.4
PFL	MeCN	55	72	40	56.2
PFL	MeCN	55	72	60	55.2
PFL	THF	55	72	40	55.2
PFL	THF/Py (4:1)	55	72	40	48.9
PFL	THF/Py (4:1)	55	72	60	47.3
PSL	MeCN	55	72	40	29.5
PSL	MeCN	55	72	60	28.5

Substrate 1t

Enzyme	Solvent	Acyl equiv.	Time (hrs)	Temp (°C)	predicted_yield_gb_tuned
PFL	THF/Py (4:1)	25	72	60	72.6
PFL	THF/Py (4:1)	25	72	70	71.9
PFL	THF	55	72	70	67.1
PFL	THF	55	72	60	64.9
PPL	THF/Py (4:1)	5	72	20	62.7
PPL	THF/Py (4:1)	25	72	20	61.5
PSL	THF/Py (4:1)	25	72	60	60.2
PSL	THF/Py (4:1)	25	72	70	58.2

Substrate 1u

Enzyme	Solvent	Acyl equiv.	Time (hrs)	Temp (°C)	predicted_yield_gb_tuned
PFL	MeCN	55	72	40	79.6
PFL	THF/Py (4:1)	55	72	40	74.5
PFL	THF	55	72	40	74.0
PFL	MeCN	55	72	60	72.9
PFL	THF	55	72	60	68.9
PFL	THF/Py (4:1)	55	72	60	66.9
PFL	Pyridine	55	72	40	47.3
PFL	Pyridine	55	72	60	40.3

Substrate 1v

Enzyme	Solvent	Acyl equiv.	Time (hrs)	Temp (°C)	predicted_yield_gb_tuned
PSL	THF/Py (4:1)	55	72	70	66.6
PPL	THF/Py (4:1)	55	48	70	66.6
PPL	THF/Py (4:1)	55	72	70	65.8

PSL	THF/Py (4:1)	55	72	60	65.5
PPL	Pyridine	55	6	60	65.3
PPL	Pyridine	55	6	70	64.9
PPL	THF	55	6	60	64.8
PPL	THF	55	6	70	64.3

Random Forest Model

Substrate 1s

Enzyme	Solvent	Acyl equiv.	Time (hrs)	Temp (°C)	predicted_yield_rf
PFL	THF	55	72	70	72.4
PFL	THF	55	72	60	71.7
PFL	THF/Py (4:1)	55	72	60	61.5
PFL	MeCN	55	72	60	60.0
PFL	THF/Py (4:1)	55	72	70	59.4
PFL	MeCN	55	72	70	57.2
PFL	Pyridine	55	72	60	42.2
PFL	Pyridine	55	72	40	41.4

Substrate 1t

Enzyme	Solvent	Acyl equiv.	Time (hrs)	Temp (°C)	predicted_yield_rf
PFL	THF/Py (4:1)	5	72	40	66.5
PFL	THF/Py (4:1)	5	72	60	63.7
PFL	THF	55	72	70	62.6
PPL	THF/Py (4:1)	5	72	40	59.8
PSL	THF/Py (4:1)	5	72	40	58.7
PPL	THF/Py (4:1)	25	72	40	58.7
PFL	THF	5	72	70	57.6
PSL	THF/Py (4:1)	5	72	60	57.4

Substrate 1u

Enzyme	Solvent	Acyl equiv.	Time (hrs)	Temp (°C)	predicted_yield_rf
PFL	THF/Py (4:1)	55	72	40	79.3
PFL	THF	55	72	40	77.7
PFL	MeCN	55	72	40	76.1
PFL	THF	55	72	60	74.7
PFL	THF/Py (4:1)	55	72	60	73.3
PFL	THF	55	72	70	70.7
PFL	MeCN	55	72	60	70.1
PFL	Pyridine	55	72	40	62.6
PFL	Pyridine	55	72	60	55.1

Substrate 1v

Enzyme	Solvent	Acyl equiv.	Time (hrs)	Temp (°C)	predicted_yield_rf
PPL	THF/Py (4:1)	55	48	70	48.7
PPL	Pyridine	55	48	40	48.7
PPL	THF	55	48	70	48.6
PPL	Pyridine	55	48	70	48.3
PPL	THF/Py (4:1)	55	48	40	48.0
PPL	THF	55	48	40	48.0
PPL	Pyridine	55	24	70	47.7
PPL	THF	55	48	60	47.7
PPL	THF/Py (4:1)	55	48	70	48.7

Gaussian Process Regression Model

Substrate 1s

Enzyme	Solvent	Acyl equiv.	Time (hrs)	Temp (°C)	predicted_yield_gpr
PFL	MeCN	55	72	40	39.9
PFL	MeCN	55	48	40	39.2
PFL	THF	55	72	20	32.7
PFL	THF	55	72	40	32.4
PFL	Pyridine	55	72	20	32.0
PSL	MeCN	55	72	40	32.0
PSL	MeCN	55	6	40	31.9
PFL	Pyridine	55	48	20	31.1

Substrate 1t

Enzyme	Solvent	Acyl equiv.	Time (hrs)	Temp (°C)	predicted_yield_gpr
PFL	THF/Py (4:1)	5	72	40	70.5
PPL	THF/Py (4:1)	25	72	40	65.0
PFL	THF/Py (4:1)	5	72	20	64.3
PPL	THF/Py (4:1)	5	72	40	63.4
PFL	THF	5	72	40	57.7
PFL	MeCN	55	72	70	57.0
PFL	THF	55	72	70	56.3
PFL	MeCN	25	72	70	52.9

Substrate 1u

Enzyme	Solvent	Acyl equiv.	Time (hrs)	Temp (°C)	predicted_yield_gpr
PFL	MeCN	55	72	40	32.8
PFL	MeCN	55	48	40	32.7
PFL	THF	55	72	20	31.9
PSL	MeCN	5	6	70	31.9

PFL	Pyridine	55	72	20	31.9
PSL	MeCN	55	6	40	31.9
PFL	THF	55	72	40	31.8
PFL	Pyridine	55	48	20	31.8

Substrate 1v

Enzyme	Solvent	Acyl equiv.	Time (hrs)	Temp (°C)	predicted_yield_gpr
PPL	Pyridine	25	48	40	63.0
PPL	THF	55	24	60	62.2
PPL	Pyridine	25	48	60	61.8
PPL	THF	55	24	70	61.0
PSL	Pyridine	25	48	60	60.4
PPL	Pyridine	25	24	40	59.6
PPL	THF	55	6	60	59.1
PSL	Pyridine	25	48	40	58.5

CatBoost Model

Substrate 1s

Enzyme	Solvent	Acyl equiv.	Time (hrs)	Temp (°C)	predicted_yield_catboost
PFL	THF	55	72	60	66.3
PFL	THF	55	72	70	65.6
PFL	THF/Py (4:1)	55	72	60	65.5
PFL	THF/Py (4:1)	55	72	70	65.4
PFL	MeCN	55	72	40	52.7
PFL	MeCN	55	48	40	47.7
PFL	Pyridine	55	72	40	40.7
PFL	Pyridine	55	48	40	35.4

Substrate 1t

Enzyme	Solvent	Acyl equiv.	Time (hrs)	Temp (°C)	predicted_yield_catboost
PFL	THF/Py (4:1)	55	72	70	66.6
PFL	THF/Py (4:1)	5	72	20	65.3
PFL	THF	55	72	70	65.1
PFL	THF	55	72	60	61.9
PPL	THF/Py (4:1)	55	72	70	52.8
PPL	THF/Py (4:1)	25	72	40	52.6
PSL	THF/Py (4:1)	55	72	70	52.2
PSL	THF/Py (4:1)	55	72	60	50.0

Substrate 1u

Enzyme	Solvent	Acyl equiv.	Time (hrs)	Temp (°C)	predicted_yield_catboost
PFL	THF/Py (4:1)	55	72	40	68.0
PFL	THF/Py (4:1)	55	72	60	65.5
PFL	MeCN	55	72	40	63.5
PFL	THF	55	72	40	61.8
PFL	THF	55	72	60	59.2
PFL	MeCN	55	48	40	53.0
PFL	Pyridine	55	72	40	52.9
PFL	Pyridine	55	48	40	43.3

Substrate 1v

Enzyme	Solvent	Acyl equiv.	Time (hrs)	Temp (°C)	predicted_yield_catboost
PPL	THF	55	6	70	64.0
PPL	THF/Py (4:1)	55	72	70	63.4
PSL	THF/Py (4:1)	55	72	70	62.8
PPL	THF	55	24	70	62.2
PPL	THF	55	6	60	61.2

PPL	THF/Py (4:1)	55	24	70	60.1
PPL	THF/Py (4:1)	55	48	70	60.0
PPL	Pyridine	55	6	70	58.5

Prediction of relative yields

We adapted the approach of Zimmerman, Cernak and co-workers[14] to our dataset, using the YetiRank algorithm implemented with CatBoost to build a ranking model based on the substrate descriptors and reaction conditions. Experiments were treated as (substrate, reaction conditions) pairs, considering substrates as independent ranking groups and using yield as the relevance figure. Numerical reaction condition variables were z-normalized with StandardScaler. Substrate descriptors (see above) were unscaled. One-hot encoding was not used for categorical features. Default hyperparameters were used: boosting iterations = 1000, learning rate ~ 0.03, tree depth = 6, Loss function = CatBoost default ranking objective, l2_leaf_reg = 3.

The mean reciprocal rank (MRR), defined as the average of 1/n, where *n* is the model-predicted rank of the ground-truth highest-yielding conditions, was calculated under LOSO cross-validation. For comparison, a baseline value of MRR was obtained using a LOSO evaluation scheme, by applying the average highest-ranked conditions across all substrates to each test substrate. The MRR for CatBoost ranking model is 0.45, a significant improvement on the baseline of 0.27; in other words, the average CatBoost-predicted ranking of the ground-truth best conditions is 2.2, versus 3.7 when applying the baseline model that assumes no relationship between substrate structure and optimum conditions.

Warm-starting experiments from the CatBoost model

Substrate 1s

Enzyme	Solvent	Acyl equiv.	Time (hrs)	Temp (°C)	rank_score
**PFL**	MeCN	55	72	70	4.13
**PFL**	THF	70	72	60	2.97
**PFL**	THF/Py (4:1)	55	72	70	2.19
**PSL**	MeCN	35	18	40	1.49
**PSL**	THF/Py (4:1)	50	72	70	1.15
**PPL**	MeCN	30	12	50	-0.73
**PFL**	Pyridine	55	72	20	-1.12
**PPL**	THF	55	36	70	-1.54

Substrate 1t

Enzyme	Solvent	Acyl equiv.	Time (hrs)	Temp (°C)	rank_score
**PFL**	THF	70	72	60	4.35
**PFL**	THF/Py (4:1)	55	72	70	3.62
**PSL**	THF/Py (4:1)	50	72	70	3.36
**PFL**	MeCN	55	72	70	3.05
**PSL**	MeCN	35	18	40	1.12
**PFL**	Pyridine	55	72	20	1.07
**PPL**	THF	55	36	70	0.07
**PPL**	THF	40	24	30	0.07

Substrate 1u

Enzyme	Solvent	Acyl equiv.	Time (hrs)	Temp (°C)	rank_score
**PFL**	MeCN	55	72	70	4.32
**PFL**	THF/Py (4:1)	55	72	70	1.42
**PSL**	MeCN	35	18	40	1.17
**PFL**	THF	70	72	60	0.81
**PSL**	THF/Py (4:1)	50	72	70	0.58
**PPL**	MeCN	30	12	50	-1.84
**PFL**	Pyridine	55	72	20	-2.79
**PSL**	Pyridine	25	12	50	-2.87

Substrate 1v

Enzyme	Solvent	Acyl equiv.	Time (hrs)	Temp (°C)	rank_score
PPL	THF	20	12	55	2.63
PPL	THF	20	6	5	2.63
PPL	THF/Py (4:1)	20	6	40	2.71
PPL	THF/Py (4:1)	20	24	30	2.71
PSL	THF	20	6	5	2.81

PSL	THF	20	6	15	2.81
PSL	THF/Py (4:1)	70	36	30	3.79
PSL	THF/Py (4:1)	70	48	25	3.79

Warm-Starting Experimental Results

2s – Methyl 6-*O*-trityl-2-*O*-acetyl-α-D-glucopyranoside

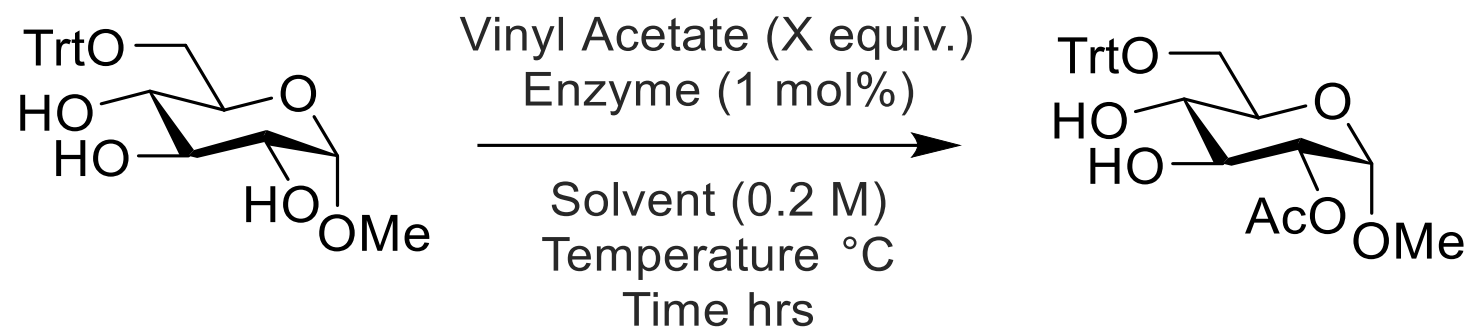

Experiment Type	Enzyme	Solvent	Acyl equiv.	Time (hrs)	Temp (°C)	Yield (%)
Warm-Start Regime 1	PFL	MeCN	55	72	40	35
Warm-Start Regime 1	PFL	MeCN	55	72	60	50
Warm-Start Regime 2	PSL	MeCN	55	72	40	18
Warm-Start Regime 2	PSL	MeCN	55	72	60	15
**Warm-Start Regime 3**	**PFL**	**THF**	**55**	**72**	**60**	**84**
Warm-Start Regime 3	PFL	THF	55	72	40	62
Warm-Start Regime 4	PFL	THF/Py (4:1)	55	72	40	40
Warm-Start Regime 4	PFL	THF/Py (4:1)	55	72	60	63

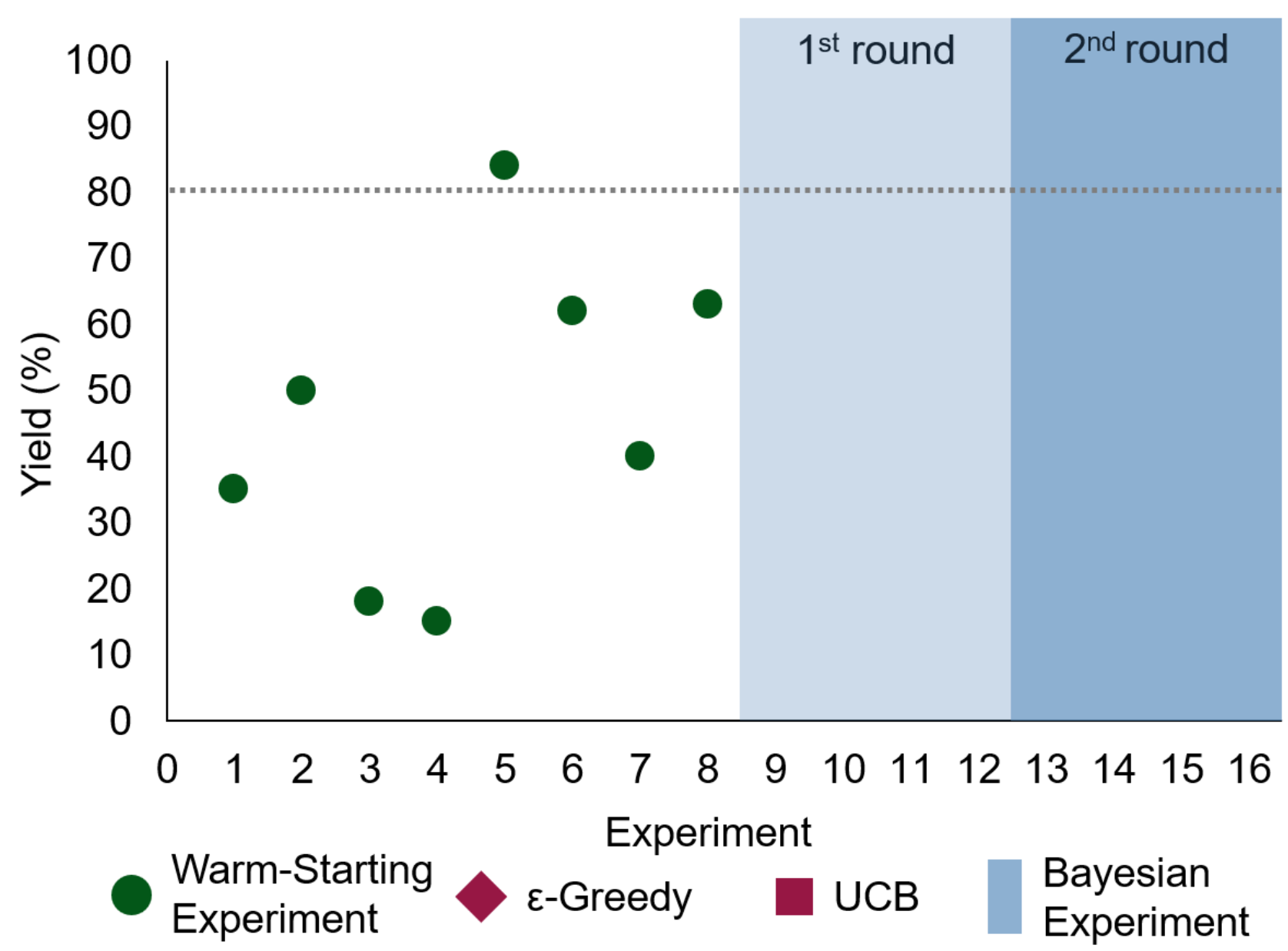

Figure S22

2t – Methyl 3,4-di-*O*-acetyl-β-ᴅ-xylopyranoside

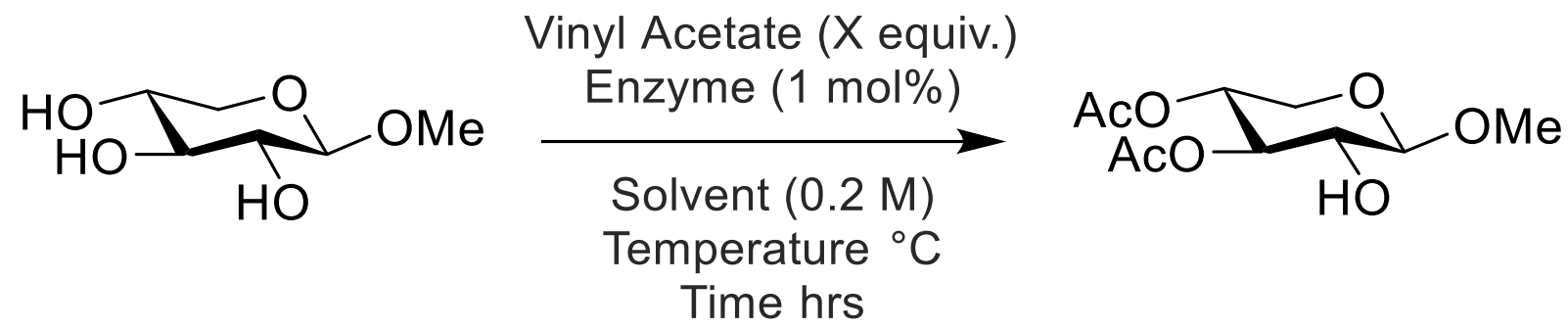

Experiment Type	Enzyme	Solvent	Acyl equiv.	Time (hrs)	Temp (°C)	Yield (%)
Warm-Start Regime 1	PFL	THF/Py (4:1)	25	72	40	78
Warm-Start Regime 1	PFL	THF/Py (4:1)	25	72	60	79
Warm-Start Regime 2	PFL	THF	25	72	60	77
Warm-Start Regime 2	PFL	THF	25	72	40	46
Warm-Start Regime 3	PPL	THF/Py (4:1)	5	72	20	0
Warm-Start Regime 3	PPL	THF/Py (4:1)	25	72	20	0
Warm-Start Regime 4	PSL	THF/Py (4:1)	25	72	40	33
Warm-Start Regime 4	PSL	THF/Py (4:1)	25	72	60	51
Bayesian (ε-greedy) 1	PFL	THF/Py (4:1)	35	72	70	75
**Bayesian (ε-greedy) 2**	**PFL**	**THF/Py (4:1)**	**30**	**72**	**70**	**82**

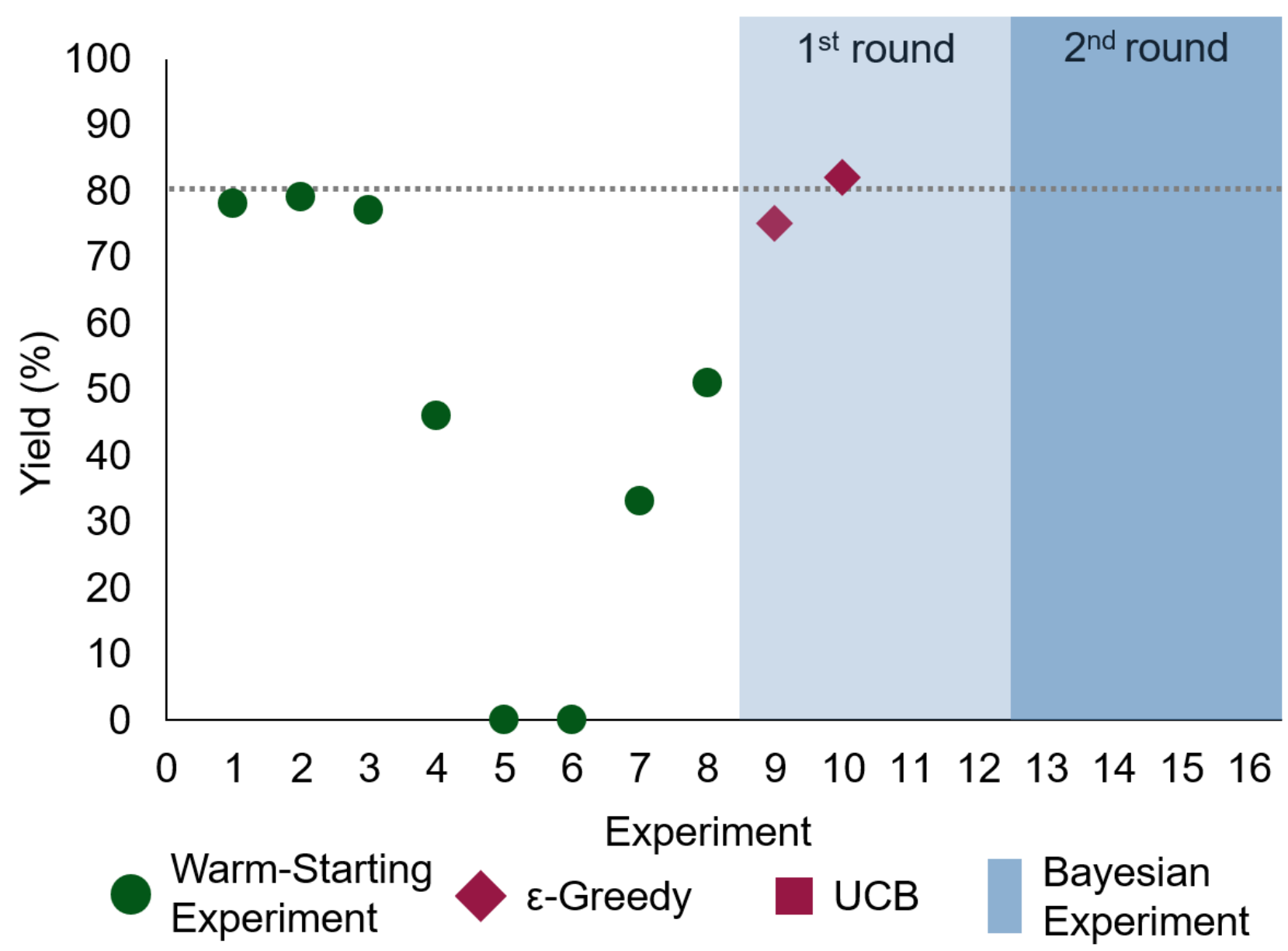

Figure S23

2u – Phenyl 1-thio-6-O-trityl-2-*O*-acetyl-β-ᴅ-galactopyranoside

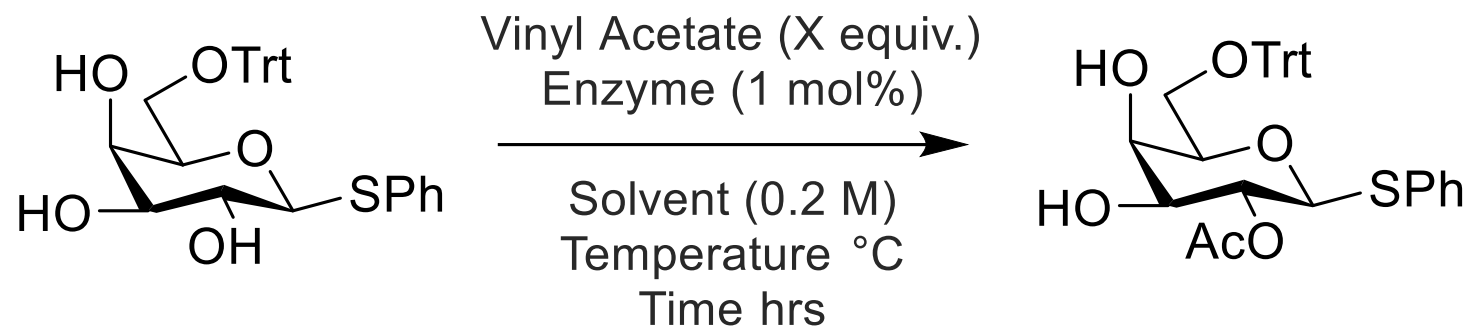

Experiment Type	Enzyme	Solvent	Acyl equiv.	Time (hrs)	Temp (°C)	Yield (%)
Warm-Start Regime 1	PFL	MeCN	55	72	40	22
Warm-Start Regime 1	PFL	MeCN	55	72	60	17
Warm-Start Regime 2	PFL	THF	55	72	40	28
Warm-Start Regime 2	PFL	THF	55	72	60	25
Warm-Start Regime 3	PFL	THF/Py (4:1)	55	72	40	31
Warm-Start Regime 3	PFL	THF/Py (4:1)	55	72	60	25
Warm-Start Regime 4	PSL	MeCN	55	72	40	22
Warm-Start Regime 4	PSL	MeCN	55	72	60	31
Bayesian (ε-greedy) 1	PFL	THF/Py (4:1)	55	72	20	11
Bayesian (ε-greedy) 2	PFL	THF/Py (4:1)	50	72	20	8

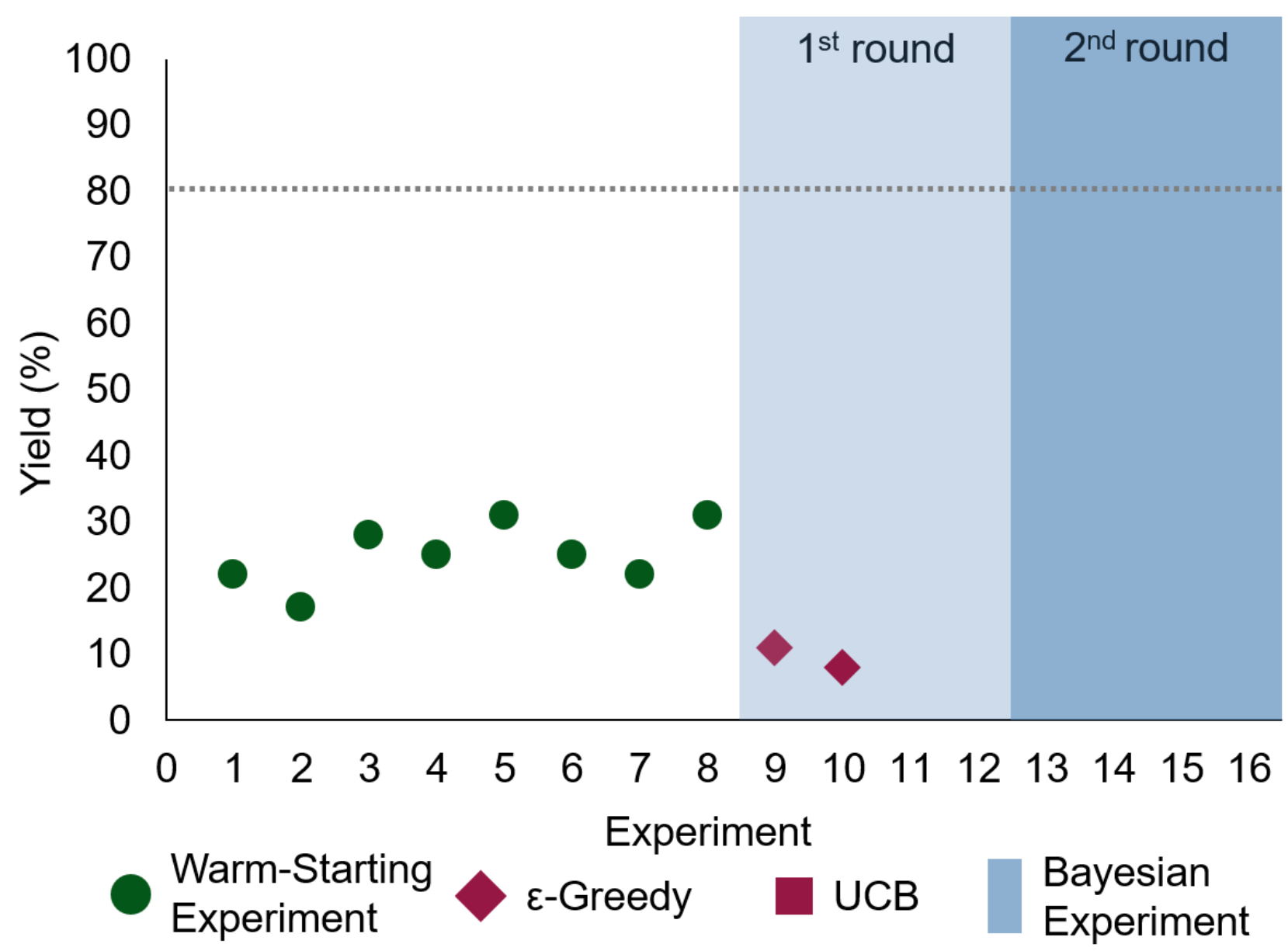

Figure S24

2v – Phenyl 6-*O*-acetyl-α-D-1-thio-mannopyranoside

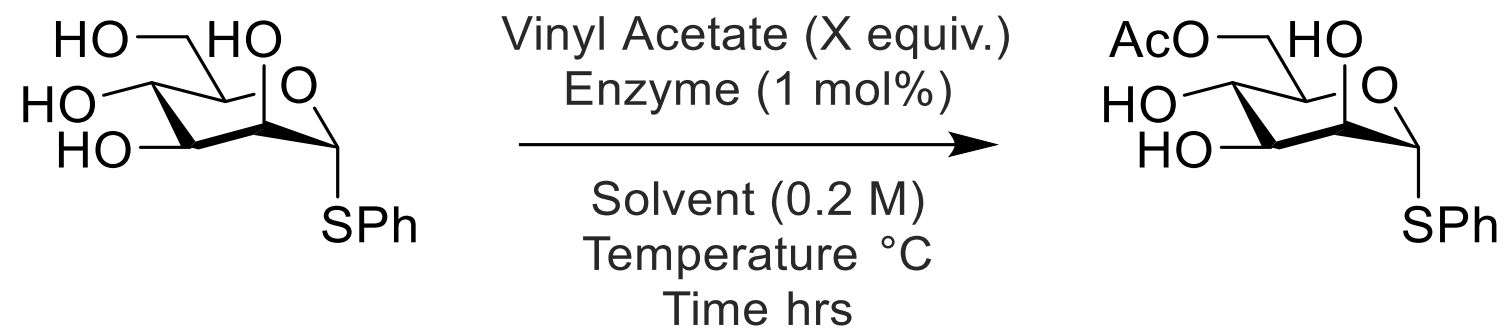

Experiment Type	Enzyme	Solvent	Acyl equiv.	Time (hrs)	Temp (°C)	Yield (%)
Warm-Start Regime 1	PPL	Pyridine	55	6	20	0
Warm-Start Regime 1	PPL	Pyridine	55	24	20	0
**Warm-Start Regime 2**	**PPL**	**THF/Py (4:1)**	**55**	**6**	**70**	**92**
Warm-Start Regime 2	PPL	THF/Py (4:1)	55	24	20	84
Warm-Start Regime 3	PSL	THF/Py (4:1)	55	72	60	87
Warm-Start Regime 3	PSL	THF/Py (4:1)	55	72	70	57
Warm-Start Regime 4	PSL	Pyridine	55	24	70	45
Warm-Start Regime 4	PSL	Pyridine	55	72	60	71

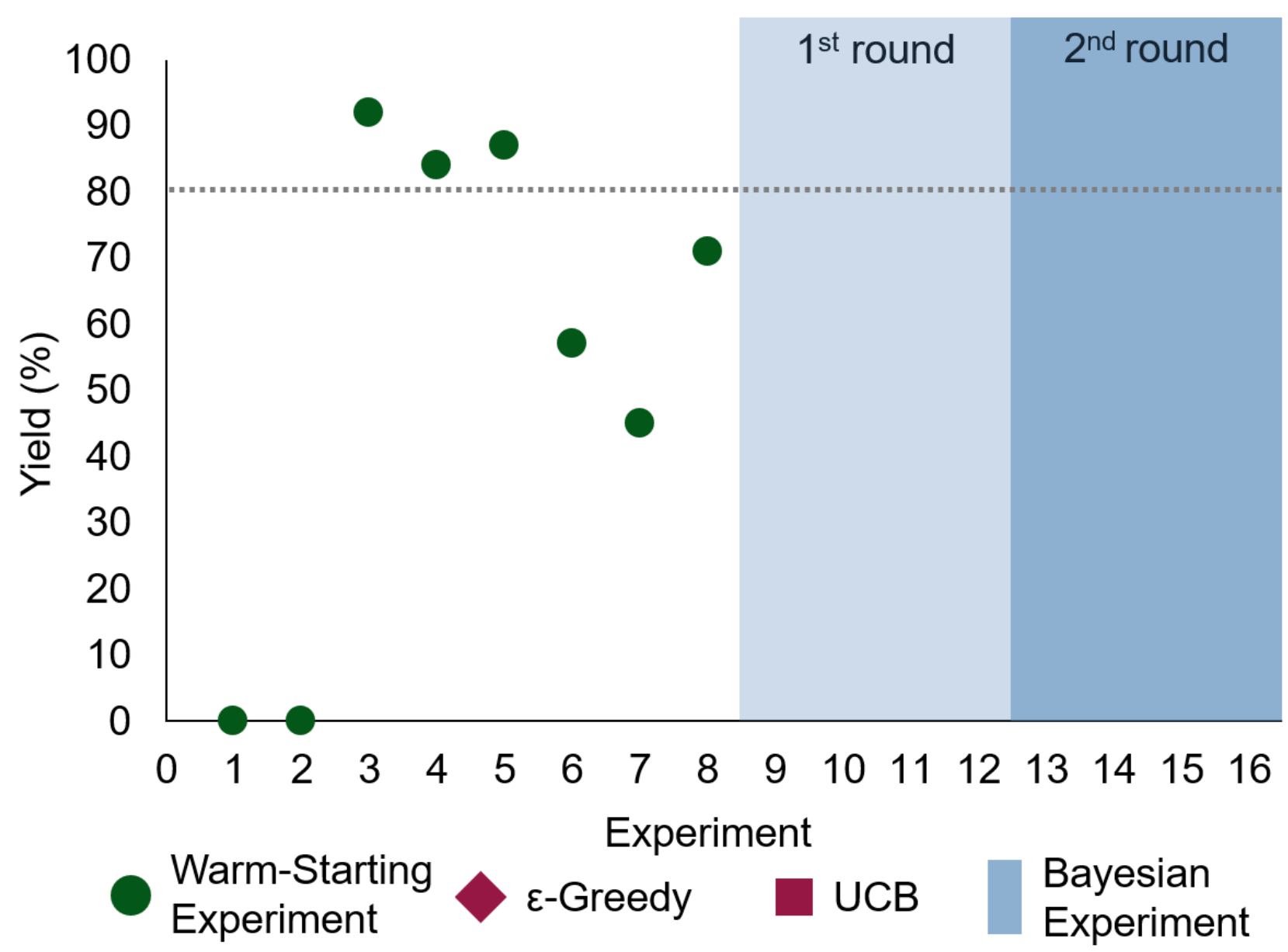

Figure S25

Comparison of transfer learning to most common reaction conditions

The transfer learning method was benchmarked against use of the "best 8" from the data acquired in the initial substrate scope. The top 8 conditions, as ranked by the number of instances of yield >80% were screened.

Experiment Type	Enzyme	Solvent	Acyl equiv.	Time (hrs)	Temp (°C)	Count (≥80%)
Top Conditions 1	PFL	THF/Py (4:1)	5	24	60	4
Top Conditions 2	PFL	THF	55	72	70	3
Top Conditions 2	PFL	THF/Py (4:1)	5	72	20	3
Top Conditions 3	PFL	THF/Py (4:1)	55	72	40	2
Top Conditions 3	PFL	THF/Py (4:1)	5	48	40	2
Top Conditions 3	PSL	THF/Py (4:1)	55	72	70	2
Top Conditions 3	PPL	THF	55	24	20	2
Top Conditions 4	PPL	THF/Py (4:1)	25	72	40	1

2s – Methyl 6-*O*-trityl-2-*O*-acetyl-α-ᴅ-glucopyranoside

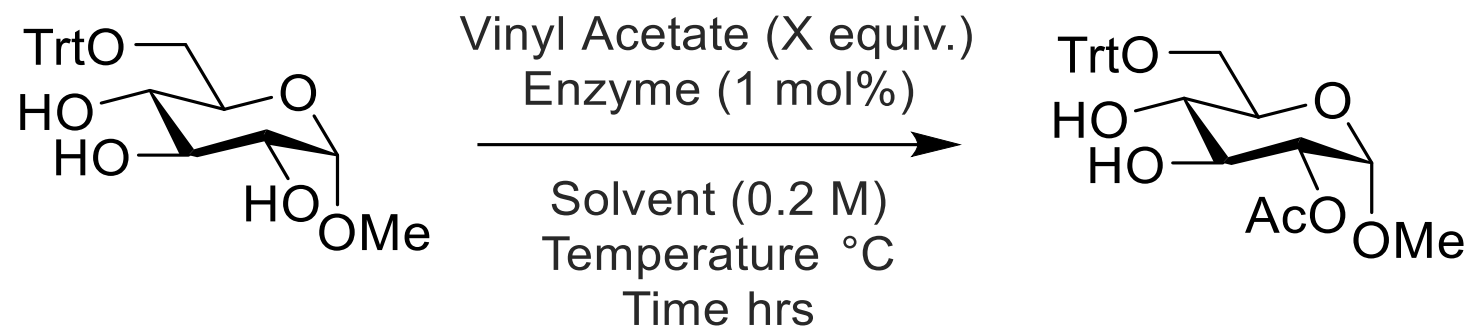

Experiment Type	Enzyme	Solvent	Acyl equiv.	Time (hrs)	Temp (°C)	Yield (%)
Top Conditions 1	PFL	THF/Py (4:1)	5	24	60	23
Top Conditions 2	PFL	THF	55	72	70	97
Top Conditions 2	PFL	THF/Py (4:1)	5	72	20	5
Top Conditions 3	PFL	THF/Py (4:1)	55	72	40	40
Top Conditions 3	PFL	THF/Py (4:1)	5	48	40	6
Top Conditions 3	PSL	THF/Py (4:1)	55	72	70	11
Top Conditions 3	PPL	THF	55	24	20	0
Top Conditions 4	PPL	THF/Py (4:1)	25	72	40	0

Average yield: 23% (compare to 46% for transfer learning)

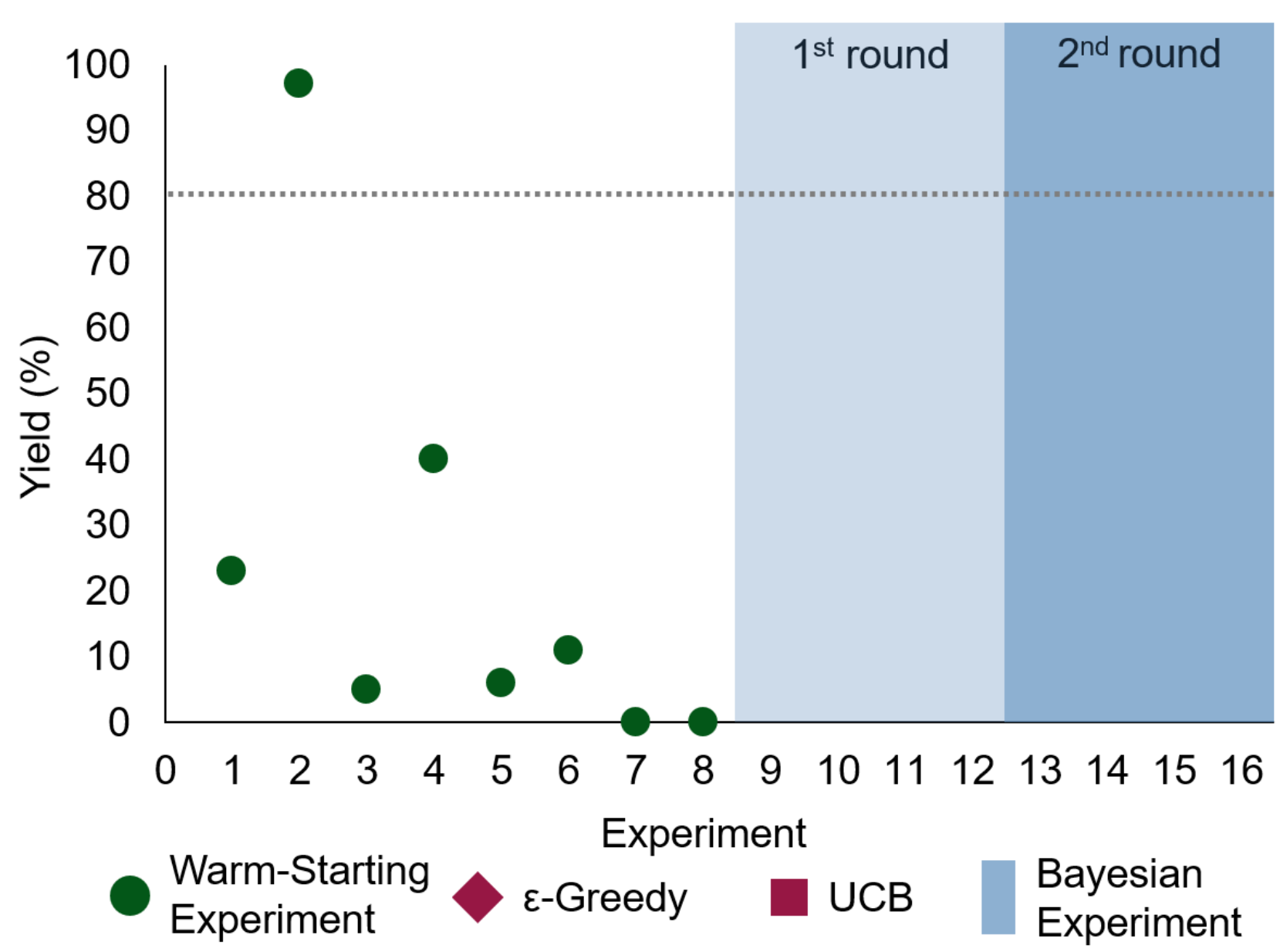

Figure S26

2t – Methyl 3,4-di-*O*-acetyl-β-D-xylopyranoside

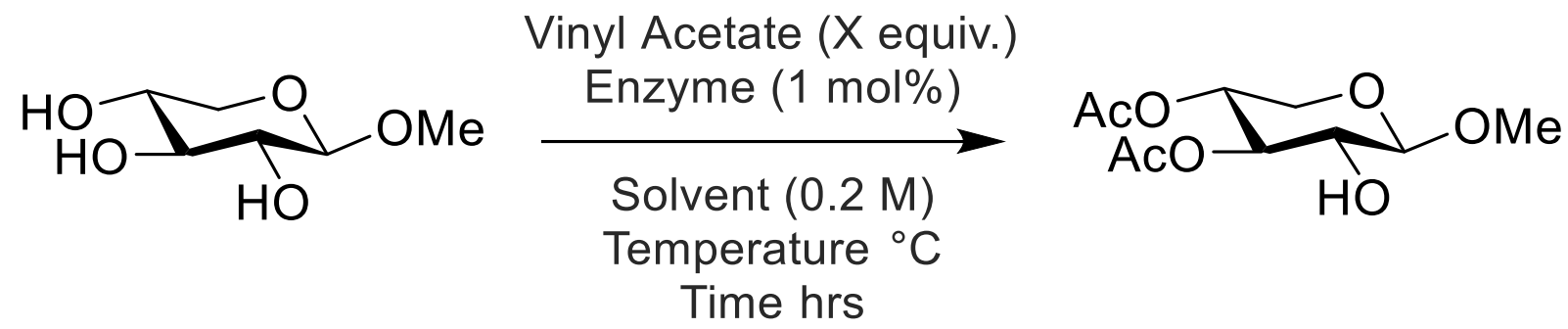

Experiment Type	Enzyme	Solvent	Acyl equiv.	Time (hrs)	Temp (°C)	Yield (%)
Top Conditions 1	PFL	THF/Py (4:1)	5	24	60	30
Top Conditions 2	PFL	THF	55	72	70	95
Top Conditions 2	PFL	THF/Py (4:1)	5	72	20	19
Top Conditions 3	PFL	THF/Py (4:1)	55	72	40	62
Top Conditions 3	PFL	THF/Py (4:1)	5	48	40	24
Top Conditions 3	PSL	THF/Py (4:1)	55	72	70	77
Top Conditions 3	PPL	THF	55	24	20	0
Top Conditions 4	PPL	THF/Py (4:1)	25	72	40	0

Average yield: 38% (compare to 46% for transfer learning)

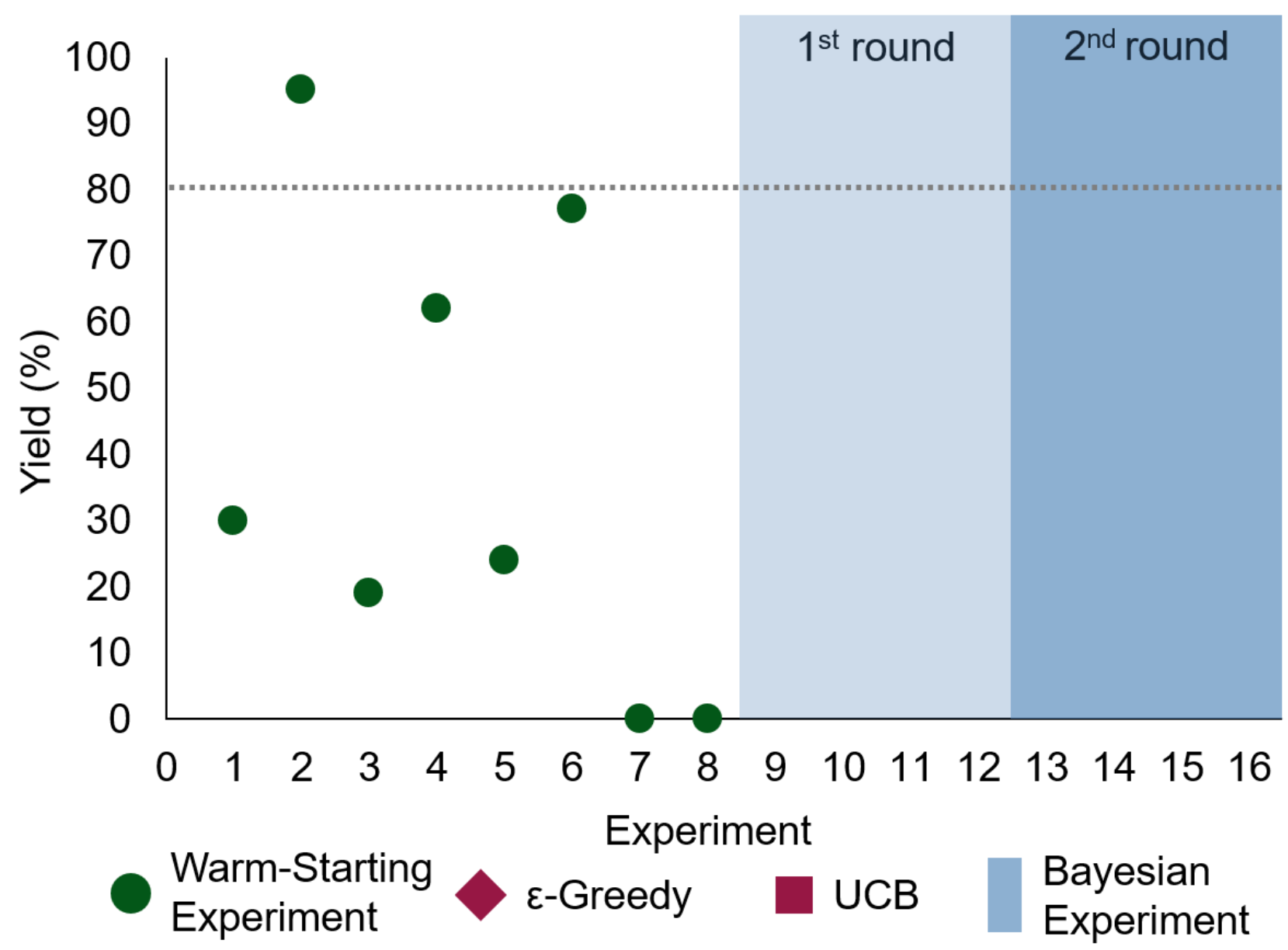

Figure S27

2u – Phenyl 1-thio-6-O-trityl-2-*O*-acetyl-β-ᴅ-galactopyranoside

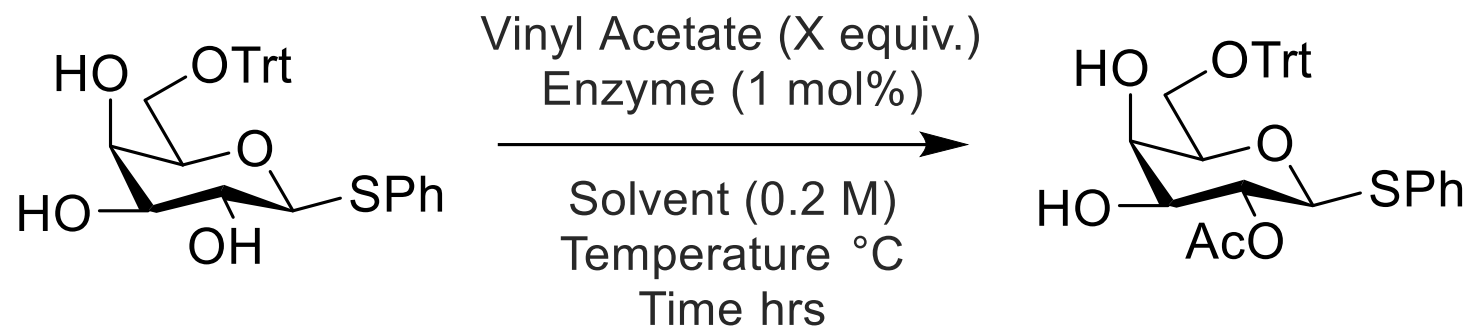

Experiment Type	Enzyme	Solvent	Acyl equiv.	Time (hrs)	Temp (°C)	Yield (%)
Top Conditions 1	PFL	THF/Py (4:1)	5	24	60	4
Top Conditions 2	PFL	THF	55	72	70	18
Top Conditions 2	PFL	THF/Py (4:1)	5	72	20	1
Top Conditions 3	PFL	THF/Py (4:1)	55	72	40	31
Top Conditions 3	PFL	THF/Py (4:1)	5	48	40	3
Top Conditions 3	PSL	THF/Py (4:1)	55	72	70	12
Top Conditions 3	PPL	THF	55	24	20	0
Top Conditions 4	PPL	THF/Py (4:1)	25	72	40	0

Average yield: 8% (compare to 25% for transfer learning)

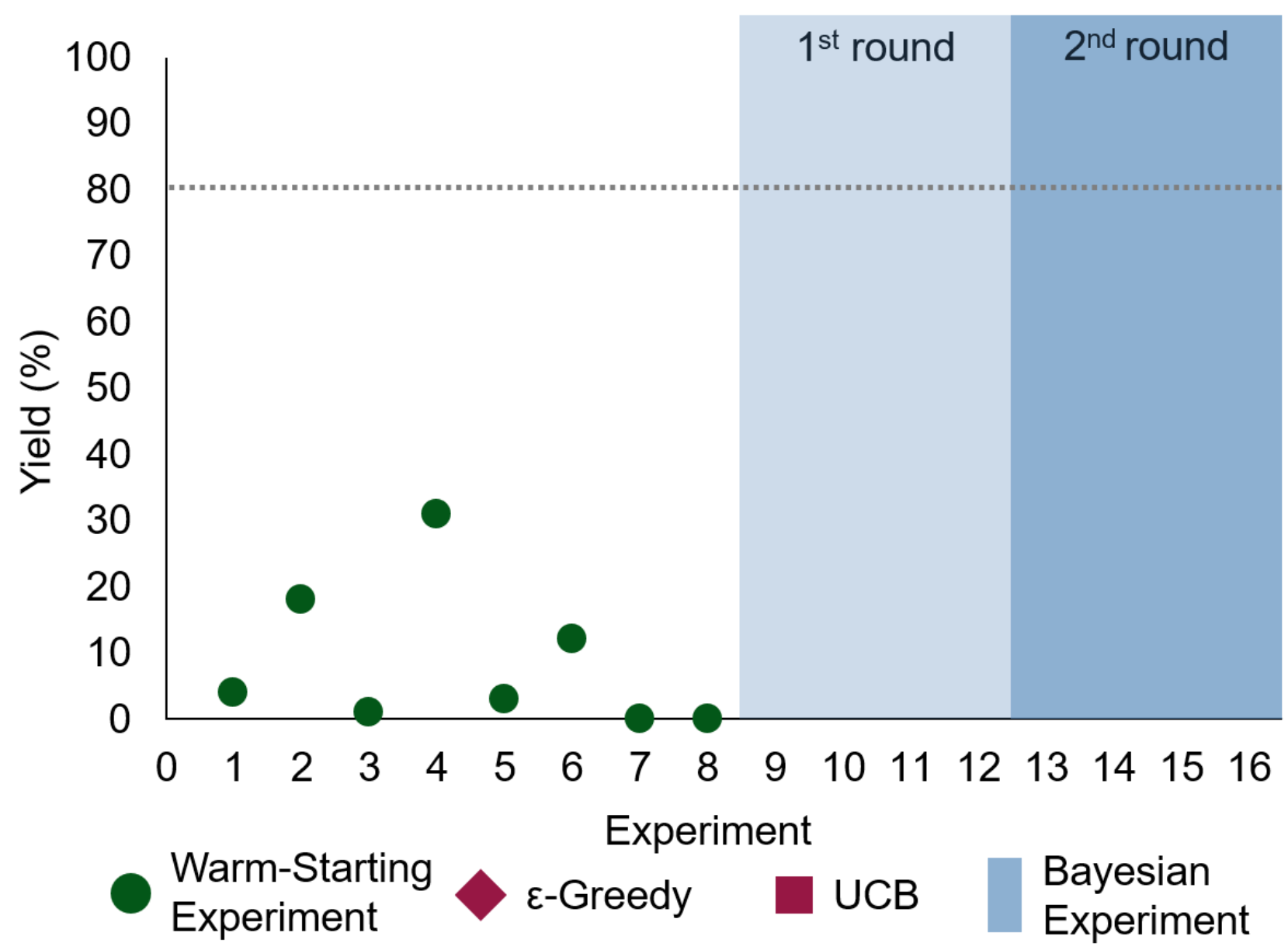

Figure S28

2v – Phenyl 6-*O*-acetyl-α-ᴅ-1-thio-mannopyranoside

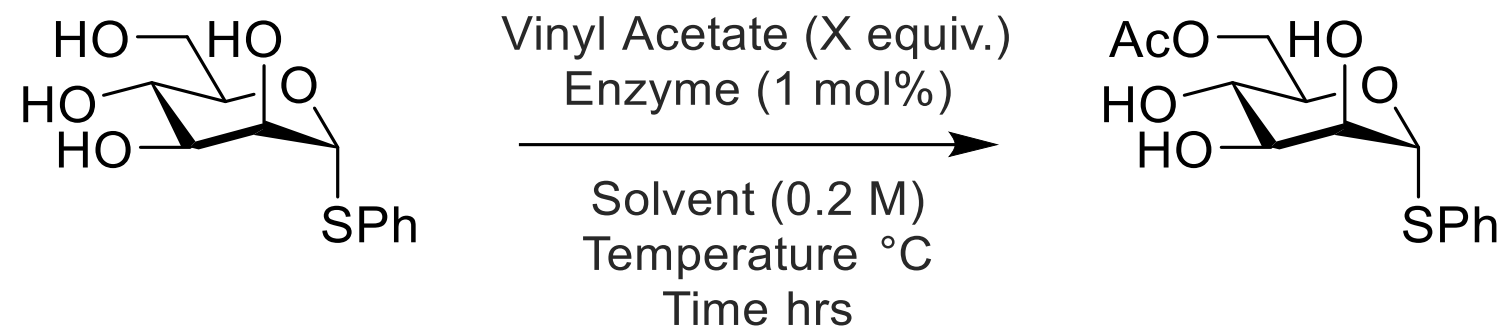

Experiment Type	Enzyme	Solvent	Acyl equiv.	Time (hrs)	Temp (°C)	Yield (%)
Top Conditions 1	PFL	THF/Py (4:1)	5	24	60	89
Top Conditions 2	PFL	THF	55	72	70	53
Top Conditions 2	PFL	THF/Py (4:1)	5	72	20	97
Top Conditions 3	PFL	THF/Py (4:1)	55	72	40	89
Top Conditions 3	PFL	THF/Py (4:1)	5	48	40	78
Top Conditions 3	PSL	THF/Py (4:1)	55	72	70	65
Top Conditions 3	PPL	THF	55	24	20	80
Top Conditions 4	PPL	THF/Py (4:1)	25	72	40	84

Average yield: 79% (compare to 55% for transfer learning)

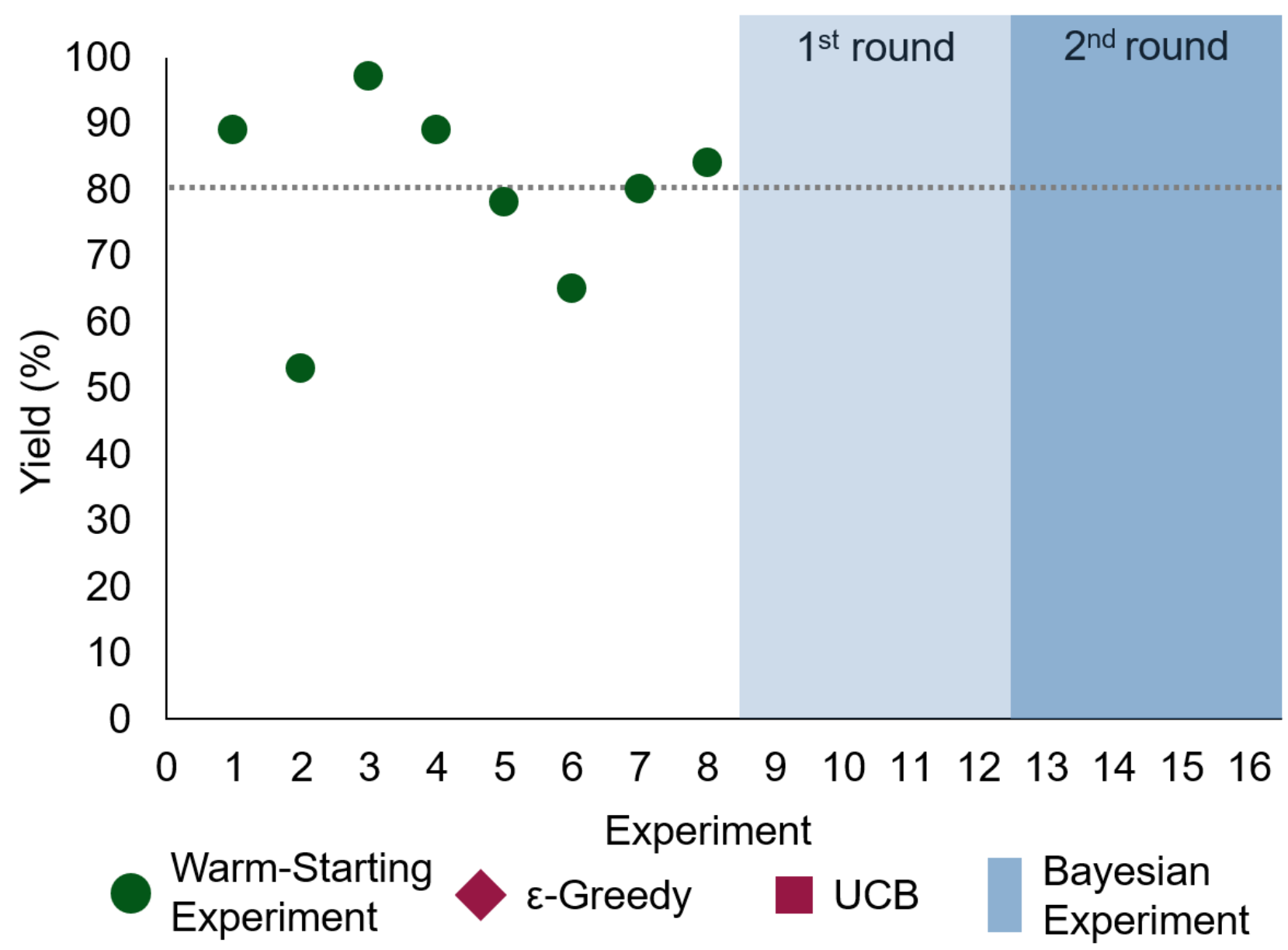

Figure S29

References

(1) Moitessier, N.; Englebienne, P.; Chapleur, Y. Directing-Protecting Groups for Carbohydrates. Design, Conformational Study, Synthesis and Application to Regioselective Functionalization. *Tetrahedron* **2005**, *61*, 6839–6853.
(2) Lee, D.; Taylor, M. S. Borinic Acid-Catalyzed Regioselective Acylation of Carbohydrate Derivatives. *J. Am. Chem. Soc.* **2011**, *133*, 3724–3727.
(3) Kalikanda, J.; Li, Z. Study of the Stereoselectivity of 2-Azido-2-Deoxygalactosyl Donors: Relationship to the Steric Factors of Glycosyl Acceptors. *Carbohydr. Res.* **2011**, *346*, 2380–2383.
(4) Dorgeret, B.; Khemtémourian, L.; Correia, I.; Soulier, J.-L.; Lequin, O.; Ongeri, S. Sugar-Based Peptidomimetics Inhibit Amyloid β-Peptide Aggregation. *Eur. J. Med. Chem.* **2011**, *46*, 5959–5969.
(5) Turner, J. A.; Rosano, N.; Gorelik, D. J.; Taylor, M. S. Synthesis of Ketodeoxysugars from Acylated Pyranosides Using Photoredox Catalysis and Hydrogen Atom Transfer. *ACS Catal.* **2021**, *11*, 11171–11179.
(6) Bashir, N. B.; Phythian, S. J.; Reason, A. J.; Roberts, S. M. Enzymatic esterification and de-esterification of carbohydrates: synthesis of a naturally occurring rhamnopyranoside of *p*-hydroxybenzaldehyde and a systematic investigation of lipase-catalyzed acylation of selected arylpyranosides. *J. Chem. Soc., Perkin Trans. 1* **1995**, 2203–2222.
(7) Ciuffreda, P.; Colombo, D.; Ronchetti, F.; Toma, L. Regioselective Acylation of 6-Deoxy-L- and -D-Hexosides through Lipase-Catalyzed Transesterification. Enhanced Reactivity of the 4-Hydroxy Function in the L Series. *J. Org. Chem.* **1990**, *55*, 4187–4190.
(8) Boissière-Junot, N.; Tellier, C.; Rabiller, C. On the Regioselective Acylation of 1,6-Anhydro-β-D- and L-Hexopyranoses Catalyzed by Lipases: Structural Basis and Synthetic Applications. *J. Carbohydr. Chem.* **1998**, *17*, 99–115.
(9) Oosterom, M. W.; Vitry, C.; Baas, J. M. A.; van Rantwijk, F.; Sheldon, R. A. The Regioselectivity of the Lipase-Catalyzed Acylation of 1,6-Anhydro-β-D-Glycopyranoses. *J. Carbohydr. Chem.* **1995**, *14*, 237–246.
(10) Panza, L.; Luisetti, M.; Crociati, E.; Riva, S. Selective Acylation of 4,6-*O*-Benzylidene Glycopyranosides by Enzymatic Catalysis. *J. Carbohydr. Chem.* **1993**, *12*, 125–130.
(11) Chinn, M.; Iacazio, G.; Spackman, D.; Turner, N.; Roberts, S. Regioselective Enzymatic Acetylation of Methyl 4,6-O-Benzylidene-α- and β-D-Glucopyranoside. *J. Chem. Soc., Perkin Trans. 1* **1992**, 661–662.
(12) Iacazio, G.; Roberts, S. M. Investigation of the Regioselectivity of Some Esterifications Involving Methyl 4,6-*O*-Benzylidene D-Pyranosides and *Pseudomonas fluorescens* Lipase. *J. Chem. Soc., Perkin Trans. 1* **1993**, 1099–1101.
(13) Holmstrøm, T.; Pedersen, C. M. Enzyme-Catalyzed Regioselective Acetylation of Functionalized Glycosides. *Eur. J. Org. Chem.* **2020**, 4612–4615.
(14) Shim, E.; Tewari, A.; Cernak, T.; Zimmerman, P. M. Recommending reaction conditions with label ranking. *Chem. Sci*. **2025**, *16*, 4109–4118.

^{1}H, ^{13}C and 2D NMR Spectra

Methyl 2-*O*-acetyl-6-*O*-(tert-butyldimethylsilyl)-α-ᴅ-glucopyranoside (2a)

^{1}H NMR (400 MHz, $CDCl_3$)

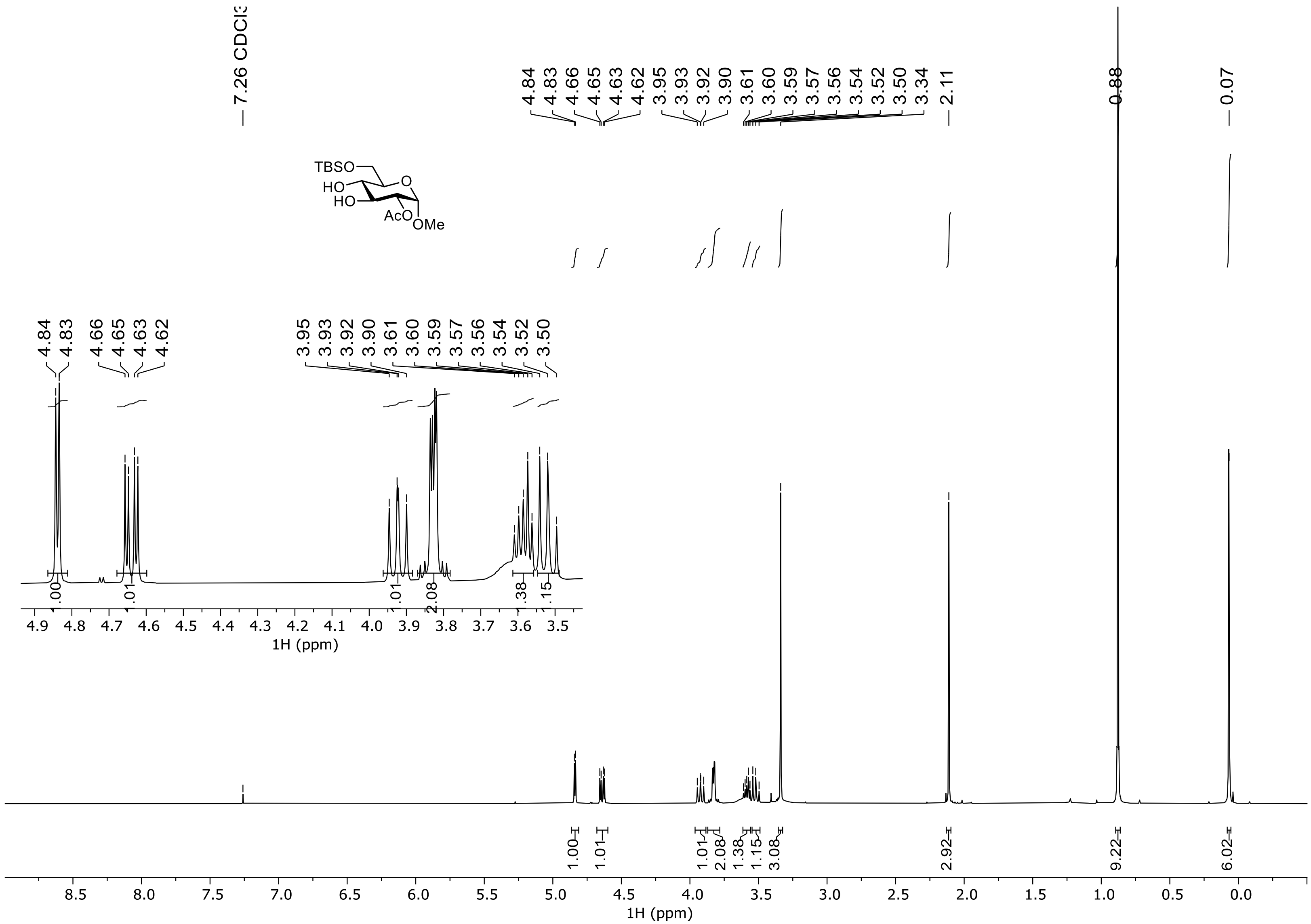

^{13}C NMR (101MHz, $CDCl_3$)

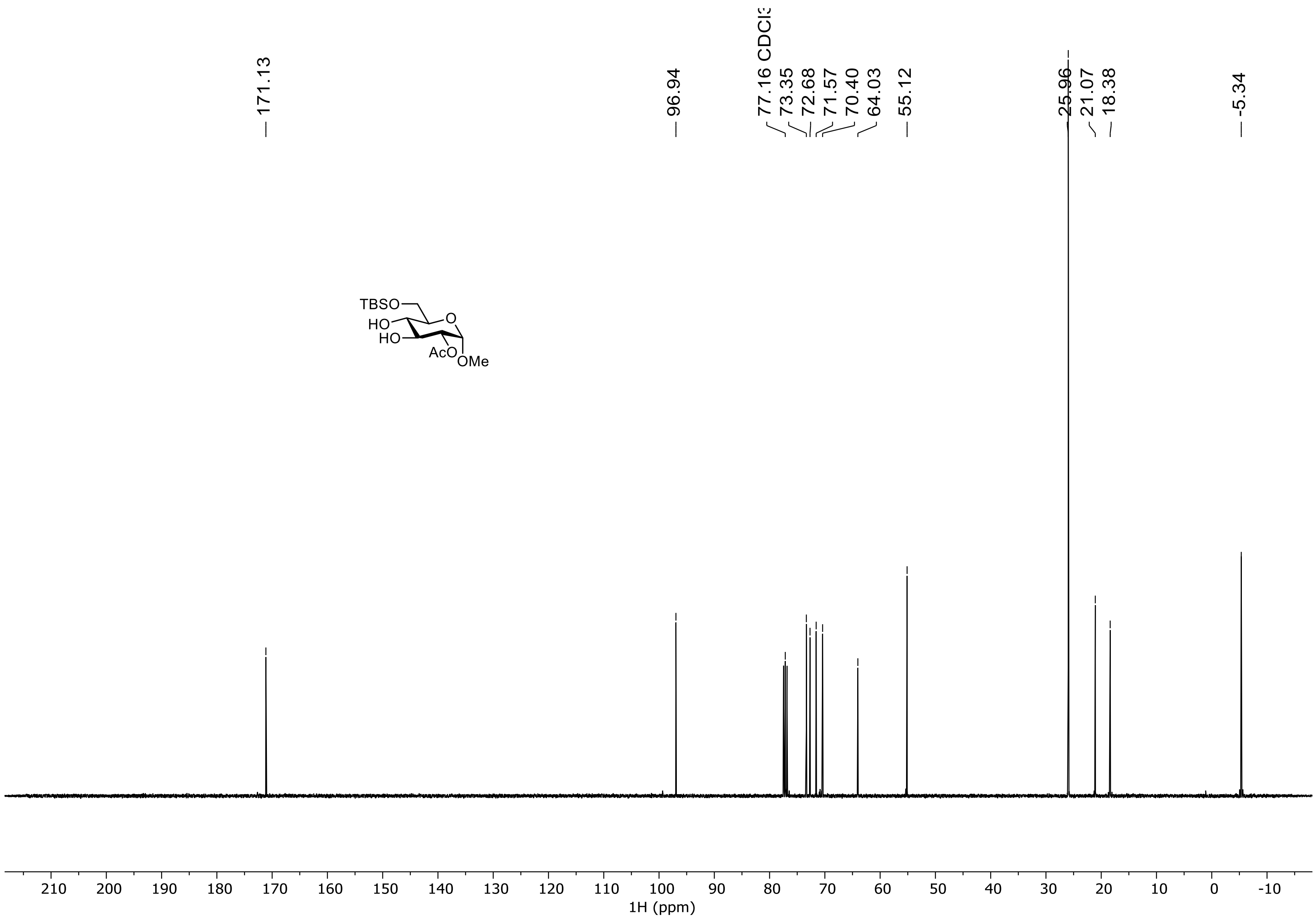

COSY (400 MHz, $CDCl_3$)

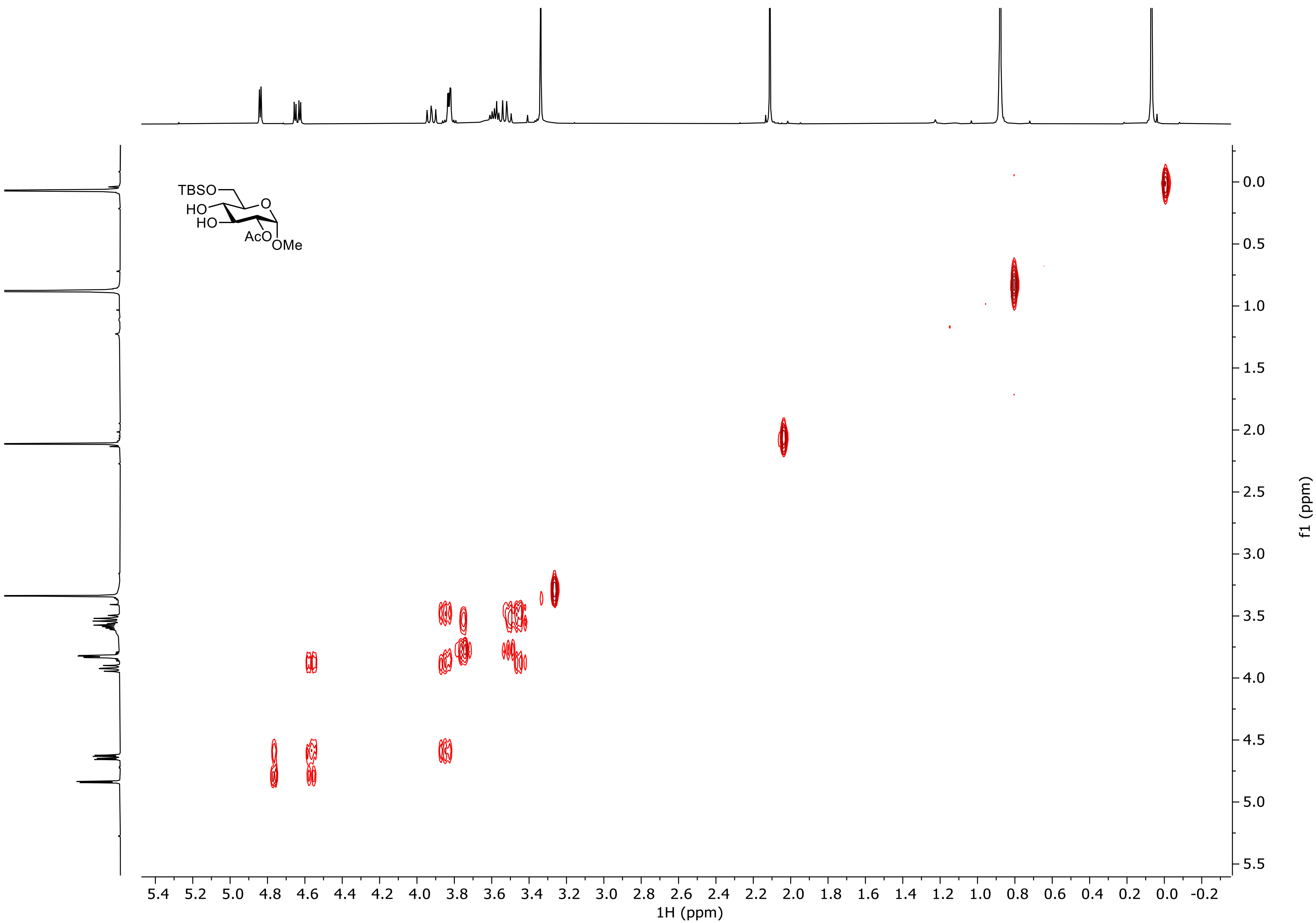

Methyl 2-*O*-acetyl-6-*O*-(tert-butyldimethylsilyl)-α-ᴅ-galactopyranoside (2c)

^{1}H NMR (400 MHz, $CDCl_3$)

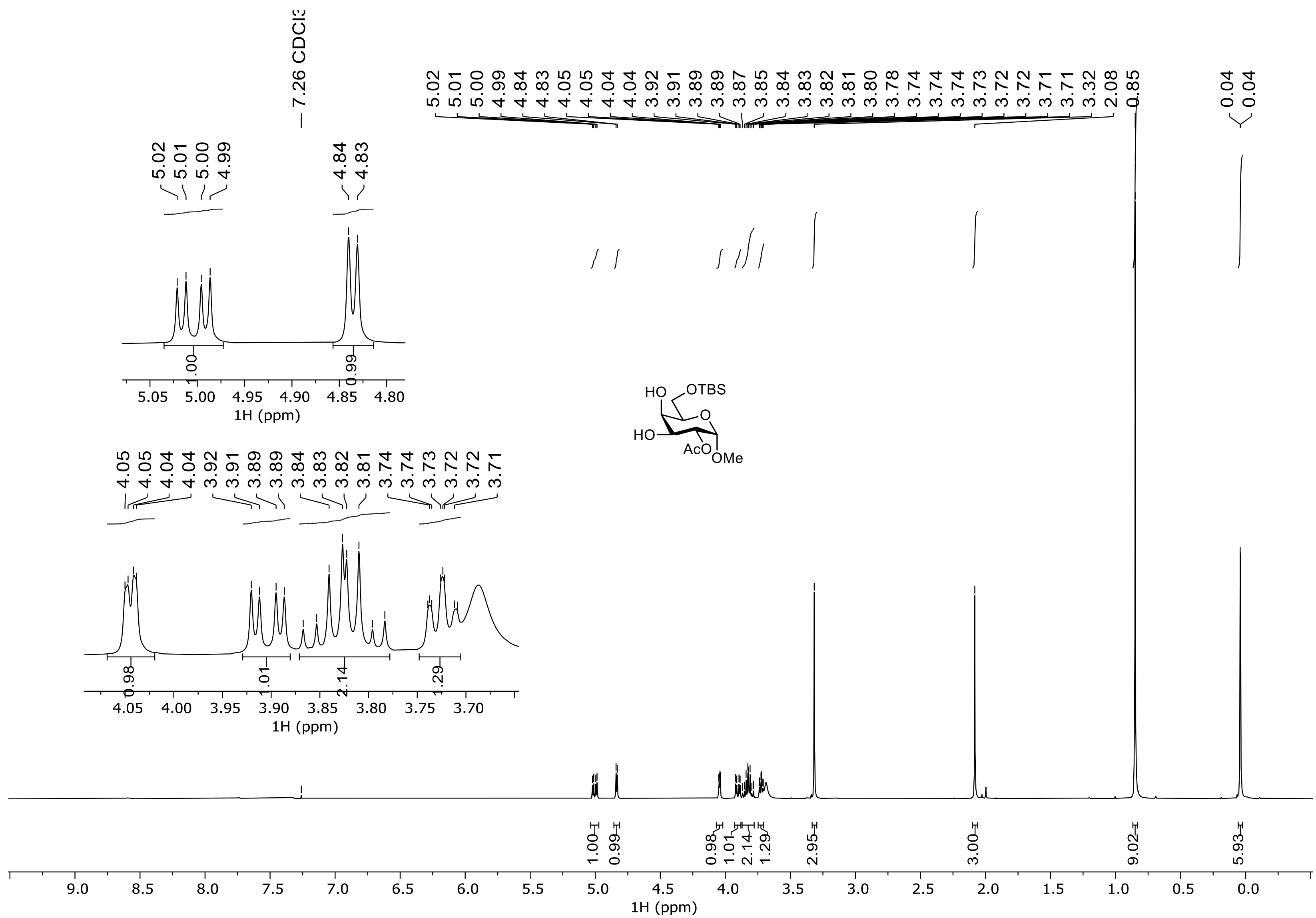

^{13}C NMR (101MHz, $CDCl_3$)

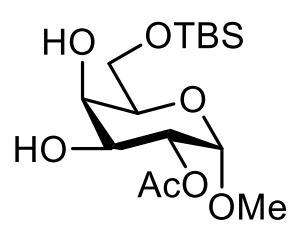

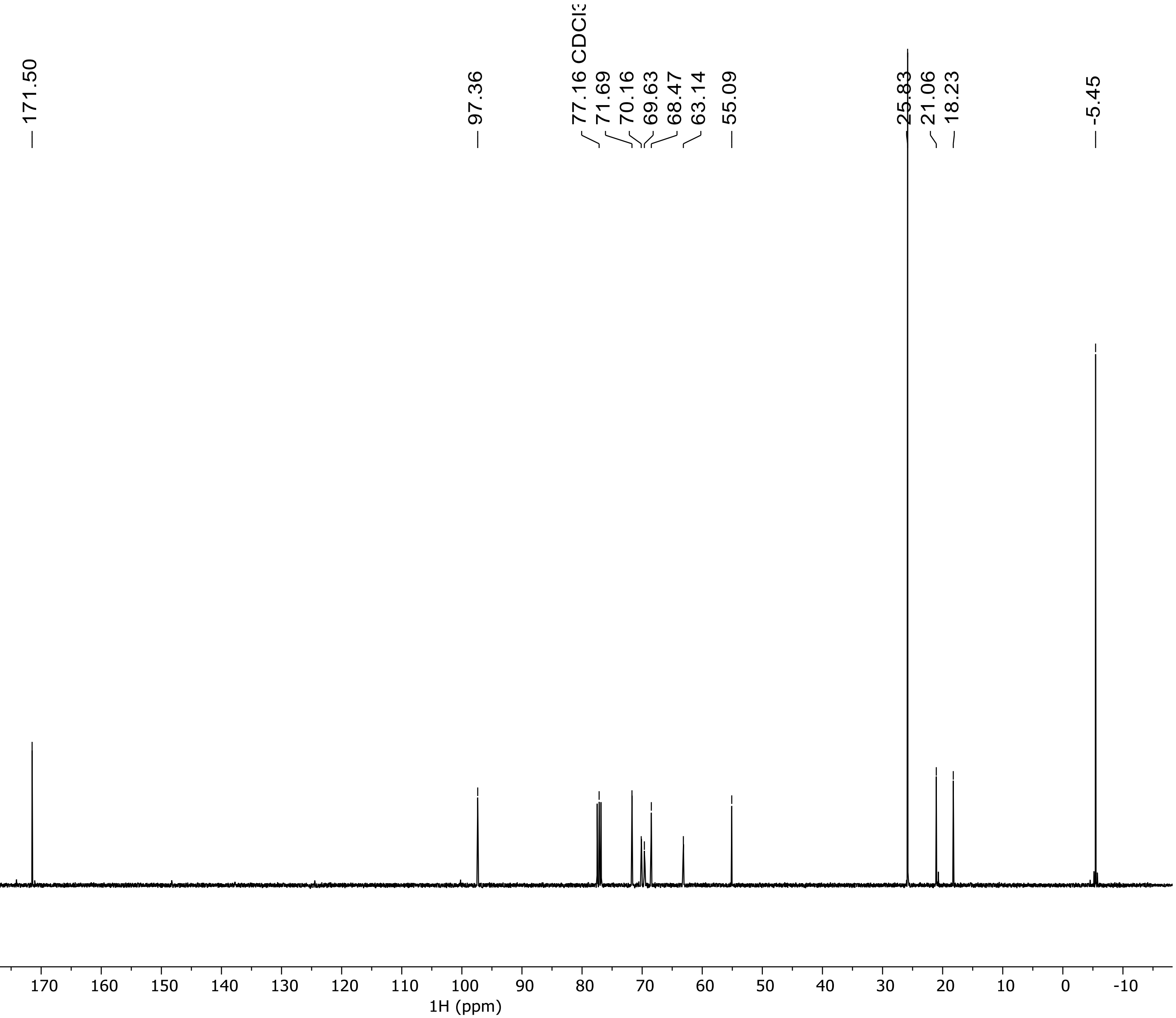

COSY (400 MHz, $CDCl_3$)

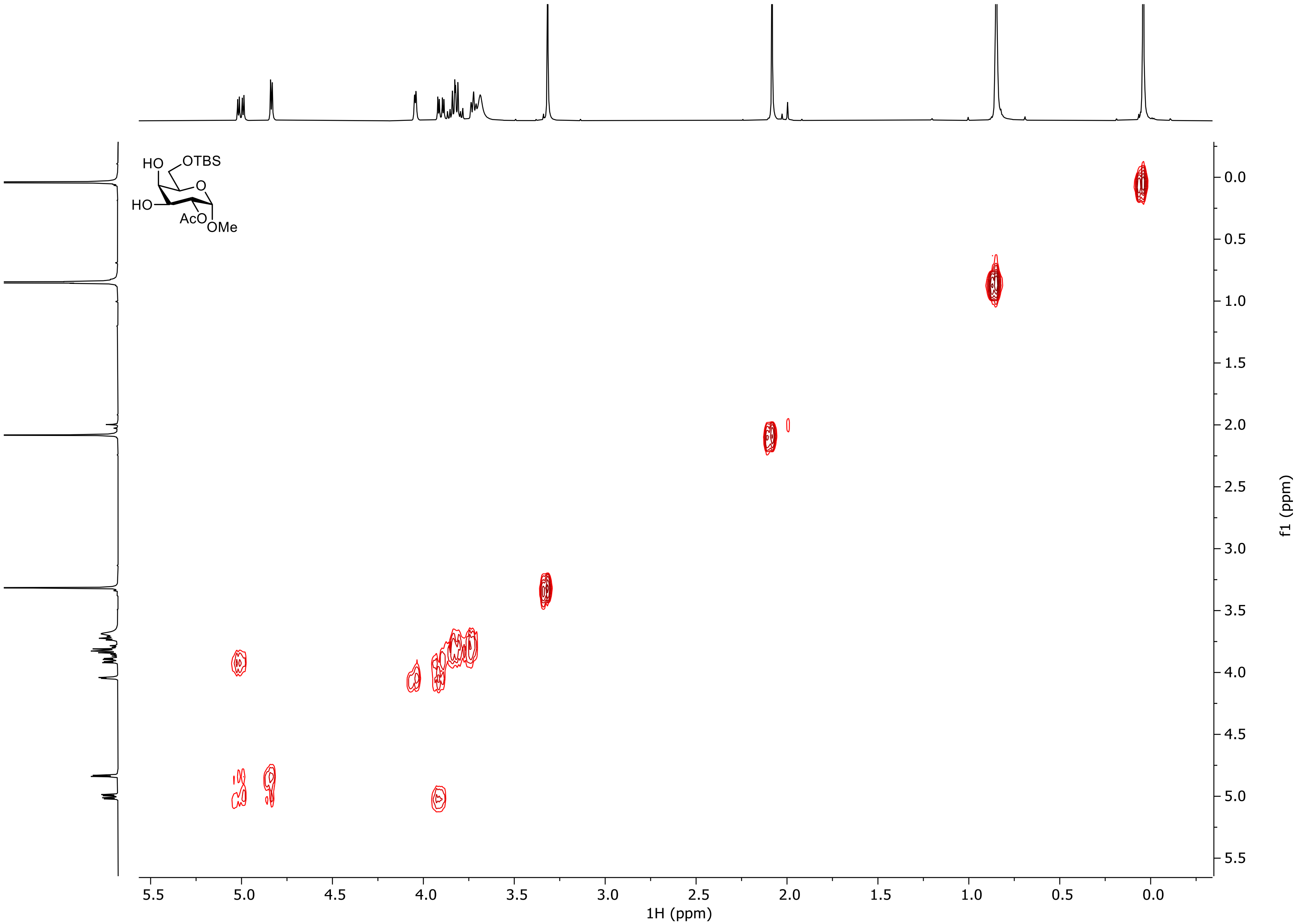

n-Octyl 2-*O*-acetyl-6-*O*-(*tert*-butyldimethylsilyl)-α-D-galactopyranoside (2d)

^{1}H NMR (400 MHz, $CDCl_3$)

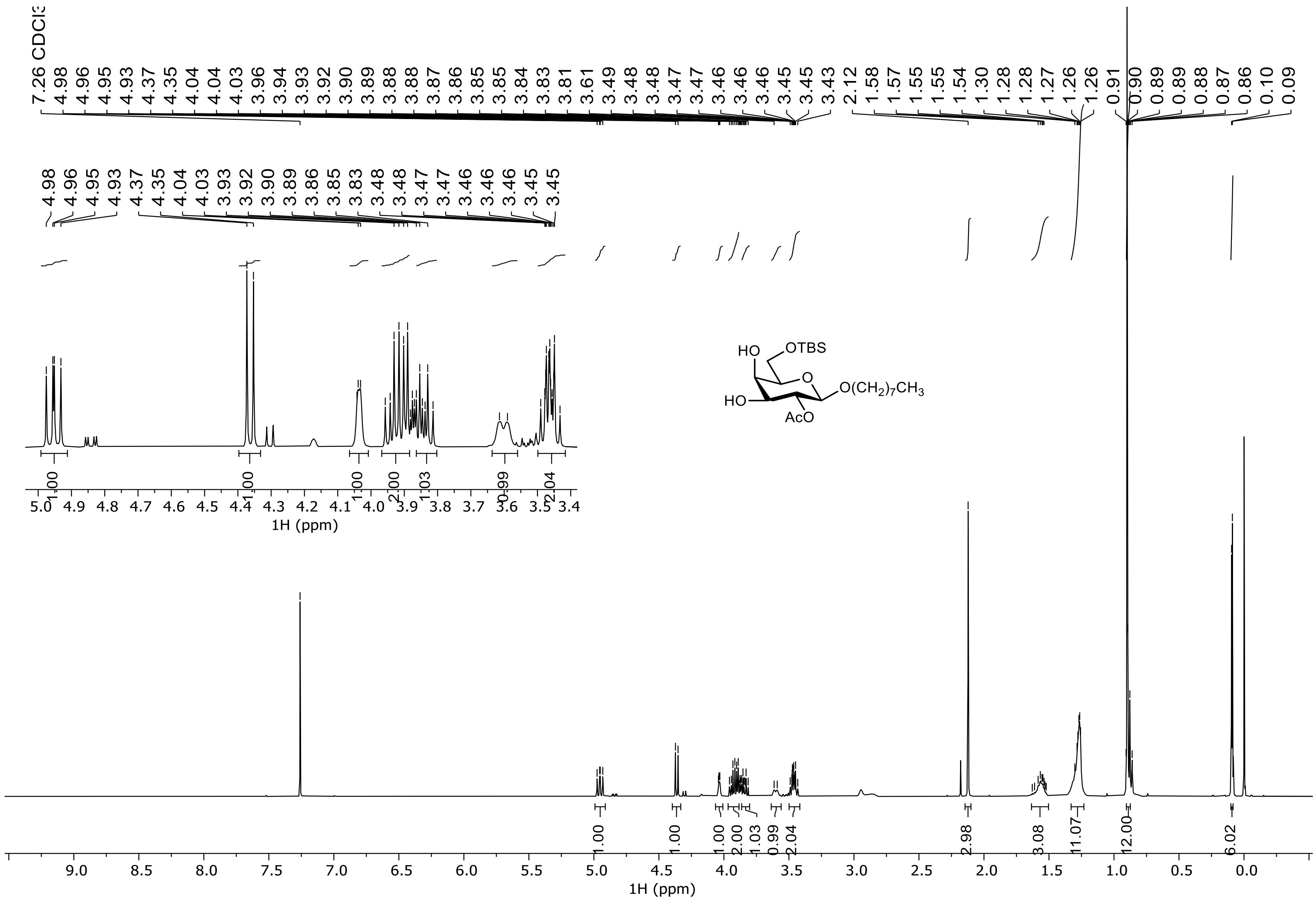

^{13}C NMR (101 MHz, $CDCl_3$)

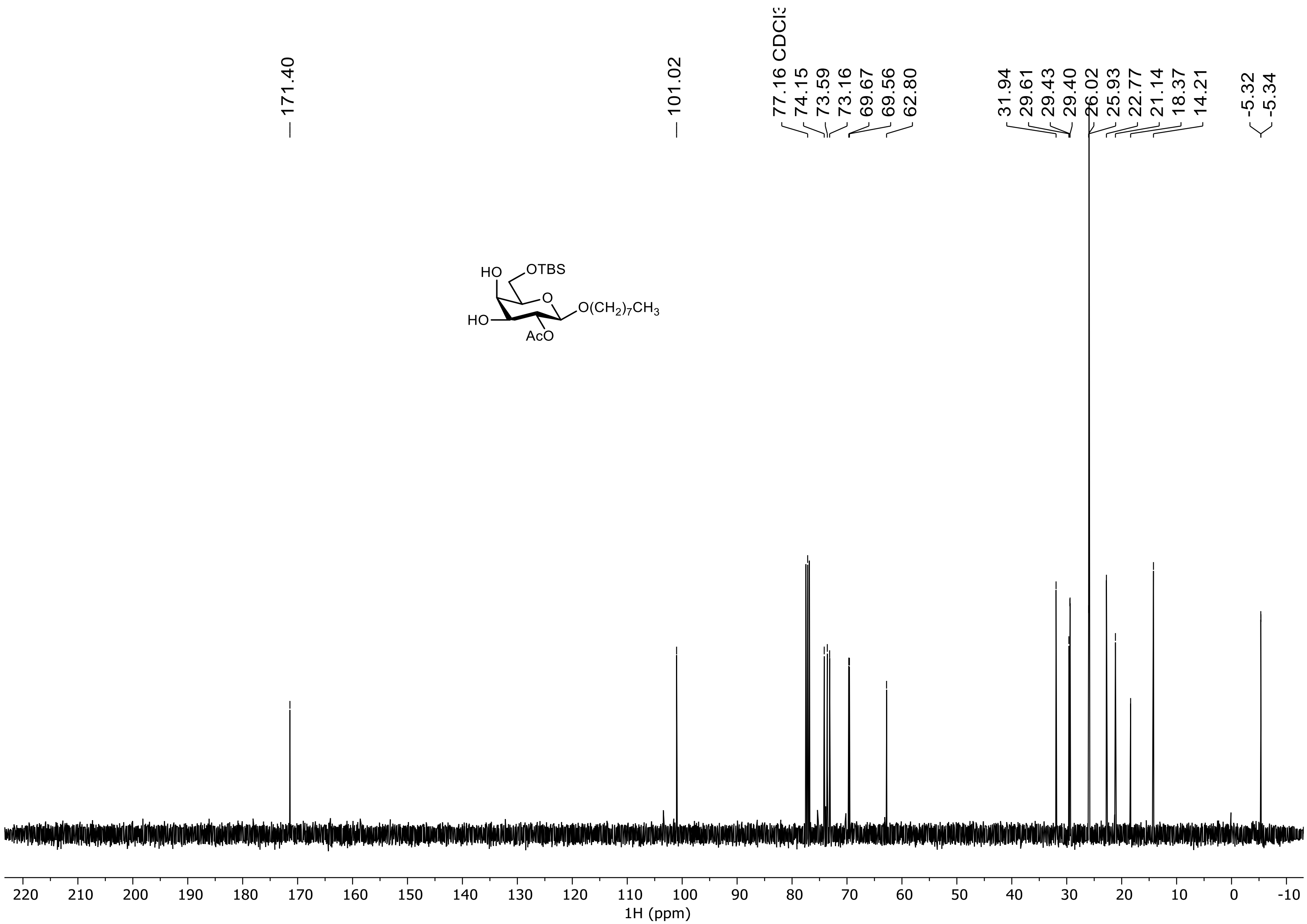

COSY (400 MHz, $CDCl_3$)

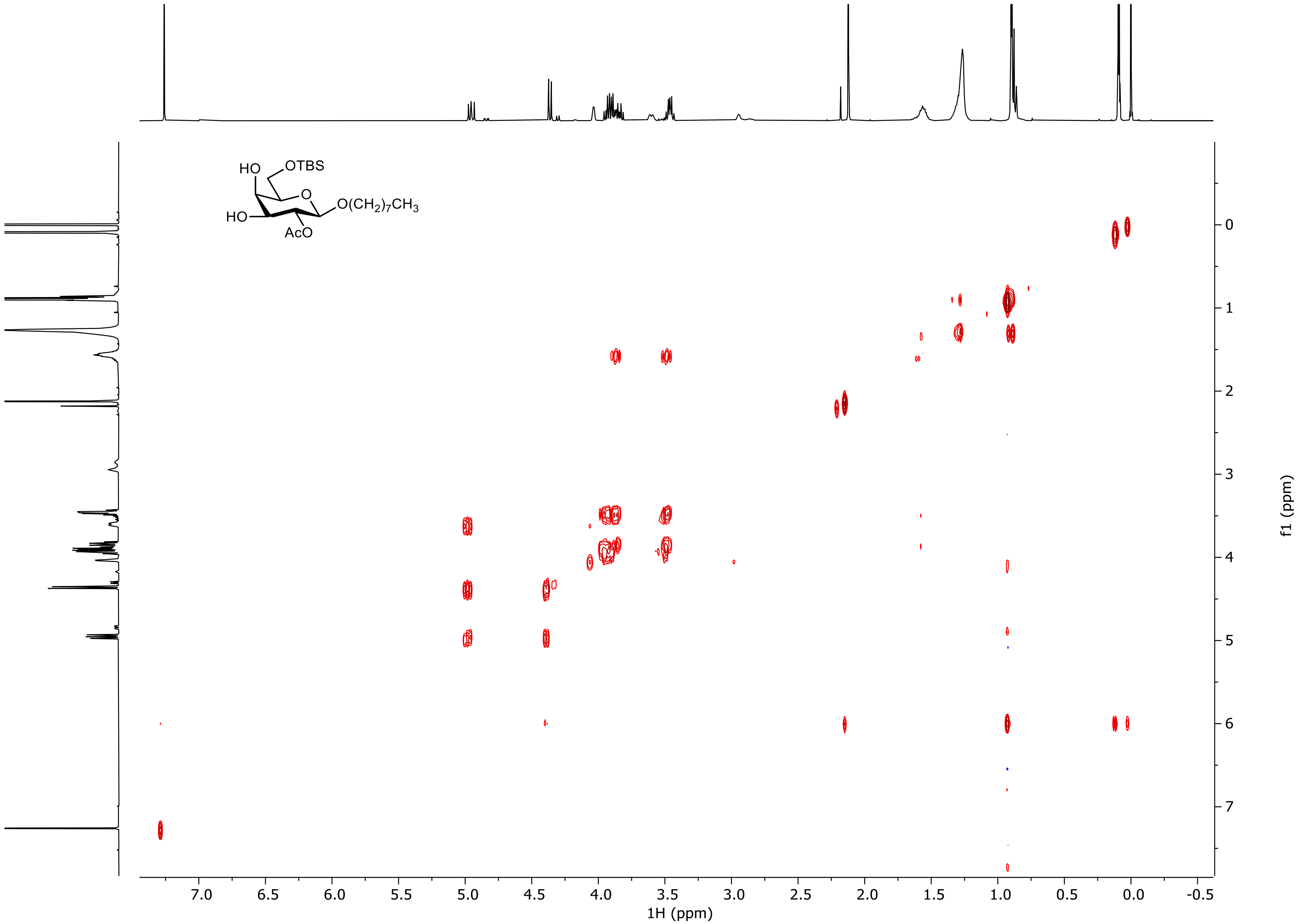

4-*O*-acetyl 1,6-β-ᴅ-anhydroglucose (2f)

^{1}H NMR (400 MHz, DMSO-d6)

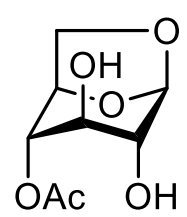

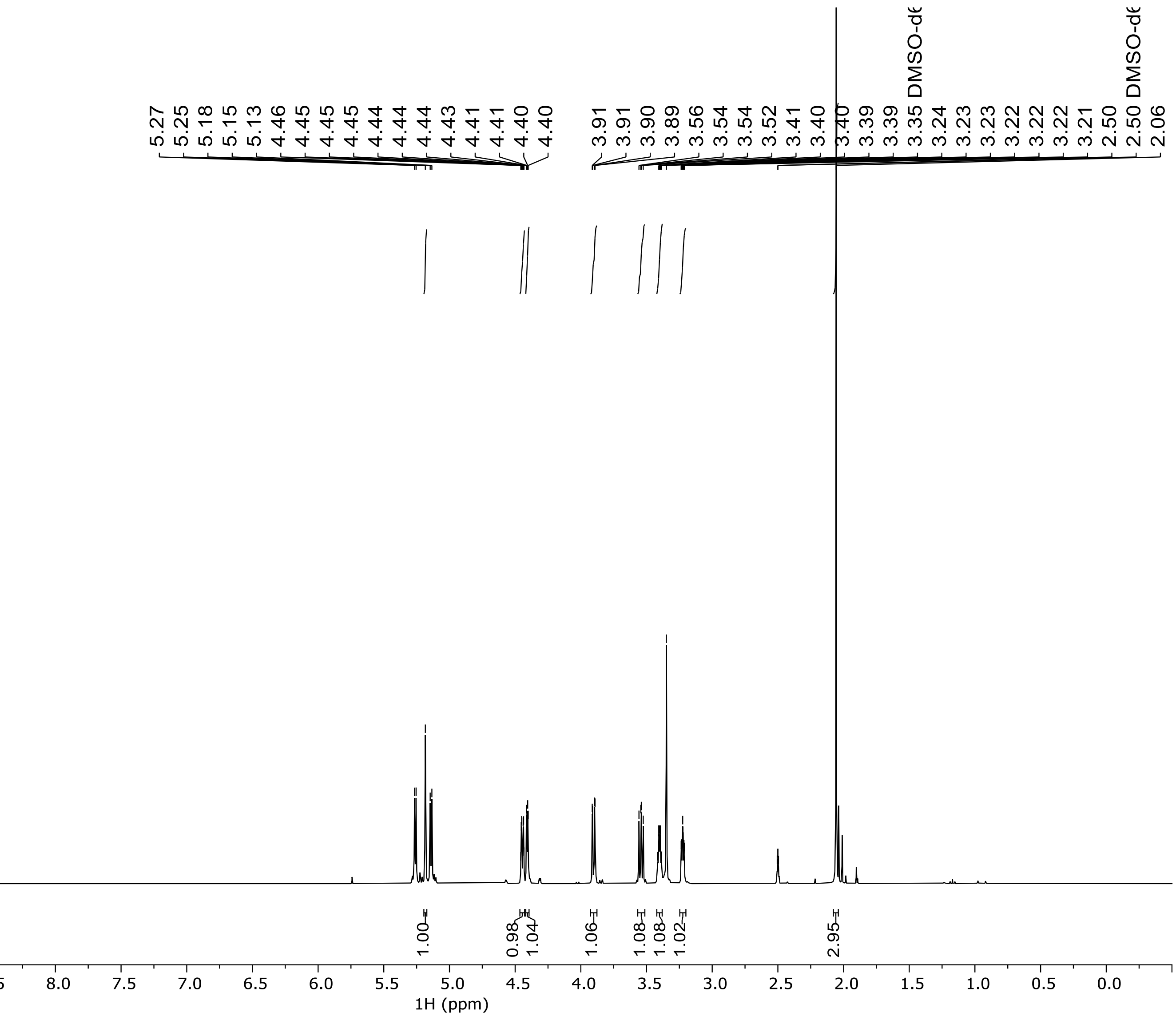

^{13}C NMR (101MHz, DMSO-d6)

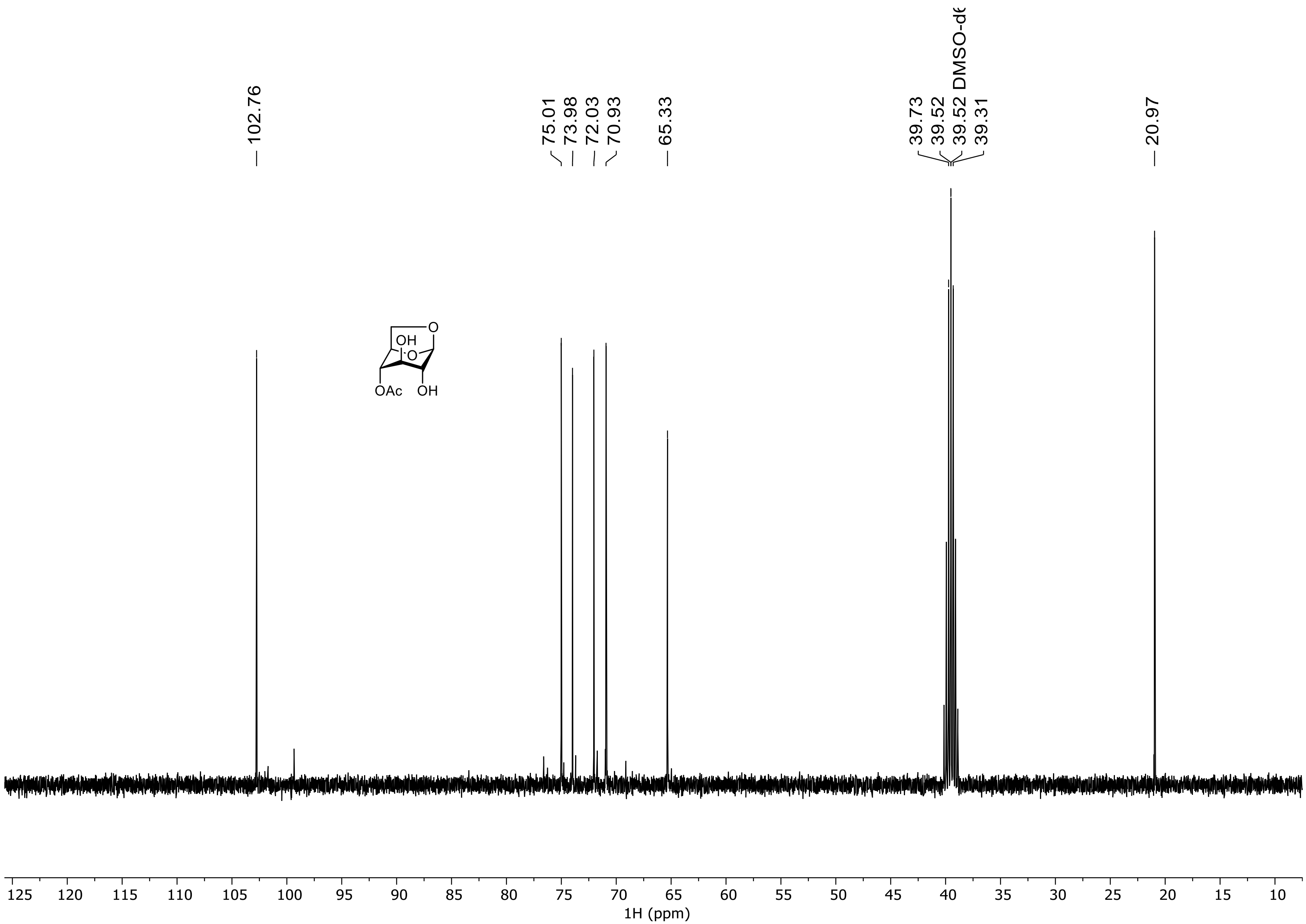

COSY (400 MHz, DMSO-d6)

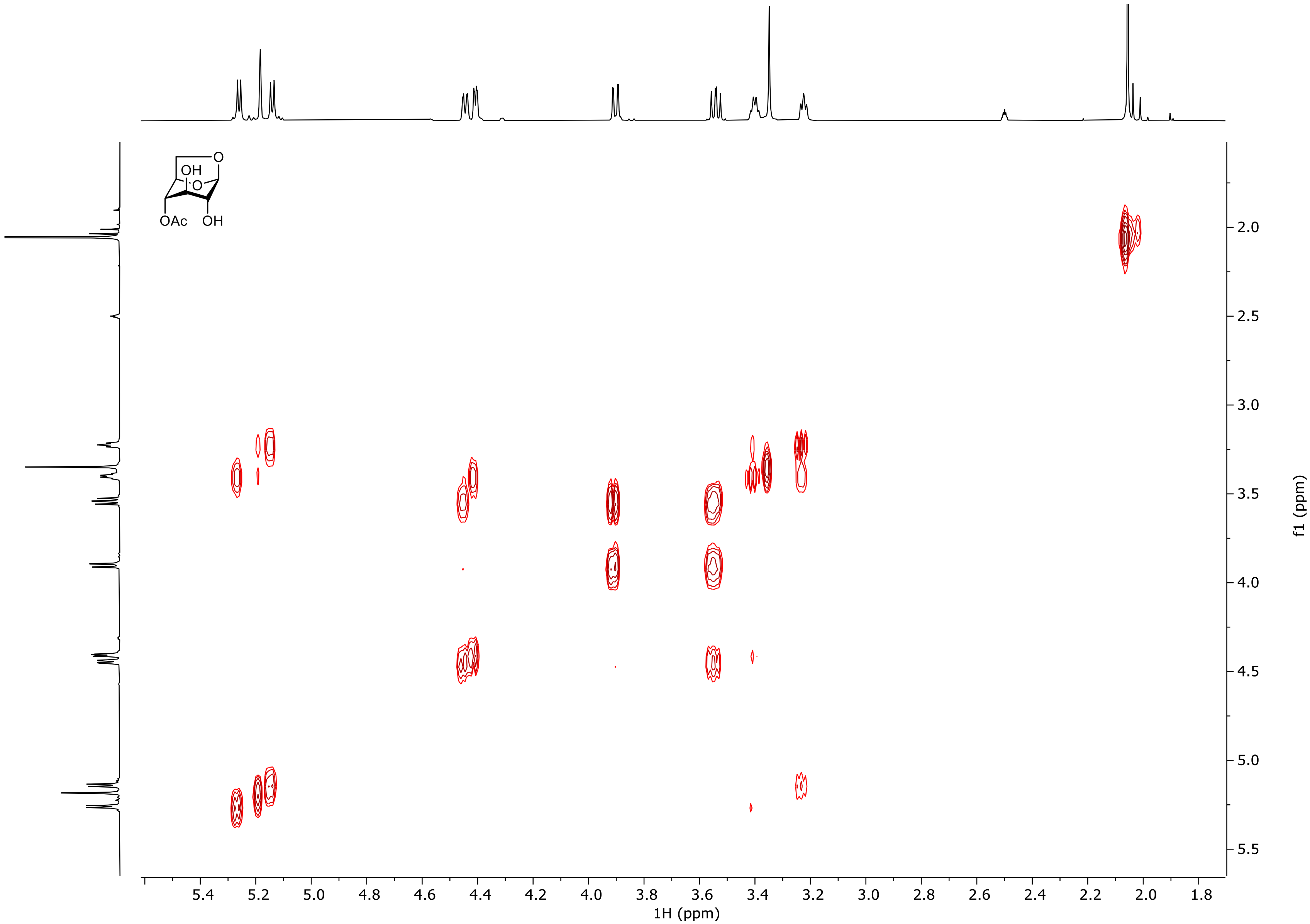

2,4-di-*O*-acetyl 1,6-β-ᴅ-anhydroglucose (2f')

^{1}H NMR (400 MHz, DMSO-d6)

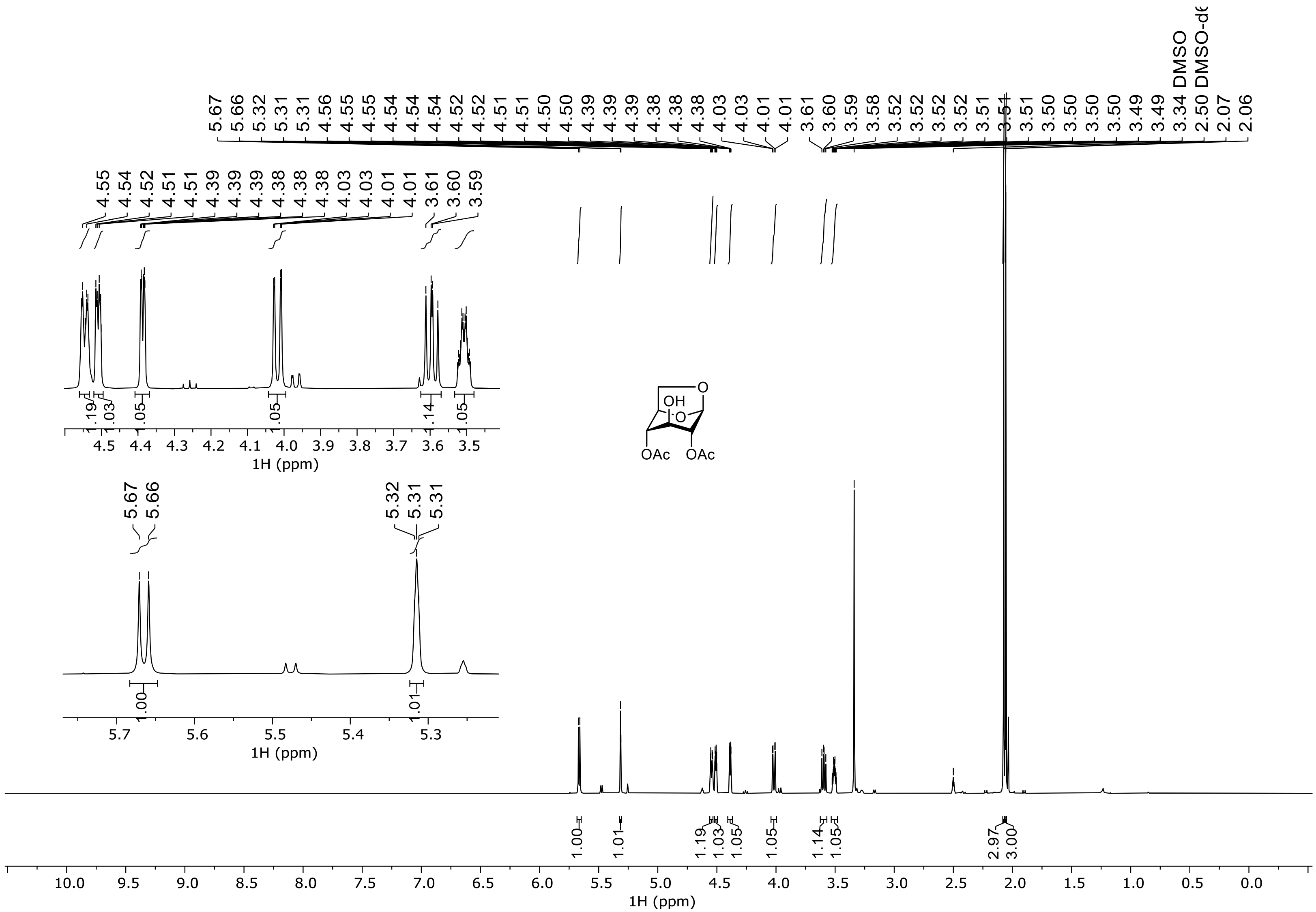

^{13}C NMR (101MHz, DMSO-d6)

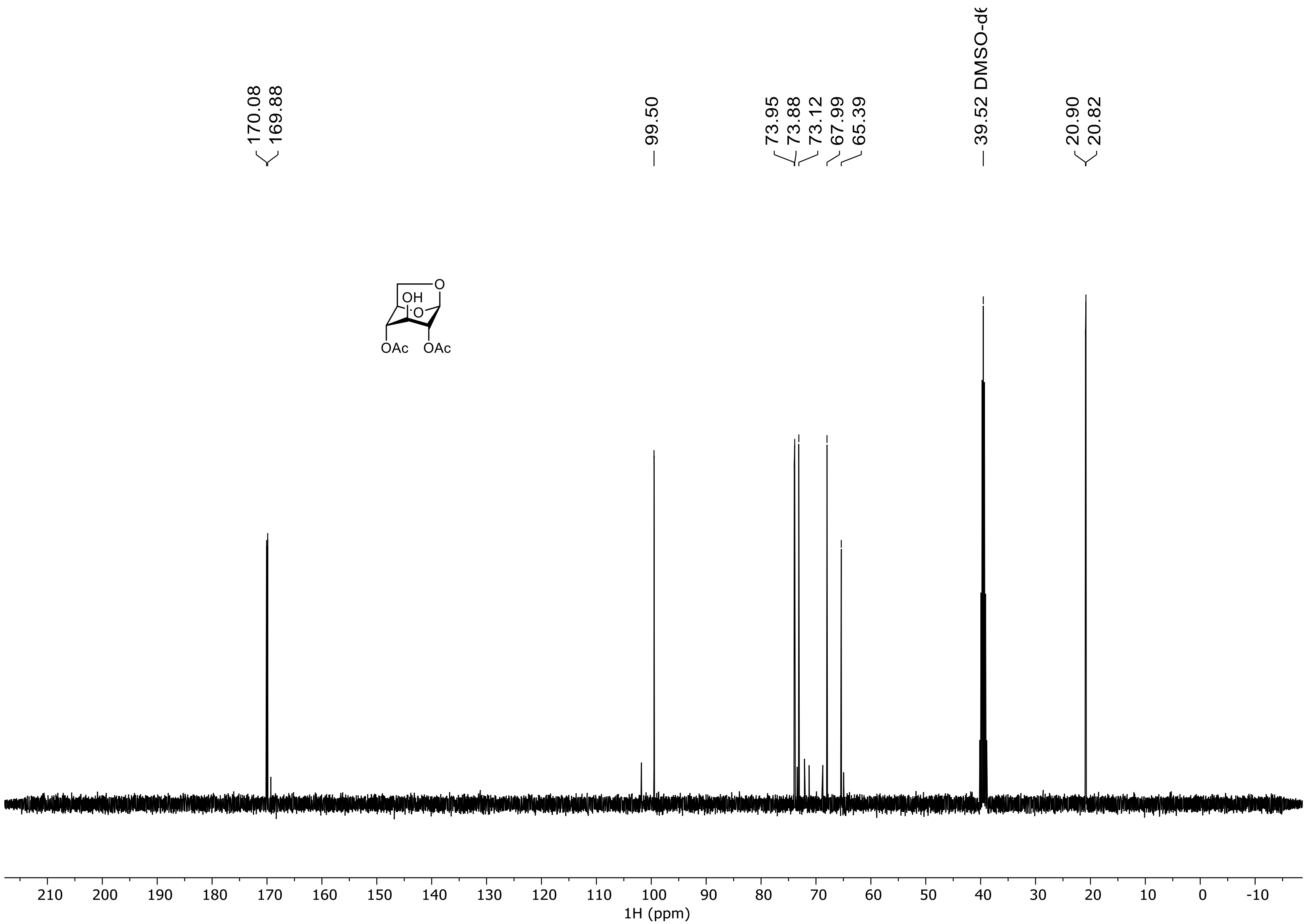

COSY (400 MHz, DMSO-d6)

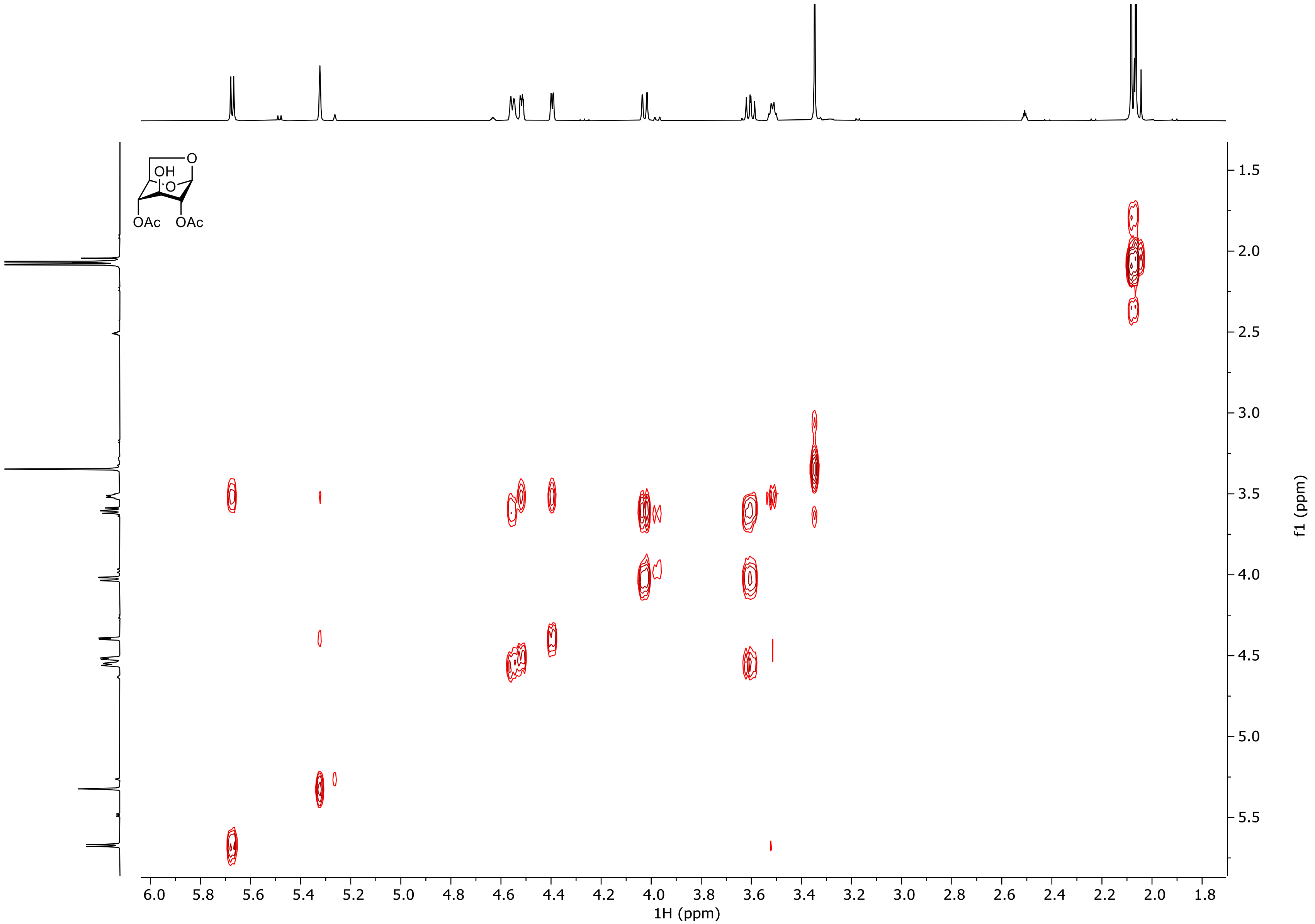

Methyl 2-*O*-acetyl-4,6-benzylidene α-ᴅ-glucopyranoside (2g)

^{1}H NMR (400 MHz, $CDCl_3$)

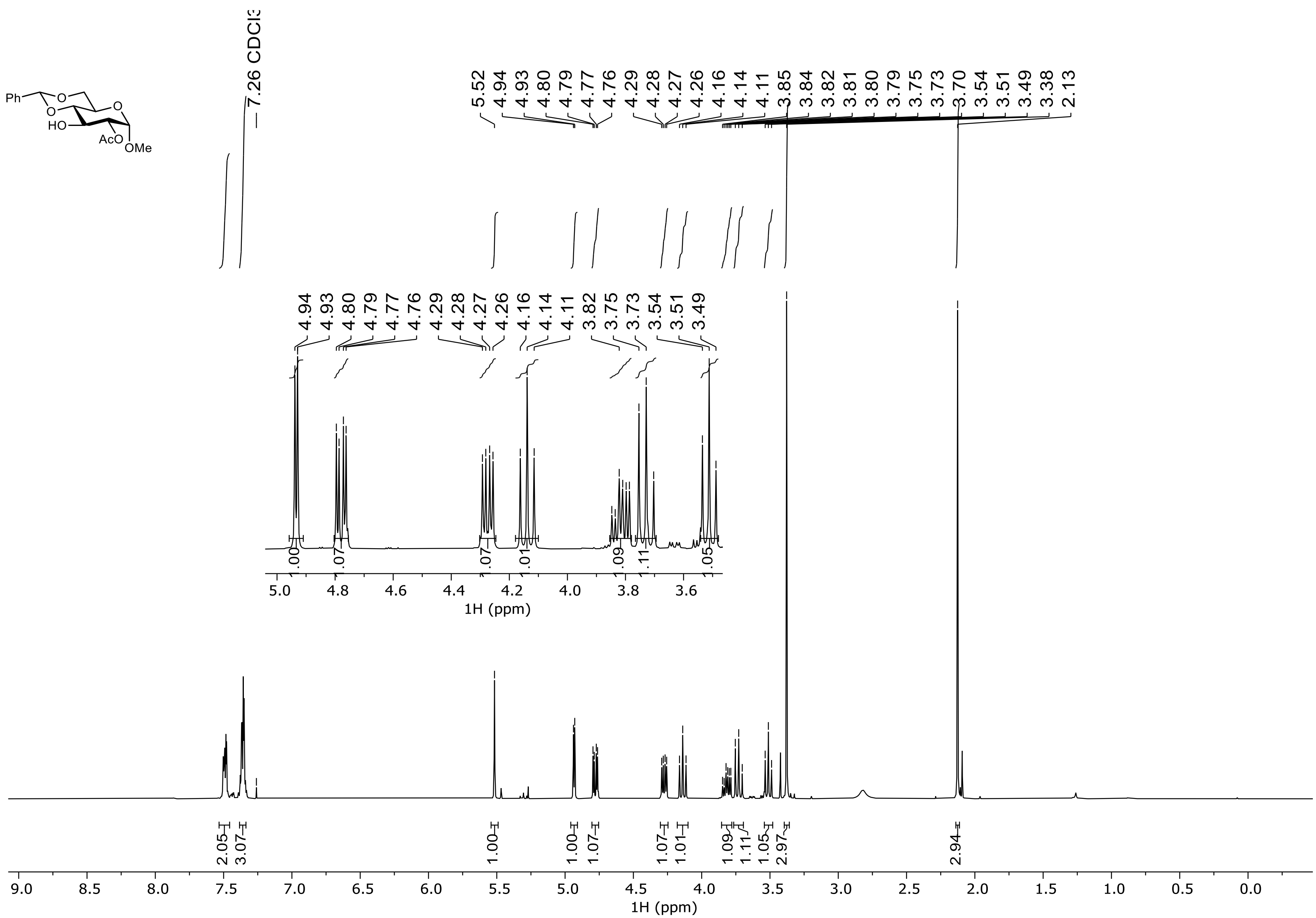

^{13}C NMR (101MHz, CDCl$_3$)

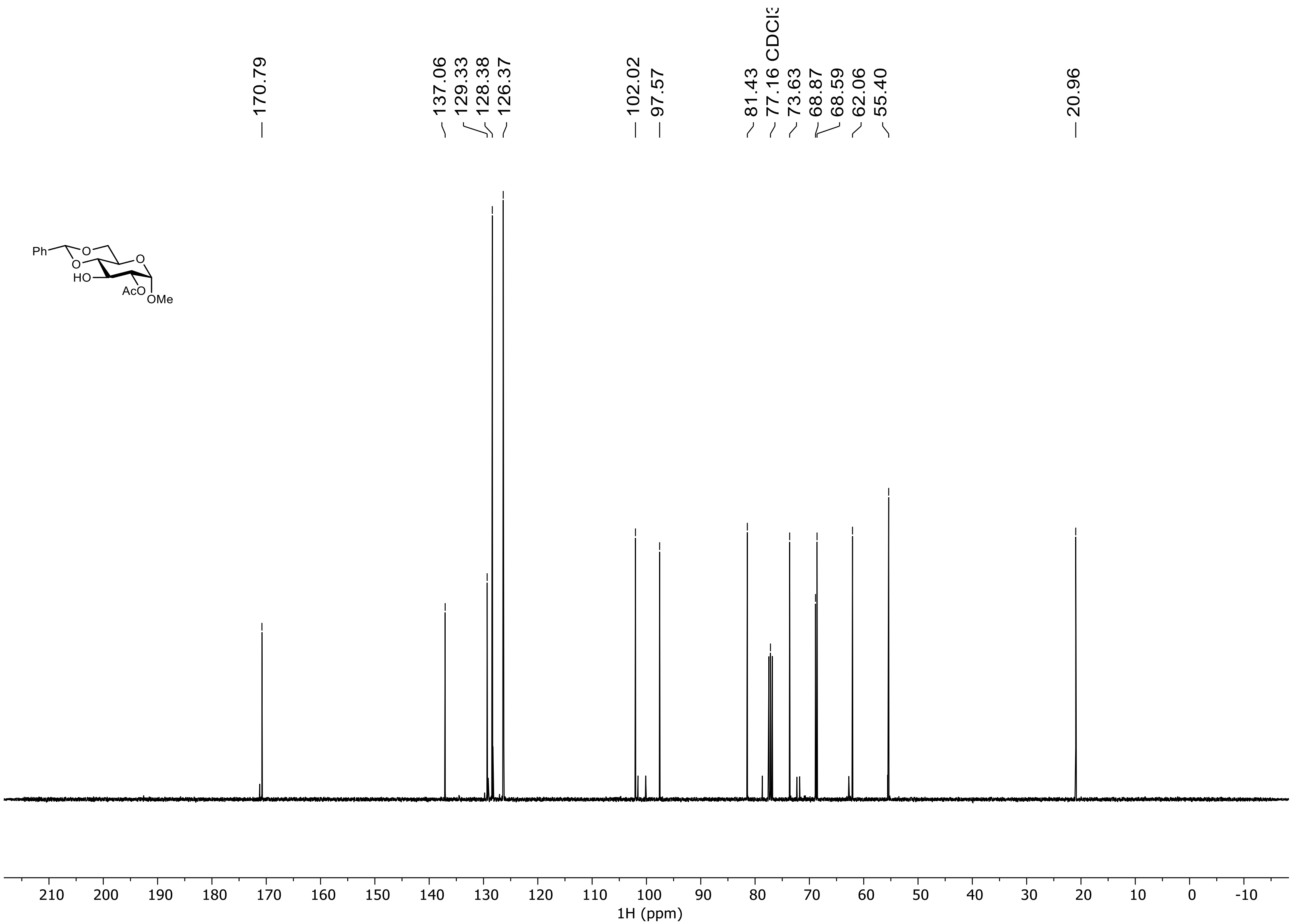

COSY (400 MHz, $CDCl_3$)

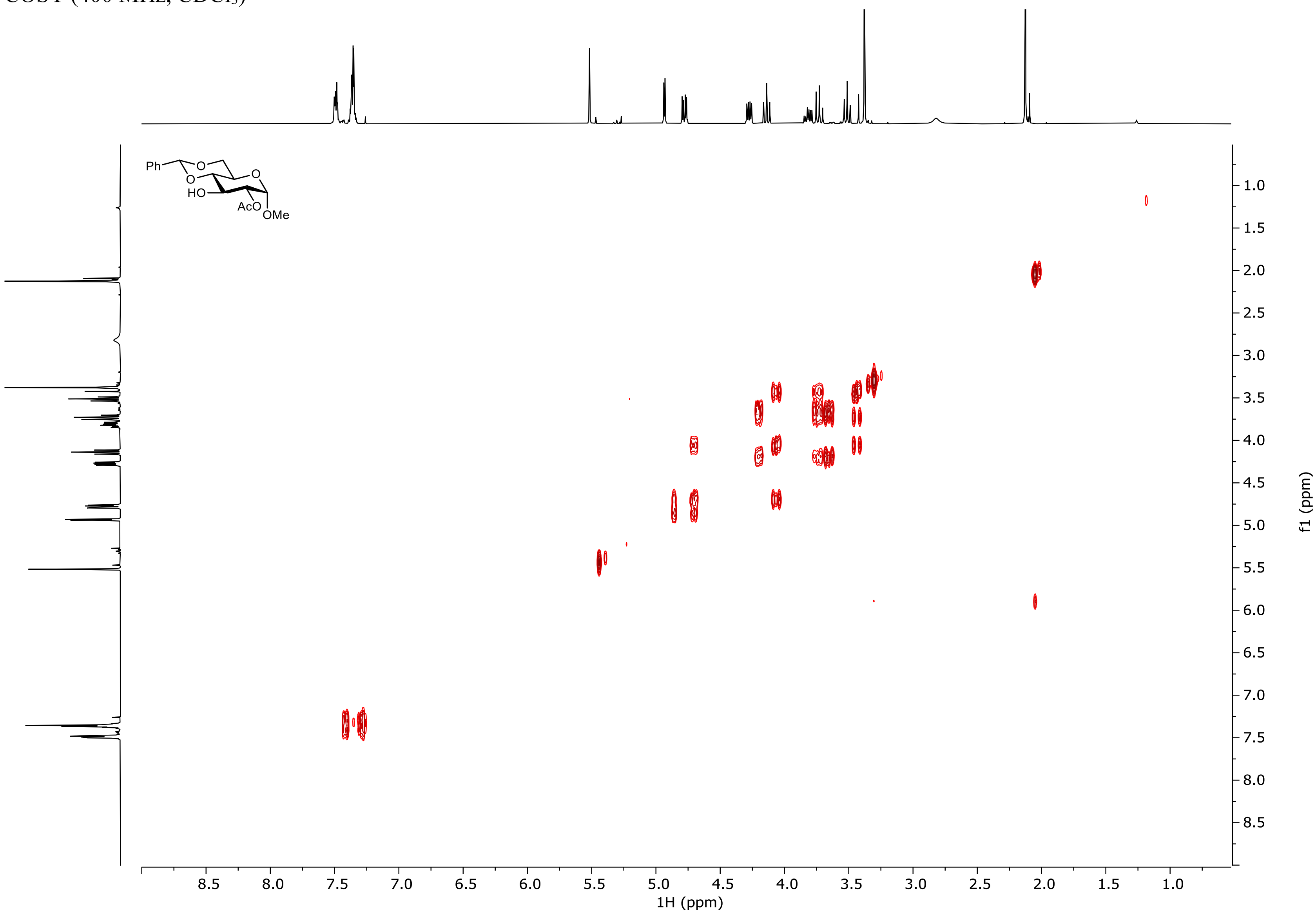

Methyl 2-*O*-acetyl-α-D-xylopyranoside (2i)

^{1}H NMR (400 MHz, CD_3OD)

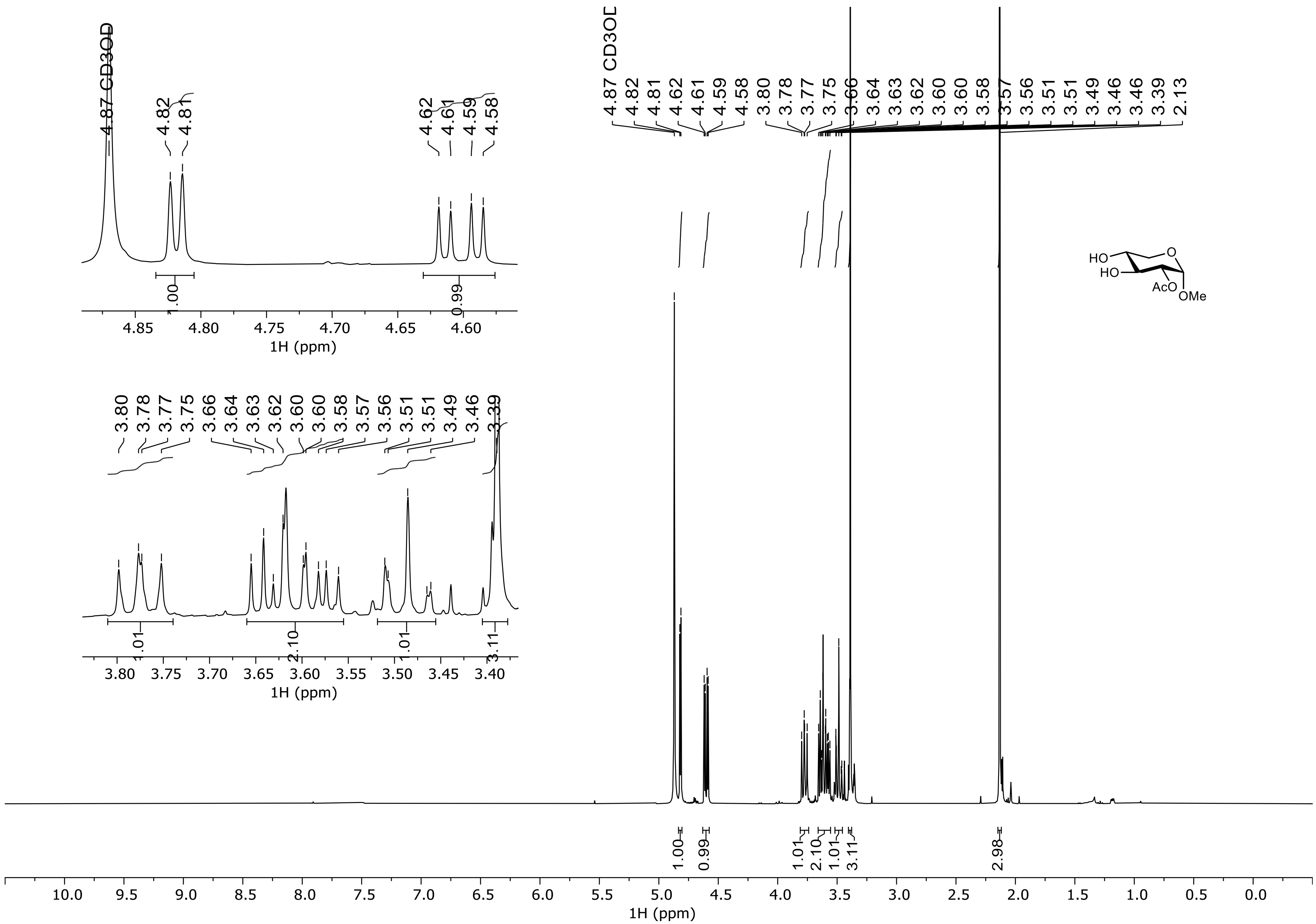

^{13}C (101 MHz, CD_3OD)

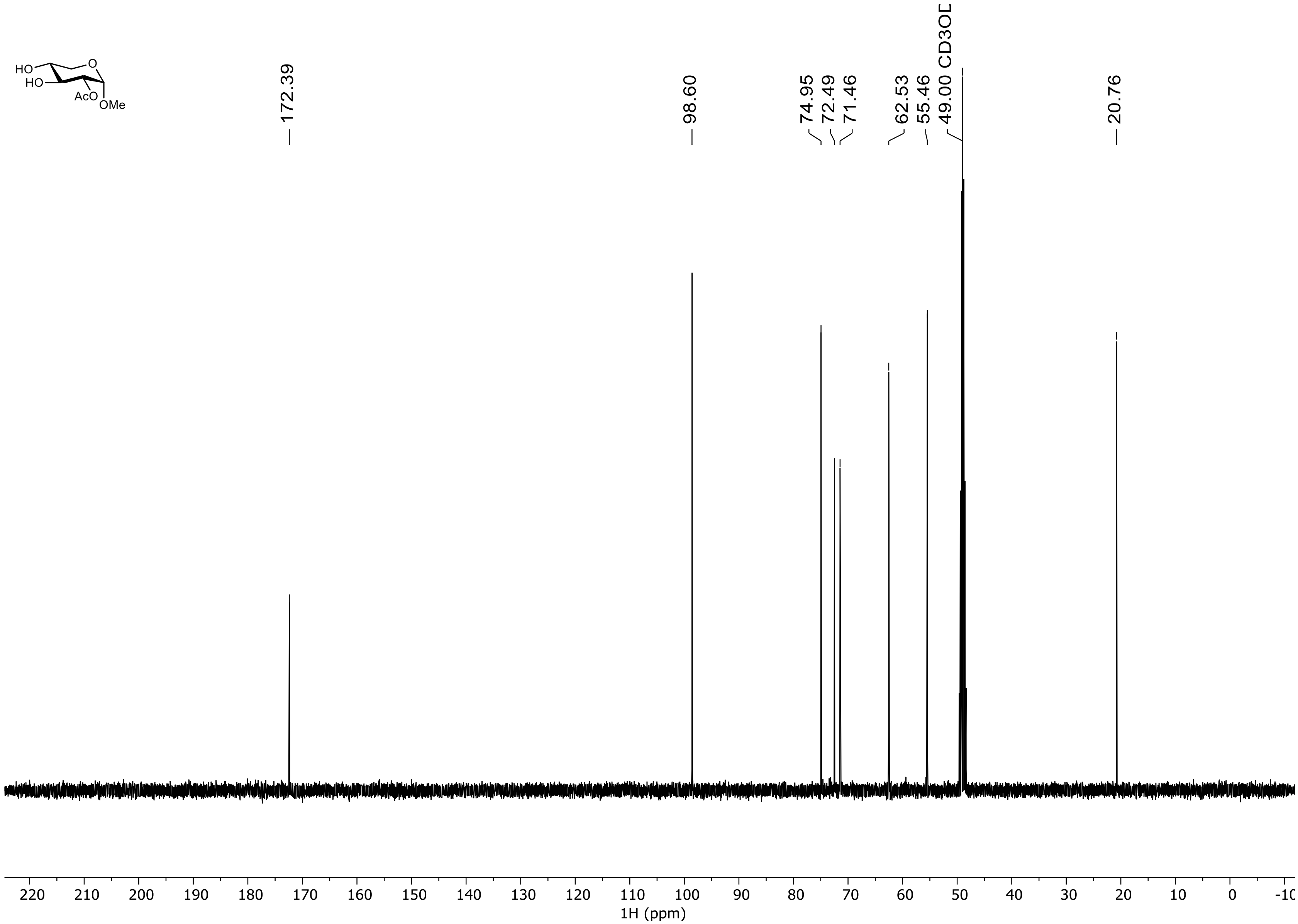

COSY (400 MHz, CD_3OD)

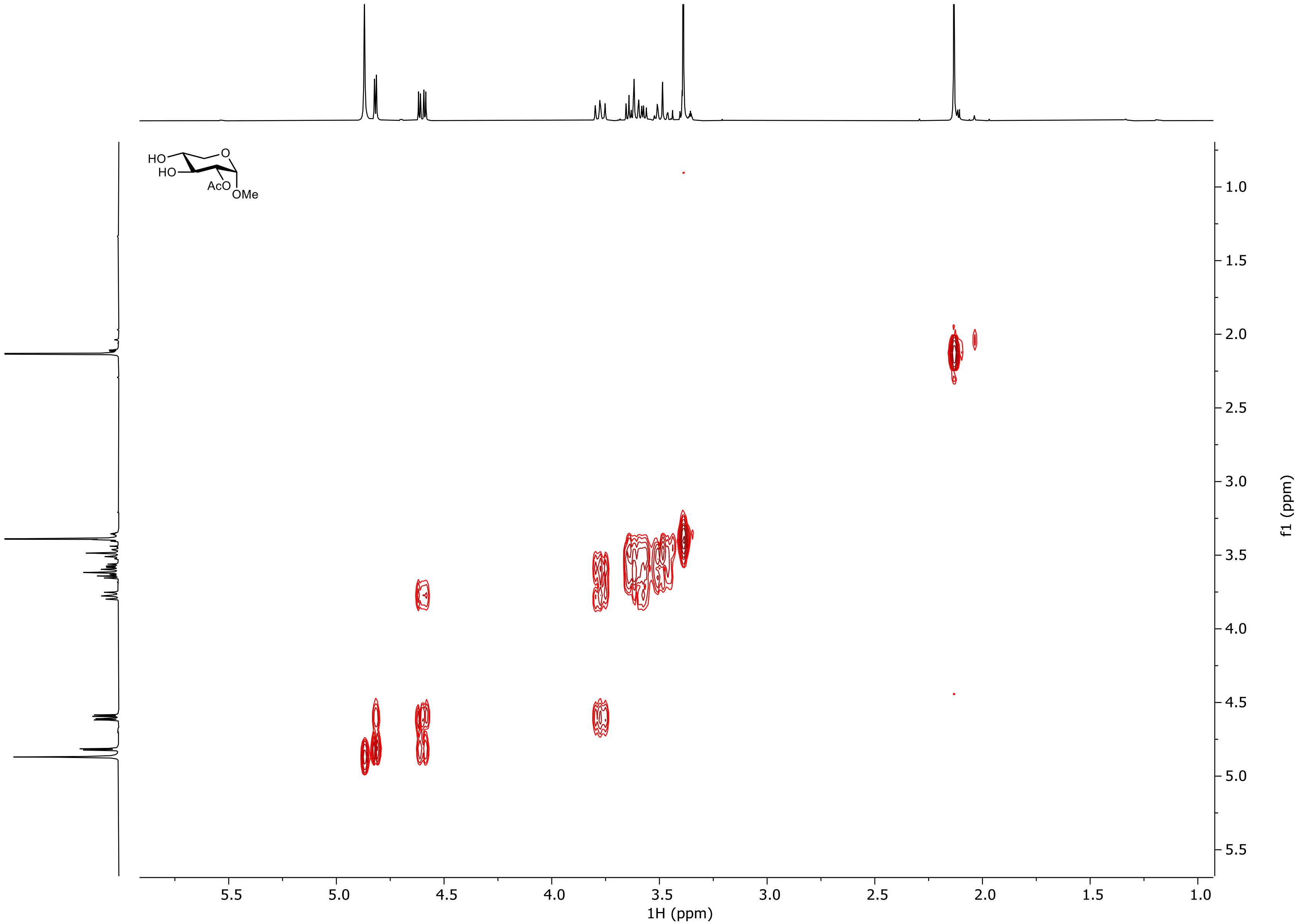

Methyl 4-*O*-acetyl-β-ᴅ-fucopyranoside (2j)

^{1}H NMR (400 MHz, CD_3OD)

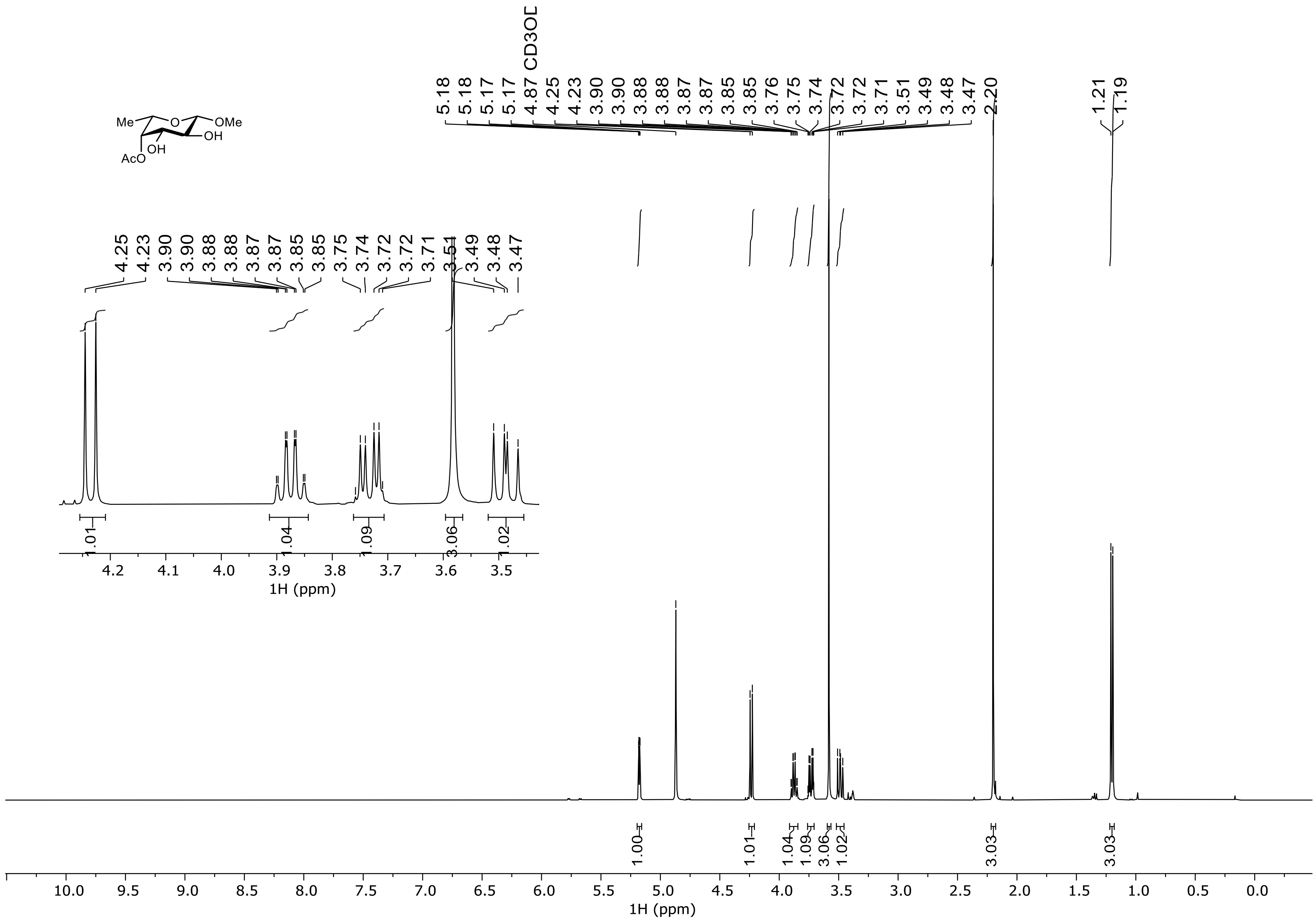

^{13}C NMR (101 MHz, CD_3OD)

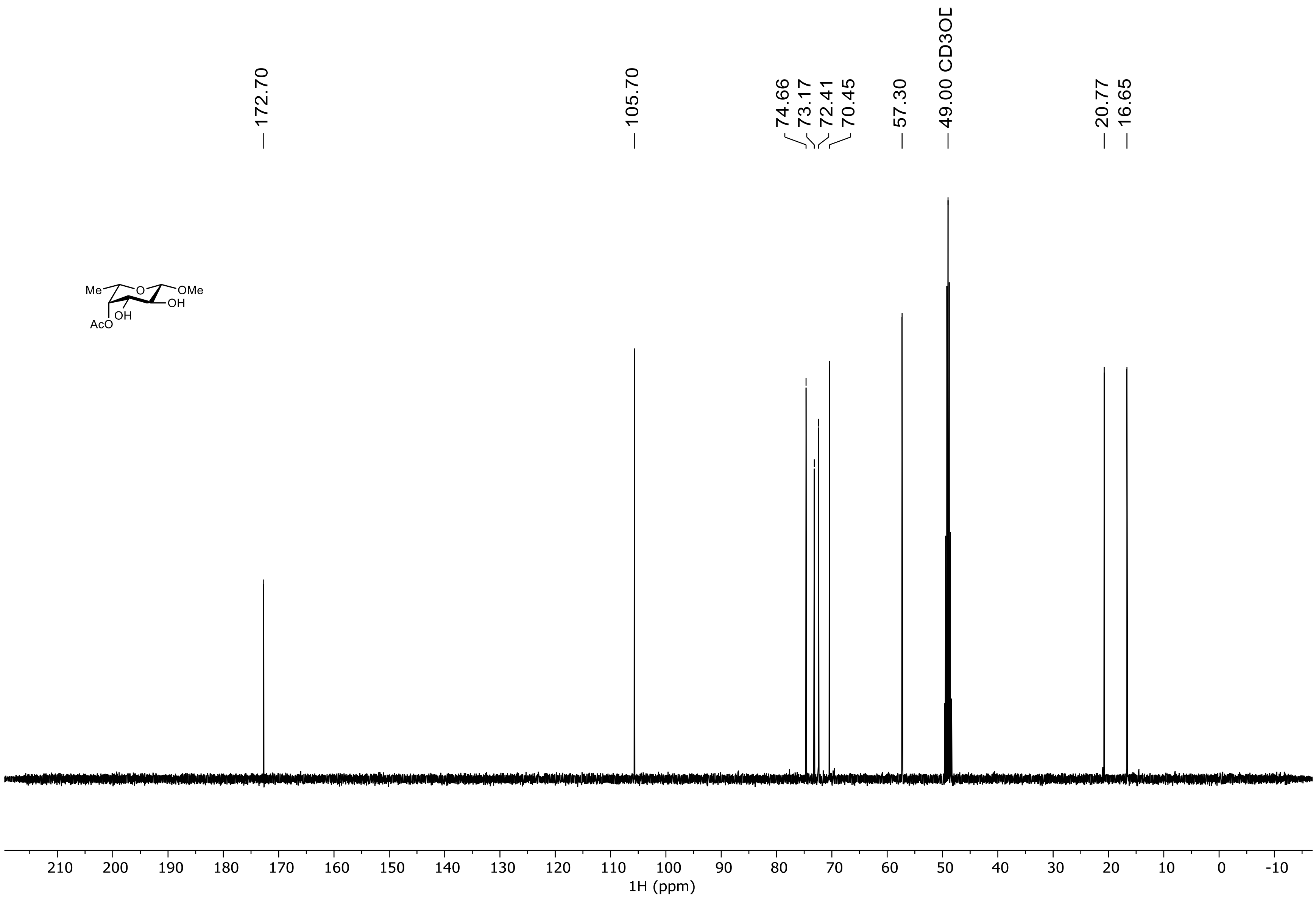

COSY (400 MHz, CD_3OD)

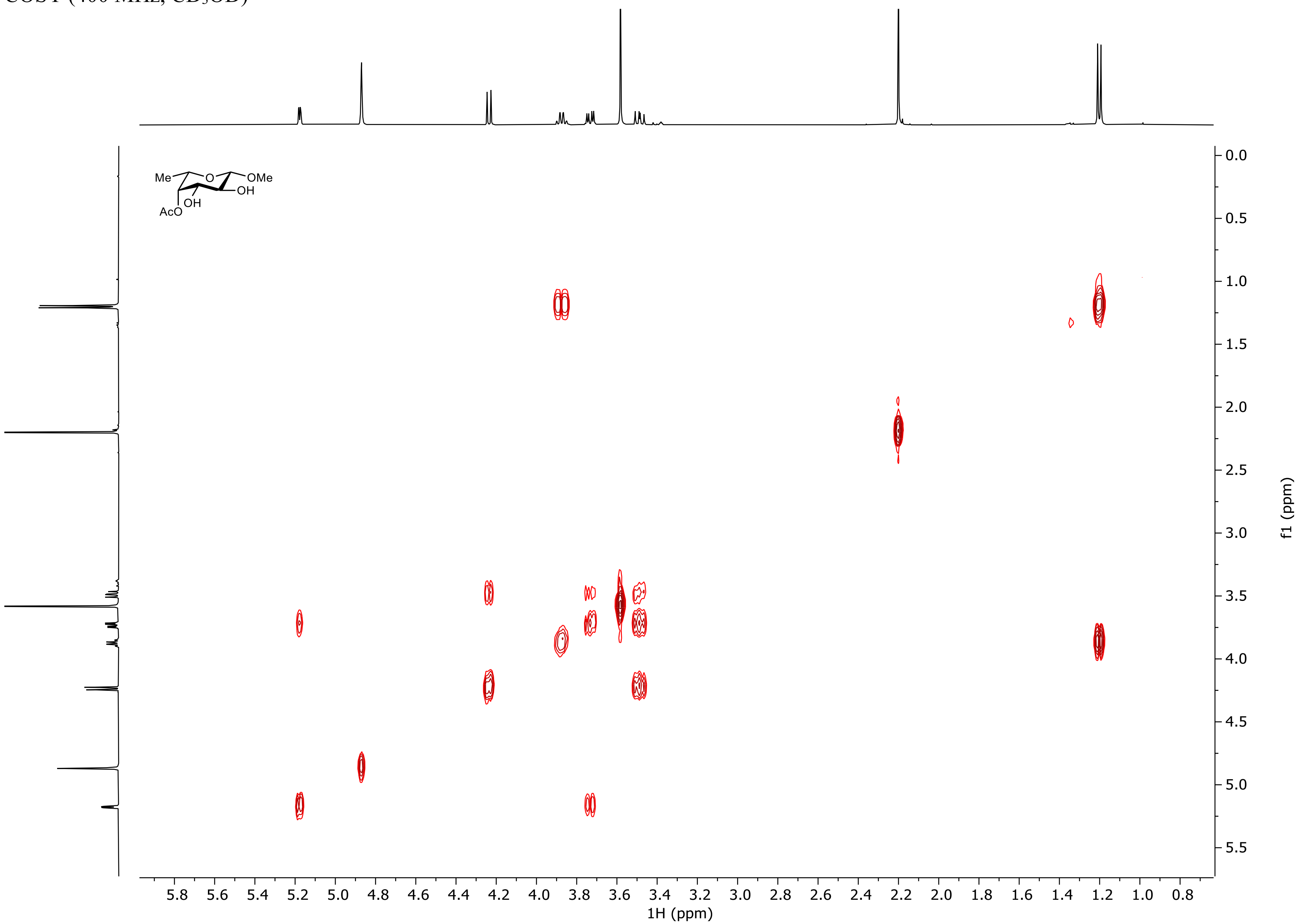

Methyl 2-*O*-acetyl-β-L-Arabinopyranoside (2k)

^{1}H NMR (400 MHz, CD_3OD)

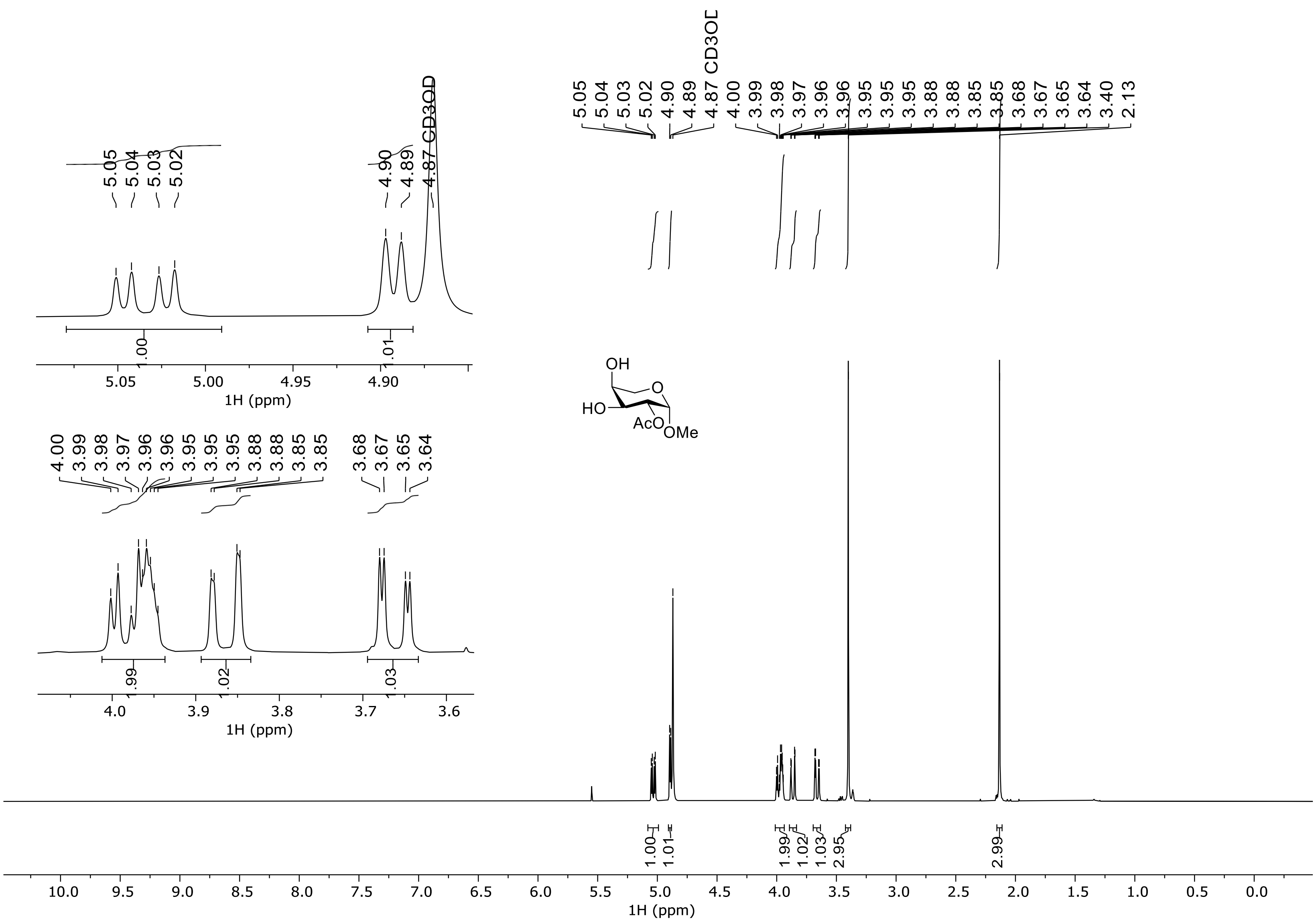

^{13}C NMR (101 MHz, CD_3OD)

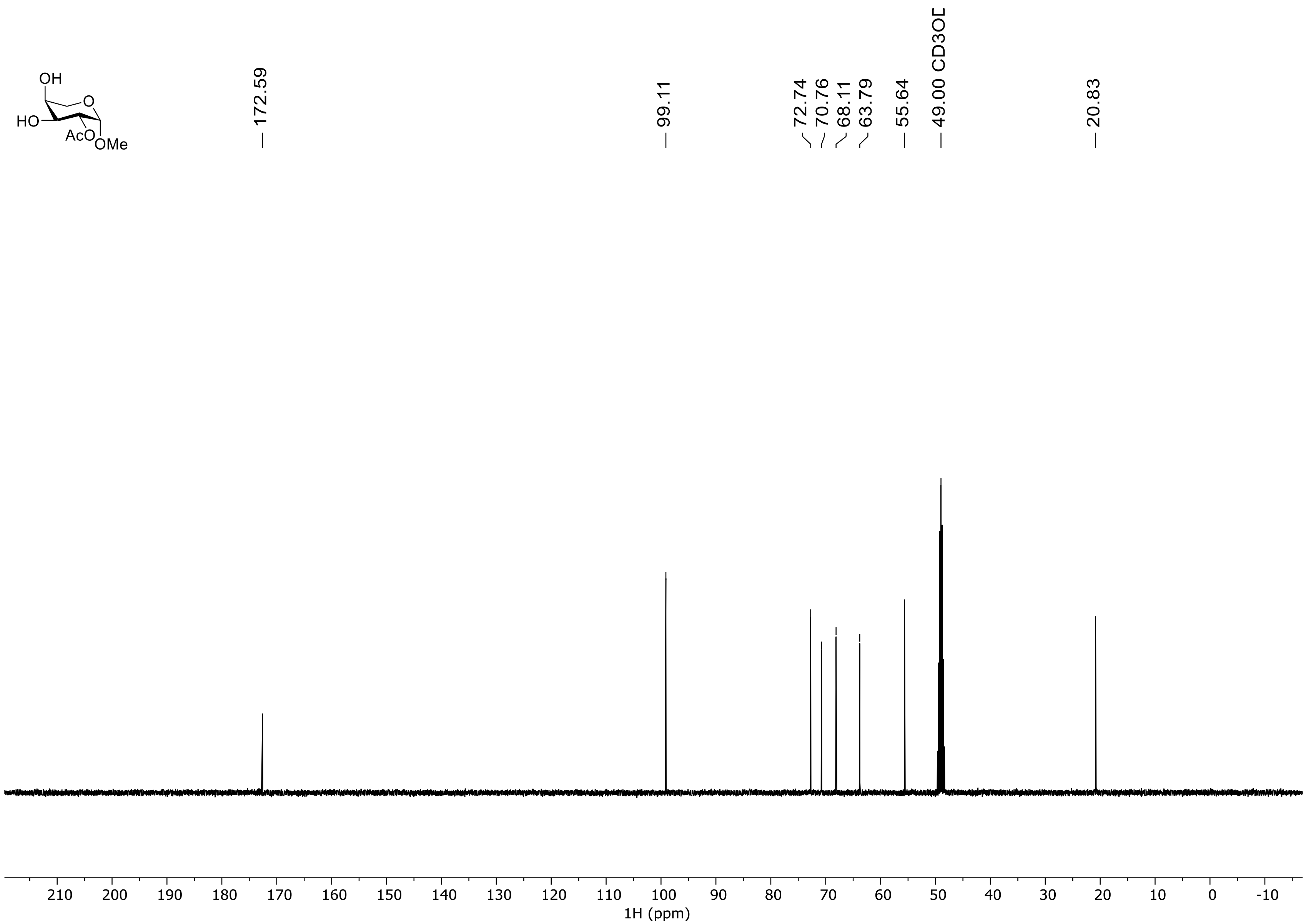

COSY (400 MHz, CD_3OD)

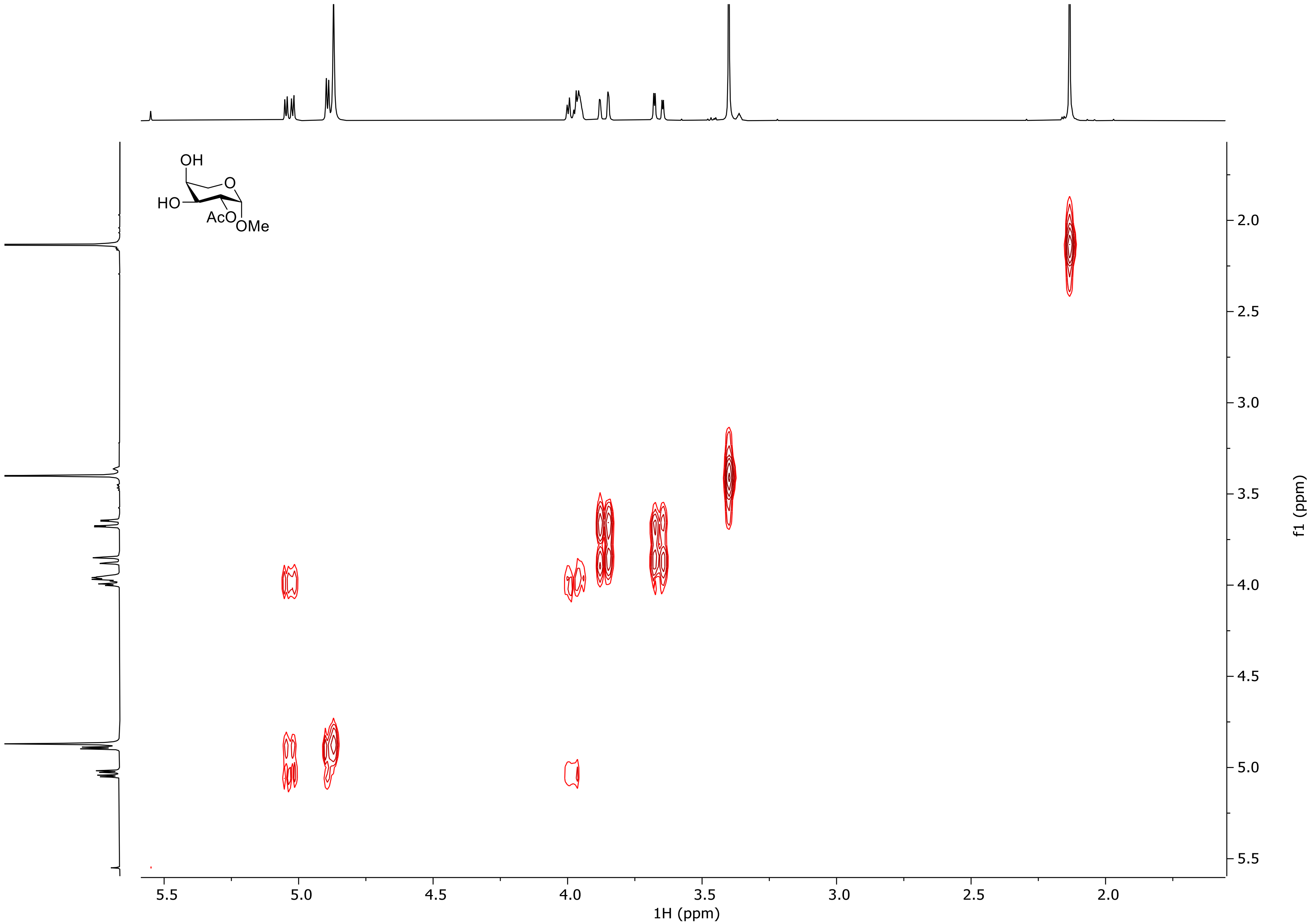

Methyl 4-*O*-acetyl-β-ᴅ-Arabinopyranoside (2l)

^{1}H NMR (400 MHz, CD_3OD)

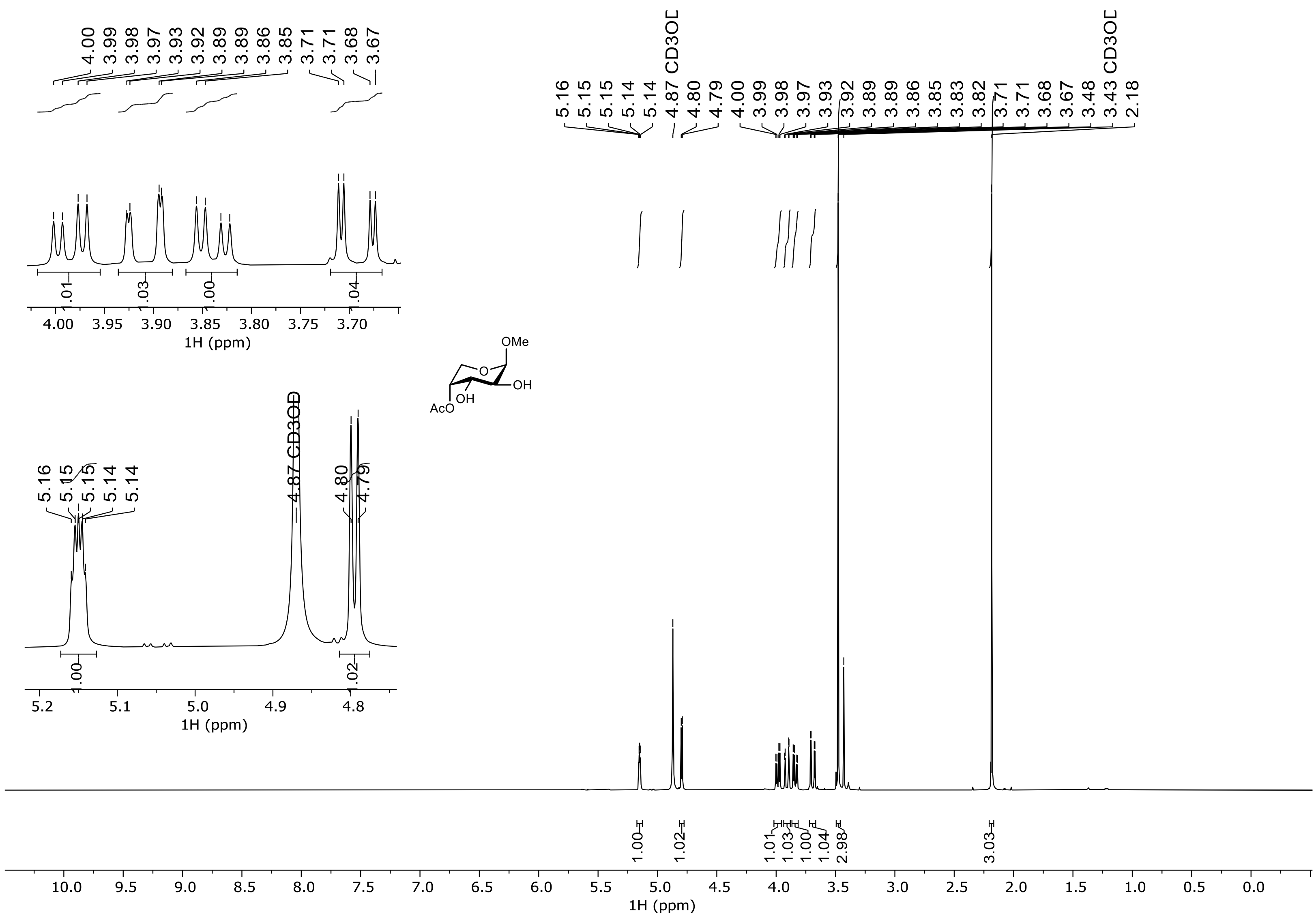

^{13}C NMR (101MHz, CD_3OD)

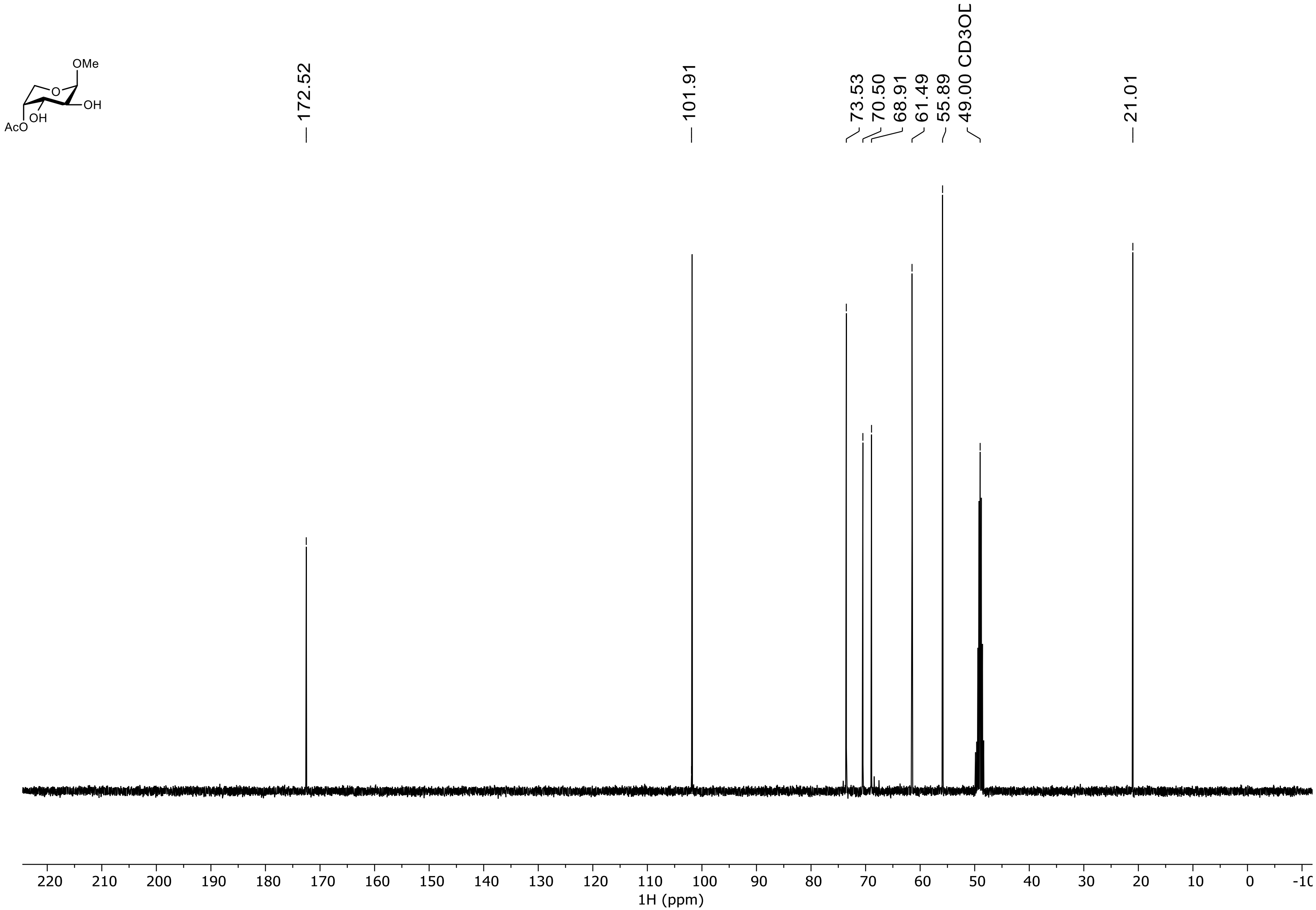

COSY (400 MHz, CD_3OD)

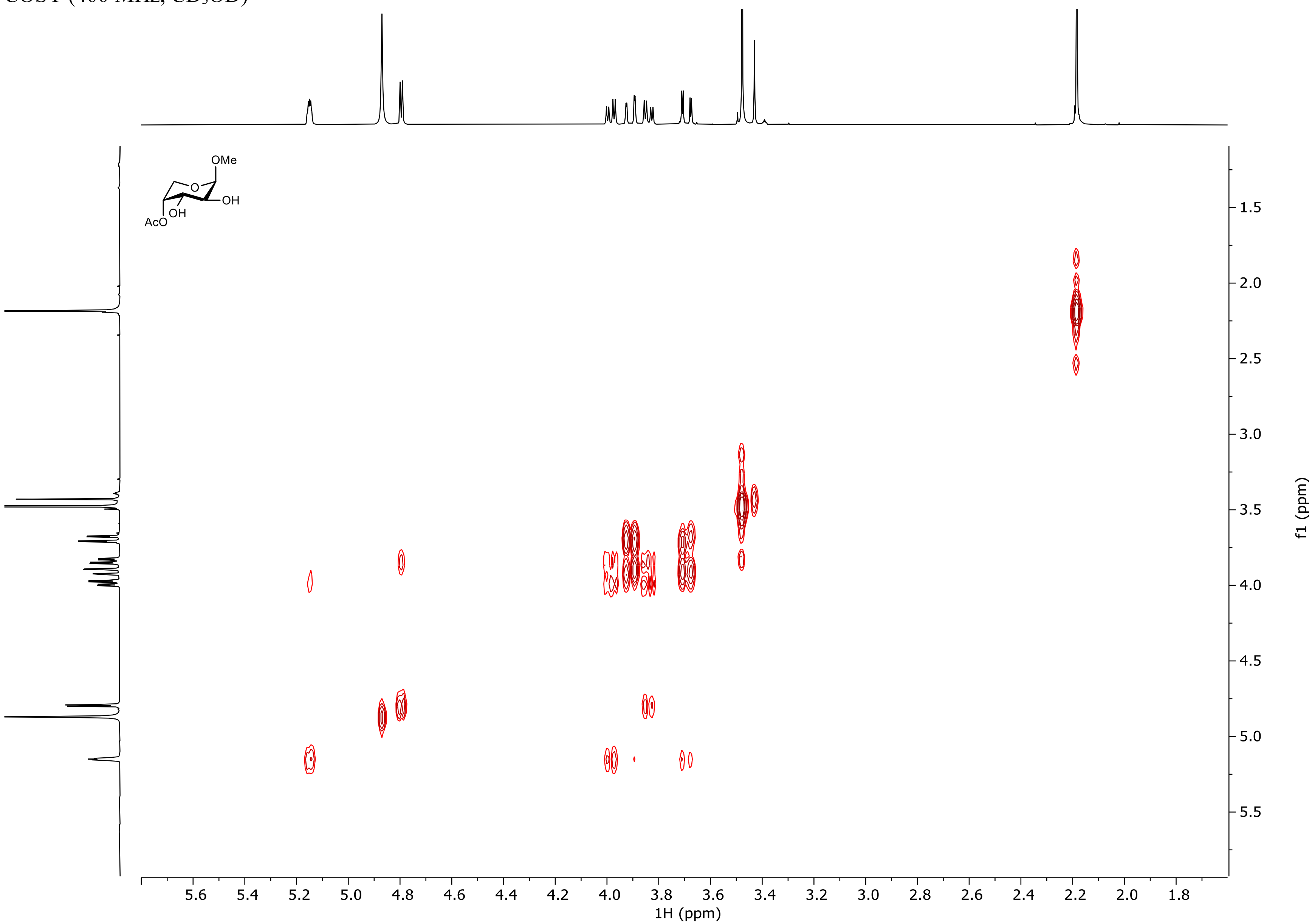

Methyl 4-*O*-acetyl-α-L-rhamnopyranoside (2m)

^{1}H NMR (400 MHz, CD_3OD)

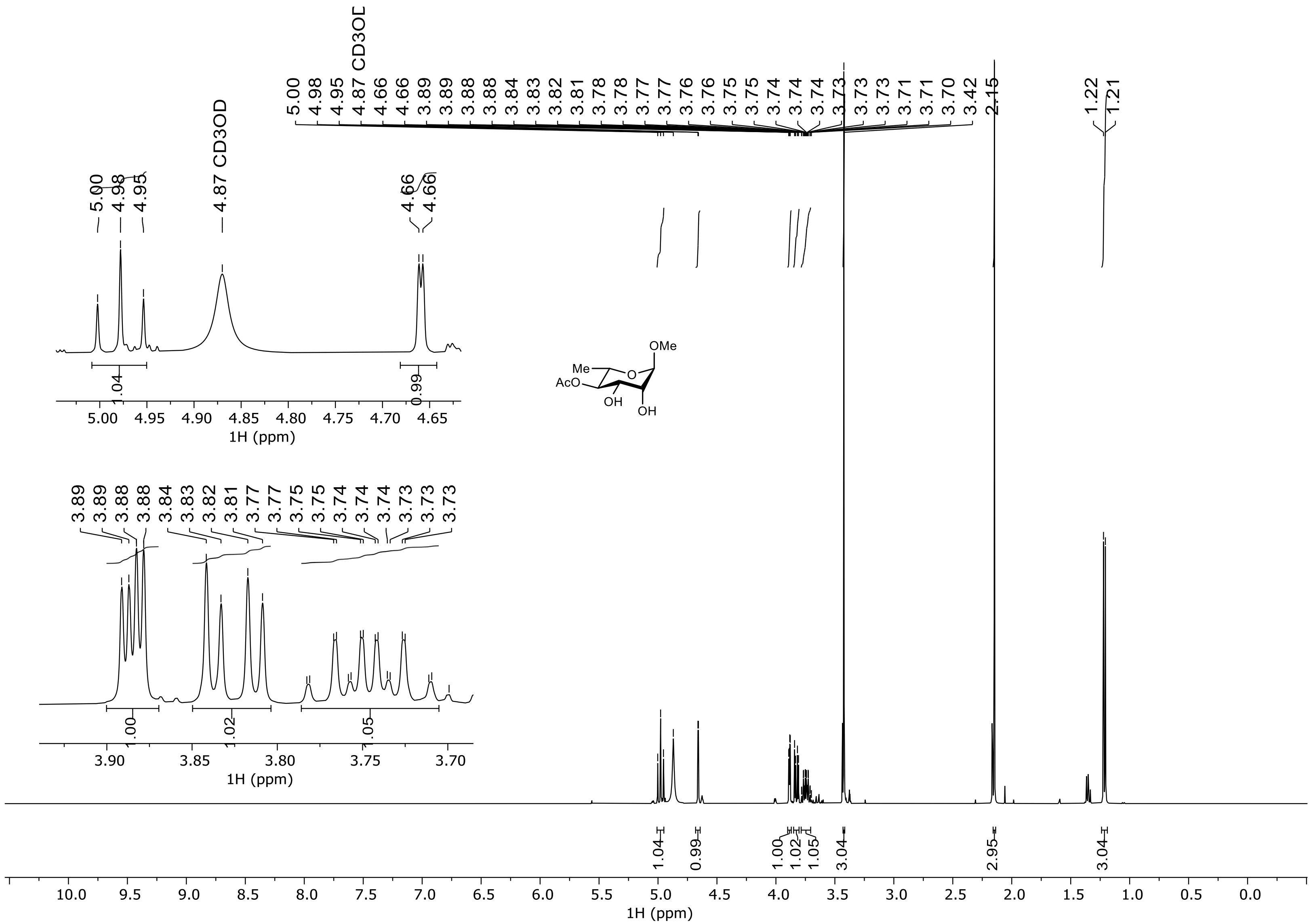

^{13}C NMR (101MHz, CD_3OD)

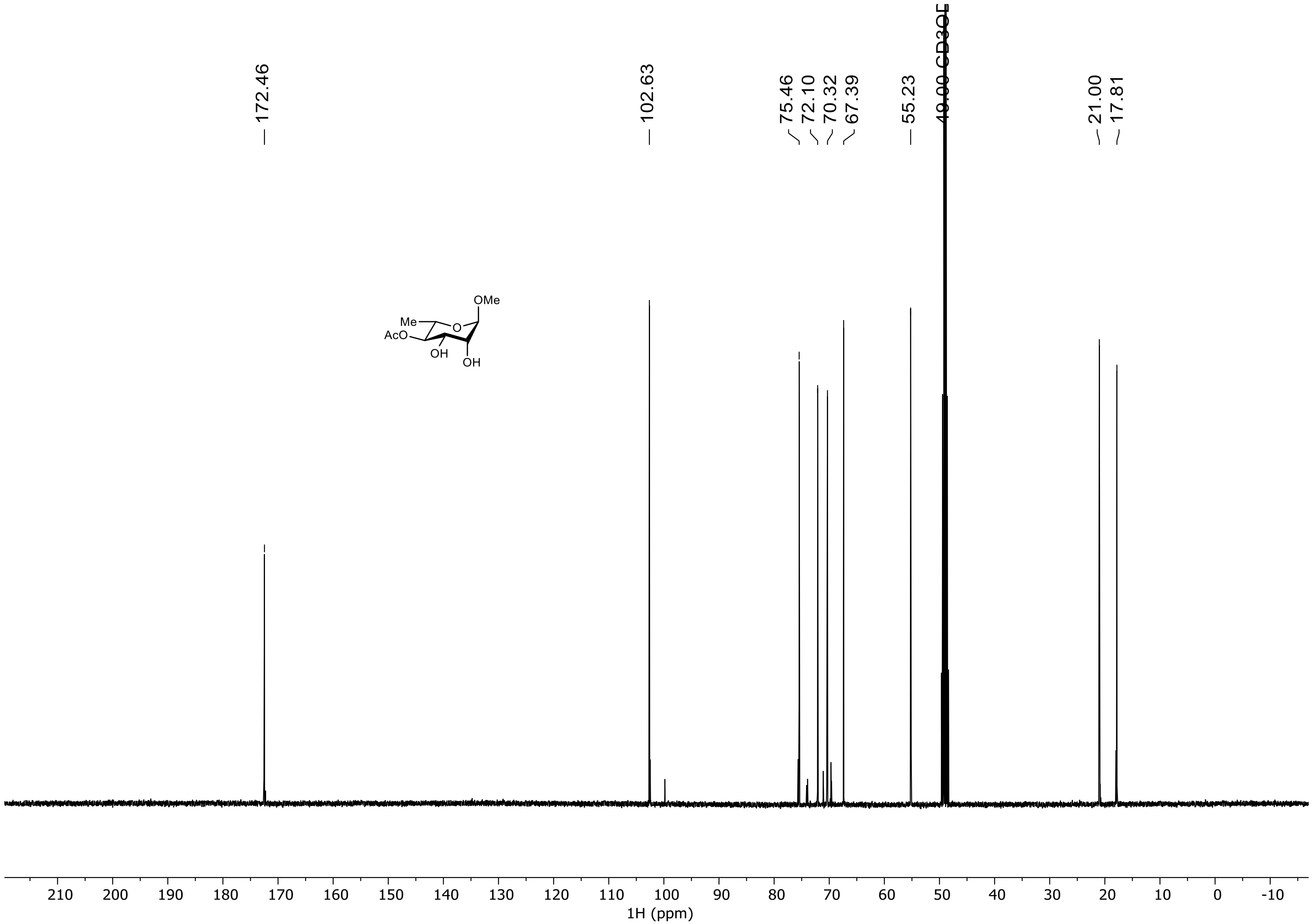

COSY (400 MHz, CD_3OD)

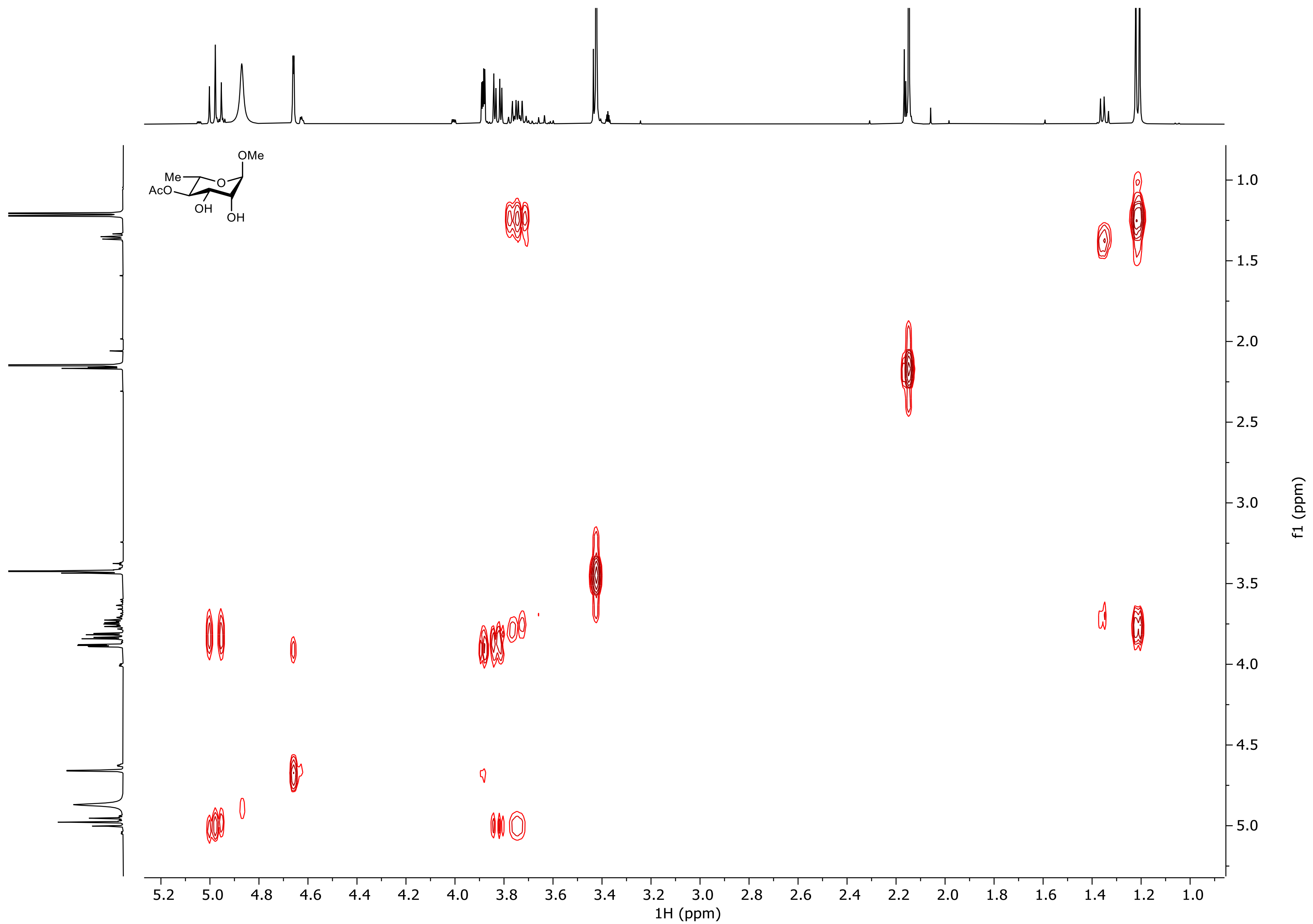

Ethyl 1-thio-4-*O*-acetyl-α-L-rhamnopyranoside (2n)

^{1}H NMR (400 MHz, CD_3OD)

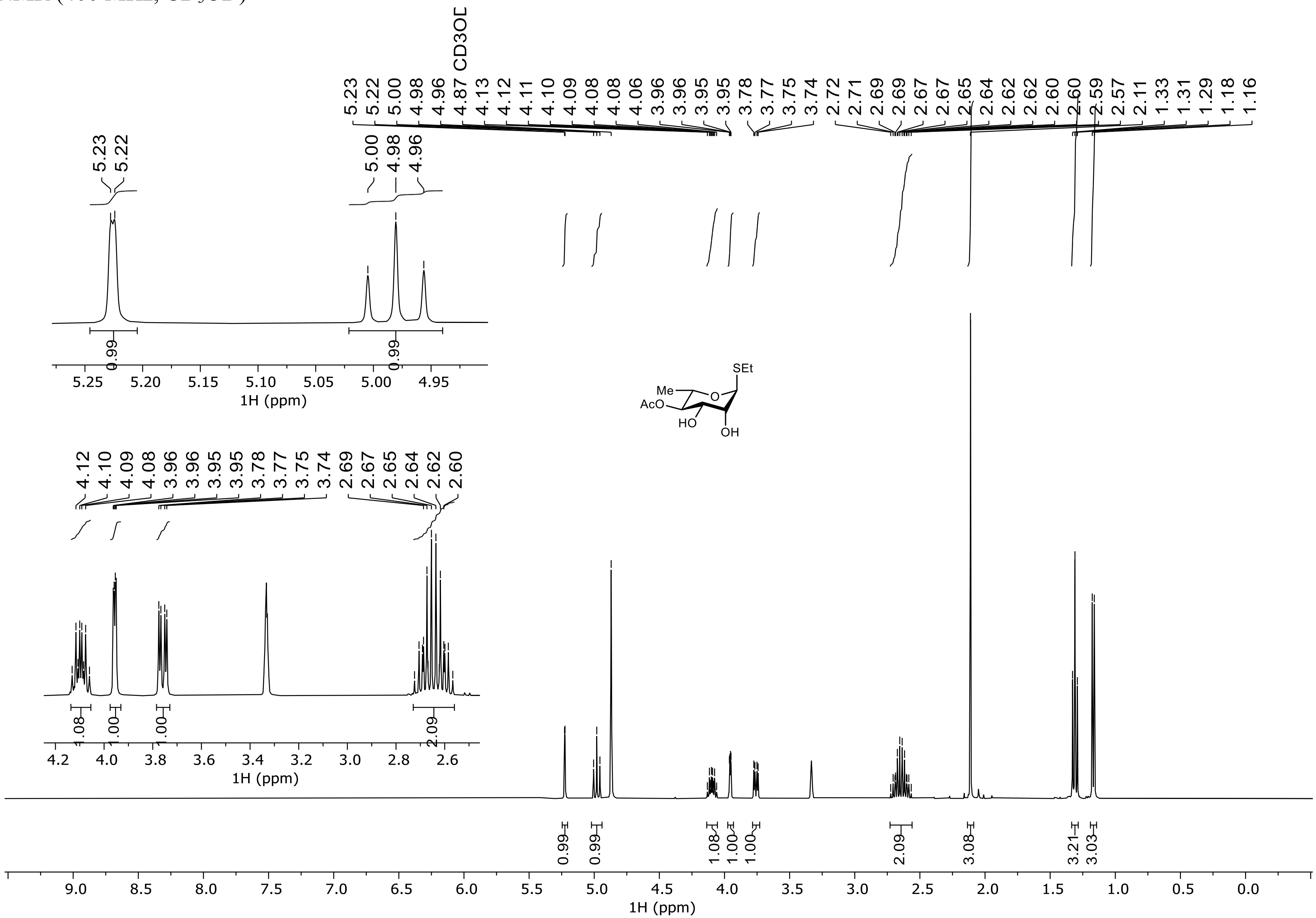

^{13}C NMR (101 MHz, CD_3OD)

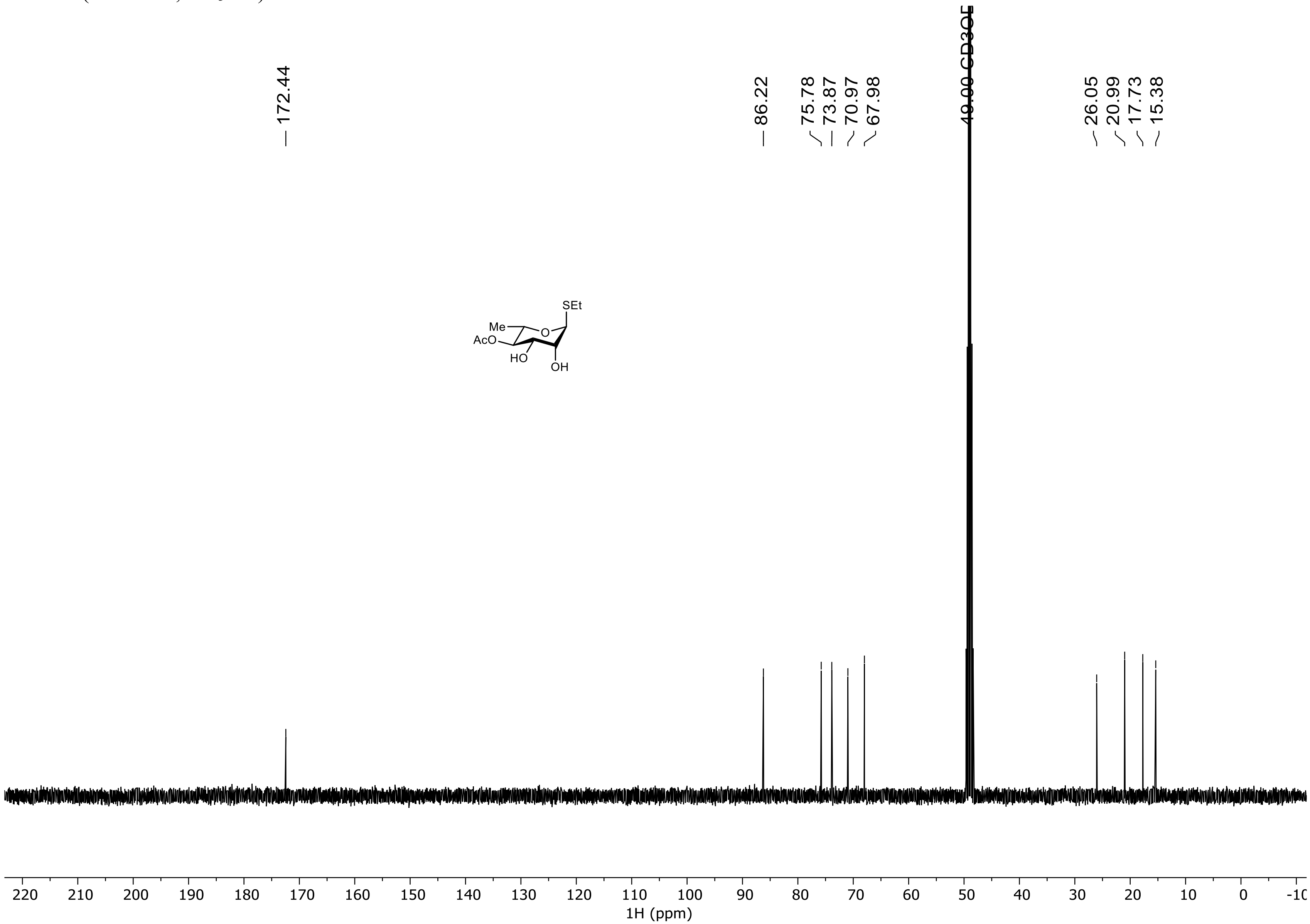

COSY (400 MHz, CD_3OD)

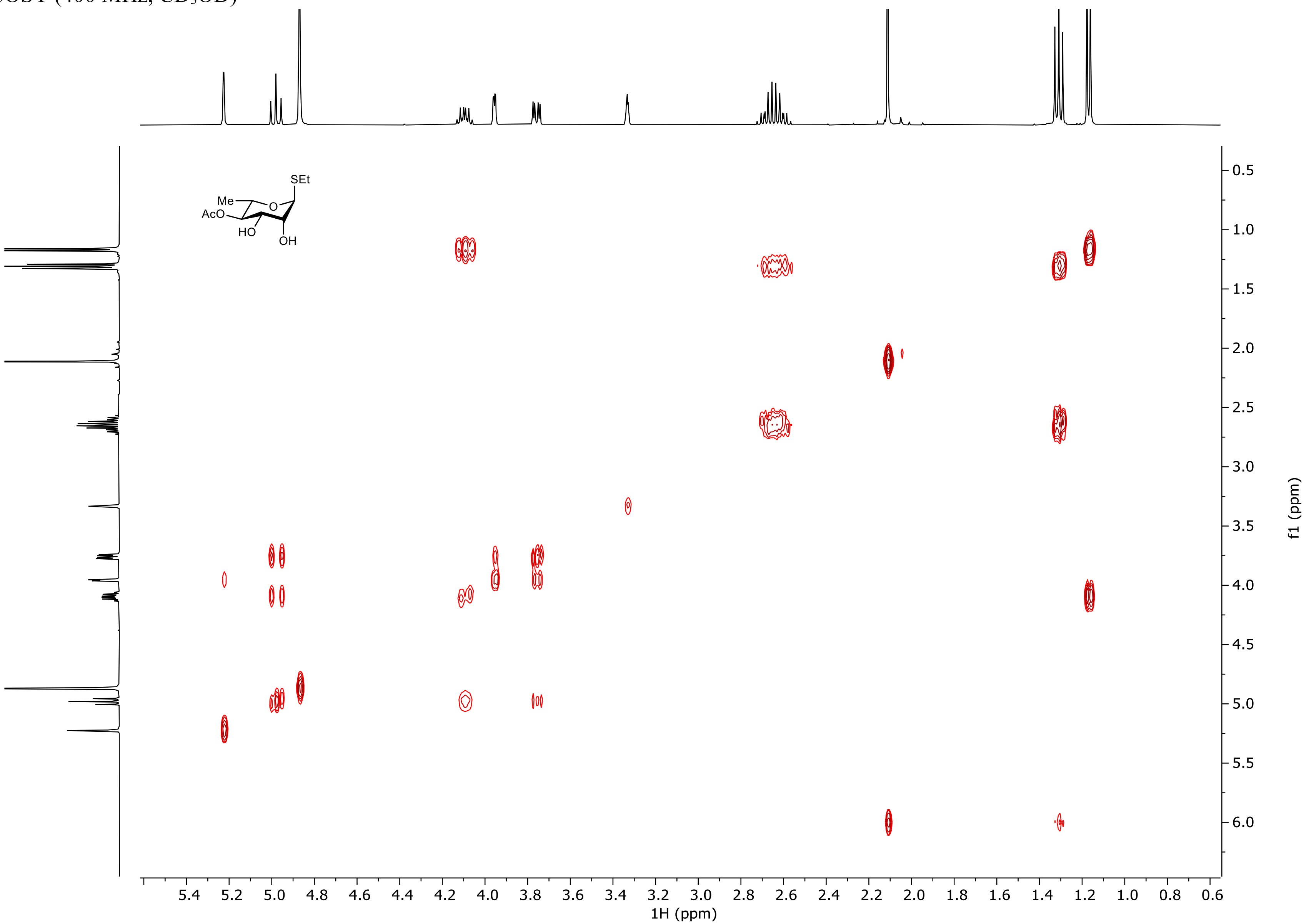

Methyl 6-*O*-acetyl-α-ᴅ-glucopyranoside (2o)

^{1}H NMR (400 MHz, CD_3OD)

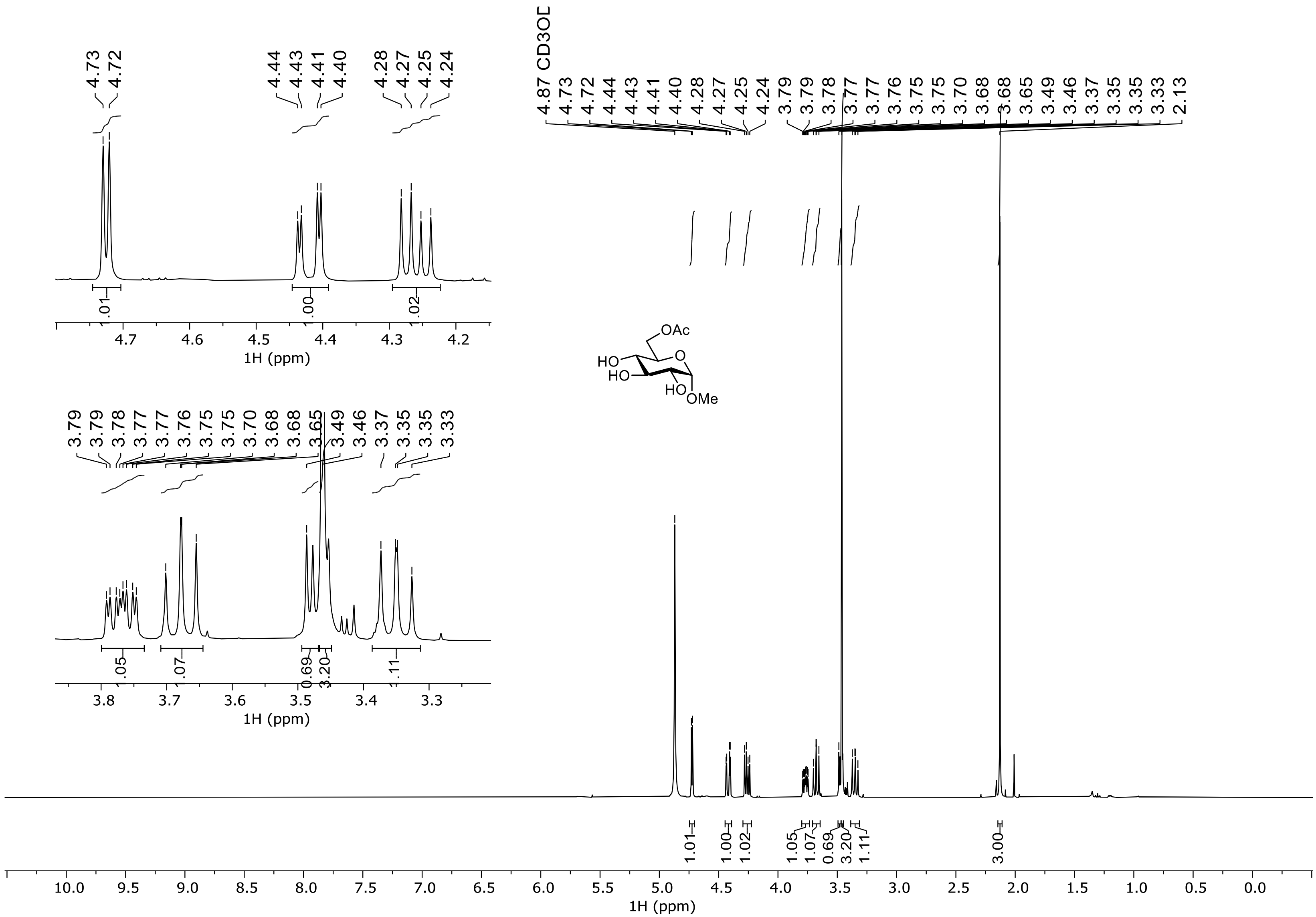

^{13}C NMR (101MHz, CD_3OD)

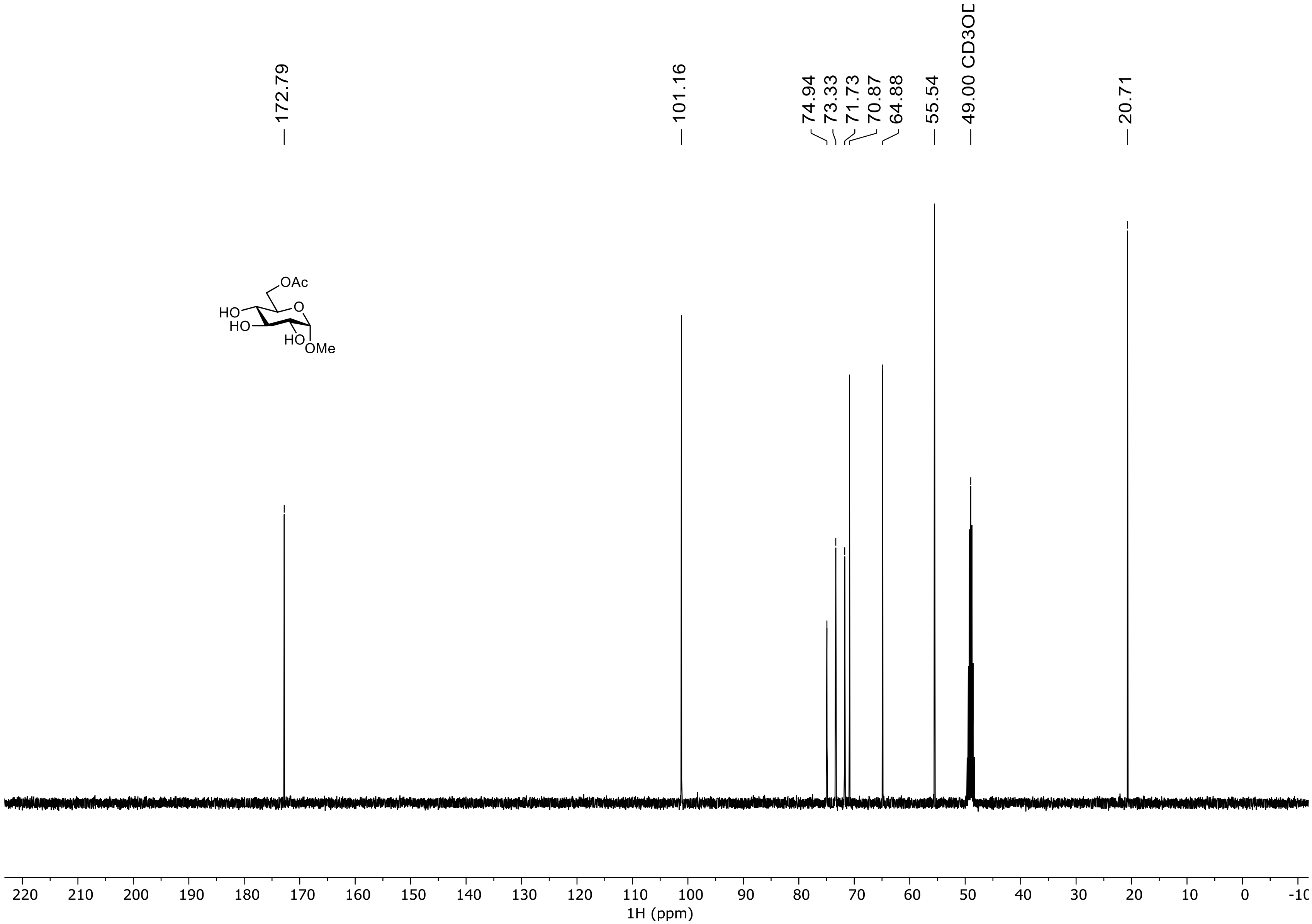

COSY (400 MHz, CD_3OD)

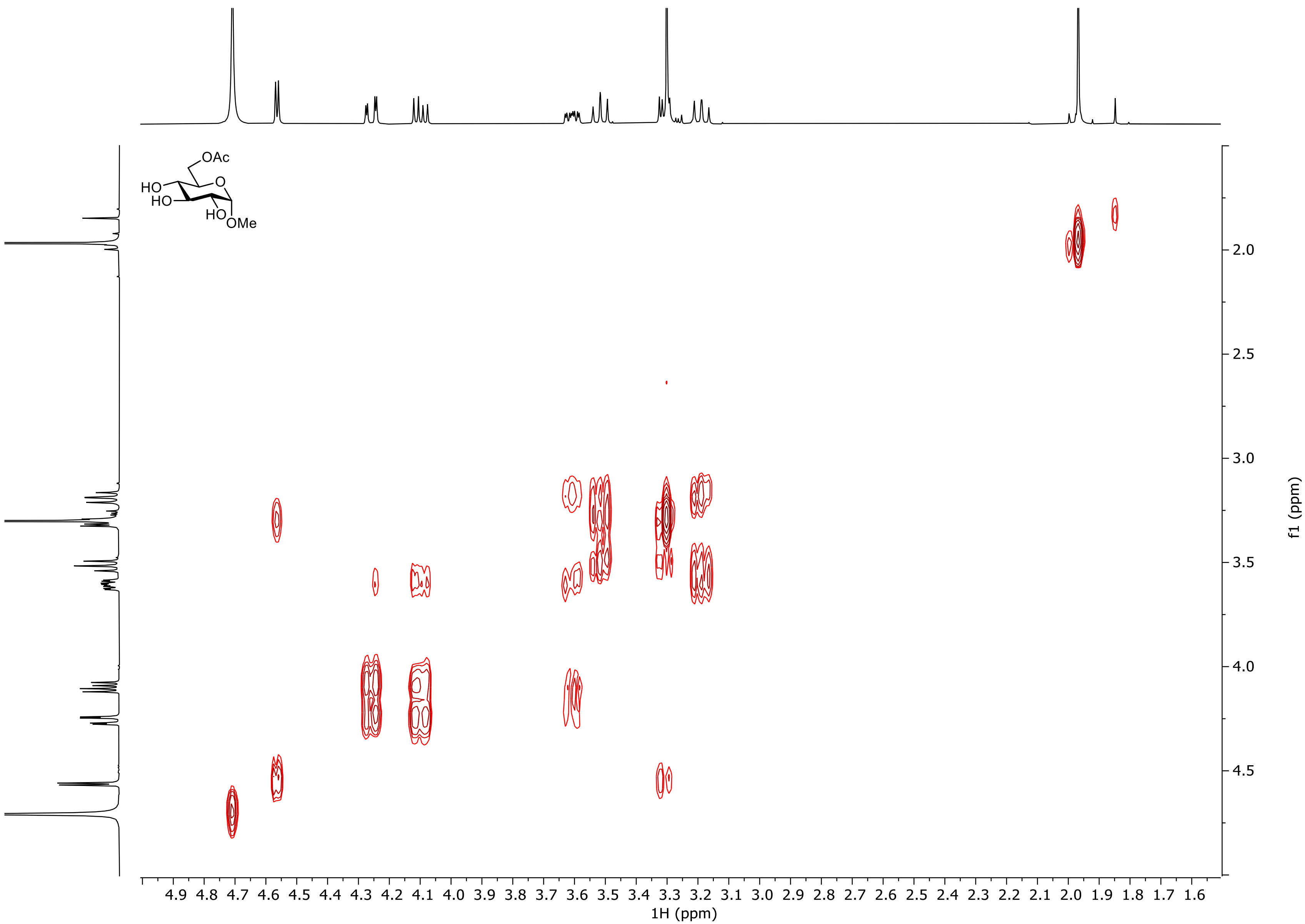

Methyl 6-*O*-acetyl-β-D-galactopyranoside (2p)

^{1}H NMR (400 MHz, CD_3OD)

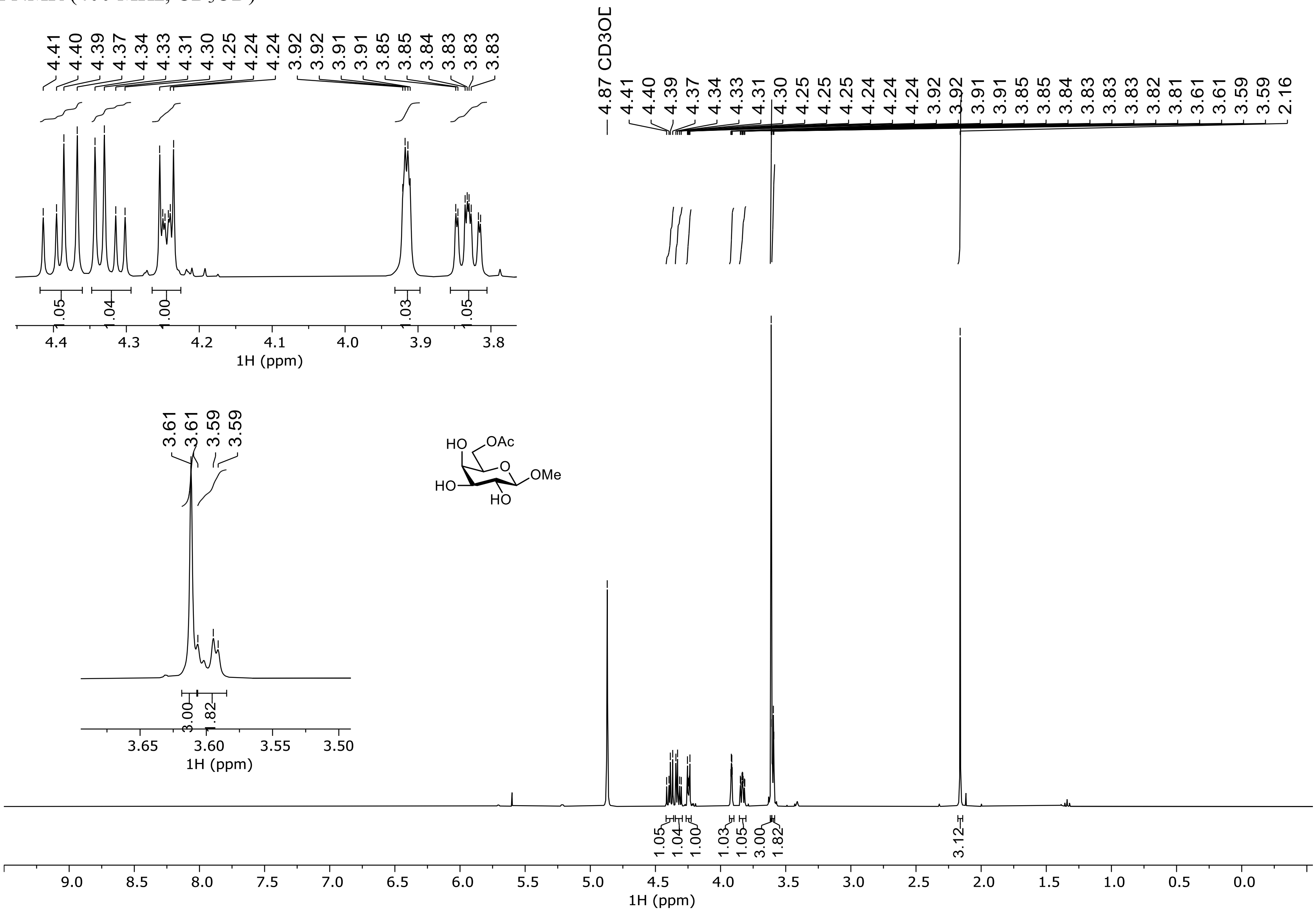

^{13}C NMR (101 MHz, CD_3OD)

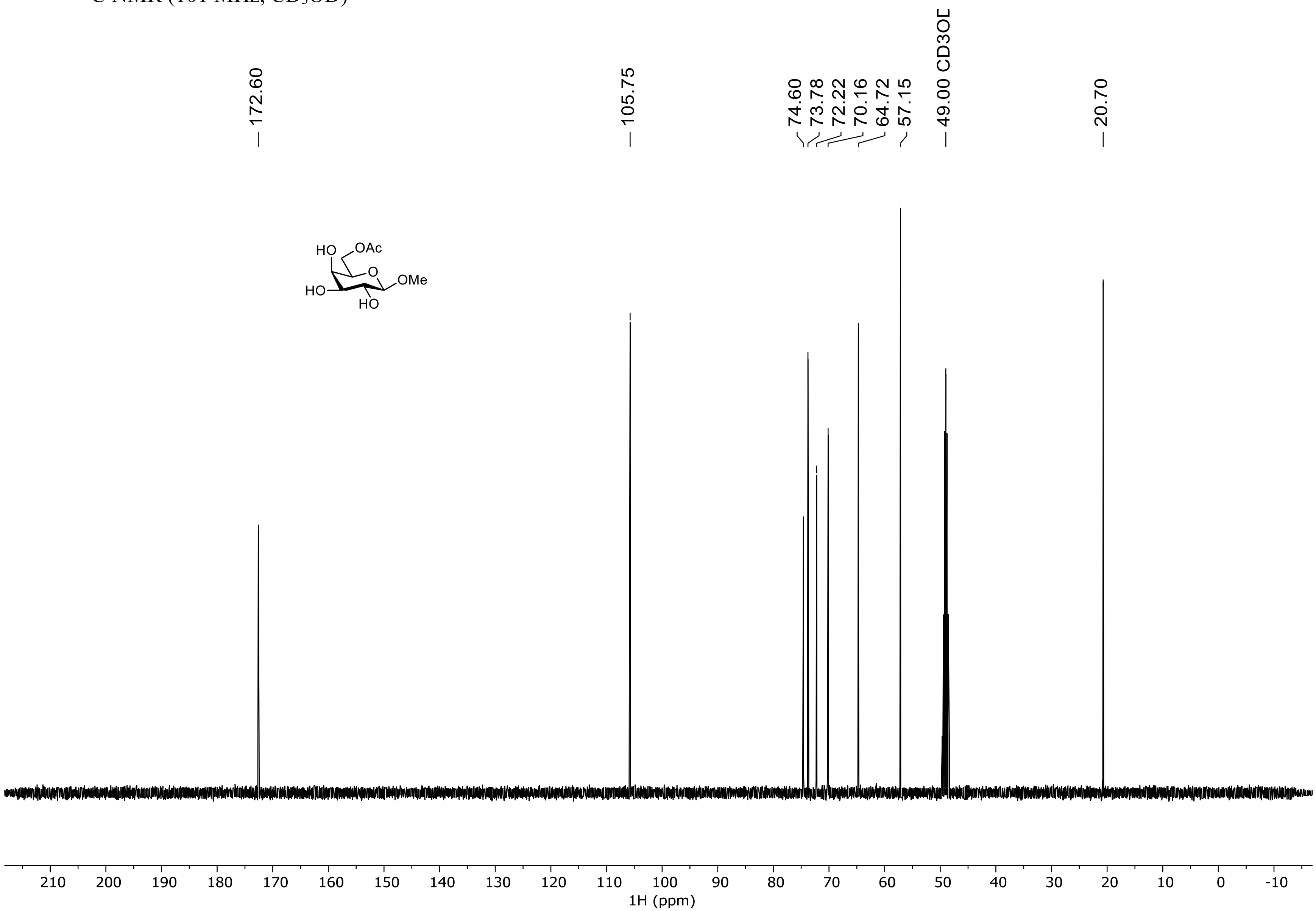

COSY (400 MHz, CD3OD)

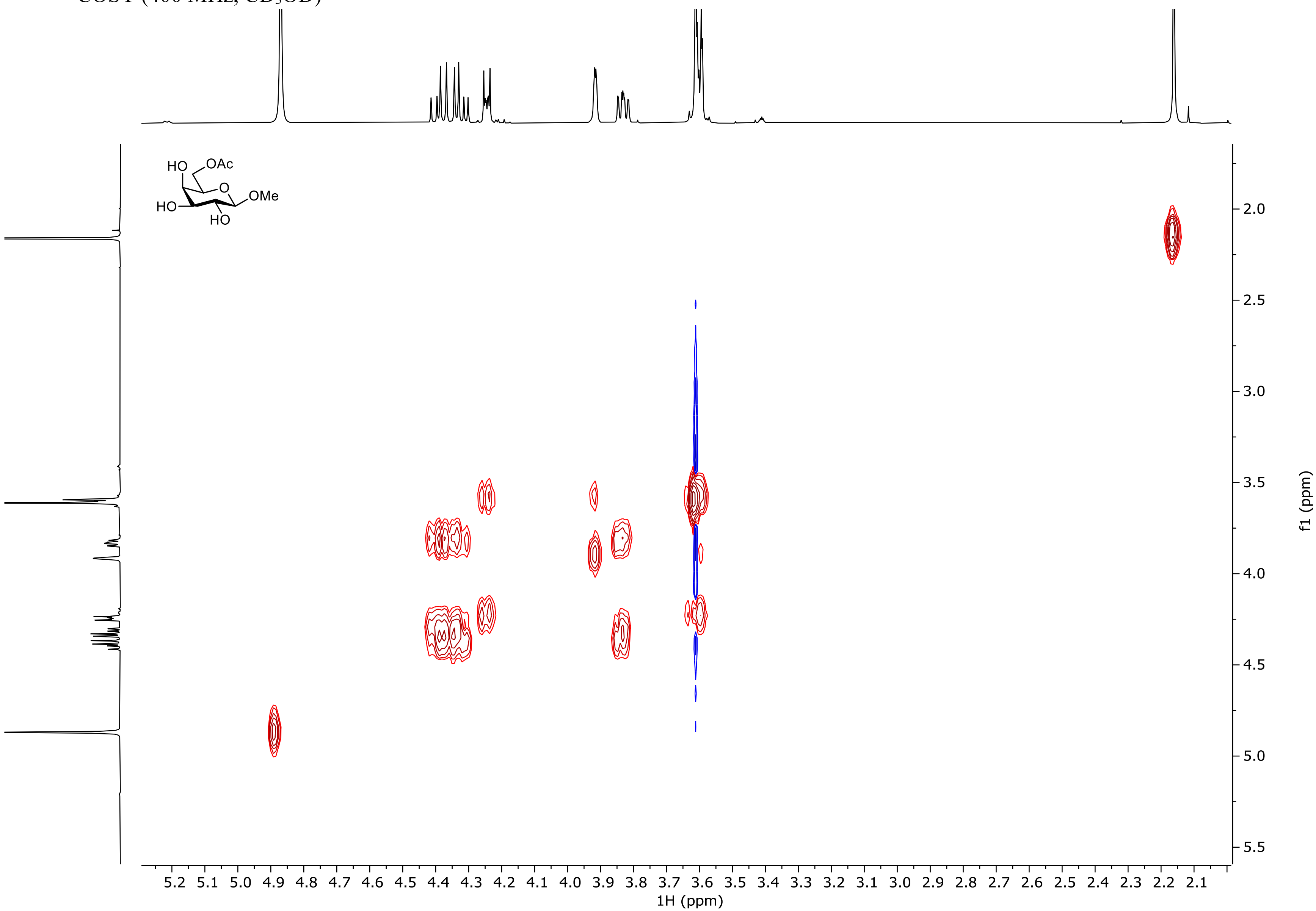

Isopropyl 6-*O*-acetyl-β-D-1-thiogalactopyranoside (2q)

^{1}H NMR (400 MHz, CD_3OD)

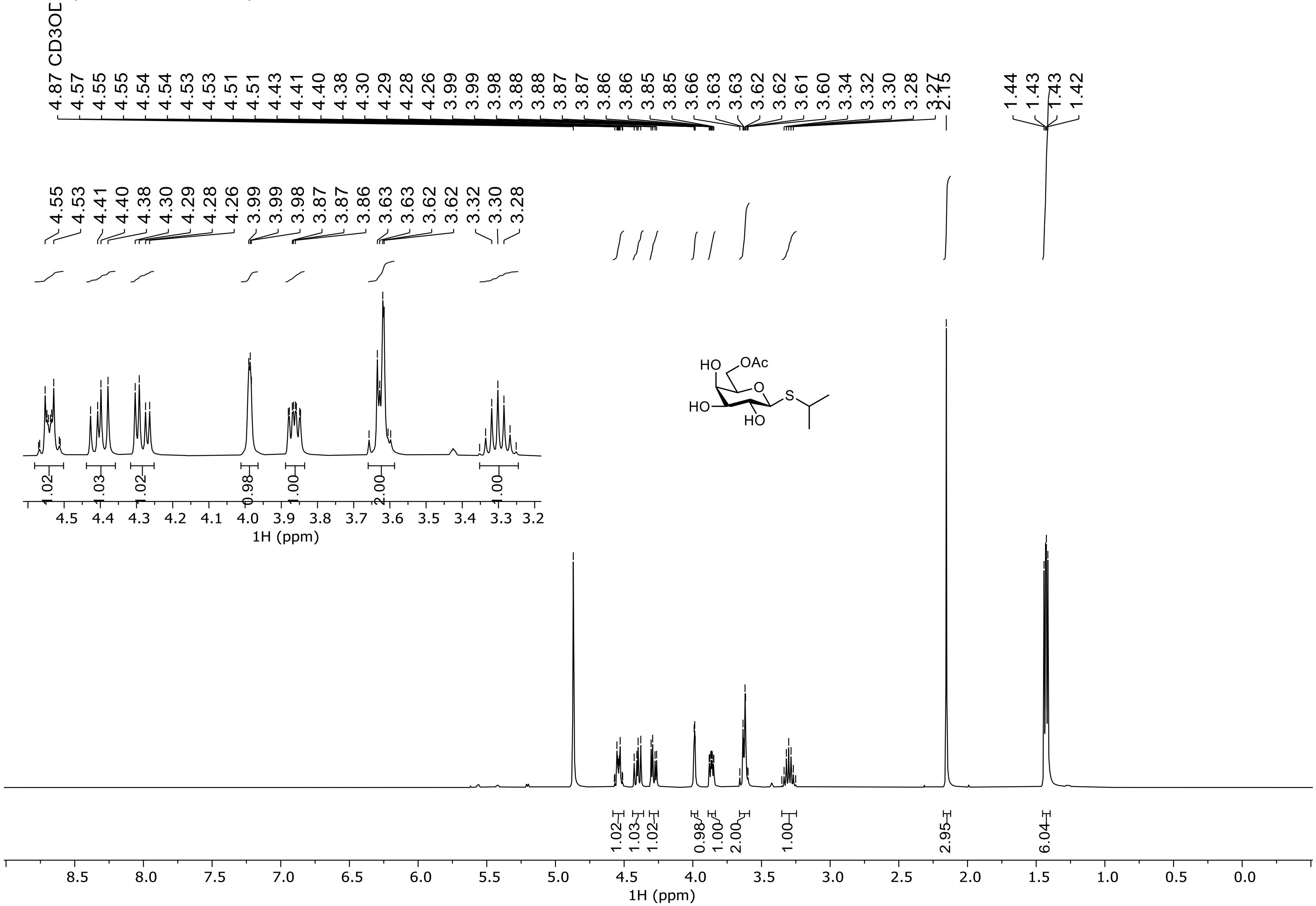

^{13}C NMR (101 MHz, CD_3OD)

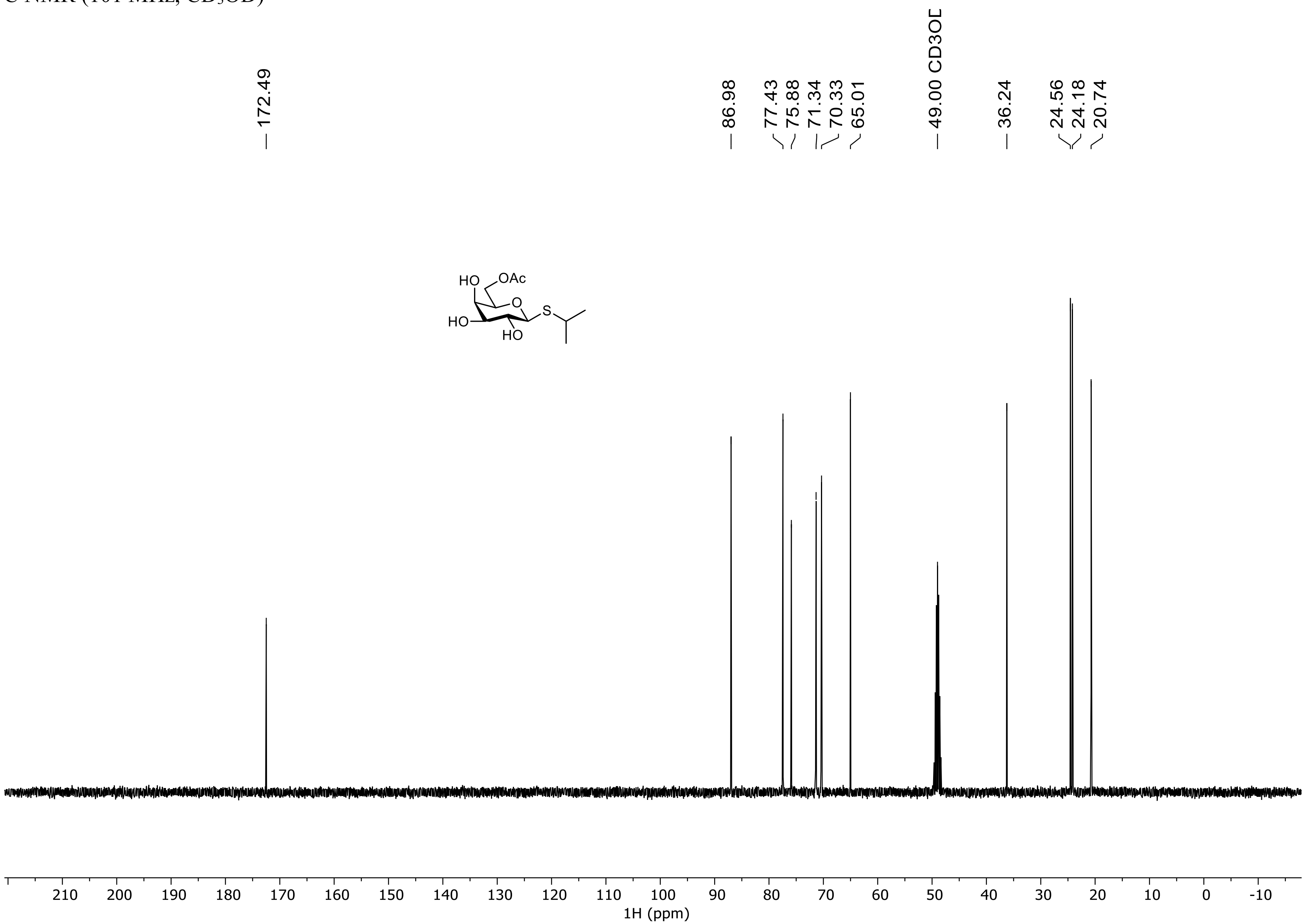

COSY (400 MHz, CD_3OD)

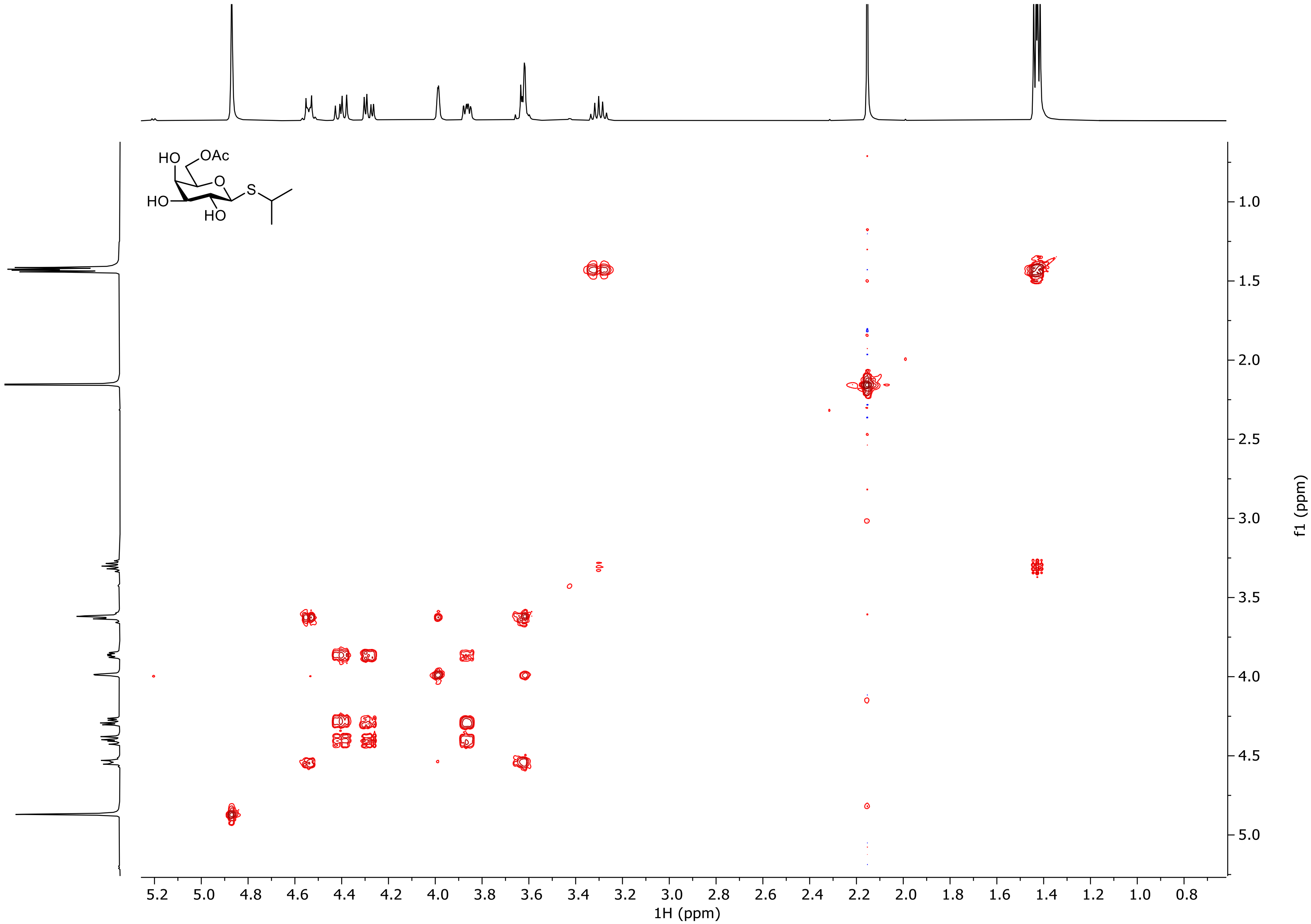

Methyl 6-*O*-acetyl-α-D-mannopyranoside (2r)

^{1}H NMR (400 MHz, CD_3OD)

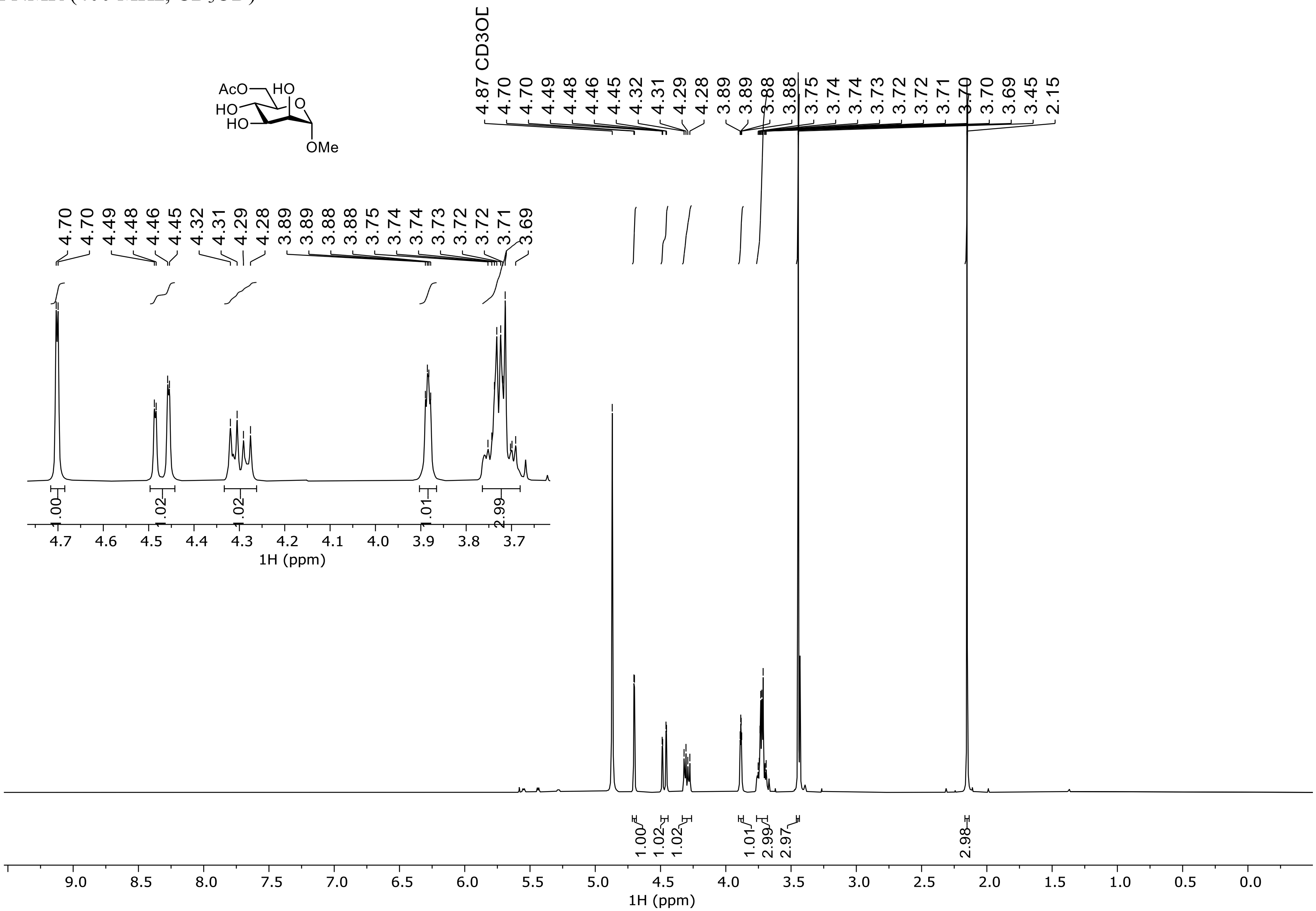

^{13}C NMR (101 MHz, CD_3OD)

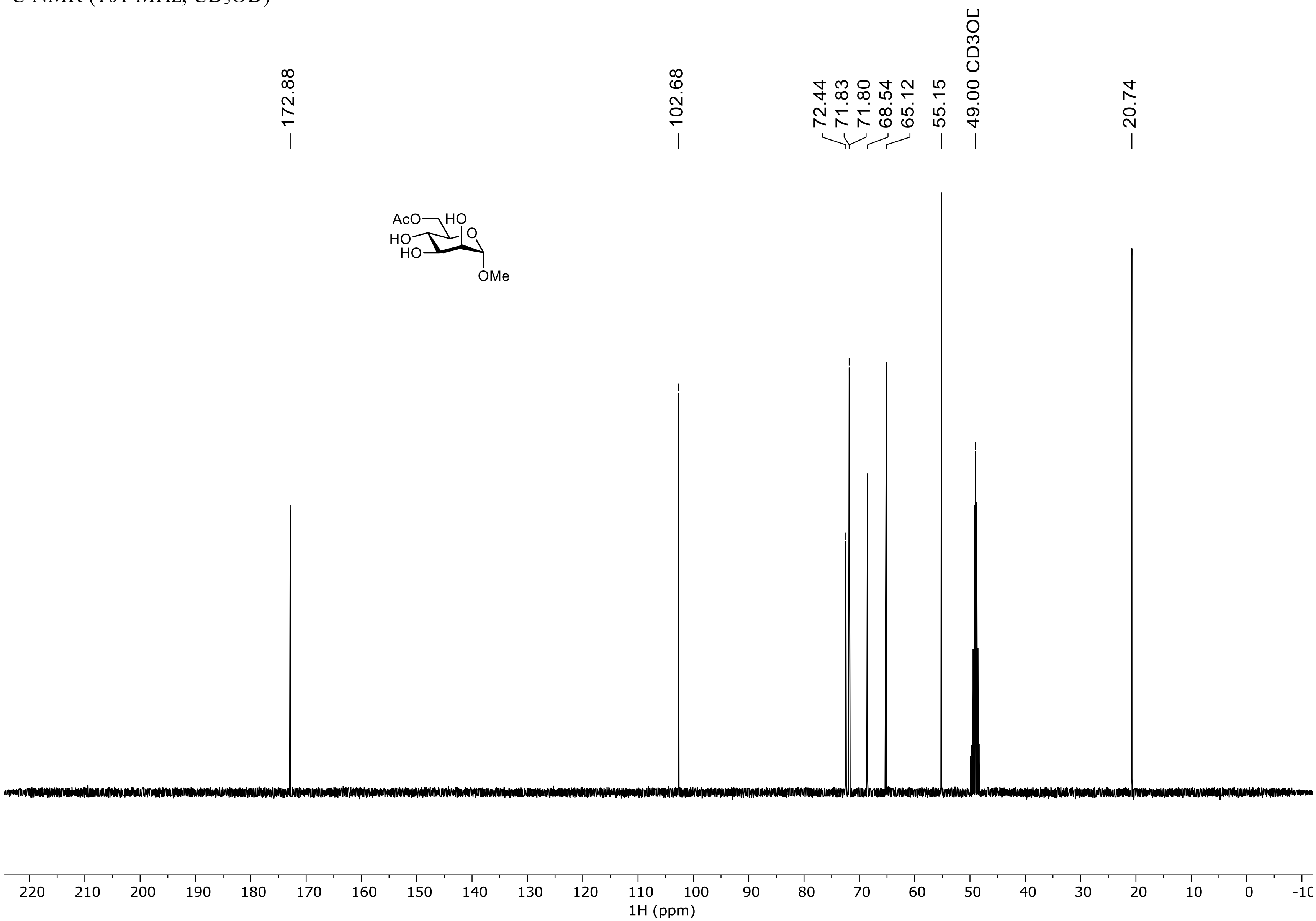

COSY (400 MHz, CD_3OD)

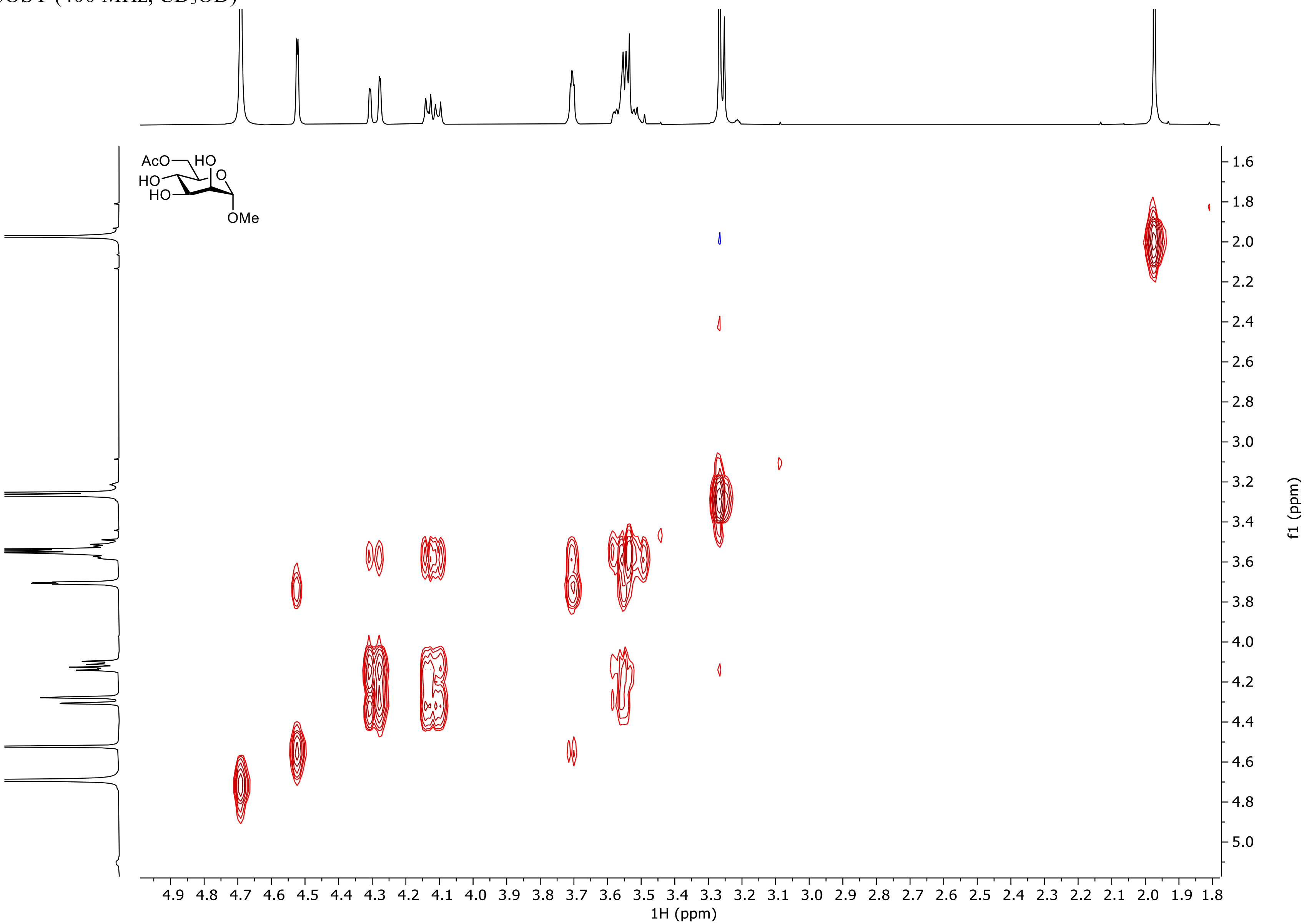

Methyl 2-*O*-acetyl-6-*O*-trityl-α-ᴅ-glucopyranoside (2s)

^{1}H NMR (400 MHz, $CDCl_3$)

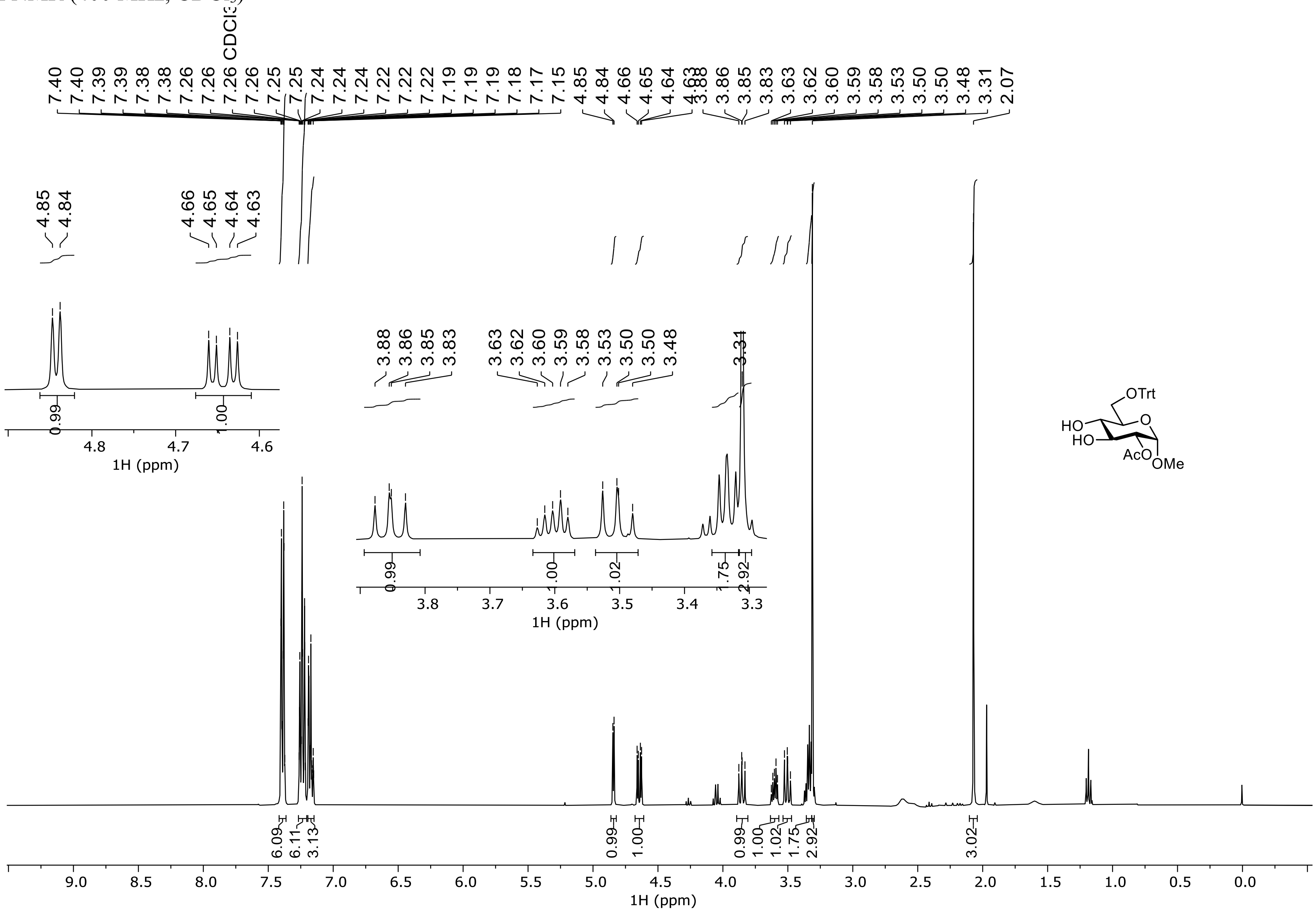

^{13}C NMR (101 MHz, $CDCl_3$)

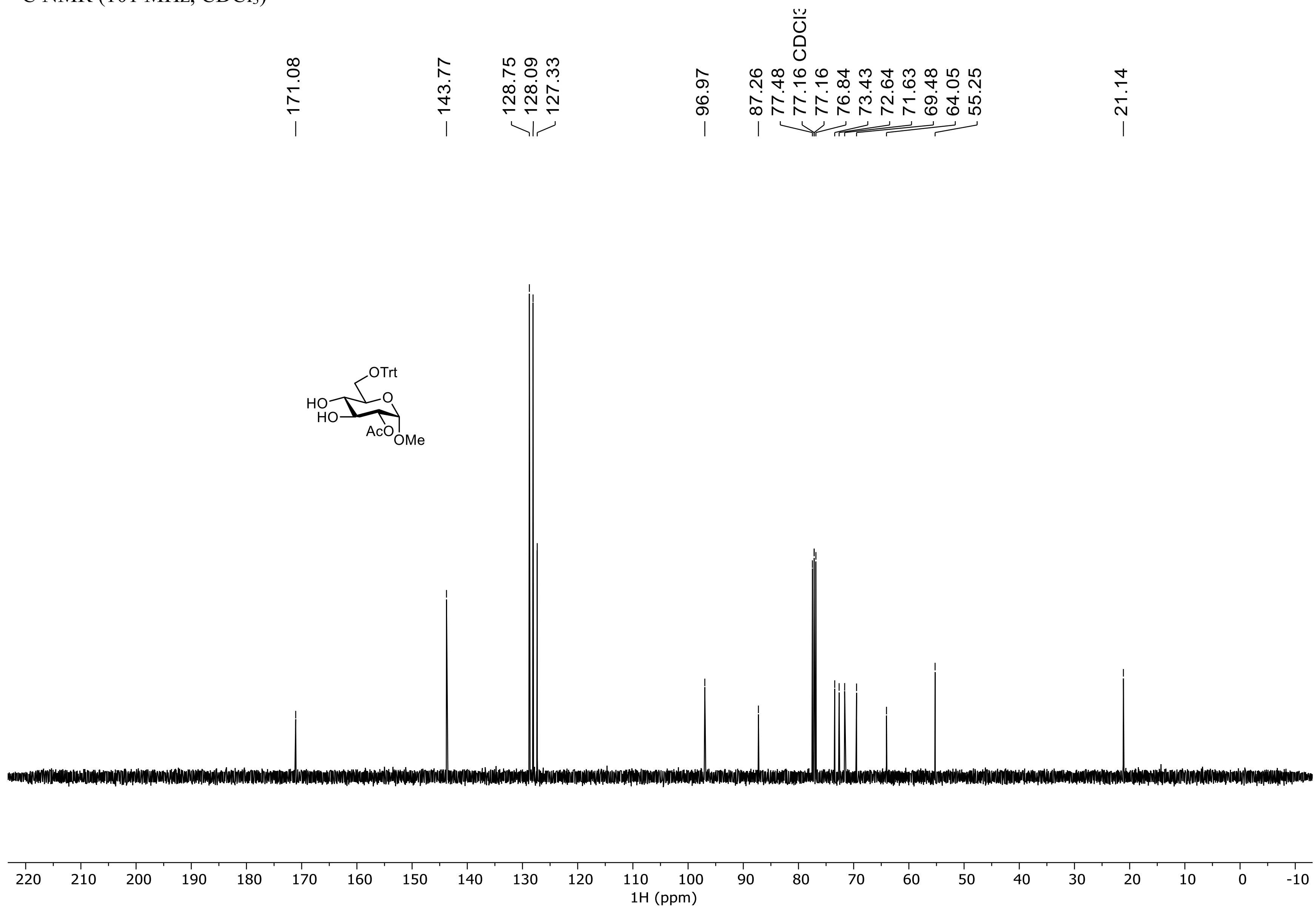

COSY (400 MHz, $CDCl_3$)

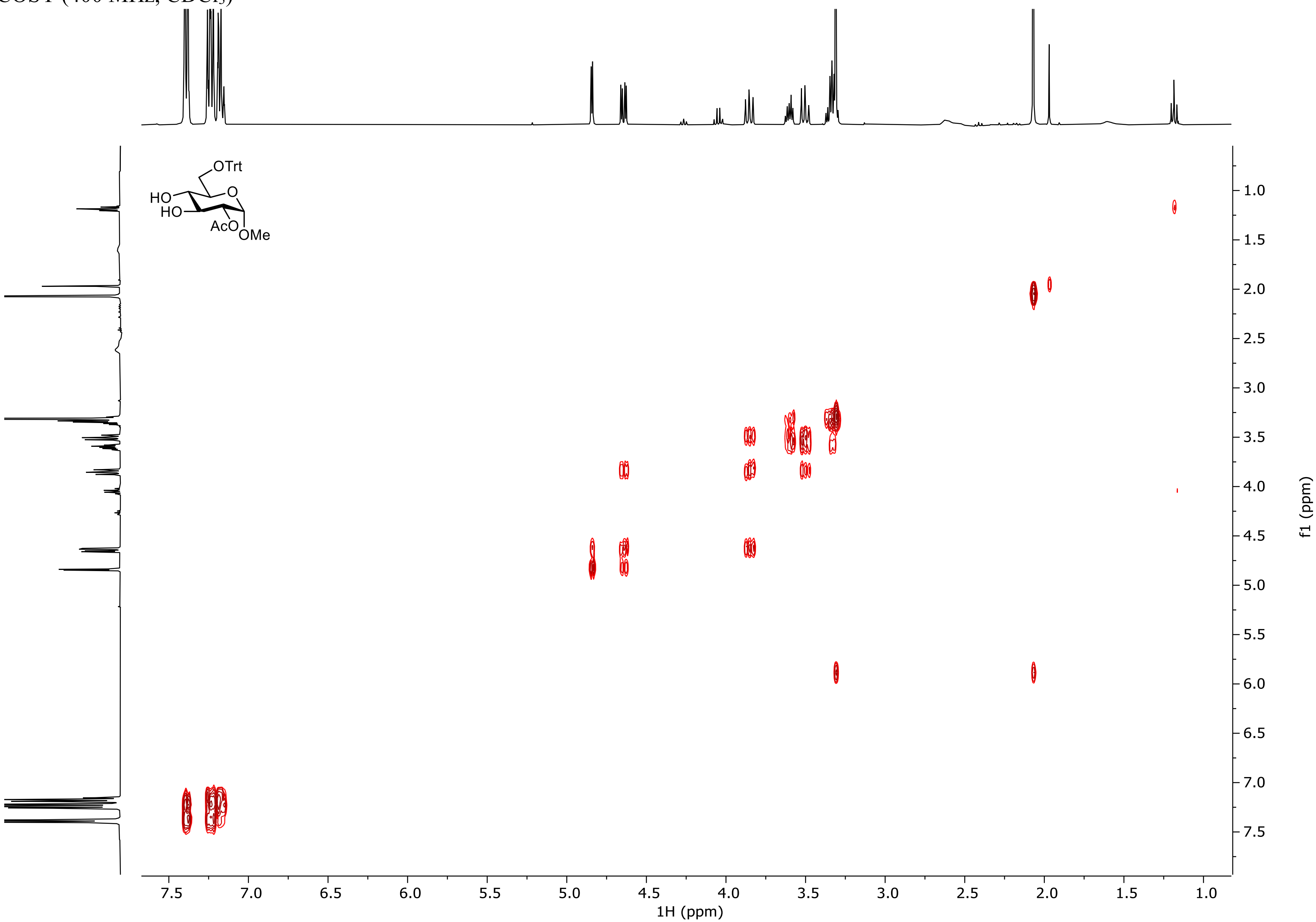

Methyl 4-*O*-acetyl β-D-xylopyranoside (2t)

^{1}H NMR (400 MHz, CD_3OD)

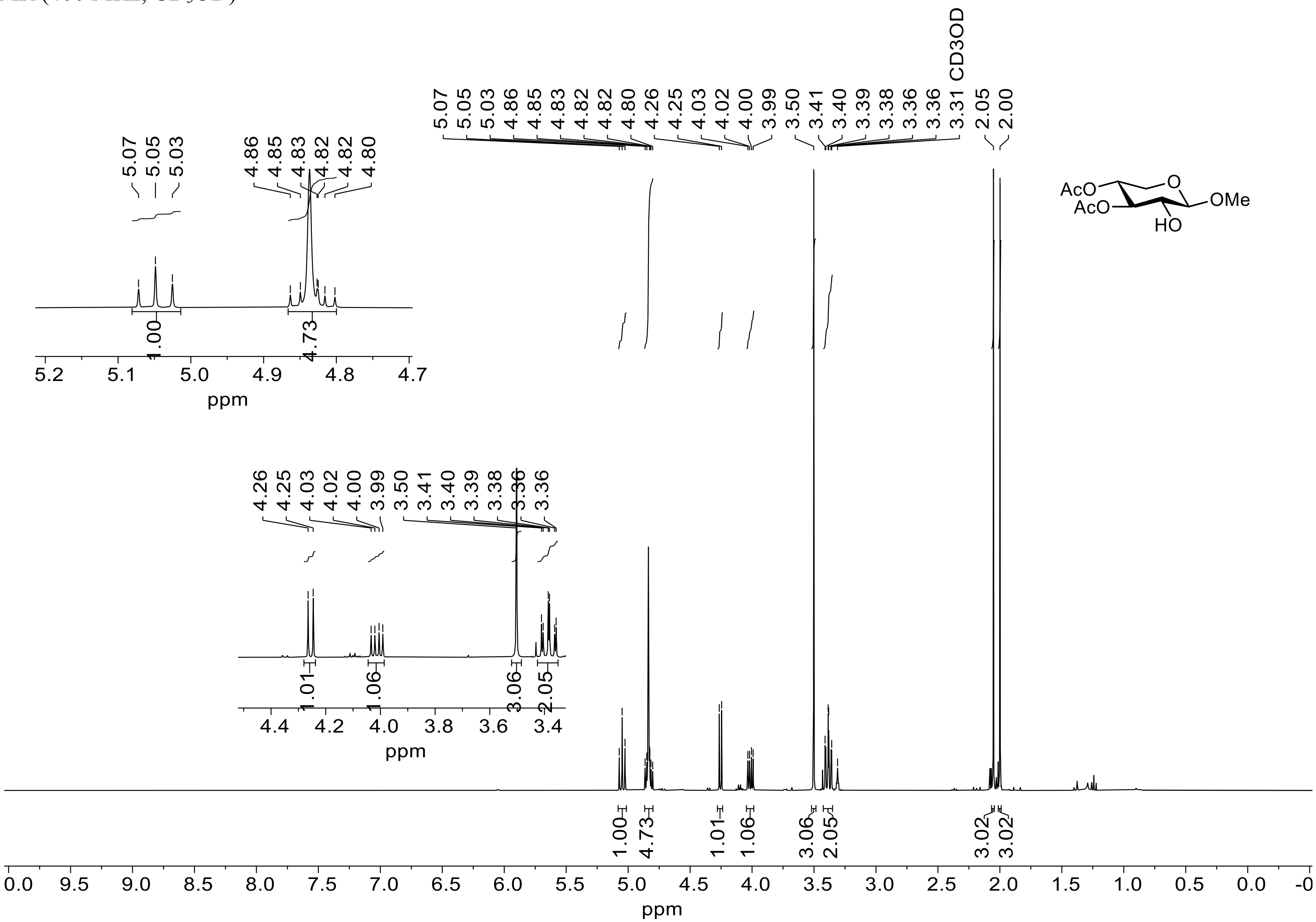

^{13}C NMR (101 MHz, CD_3OD)

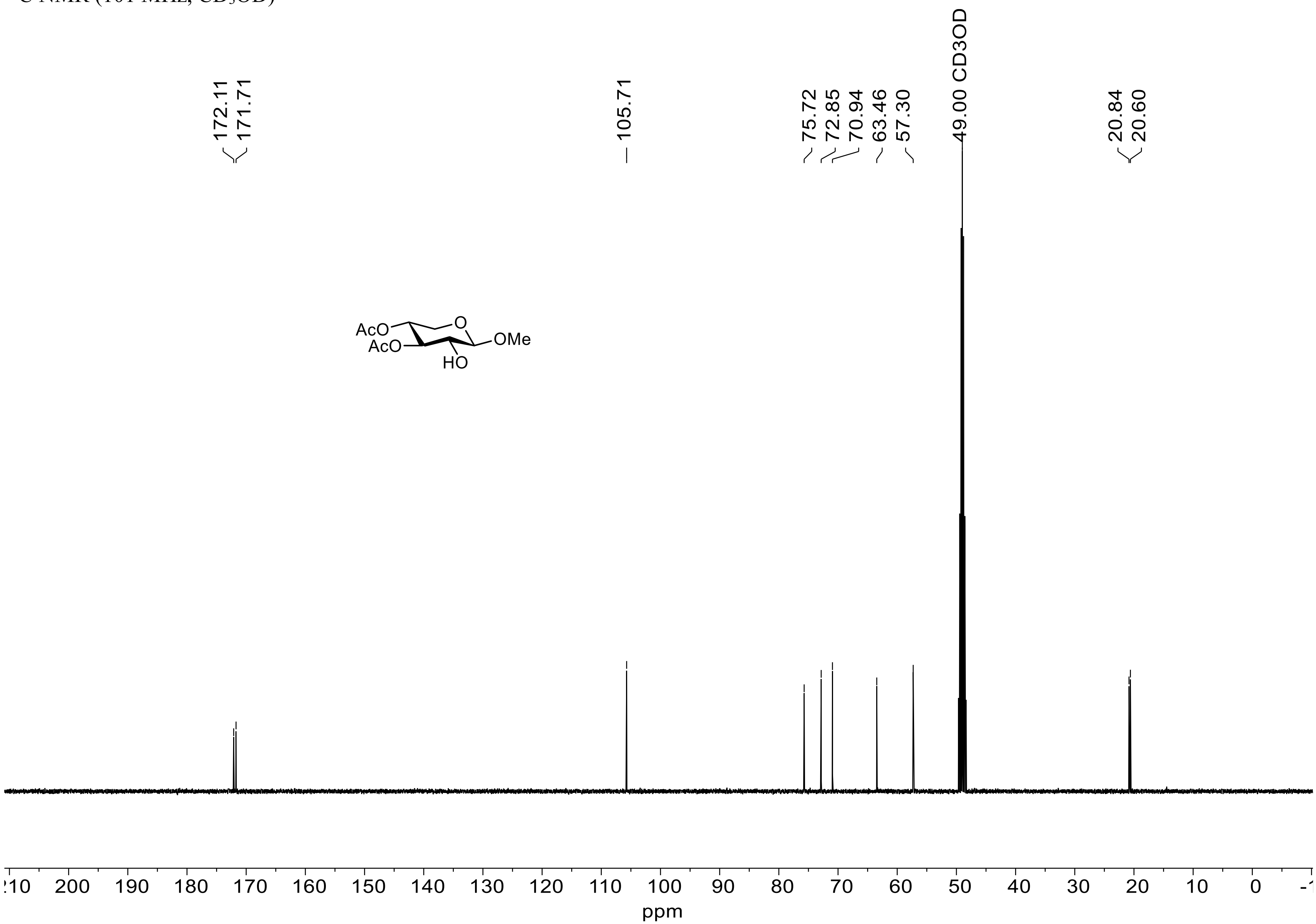

COSY (400 MHz, CD_3OD)

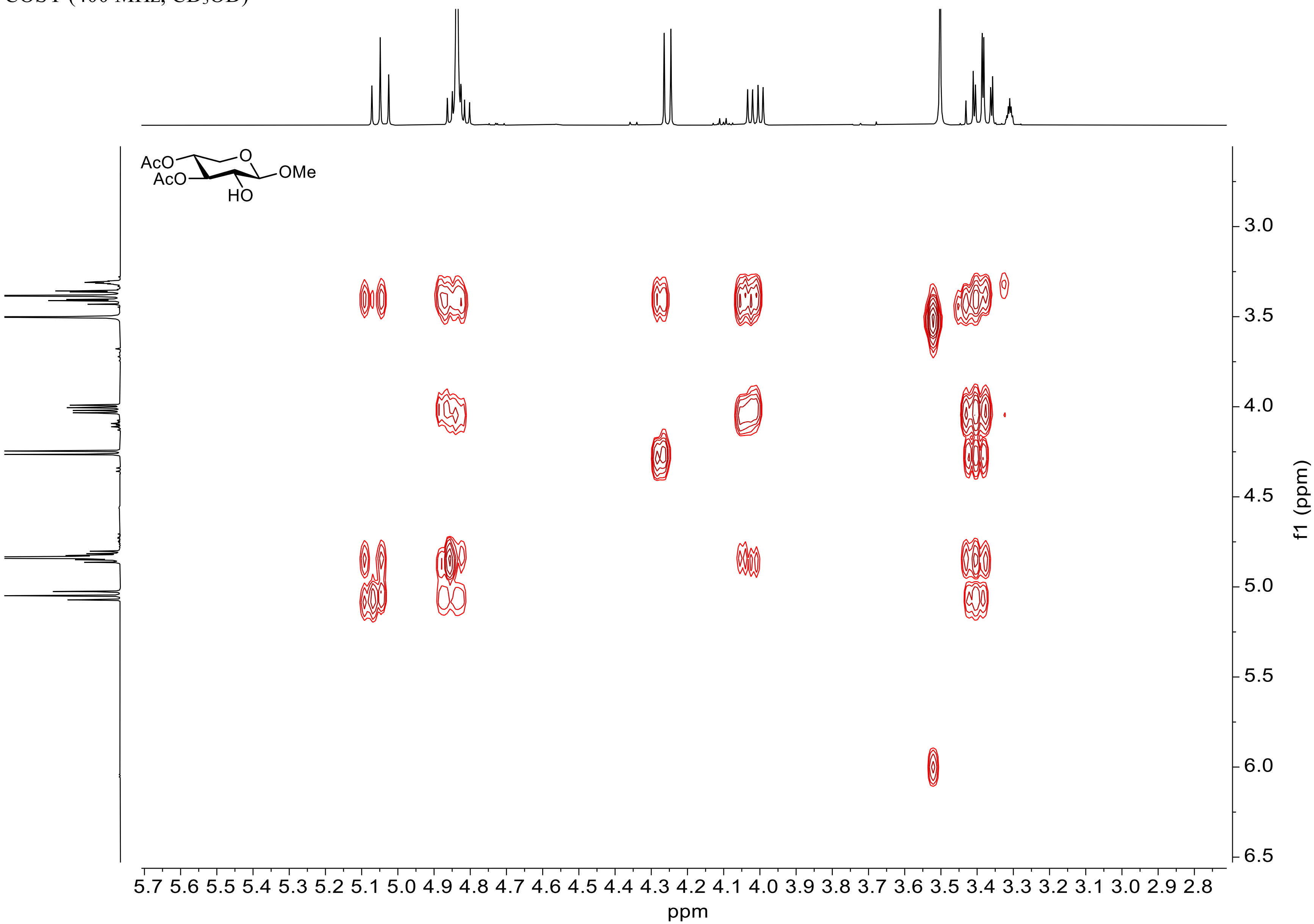

Phenyl 6-O-acetyl-α-ᴅ-1-thio-mannopyranoside (2v)

^{1}H NMR (400 MHz, $CDCl_3$)

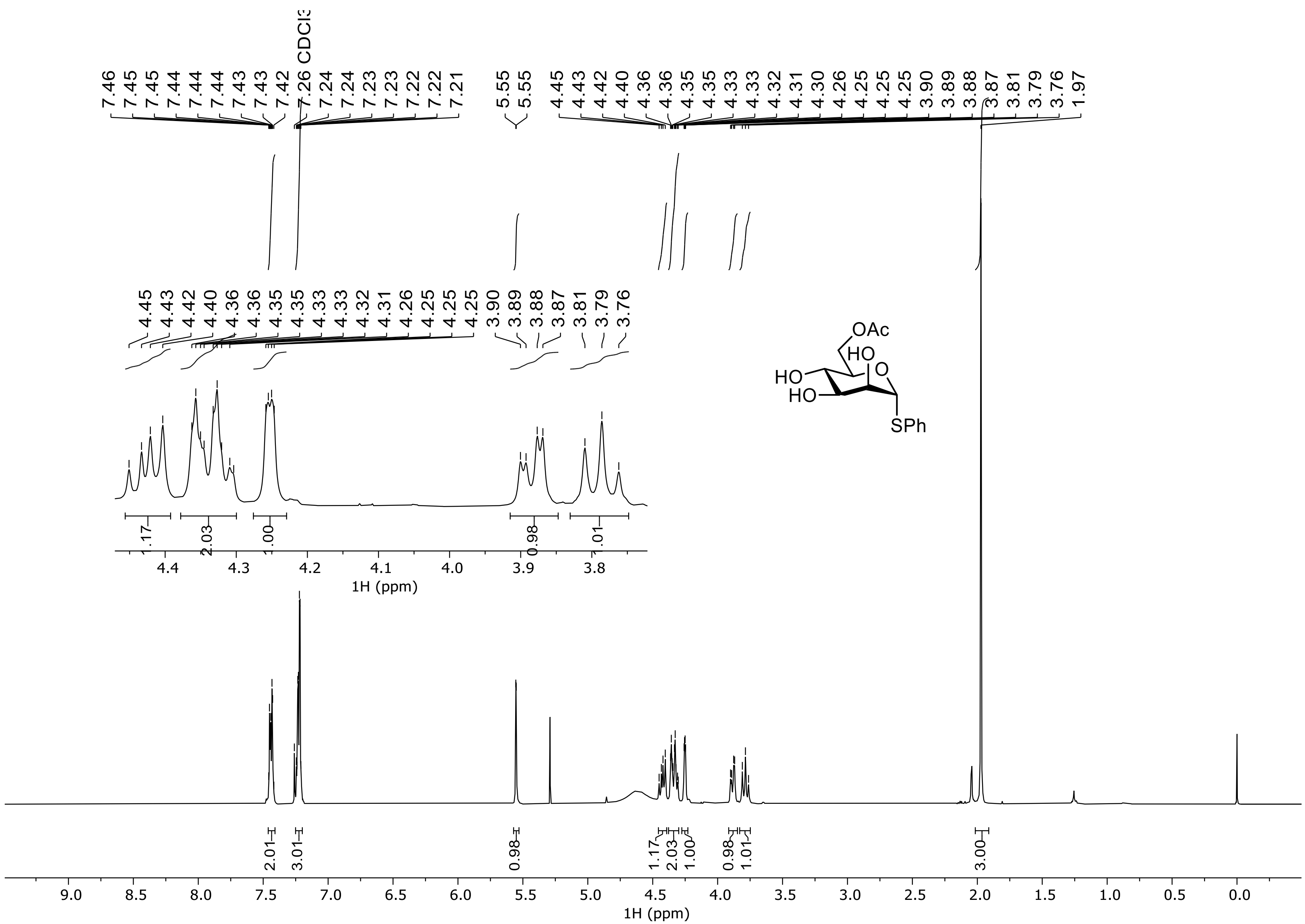

^{13}C NMR (101MHz, $CDCl_3$)

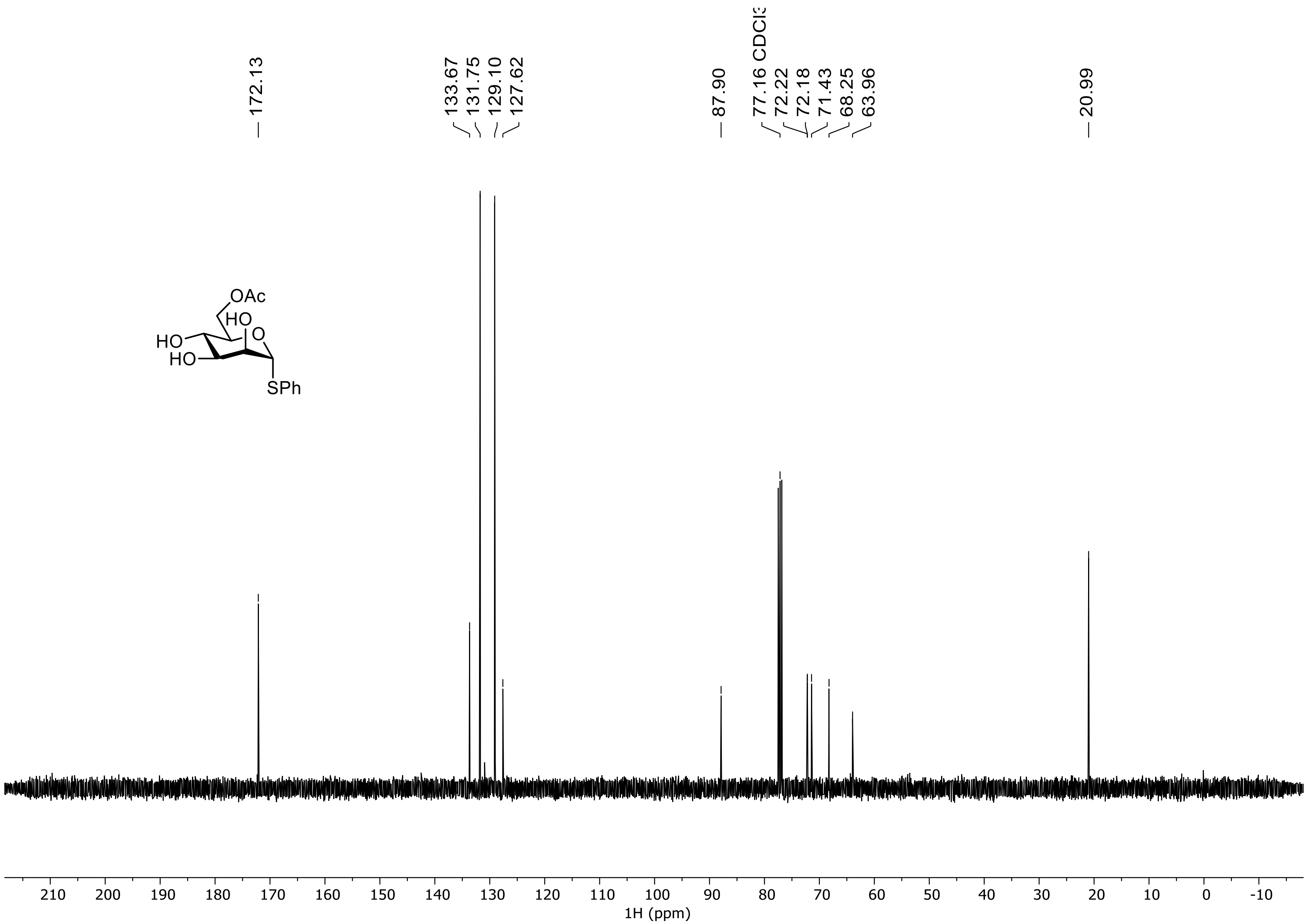

COSY (400 MHz, CDCl$_3$)

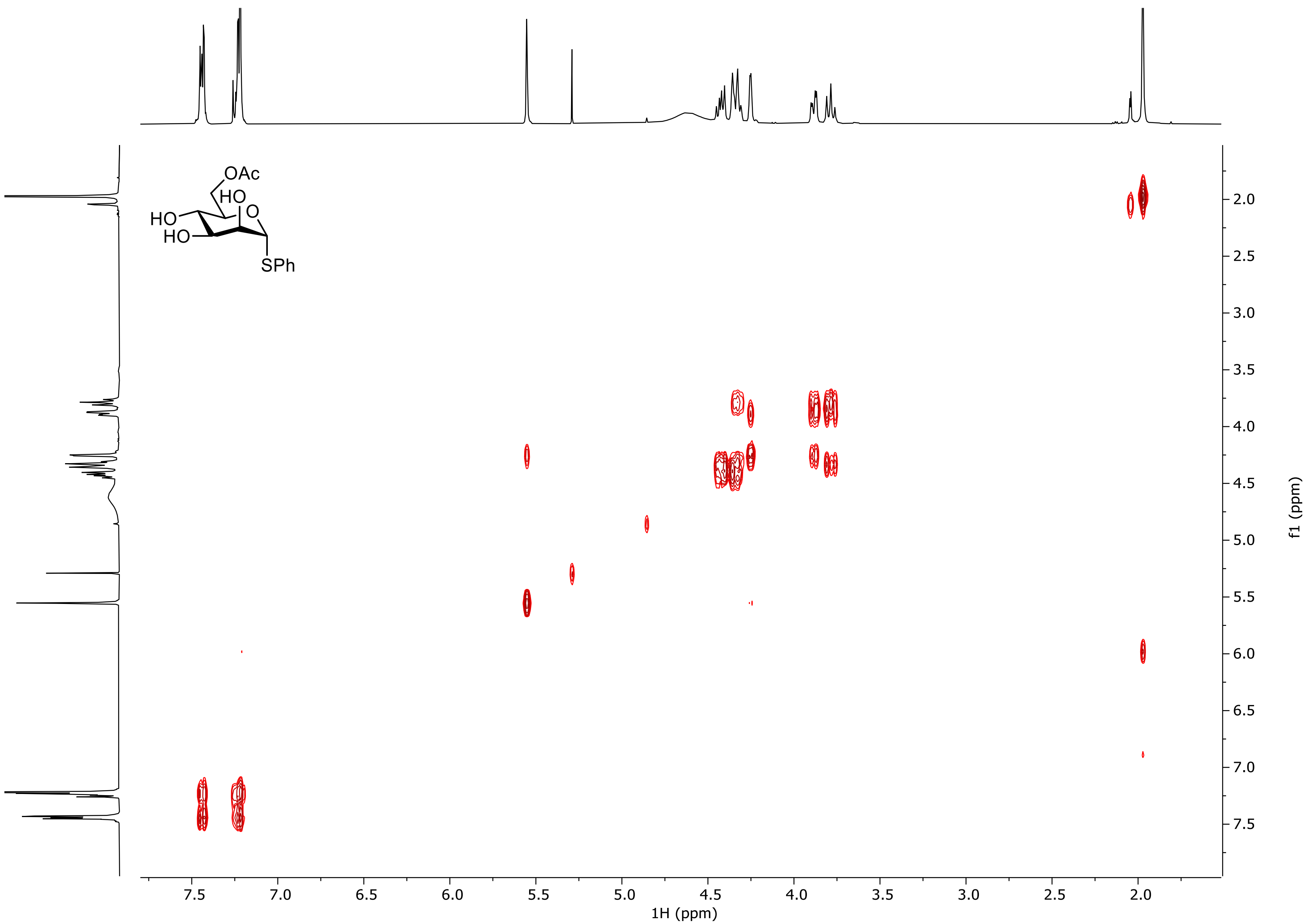

Methyl 2-deoxy-6-O-(tert-butyldimethylsilyl)-α-D-erythro-hexopyranoside-3-ulose (3a)

1H NMR (400 MHz, CD_3OD)

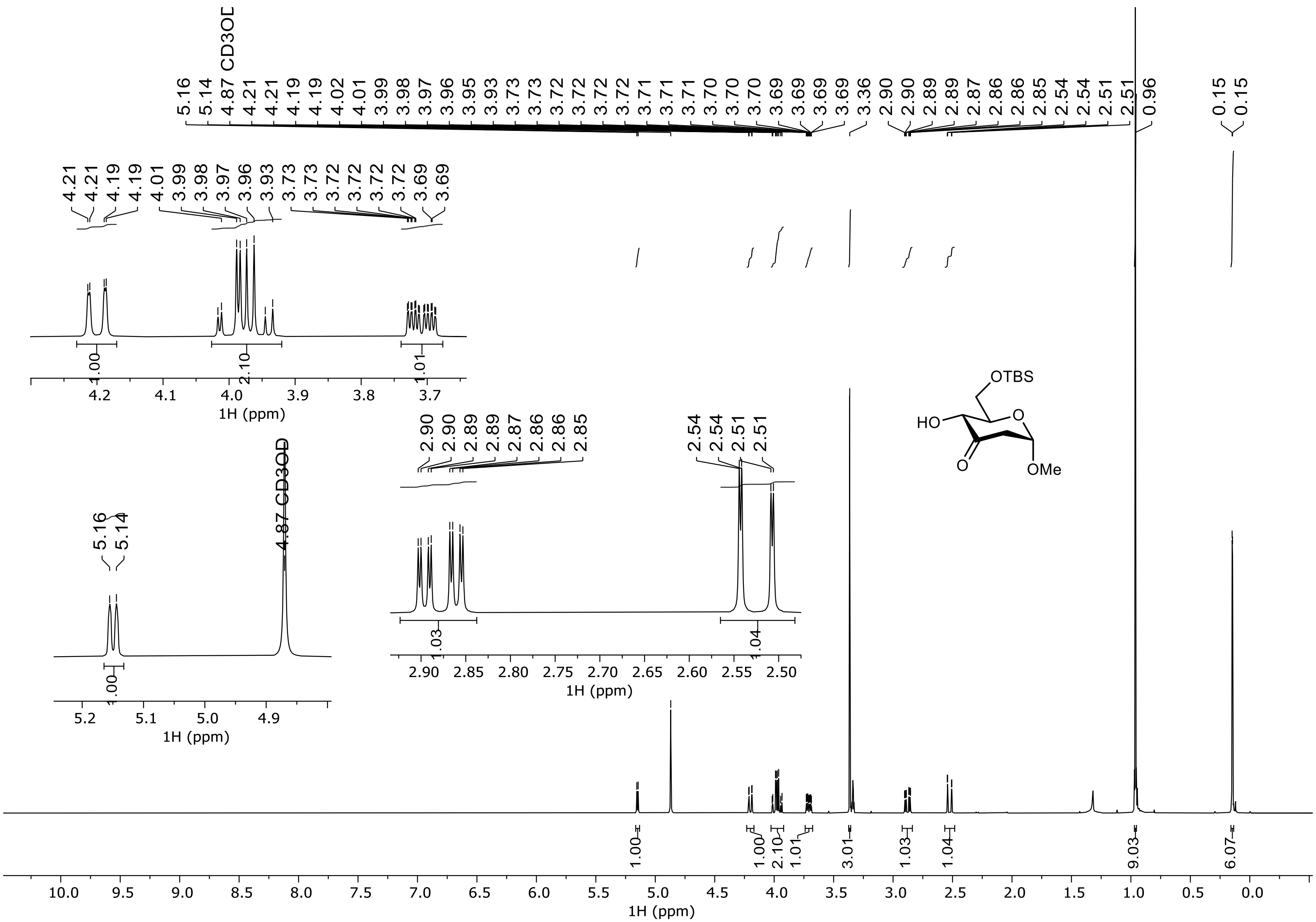

^{13}C NMR (101MHz, CD_3OD)

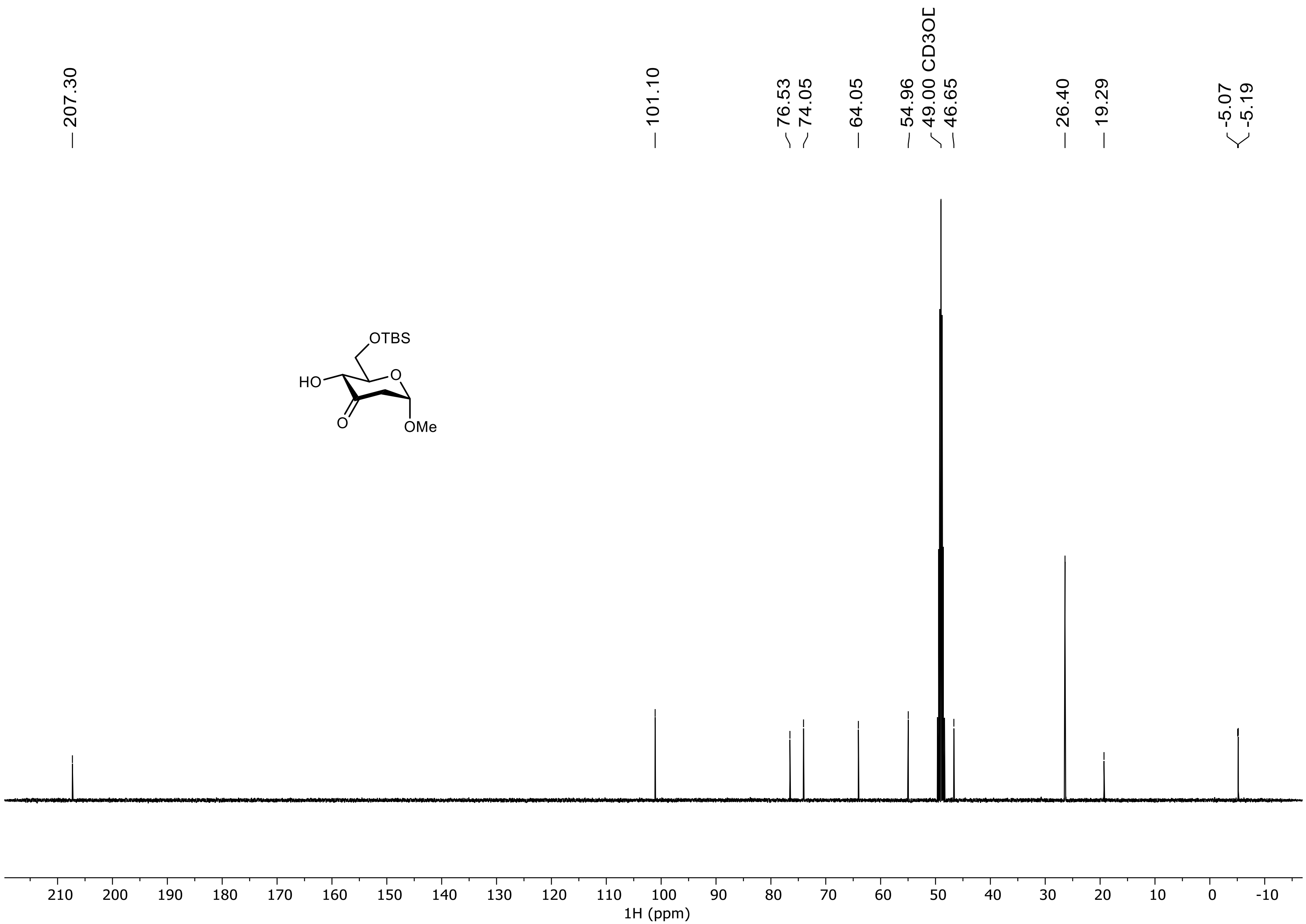

COSY (400 MHz, CD_3OD)

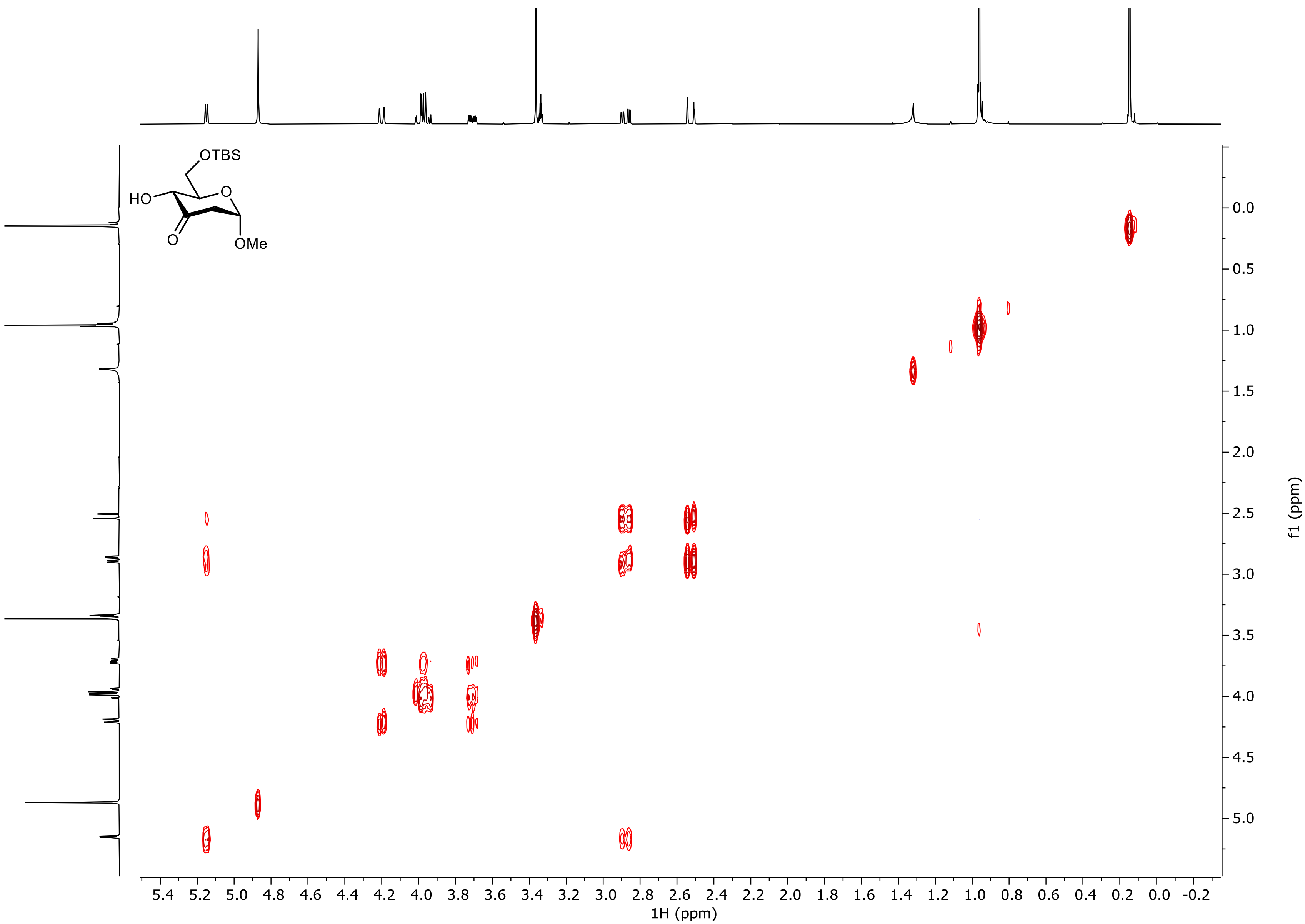

Methyl 4,6-dideoxy-α-D-erythro-hexopyranoside-3-ulose (3m)

^{1}H NMR (400 MHz, CD_3CN)

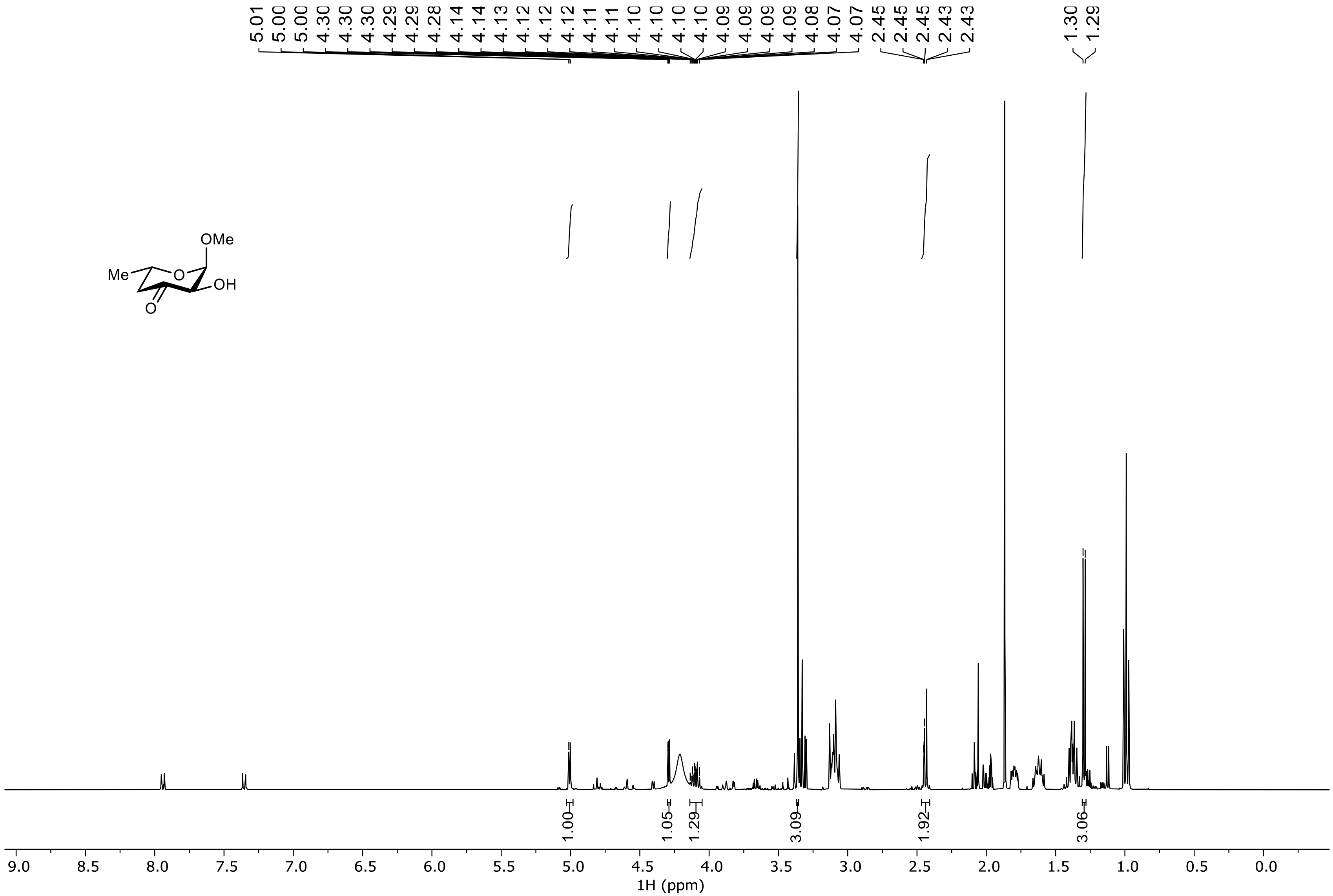

COSY (400 MHz, CD_3OD)

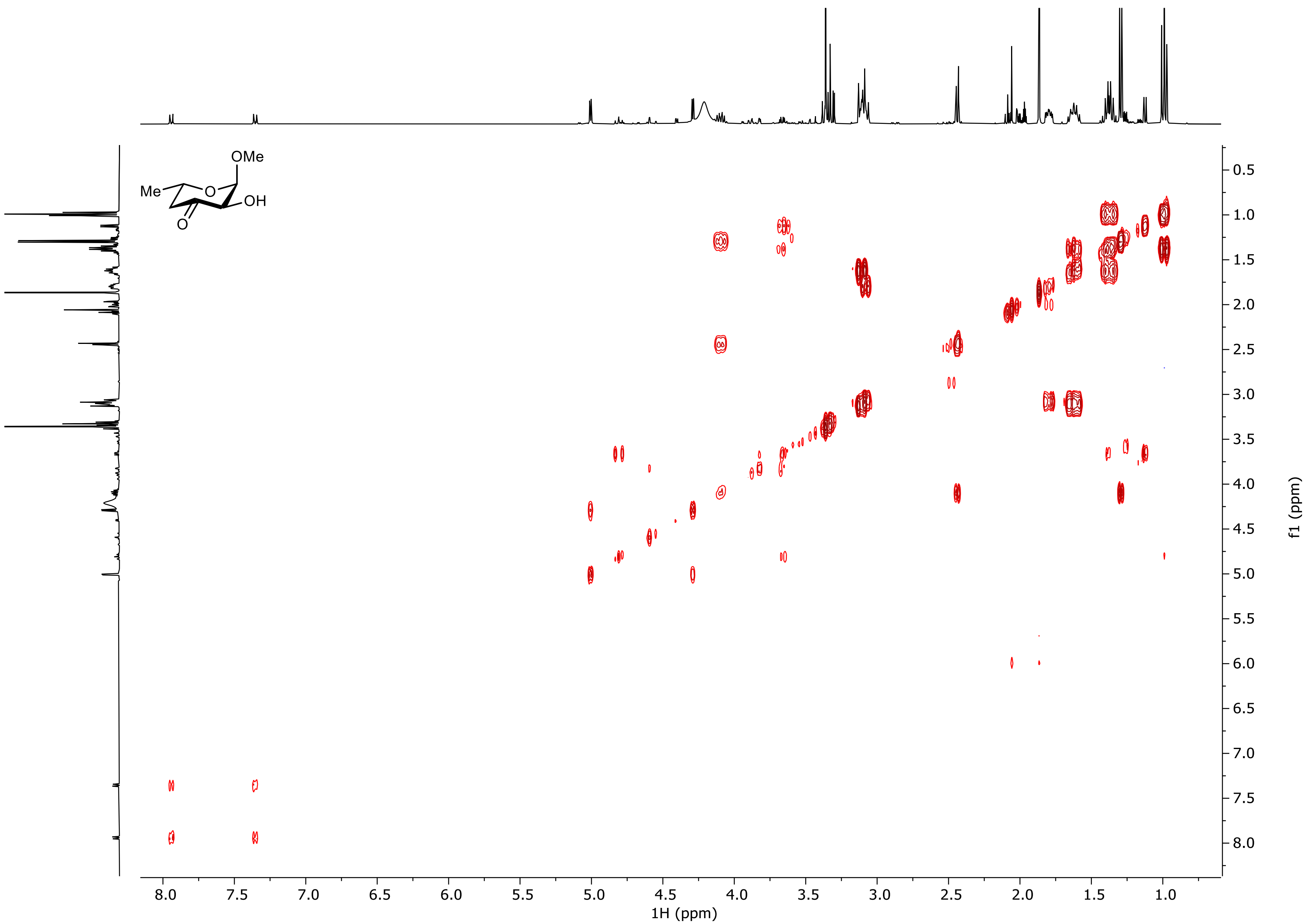

Methyl 4,6-dideoxy-β-D-erythro-hexopyranoside-3-ulose (3j)

^{1}H NMR (400 MHz, CD_3CN)

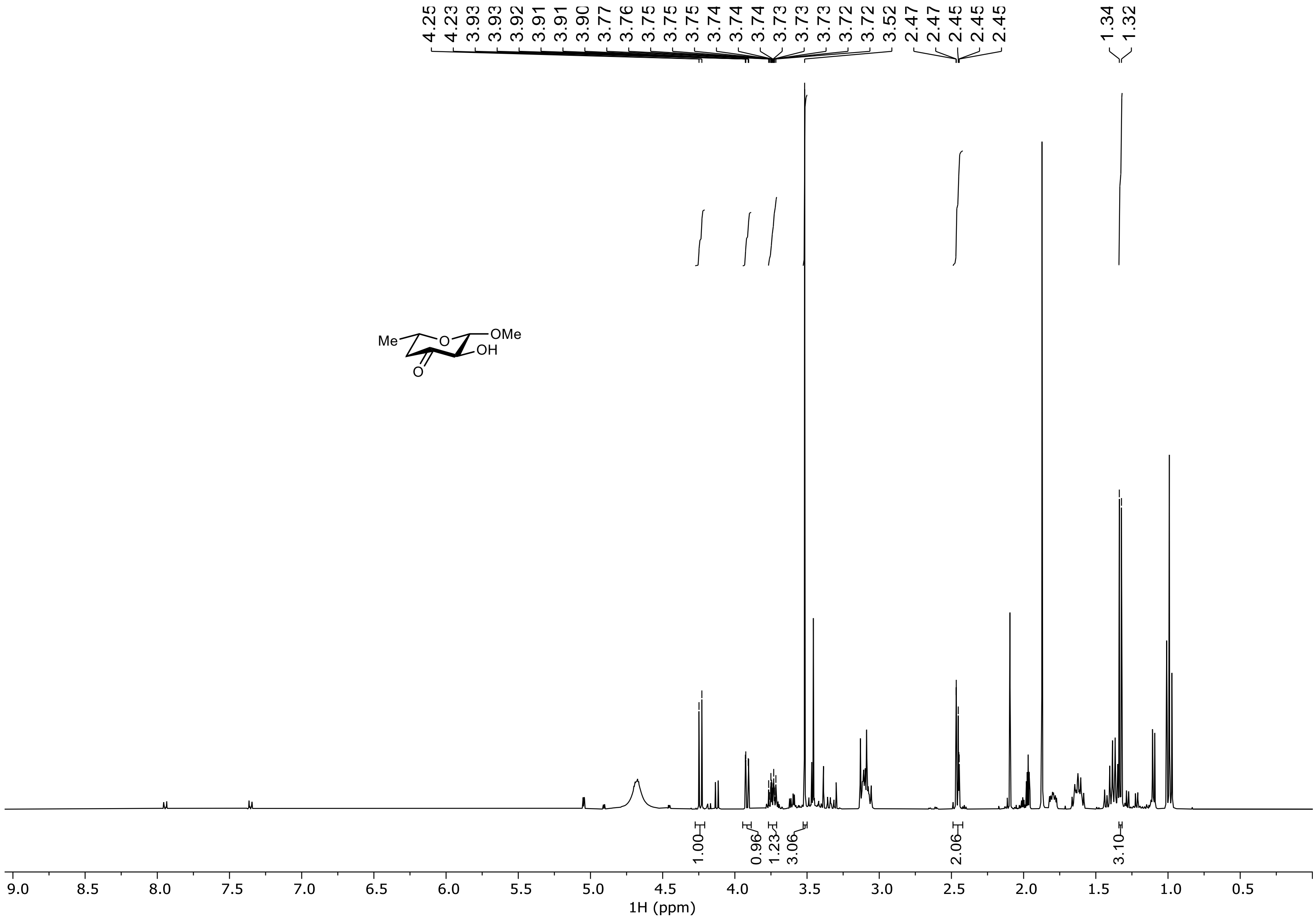

COSY (400 MHz, CD_3CN)

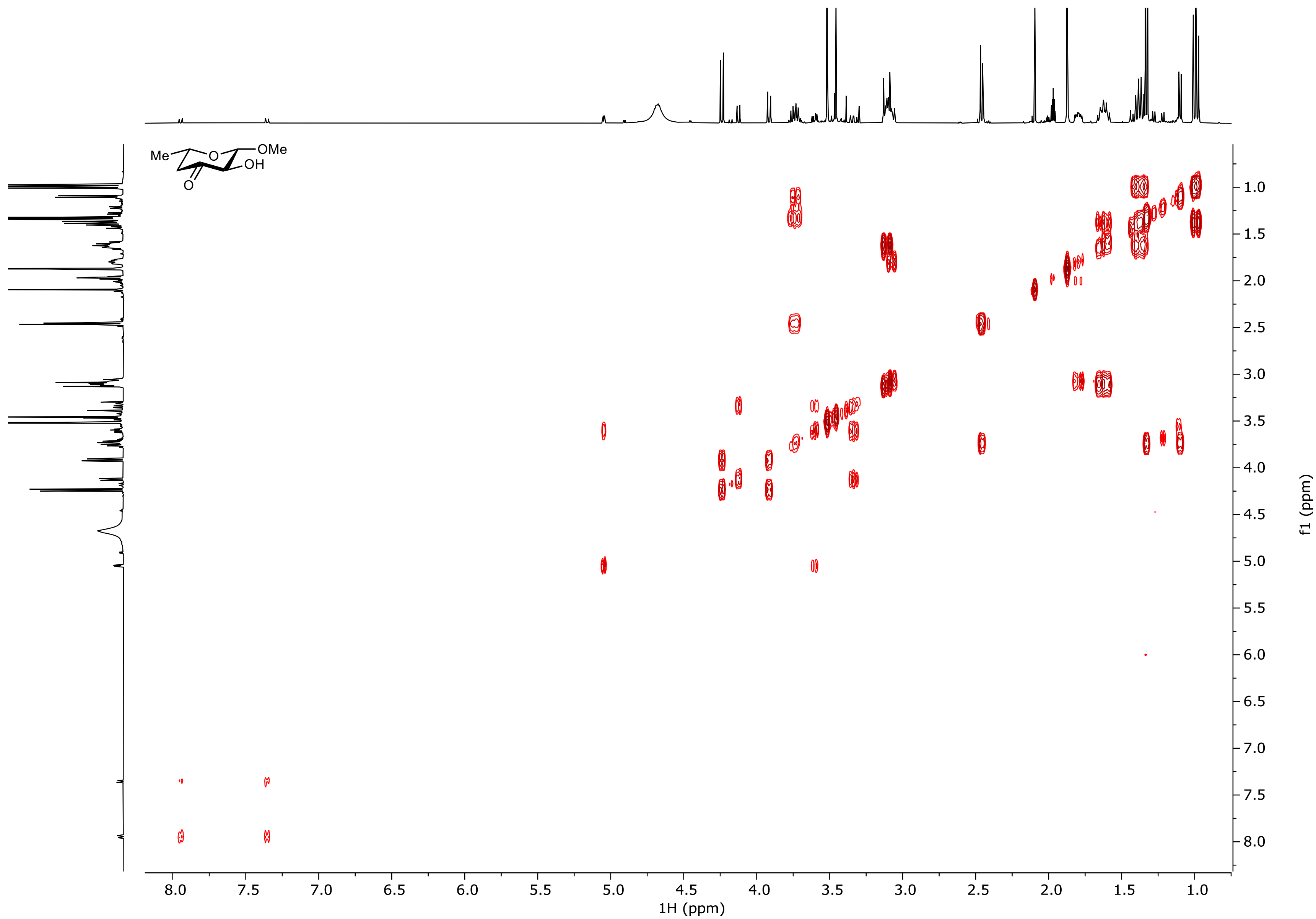

Methyl 2,6-dideoxy-α-D- erythro-hexopyranoside-3-ulose (3i)

^{1}H NMR (400 MHz, CD_3CN)

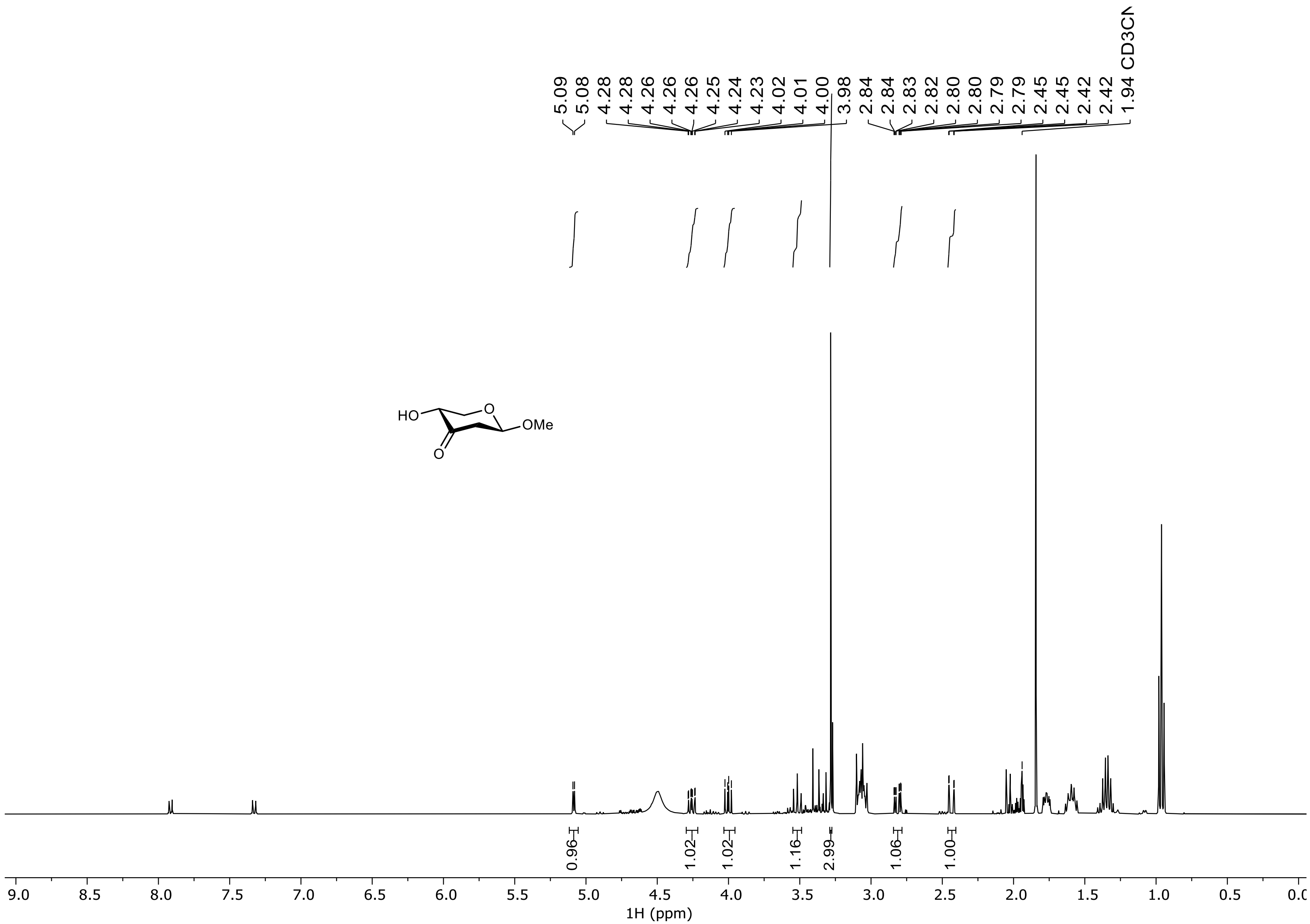

COSY (400 MHz, CD_3CN)

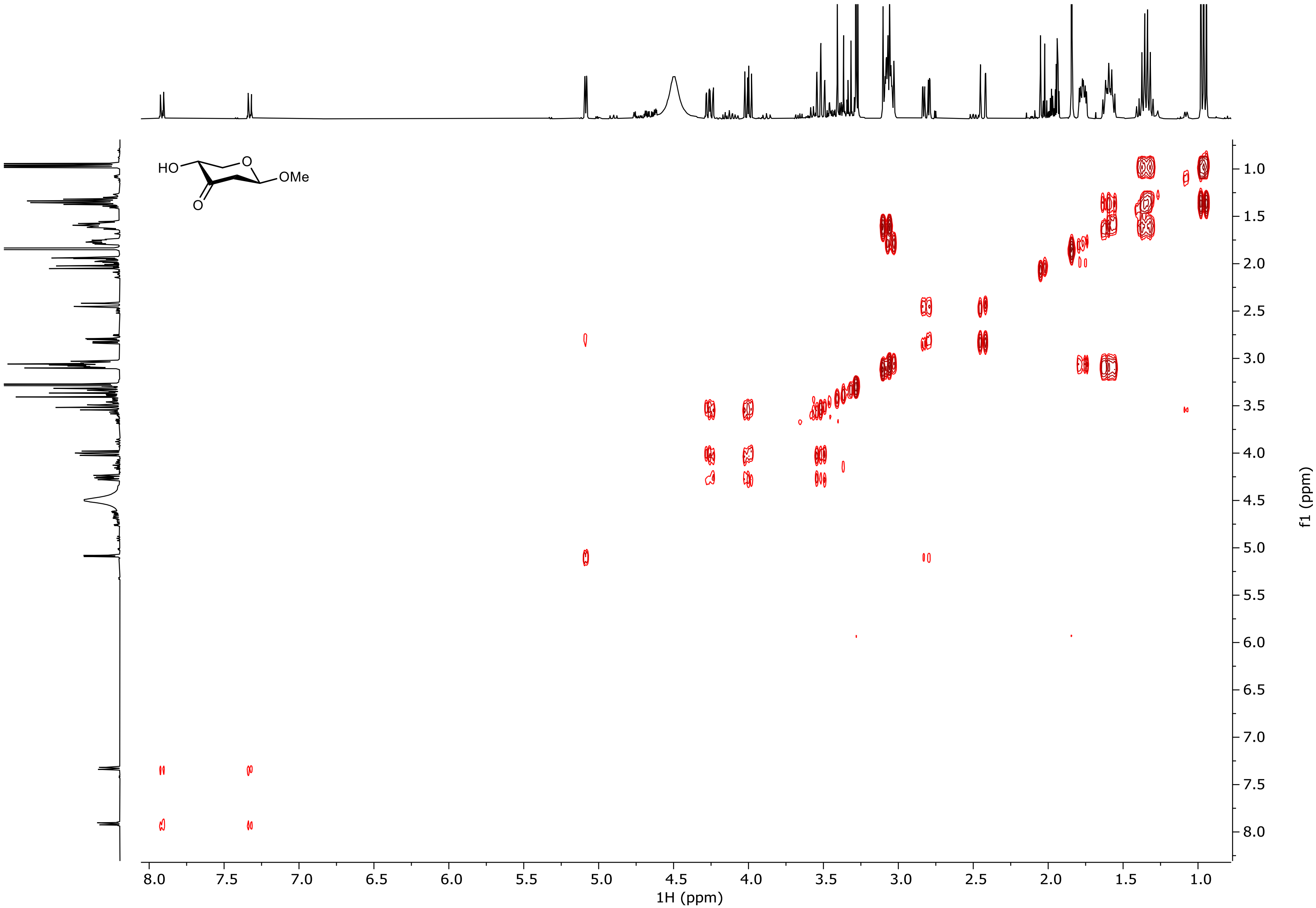

Methyl 5,6-dideoxy-α-D-erythro-hexopyranoside-3-ulose (3l)

^{1}H NMR (400 MHz, CD_3CN)

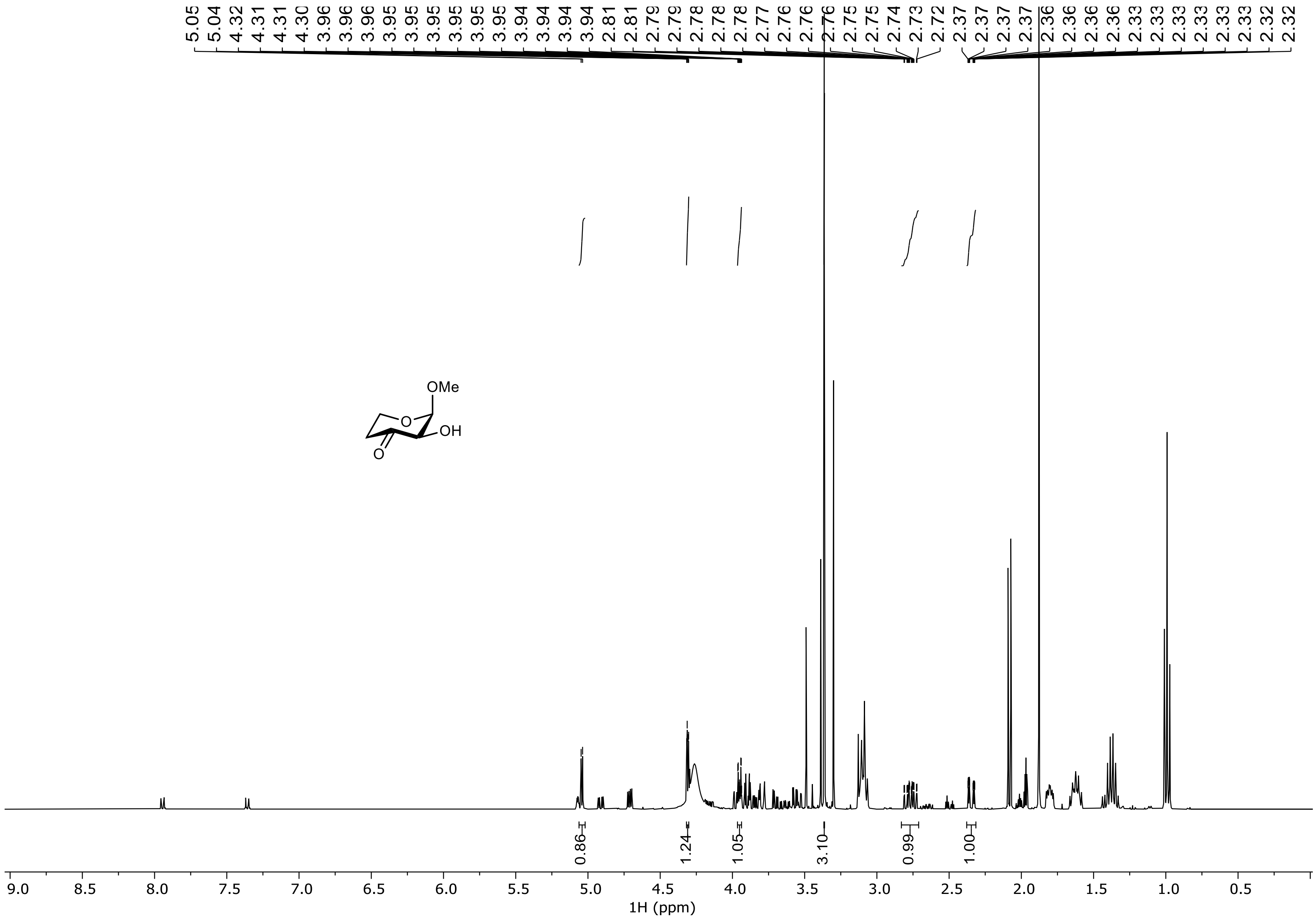

(400 MHz, CD_3CN)

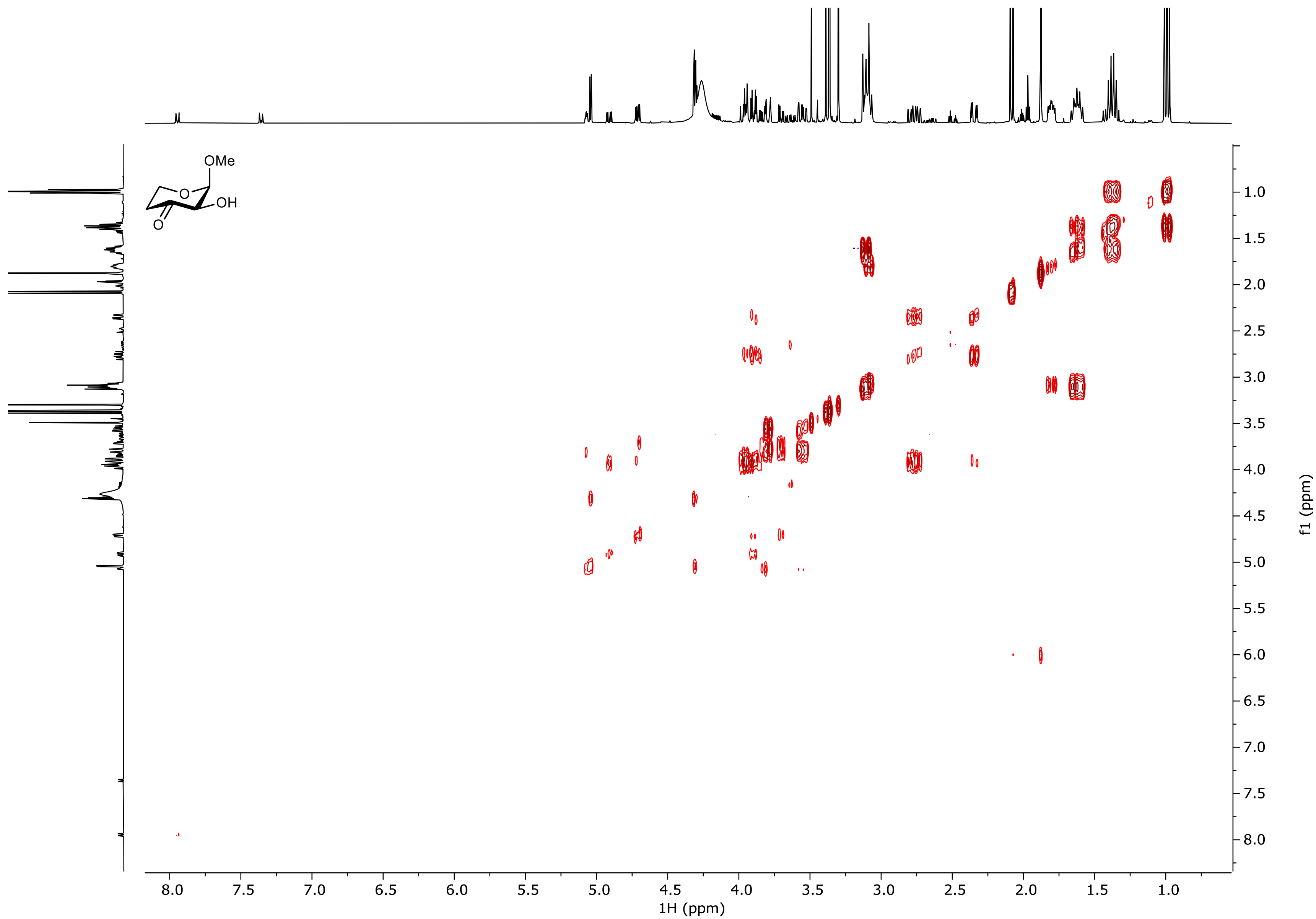

Supplement: Supplementary file 1 [file SC-OLF-D6SC06368C-s001.pdf]
